# Digital twins are funhouse mirrors: Five systematic distortions

**DOI:** 10.1126/sciadv.aeh8260

**Published:** 2026-09-02

**Authors:** Tianyi Peng, Melanie Brucks, George Gui, Daniel J. Merlau, Grace Jiarui Fan, Malek Ben Sliman, Eric J. Johnson, Abdullah Althenayyan, Silvia Bellezza, Dante Donati, Hortense Fong, Elizabeth Friedman, Ariana Guevara, Mohamed Hussein, Kinshuk Jerath, Bruce Kogut, Akshit Kumar, Kristen Lane, Hannah Li, Vicki Morwitz, Oded Netzer, Patryk Perkowski, Olivier Toubia

**Affiliations:** ^1^ Columbia University, New York, NY 10027, USA.; ^2^ Yale University, New Haven, CT 06511, USA.; ^3^ Yeshiva University, New York, NY 10033, USA.

## Abstract

Scientists and practitioners are aggressively moving to deploy digital twins—large language model (LLM)-based models of individuals—across social science and policy research. We conducted 19 preregistered studies with 164 diverse outcomes (e.g., attitudes toward hiring algorithms and intention to share misinformation) and compared human responses with those of their digital twins (trained on each person’s previous answers to more than 500 questions). We establish an empirical benchmark for digital twin performance: Digital twins’ answers are only modestly more accurate than those from the (homogeneous) base LLM and correlate weakly with human responses (average correlation coefficient of 0.20). To guide future development, we document five ways in which digital twins distort human behavior: (i) insufficient individuation, (ii) stereotyping, (iii) representation bias, (iv) ideological biases, and (v) hyper-rationality. We make our full dataset and code public as a standardized testbed. Our results caution against premature deployment while laying the groundwork for the transparent, replicable, and iterative science necessary for responsible deployment of digital twins.

## INTRODUCTION

Large language models (LLMs) hold the capacity to fundamentally transform psychological measurement by overcoming the long-standing cognitive, ethical, and practical barriers inherent to human participant research (*1*–*4*). Through the scalable simulation of human responses, LLMs offer not only faster and cheaper data collection but also potentially “better” data for researchers and managers: LLMs do not tire, can be repeatedly surveyed, and can simulate high-stakes or potentially harmful scenarios without ethical risk. Although LLMs can show human-like behavior (*5*, *6*) and directionally replicate experimental treatment effects (*4*, *7*), many researchers have expressed skepticism, both on theoretical and empirical grounds (*8*–*12*). Studies have shown that LLMs often struggle to reproduce human opinions (*13*–*15*), tend to lack heterogeneity (*16*), and overestimate experimental effect sizes (*7*).

To address these concerns, experts and practitioners alike have proposed using digital twins: base LLM models (e.g., GPT and Llama) augmented with rich personal information to simulate specific individual behavior (*17*). At first glance, digital twins present a remarkably elegant solution, offering what appears to be a panacea to the foundational limitations documented in prior synthetic data research. First, distinct, tailored models offer the potential to infuse human variance into responses, overcoming the homogenization that often plagues general models (*16*). Second, anchoring the model in rich and real human data may provide the detail necessary to prevent hallucinations or confabulations and address default sycophancy and normativity that undermines base model accuracy (*7*, *10*, *11*, *13*). Together, this would allow for “simulations that better reflect the myriad, often idiosyncratic, factors that influence individuals’ attitudes, beliefs, and behaviors” (*17*). The allure of digital twins is further amplified by the unique applications they unlock, from agentic artificial intelligence (AI), e.g., negotiating, working, or dating on one’s behalf, to self-reflection, where individuals interact with digital copies of themselves.

Given these promises, digital twin applications are being frantically built, deployed, and sold across academia and industry. For example, the startup simile.ai, based on (*17*), recently raised $100 million, joining a growing list of synthetic data startups with valuations in the order of $1 billion (e.g., Aaru).

Despite fervent interest, the empirical foundation of digital twins remains remarkably thin. To date, only two initial studies have directly examined whether twins can serve as human surrogates. One study created digital twins of more than 1000 individuals on the basis of transcripts from 2-hour interviews (*17*) and reported encouraging results (relative accuracy of 85% on the General Social Survey, based on the ratio of digital twin accuracy to test-retest accuracy). However, the data are not publicly available, presenting a substantial hurdle to the scientific understanding of digital twins. The second study developed Twin-2K-500, a public dataset permitting the creation of digital twins of more than 2000 individuals based on their answers to more than 500 questions (*18*) and reported relatively high accuracy of digital twins on holdout data (average accuracy of 72%, relative accuracy of 88% based on the ratio of digital twin accuracy to test-retest accuracy). As a first test, however, it was necessarily limited in scope: Twins were not compared against benchmarks such as demographic-only personas. Furthermore, twins only replicated about half the experimental effects in humans. In addition, in both studies (*17*, *18*), validation was based on a relatively narrow set of well-known experiments and surveys, which may overlap with the training data of the base model, raising concerns of leakage (*19*). Consequently, the field is rapidly adopting a technology that lacks essential empirical validation, leaving open the risk of making consequential decisions based on an illusion of personalization rather than genuine individual-level insight.

We conducted a large-scale, preregistered mega-study to address this urgent empirical gap. We assembled 23 coauthors from various backgrounds within social science, each curious to test the validity of digital twins in their domain of interest. Together, we jointly designed and ran the most comprehensive empirical test of digital twins to date, with 19 new preregistered (https://researchbox.org/4145) substudies covering 164 diverse outcomes (cumulative sample size of *n* = 13,506 human participants across substudies and 1784 unique participants). We matched each human answer to their digital twin’s answer to the same question. To give digital twins their best shot, we conducted our study on the validated Twin-2K-500 human sample and their corresponding digital twins. We invited the original Twin-2K-500 participants to complete entirely new studies [including unpublished stimuli; available in the Supplementary Materials (SM)] and then compared their answers against their twins’. This mega-study, unprecedented in scale and scope, presents an earnest, transparent, and replicable empirical benchmark of digital twin performance.

Our evaluation demonstrated underwhelming performance overall. While digital twins showed revealing variance across domains and individuals, they largely failed to faithfully mimic human responses. We also gathered (preregistered) human expert predictions on twin performance to gauge relative understanding of this nascent technology. We find that these experts generally failed to predict the behavior of digital twins (see the SM), underscoring the value of empirical tests.

In light of these disappointing results, we conducted exploratory (non-preregistered) analyses to identify how digital twins distort human behavior and therefore how they may be improved. We document five distortions: (i) insufficient individuation, (ii) stereotyping, (iii) representation bias, (iv) ideological biases, and (v) hyper-rationality. These distortions are generally consistent with previous literature on synthetic data, suggesting that state-of-the-art digital twins are not yet able to fully resolve known limitations of synthetic data. Without actively addressing these distortions, the premature deployment of twins risks systematically misrepresenting human cognition in ways that could undermine both scientific understanding and practical applications.

Together, this work advances the scientific foundation of digital twins on three fronts. First, we establish an empirical baseline for where the technology stands today, grounding a field thus far driven more by promise than evidence. Second, by consolidating disparate prior findings into a unified evaluative framework, we offer a roadmap for the improvement of digital twins and a clear set of indicators on which to evaluate them. This enables more targeted improvements in future digital twin research and direct comparisons across methodologies. It is important to note that these improvements are necessary but not sufficient conditions for substituting digital twins for human participants. That is, our study is an empirical, “heuristic validation” (*20*) of claims that digital twins can be substituted directly for human participants. It is motivated by how many are assuming that digital twins can be substituted directly for human participants, not to exemplify how we should plan to decide if we can trust them in the future. Third, by releasing our full dataset and code, we provide a standardized testbed for the improvement of digital twin pipelines. By quantifying current performance, establishing clear, standardized metrics and benchmarks for safety and accuracy, and engaging in open science practices, we enable transparent, replicable, and iterative science necessary to move this technology forward to the point where it can be deployed responsibly in both academia and industry.

### Empirical context

Our studies cover a wide range of domains and topics including creativity, politics, privacy preferences, storytelling, fairness perceptions, interactions with technology platforms, luxury consumption, news consumption, and labor market preferences, among others. Unlike prior work that primarily examined digital twins’ performance in published studies (*17*, *18*), our substudies combined established paradigms from published research, new paradigms from unpublished research, and newly designed studies. Our contexts range from behavioral economics paradigms to personality scale development and include correlational, within-participant, and between-participant designs. Together, they span a considerably broader and richer scope of use cases than any paper to date, providing an ecologically valid test of digital twins as they are available today.

To create each digital twin, we used in-context learning on the basis of answers to more than 500 questions (approximately 128K characters) provided by the twin’s human counterpart. These questions covered demographics (14 questions), personality traits (279 questions from 19 personality tests measuring 26 constructs), cognitive abilities (85 questions covering 11 measures), economic preferences (34 questions covering 10 measures), as well as heuristics and bias experiments (48 questions from 16 experiments), and a pricing study (40 questions), collected during a four-wave, longitudinal study (average completion time of 145 min). These (human) data have high test-retest accuracy and good face validity and largely replicate known effects, suggesting that they provide high-quality training data for digital twins (*18*). While no single study can evaluate all possible instantiations of digital twins, our implementation represents the most replicable and scientifically rigorous approach currently available. Unlike twins built on proprietary data, ours are built using the most comprehensive dataset that is publicly available today. We complement this public resource by contributing an even more comprehensive dataset (including responses to unpublished stimuli and paradigms—all included in the SM), which can be merged with the Twin-2K-500 dataset by matching participants across the two datasets.

**Fig. 1.**
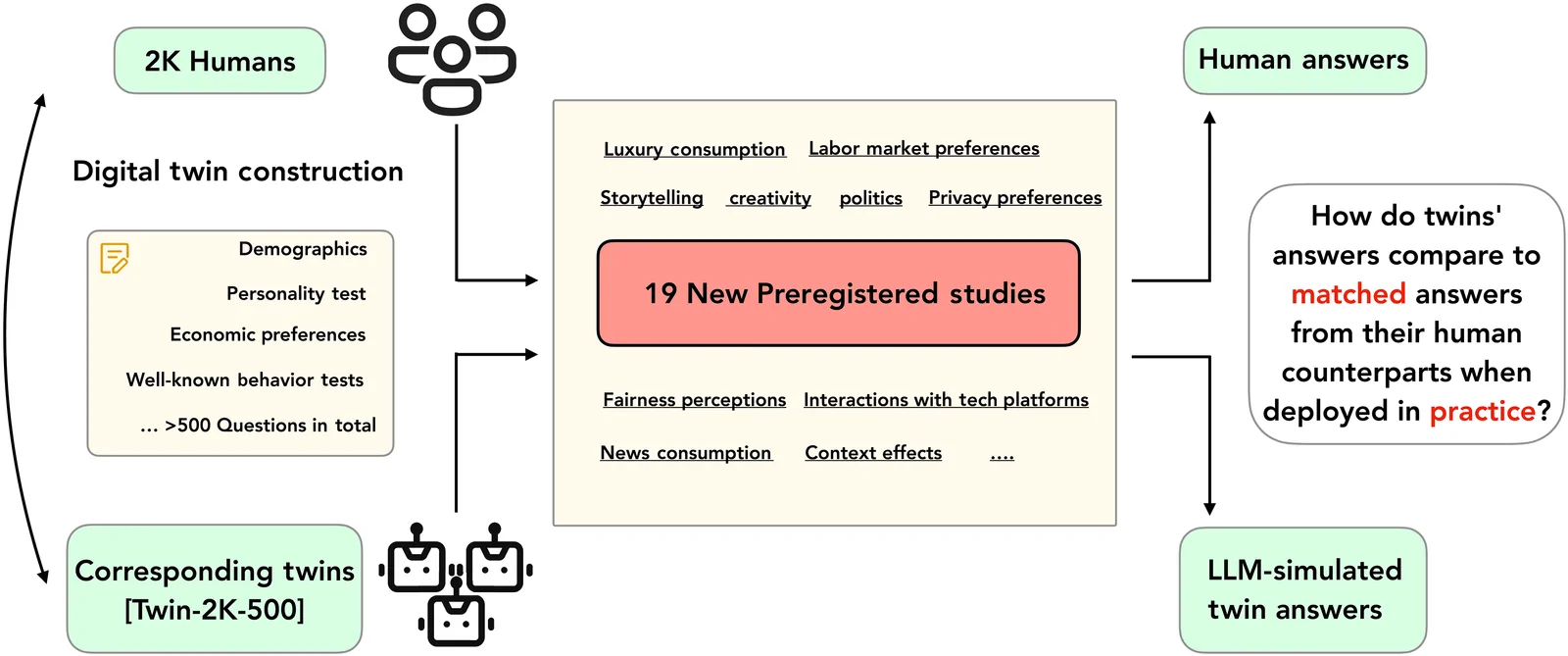
Mega-study overview. We ran 19 preregistered studies on digital twins from the Twin-2K-500 dataset and their human counterparts. The studies were proposed by a diverse group of scholars and cover a wide range of behaviors and domains. As a set, they represent how digital twins may be leveraged today by social scientists. We match the answer of each digital twin to each question with the answer from their human counterpart, allowing us to explore the performance of digital twins at both the individual and population levels.

In each substudy, both human participants and their corresponding digital twins answered the exact same questions. By matching each digital twin’s answers to those given by its human counterpart to the same questions, we are able to test, across a wide range of domains, the individual-level accuracy of digital twins, as well as the correlation between responses from the digital twins and their human counterparts. Further, because our digital twins are based on a representative sample of the US population, we can explore not only how performance varies across types of domains and questions but also which groups are well represented by digital twins and which are not. We share the detailed analyses of each substudy for researchers interested in using digital twins in specific domains (see the SM). We make our data (https://huggingface.co/datasets/LLM-Digital-Twin/Twin-2K-500-Mega-Study) and code (https://github.com/TianyiPeng/Twin-2K-500-Mega-Study) publicly available. See also https://zenodo.org/records/20603429 for a permanent public repository. Researchers may combine our data with the original Twin-2K-500 dataset, with twins matched using a Twin ID. For privacy reasons, we are unable to share the Prolific ID associated with each Twin ID; therefore, other researchers are not able to run new studies on the human sample as we did. See Fig. 1 for an overview and the SM for details of our mega-study.

We preregistered all our studies and their 164 associated outcomes (one preregistration per study, ResearchBox no. 4145), as well as the use of correlation as a basic performance metric (AsPredicted no. 223921). Our exploratory analysis revealed the benefits of including additional metrics to provide a fuller description of the results and the need to include multiple benchmarks and implementation methods. This means that our meta-analysis reported in the “Digital twin performance across domains” section was preregistered (AsPredicted no. 223921), but the benchmarking reported in the “Performance comparisons” section, the comparison of implementation methods in the “Digital twin performance across implementation methods” section, and the five distortions (reported in the “Five key distortions” section) emerged from exploratory analysis and were therefore not preregistered.

## RESULTS

### Digital twin performance

#### Performance metrics

We preregistered 164 outcomes (e.g., willingness to share a particular article on social media and likelihood of applying for a particular job) across our 19 substudies. We focus on performance measures that directly match the response of each digital twin to that of its human counterpart. Specifically, for each outcome, we match each twin’s response to that of its corresponding human and compute two metrics (correlation was preregistered, individual-level accuracy was not):

1) Individual-level accuracy: Following previous research (*18*, *21*), we measure individual-level accuracy as 1 − (MAD/range), where MAD is the mean absolute deviation between a human’s response and that of their twin, and range is the natural range of the outcome. We exclude outcomes without a defined range, such as open-ended price estimates, resulting in 161 outcomes for this metric. We compute the average individual-level accuracy across participants for each outcome, where higher values indicate greater accuracy (between 0 and 1, where 1 denotes perfect accuracy). For example, on a 1 to 7 scale, if the human answered 5 and the twin answered 6, then accuracy would be 0.833.

2) Correlation: We calculate the correlation between human and twin responses across participants, for each outcome. This metric reflects the degree to which twins capture individual-level heterogeneity in human responses. Following previous research (*17*), we compute an overall average correlation across outcomes by first applying a Fisher *z*-transformation to individual correlations, averaging these values, and then converting back using an inverse transformation.

In addition to the metrics above, which are based on matched human-twin data, we also compute two distribution-level performance metrics that compare the aggregate distributions of responses from twins and humans for each outcome (not preregistered). These metrics are more commonly available (no individual-level matching required) but arguably less informative:

1) Comparison of averages: We compare the average response of humans with that of their twins for each outcome using the absolute value of Glass’s Δ. Glass’s Δ is an effect size measure calculated as: $\Delta =\left({\hat{X}}_{\text{twin}}-{\hat{X}}_{\text{human}}\right)/s_{\text{human}}$, where ${\hat{X}}_{\text{twin}}$ and ${\hat{X}}_{\text{human}}$ are the sample means of the twin and human groups, respectively, and $s_{\text{human}}$ is the SD of the human group. It is similar to Cohen’s *d*, but it relies on the SD from the human sample only, as the assumption of equal variances between the two samples is not valid in our setting. Higher values of this metric indicate larger differences between group means (expressed in SDs).

2) Comparison of SDs: We compute the ratio of SDs between humans and twins for each outcome. Values below (above) 1 suggest lower (higher) variance in twins relative to humans.

#### Performance comparisons (not preregistered)

**Fig. 2.**
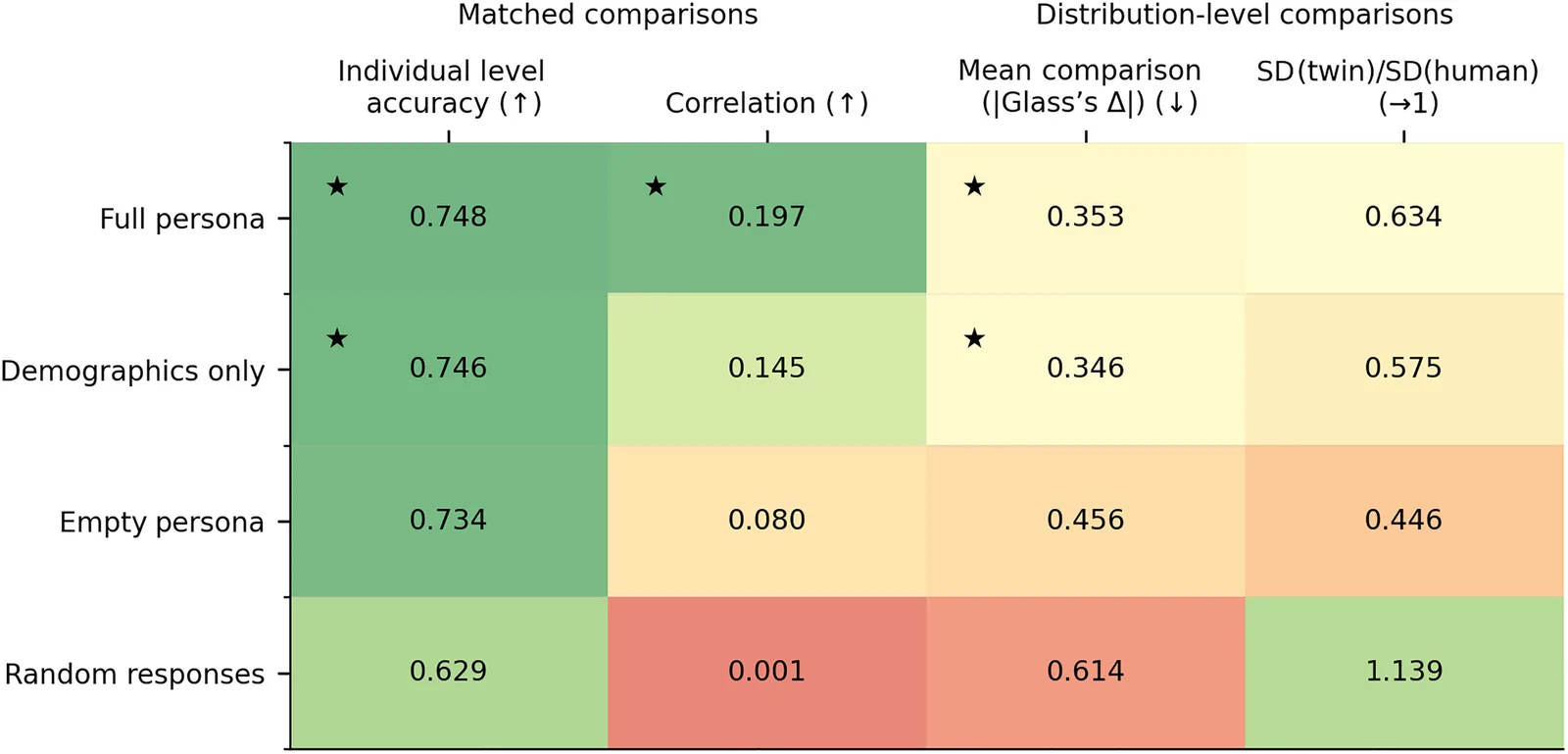
Gains from leveraging individual-level data. *Best performing benchmark, or not significantly different from best at *P* < 0.05 (not applicable to ratio of SDs), after applying Bonferroni correction for multiple comparisons in each column. Creating digital twins using rich individual-level data leads to a modest improvement in accuracy over predictions made without any individual-level data (empty persona) and no significant improvement over personas based on demographics only. Full personas improve the correlation between twin and human responses compared with these benchmarks, although the correlation is modest and digital twins remain underdispersed.

By default (and unless otherwise specified), we build each twin using a “full persona” approach that augments the LLM’s context window with that human’s complete data from the Twin-2K-500 dataset. Across all outcomes, the average individual-level accuracy of the full persona approach is 0.748. See Fig. 2 and the SM for histograms of this and other performance metrics across outcomes for the full persona approach.

To contextualize this level of accuracy and quantify gains from leveraging individual-level data for out-of-distribution predictions, we compare this performance with several benchmarks. First, we compare performance with that of random responses, where responses to each outcome are drawn from a uniform random distribution with support equal to the outcome’s range (i.e., no LLM is required for this benchmark). The random benchmark already achieves an individual accuracy level of 0.629, underscoring that absolute levels of individual-level accuracy should be interpreted relative to this baseline. As an illustration, if the true responses were uniformly distributed on [0,1], always predicting 0 would yield an individual-level accuracy of 0.5.

Next, we compare the full persona digital twin approach with a simple “empty persona” benchmark, which uses an identical prompt for all respondents (i.e., no individual information) and thus only reflects the base model’s behavior (with serving status and characteristics of the base model such as temperature being the only source of variation). The empty persona benchmark achieves an average accuracy of 0.734, which is only modestly lower than full personas (a difference of 0.014, albeit statistically significant). We perform paired *t* tests to compare each pair of benchmarks on each metric, with appropriate corrections for multiple comparisons. We drop one outcome in these comparisons, the creativity rating of ideas generated by humans and their twins in one of the substudies, as it was provided by humans and was only available for ideas from twins based on the full persona approach.

We lastly introduce a “demographics only” benchmark, which uses a persona description that only includes the 14 demographic variables (region, sex, age, education, race, citizenship, marital status, religion, religious attendance, political party, household income, political ideology, household size, and employment status) which are part of the Twin-2K-500 dataset (and therefore also included in the full persona description). This benchmark captures the performance that would be obtained from generic personas based on demographic characteristics only. We find that individual-level accuracy of full-persona twins is not significantly better than personas based only on demographic information (${\text{Accuracy}}_{\text{full}\;\text{persona}}=0.748$, ${\text{Accuracy}}_{\text{demographics}\;\text{only}}=0.746$, *P* = 0.37). This provides initial evidence that, when predicting an individual’s response, digital twins may mostly rely on stereotypical demographic-based tendencies rather than modeling an individual’s distinct cognition. We explore this phenomenon and the comparison between full-persona and demographics-only twins more thoroughly in a subsequent section.

At the aggregate level (comparing the mean twin response with the mean human response for each outcome), twin responses differ from human responses by 0.352 SDs on average. This means the mean squared error (MSE) of the digital twin’s mean estimate is about ${\left(0.352\sigma \right)}^{2}\approx 0.12\sigma^{2}$, which is approximately equal to the MSE of the sample mean from 10 humans, $0.1\sigma^{2}$. A paired *t* test (using Bonferroni correction for multiple comparisons) indicates a statistically significant difference (P<0.05/164) between humans and digital twins in 105 of 164 outcomes (64.0%).

We next turn to our correlation metric, which captures how well twins reflect relative human-level differences in individual responses across participants. For correlation, we apply a *z*-transformation before conducting *t* tests. The correlation between twin and human responses is positive in 157 of the 164 outcomes (95.7%) and significantly positive (P<0.05/165—applying Bonferroni correction) in 97 of these cases (59.1%). The overall average correlation is 0.197. We set correlation to 0 for cases where there is no variation among twin answers, which may occur with the empty persona or demographics only benchmarks. For the three outcomes that do not have a natural range, we use the empirical range in humans’ answers as support for the random distribution. On that metric, full personas perform markedly better than demographics-only personas [correlation coefficient (*r*) = 0.145], empty personas (*r* = 0.080), and random responses (*r* = 0.001). Nevertheless, the correlation achieved by digital twins is modest by most social science standards and comparable with the correlation found between height and intelligence (*22*). Note that Park *et al*. (*17*) report a much higher correlation in their digital twin study. However, they compute the correlation across questions for each participant. In contrast, we compute the correlation across participants for each outcome. This is more often the measure of interest, as social scientists often ask questions such as who is more likely to vote for a candidate or who is more likely to purchase a specific product.

In sum, enriching digital twins with detailed individual-level information significantly improves individual-level prediction, that is, the ability to reproduce the exact responses given by specific participants. However, the improvement over an empty persona is arguably negligible. Second, digital twins significantly improve our ability to capture heterogeneity across participants and predict relative differences between them, that is, it enhances our ability to distinguish one participant from another. However, the correlation between answers from digital twins and their human counterparts is modest. Together, these findings establish an empirical benchmark for where digital twin technology stands today, revealing that although they show promise, digital twins fall well short of their promise when placed in a rigorous and ecologically valid test.

#### Digital twin performance across implementation methods (not preregistered)

All results reported so far use GPT4.1 (dated 14 April 2025) with a default temperature of 0.7. One might wonder whether poor digital twin performance was, in part, due the specific configurations we used rather than fundamental limitations of current digital twin technology. To explore the role of these factors, we systematically varied the temperature and base LLM (GPT-5, Deepseek, Gemini, and a version of GPT4.1 fine-tuned on the Twin-2K-500 dataset). We find the best results overall using GPT4.1 with temperature = 0 (e.g., highest correlation of 0.232 across all implementation options, tied for best on individual-level accuracy at 0.752). We also experimented with replacing the full-persona information with a concise (approximately 13,000 characters), statement-based summary of the questions and responses with distributional information (e.g., Big 5 personality scores with percentile ranks rather than 44 detailed questions and answers on which the scores are based). We find that this simpler version performs very similarly to the full persona, offering a viable lower-cost alternative. See the SM for details.

We also explored using an LLM fine-tuned specifically for human behavioral prediction as the base model for the creation of digital twins (*21*, *23*). These fine-tuned models, trained on large cross-sectional datasets, may indeed serve as alternative base LLMs, i.e., they can be combined with additional panel data in which participants are tracked across multiple experiments or studies, allowing the creation of twins associated with specific individuals. We explored using Centaur (*23*) as a base model. Performance was lower than that achieved using GPT as the base model. The relatively low performance may be due to limitations of the Llama base model on which Centaur is built or to our formatting and out-of-distribution testing, among other reasons. We leave a thorough investigation of the training of digital twins on such enhanced LLMs for future work. See the SM for details. Overall, twin performance across temperatures, base models, and persona formatting was, at best, modest.

#### Digital twin performance across domains (preregistered)

The breadth of our study allows exploring the performance of digital twins across domains, using a meta-analytic approach. We conduct regressions with each performance metric as the dependent variable. Given that correlation is the performance metric most sensitive to the individual-level information captured by digital twins, we focus on the regression with the (*z*-transformed) correlation for each outcome as the main dependent variable. We *z*-transform the correlation due to better statistical properties, e.g., being approximately normally distributed. Our independent variables include a set of categorical labels that characterize each outcome, related to the following domains: social, preferences/attitudes, cognitive skills/rationality, content evaluation, and human-tech interactions. We also include mechanical features that might influence performance (e.g., sample size).

We estimate a mixed linear model, including random intercepts for each substudy (see the SM for details). We find that correlation was, on average, higher in the cognitive domain, on outcomes related to human-technology interactions, and on outcomes that use response scales. Digital twins also performed relatively better overall in the social domain. The correlation was higher when outcomes related to conflict, pro-social issues, social cognition, or personality. However, the correlation was significantly lower for outcomes where social desirability was salient, suggesting twins are less capable of mimicking human responses in socially sensitive contexts (descriptive evidence suggests twins are more likely to provide socially desirable responses). Correlations were also lower in the political domain [consistent with previous findings such as (*13*) or (*14*)], on outcomes with valenced (positive versus negative) evaluations, and when questions varied across participants [consistent with prior findings that LLMs struggle to treat prompt-level variation as exogenous (*24*)].

### Five key distortions (not preregistered)

A natural objection to benchmarking digital twin performance today is that the technology will undoubtedly improve as LLMs continue to evolve, making current performance largely beside the point. However, meaningful improvement requires both an understanding of how digital twins deviate from humans and a clear rubric of indicators against which progress can be measured. To that end, we next characterize systematic distortions in digital twins’ representations of human behavior that may underlie their poor performance. These distortions are consistent with previous literature on synthetic data, suggesting that digital twins in their current form are not able to solve known limitations of synthetic data. Of course, we cannot claim that our list is exhaustive, but addressing these distortions is a necessary, if not sufficient, condition for improving the validity of digital twins.

#### Insufficient individuation

Adopting a Bayesian framework, one may think of the base LLM as reflecting a prior distribution over how an individual may respond to a particular question. The additional information fed to the LLM to build that person’s digital twin leads to an updated, posterior distribution of answers tailored to that individual. Previous work on other forms of synthetic data found that LLM-generated responses often suffer from excessive homogeneity (*16*). Santurkar *et al*. (*13*) argue that LLMs are steerable through customized prompting but that steerability does not guarantee alignment with human responses.

By augmenting the base LLM with extensive individual-level data, digital twins offer the potential to address this issue. To empirically assess whether this is the case, we first compare the amount of variation in answers from twins versus humans. The SD of the twin responses is lower than that of human responses in 154 of 164 cases (93.9%), indicating underdispersion in twin responses. This difference is statistically significant (P<0.05/164—applying Bonferroni correction) in 140 of those 154 cases.

The underdispersion of digital twin answers relative to their human counterparts suggests that while the additional data are able to steer the distribution in an appropriate direction (i.e., the variations across digital twins better mirror the variations across people), the base model (i.e., the prior) still carries substantial weight and influence on the digital twins’ responses. In other words, the answers are overly “shrunk” toward a base model. This suggests that providing extensive individualized data does not necessarily address the homogeneity bias in LLMs.

To further quantify this, we compare the MAD between answers from full-persona twins versus humans to the MAD between answers from full-persona twins versus empty-persona twins (which only reflect the base LLM). Ideally, full-persona twins would behave more similarly to humans than to empty-persona twins. We find the opposite: Full-persona twins are closer to empty-persona twins than they are to humans (${\text{MAD}}_{\text{full}\;\text{vs.}\;\text{empty}}=0.175$, ${\text{MAD}}_{\text{full}\;\text{vs.}\;\text{humans}}=0.252$, P<0.01).

In sum, we find that digital twins distort human behavior by insufficiently deviating from the base model. This yields the first benchmark for progress: comparing SD of twin responses with human responses. If full-persona twins approximate the SD of human responses, this suggests that twins have overcome the homogenization that plagues LLMs.

#### Stereotyping

We have shown that digital twins do not deviate sufficiently from the base LLM. To the extent they deviate, do they do so in a way that over-relies on generic characteristics such as demographics? Previous research has documented that LLMs prompted to mimic specific demographic groups tend to provide answers that are stereotypical of these groups (*16*, *25*). Here again, we explore whether augmenting persona description with rich individual-level data is enough to correct such phenomenon.

The fact that full-persona twins achieve only slightly higher individual-level accuracy than those based only on demographic information provides initial evidence for demographic-based stereotyping. However, to test this more directly, we compute the MAD between the answers (normalized between 0 and 1) given by full-persona twins versus demographics-only twins. The average MAD across outcomes is 0.132, which is significantly lower than the average MAD that we reported above between full-persona twins and empty-persona twins (${\text{MAD}}_{\text{full}\;\text{vs.}\;\text{demographics}}=0.132$, ${\text{MAD}}_{\text{full}\;\text{vs.}\;\text{empty}}=0.175$, P<0.01) or the actual human answers (${\text{MAD}}_{\text{full}\;\text{vs.}\;\text{demographics}}=0.132$, ${\text{MAD}}_{\text{full}\;\text{vs.}\;\text{humans}}=0.252$, P<0.01). That is, answers from full-persona twins are closer to those from demographics-only twins than to those from empty-persona twins or real humans. This suggests that digital twins rely heavily on demographic characteristics rather than the wealth of additional individual-level information that they were provided.

As discussed earlier, correlation on the other hand improves more substantially when using full versus demographics-only personas. This suggests that, to the extent full-persona twins deviate from demographics-only twins, they do so in a way that is somewhat consistent with variations across humans.

**Fig. 3.**
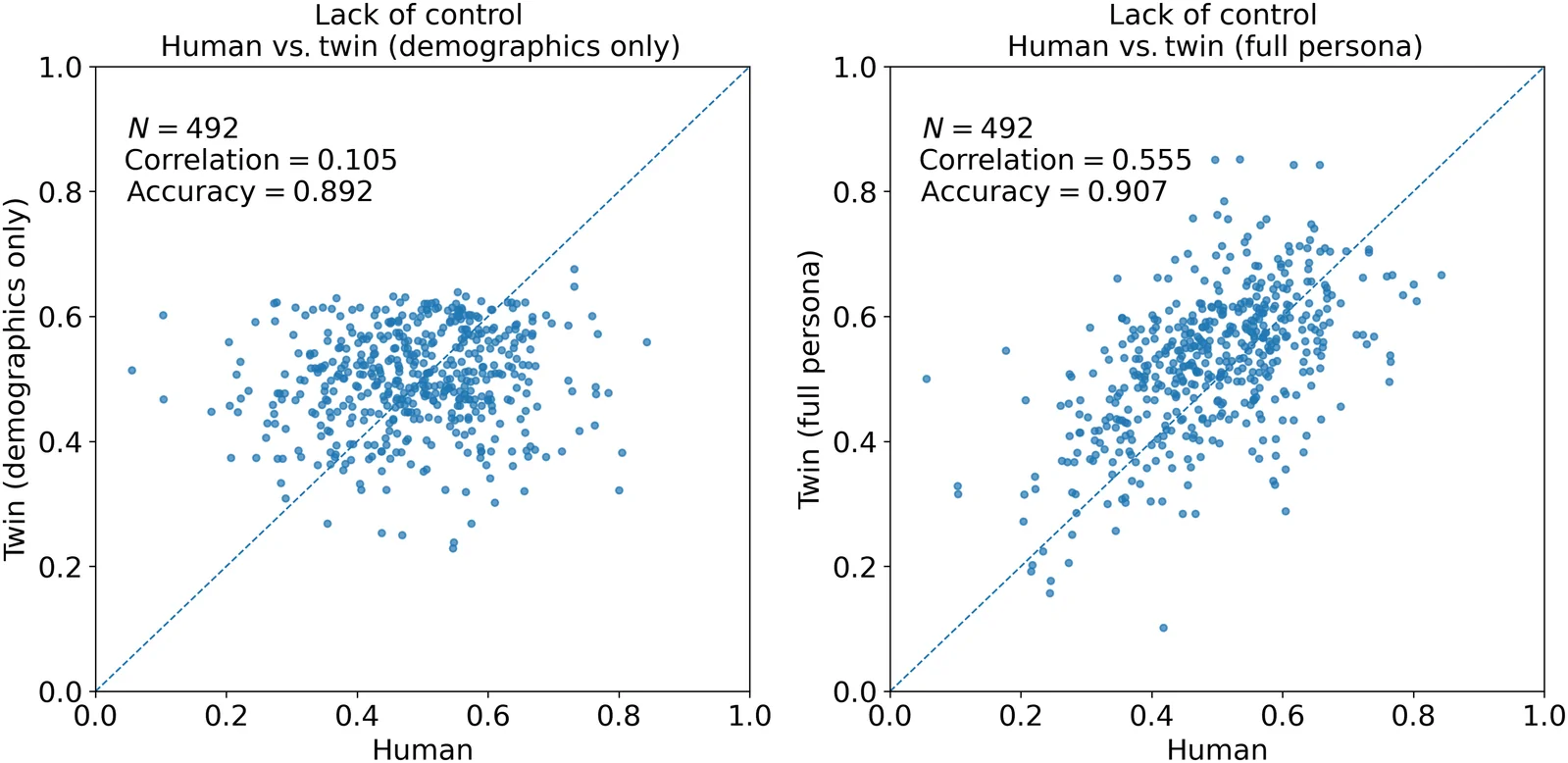
Human responses versus digital twin responses based on demographics only (left) and full persona (right), for one particular outcome. The 45° line is included. This scatter plot illustrates how correlation may be improved when full personas are used, without significant change to individual-level accuracy (captured by the average distance of each point to the 45° line).

It may be counterintuitive that full personas significantly improve correlation compared with demographics-based personas, without improving accuracy. However, accuracy and correlation are distinct constructs. To illustrate the distinction between improving correlation and improving individual-level accuracy, we select one outcome (Lack of Control) from one substudy (substudy 2, Affective Primes), for which the distinction between individual-level accuracy and correlation is particularly sharp. Figure 3 reports scatter plots of the predictions from synthetic personas created using demographics only versus human responses (left panel), and predictions from digital twins created using full personas versus human responses (right panel). These plots clearly illustrate how the correlation is much improved when full personas are used ($r_{\text{demo}}=0.105$ versus $r_{\text{full}}=0.555$). Yet, individual-level accuracy (captured by the average distance of each point to the 45° line) is virtually unchanged (${\text{Accuracy}}_{\text{demo}}=0.892$ versus ${\text{Accuracy}}_{\text{full}}=0.907$).

To illustrate this comparison more concretely, consider two people with similar demographics. One person rates themselves a 2 out of 7, and the other rates themselves a 4 out of 7 on feeling a lack of control. Using demographics only, the twins might report 3 for each, 1 point away from the human’s actual response on average. Using the full persona, the twins might now report 3 and 5, respectively, still 1 point off on average, failing to capture the individual’s subjective experience to a similar extent, but now capturing the relative difference across people. This suggests that personalized twins can sort people more accurately (who feels more or less control) but still fail to capture individuals’ actual cognition and subjective experiences. Note that individual-level accuracy should not be confused with other aggregate measures of accuracy, such as the accuracy of the average difference between groups of participants (e.g., average treatment effect). These measures may be more strongly affected by improvements in correlation.

In sum, one way digital twins distort human behavior is by over-relying on demographic information rather than modeling the complexity of individual cognition. This yields the second benchmark for progress: comparing responses of full-persona twins with those of (i) demographic personas, (ii) empty personas, and (iii) humans. If full-persona twins are closer to demographic personas than humans, that suggests an over-reliance on demographics at the expense of true individuation.

#### Representation bias

We have so far documented two distortions that can explain inaccuracy. However, is this inaccuracy uniform across the humans that digital twins are attempting to represent? As training data are typically based on corpora that systematically overrepresent certain demographics (*26*), previous research has shown that LLMs struggle to reflect the opinions of certain demographic groups (*13*, *27*). Does creating digital twins using demographic data combined with extensive individual-level data address this issue? To explore this, we compute accuracy (across all outcomes) between each participant’s normalized responses and those of their twin. We use accuracy for this analysis as a more natural way to compare the answers between a particular human and their twin across outcomes. We then analyze how this accuracy relates to participants’ demographic characteristics. All demographic variables are dummy coded, yielding 61 demographic dummy variables coming from the 14 demographic questions. To identify which participant characteristics are most predictive of digital twin performance, we train an XGBoost (extreme gradient boosting) model to predict twin accuracy using these features (*28*).

**Fig. 4.**
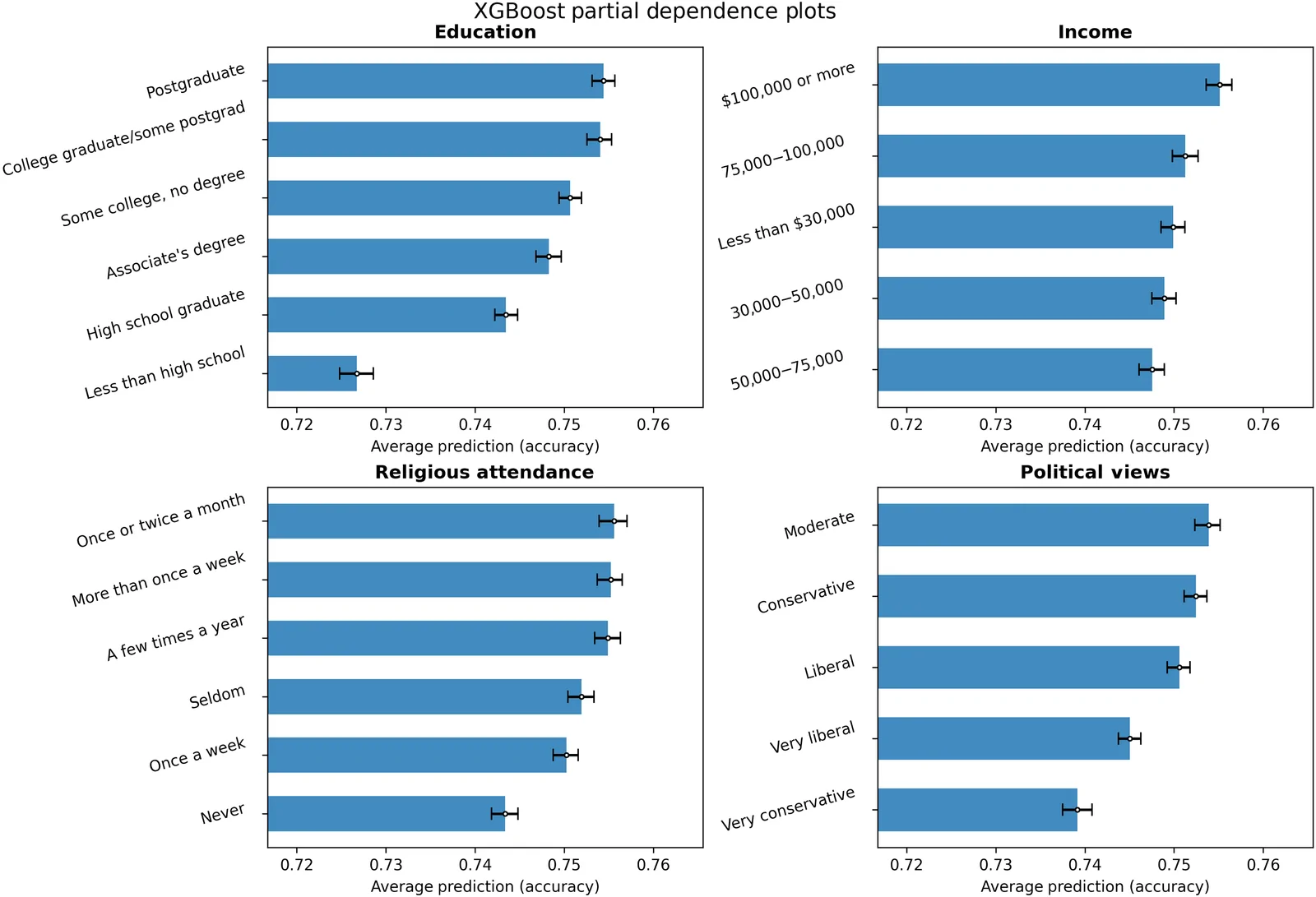
PDPs for understanding heterogeneity in digital twin performance. Twins tend to be more accurate for participants with higher education levels and higher income, as well as those with moderate political views and religious attendance habits.

To interpret the model’s findings, we generate grouped partial dependence plots (PDPs) for each categorical variable. PDPs visualize the average predicted accuracy for each level of a feature while holding all other variables constant. For example, the PDP for political ideology shows predicted accuracy for participants identifying as “Very Conservative,” “Liberal,” “Moderate,” “Liberal,” and “Very Liberal” on a single chart, enabling direct comparison across different demographic groups. Plots for education, income, religious attendance, and political views are presented in Fig. 4, while the remaining plots are in the SM.

Our findings suggest representation bias, i.e., systematic differences in twin accuracy across demographic groups (*29*). Twins tend to be more accurate for participants with higher education levels and higher income, indicating a potential bias in favor of more socioeconomically advantaged individuals, which could exacerbate a focus on WEIRD (Western, Educated, Industrialized, Rich, Democratic) populations in the social sciences (*27*, *30*). Accuracy also tends to be higher for participants with moderate political views and religious attendance habits.

In sum, this differential accuracy constitutes another distortion: Digital twins systematically misrepresent certain groups more than others. This yields the third benchmark for progress: visualizing PDPs across segments (such as demographic or psychographic variables). Flat plots suggest that these different groups of people are represented equally well by their digital twins.

#### Ideological biases

The first three distortions discussed above represent failures to capture individual cognition by over-relying on demographic information, insufficiently adjusting away from the base model, or insufficiently capturing the specific behaviors of certain groups. These can be seen as errors of omission. However, another possible avenue of distortion is the introduction of bias in responses. Our data provide a unique opportunity to contribute to the growing literature on LLM biases by directly comparing opinions expressed by humans with those expressed by an LLM instructed to mimic those humans, across a wide range of substantive areas.

Specifically, in addition to 164 detailed outcomes (e.g., willingness to share a particular article on social media and likelihood of applying for a particular job), we also preregistered 31 higher-level comparisons between digital twins and humans (1 to 4 comparisons per substudy). These comparisons evaluated whether twins responded similarly to humans on average to a particular question or set of questions (e.g., “How does the knowledge of digital twins regarding fees and surcharges in the marketplace compare to that of their human counterparts?”), whether they replicated known treatment effects (e.g., “Can digital twins demonstrate default effects?”), etc. A complete list of these comparisons and their results is available in the SM; we only highlight a subset here, which reveal systematic ways in which the content of digital twin answers differ from that of humans. In particular, our diverse mega-study allowed us to uncover subtle ideological biases embedded in the twins’ responses.

Specifically, we found that twins tended to express views that were often more “pro-human” than those expressed by humans. For example, compared with their human counterparts, twins were more likely to believe that people are fair and can be trusted (substudy 15), that people should take care of themselves (substudy 15), and twins were relatively more favorable to people who donate to both political parties (substudy 16). They were also more favorable toward government regulation of fees and surcharges in the marketplace (substudy 7) and more willing to pay taxes to improve healthcare for all people (substudy 15).

At the same time, twins also tended to express views that could be described as “pro-technology.” In particular, twins tended to provide answers that were consistent with a view of technology as a safe tool under the control of humans (e.g., substudies 6, 9, 14, and 19). For example, twins were relatively more accepting of algorithmic hiring compared with their human counterparts (substudy 9), perceived online targeting as less intrusive on their privacy (substudy 14), and underreported usage of platforms like Netflix and TikTok (substudy 19). While it is possible to be simultaneously pro-human and pro-technology, do digital twins show any preference in favor of humans versus technology? When asked to rate the creativity of ideas (substudy 10), twins showed human aversion (rating ideas from humans lower because of their source).

These biases may come from various inputs into the base model. In particular, they may be reflected in the training corpus of the base model, or they may have been acquired through reinforcement learning with human feedback (*13*) or through the base model’s system prompt that provides overarching instructions on how the model should behave. Irrespective of their exact sources, they imply that blindly deploying digital twins might lead to distorted conclusions about human behavior and opinions, which may, sometimes in very subtle ways, push specific ideological agendas.

In sum, we find that digital twins distort human behavior by exerting ideological sway. This yields the fourth benchmark for progress: checking for systematic ideological biases (including pro-technology, pro-social, and pro-human biases) by testing whether human-twin divergences are consistently in a specific direction across substantive domains.

#### Hyper-rationality

Another form of bias that may be introduced by digital twins relates to cognitive skills. Specifically, one of the features of LLMs—their vast knowledge base—may be a source of error when it comes to predicting human behavior. Indeed, prior work finds that LLMs suffer from hyperaccuracy (*18*, *31*). Does training digital twins on extensive data, including response from numerous cognitive tests, help replicate human’s imperfect knowledge and cognitive skills? Or, akin to the curse of knowledge in humans (*32*), are digital twins unable to “forget” their base knowledge leading them to behave as more informed, rational agents? We first examined this question in substudies that contained questions with objectively correct answers (Substudies 7,19). We find that twins tended to display perfect knowledge, deviating from human participants.

We next examined this distortion for substudies that contained questions that test rationality. We again find evidence of hyper-rationality distortion: Twins were more likely to select normative answers reflective of higher cognitive abilities, e.g., by selecting answers associated with stronger quantitative intuition (substudy 17) or by being more likely to strategically consume online content to influence future recommendations (substudy 19). Twins also failed to replicate the attraction and compromise effects (substudy 4), which are indicators of bounded rationality. However, an interesting phenomenon emerges when using a well-known, published paradigm. Here, twins were *more* likely to show the effect, probably because of the phenomenon of leakage, whereby the answer is directly influenced by information contained in the training corpus (*19*). For example, digital twins did not replicate a default effect using an unpublished paradigm (substudy 5), but they did display a strong default effect using a classic paradigm (*33*). Other substudies (e.g., substudy 1) confirmed the tendency of digital twins to behave consistently with the literature when a clear prediction could be made. This highlights a key contribution of our megastudy: We systematically test twins across both established and novel paradigms.

In sum, digital twins distort human behavior by imposing a hyper-rational lens. This yields the final benchmark for progress: testing twin performance in novel contexts that contain objectively correct answers and test rationality. If twins deviate less from correct responses than humans, and if twins display irrationality at lower rates to humans, this suggests that they are still subject to the hyper-rationality bias in LLMs.

## DISCUSSION

Although many are rushing to simulate surveys and experiments using digital twins of humans, little is known about how responses from digital twins compare with those of their human counterparts. Further, there is little understanding of the best way to construct digital twins, of the domains of application in which digital twins are likely to be accurate, or on the profiles of humans whose digital twins are likely to be more accurate. Deploying digital twins without answering these fundamental questions risks misrepresenting human cognition in ways that could undermine both scientific understanding and practical applications. To help advance knowledge on digital twins, we ran a preregistered mega-study (and make the data publicly available) that (i) allows matching the answers of digital twins to those of their human counterparts, (ii) covers a wide range of application domains, and (iii) involves a relatively large, representative sample of participants.

We find that although digital twins show promise, they are not yet “ready for primetime.” Digital twins capture individual cognition only minimally better than empty personas. However, twins show slightly more promise when it comes to sorting people. This distinction is consequential for understanding when indiscriminate twin use may mislead decision-makers. For example, although twins may be able to modestly estimate relative receptiveness to a policy (identifying who is more or less likely to respond positively), they are barely better at predicting an individual’s likelihood to agree with a given policy than a base LLM.

Could human experts have predicted these results? To assess whether empirical tests are valuable and potentially corrective in the domain of digital twins, in a preregistered study (AsPredicted no. 246459) we asked a sample of 68 expert respondents (primarily scholars and managers) to estimate average outcomes in five of our experimental studies representing different paradigms. We asked separately for the average outcome for humans and their twins in a treatment condition (e.g., likelihood of staying enrolled in a green energy plan when enrolled by default), providing the average outcome in the control condition as baseline (e.g., likelihood of actively choosing the green energy plan when enrolled in another plan by default). Overall, the pattern suggested that respondents’ predictions were less accurate for twins than for humans (preregistered; see the SM). Comparing the predicted outcome in the treatment condition with its value in the control condition also yields estimated treatment effects. While the observed average treatment effect among humans was within the 95% confidence interval of predictions made by our participants in all five cases, the average treatment effect among twins was outside the 95% confidence interval in three cases (this particular analysis was not preregistered). This suggests that human experts struggle to predict the behavior of digital twins, highlighting the importance of our rigorous examination.

Beyond performance metrics, our analysis highlights five systematic ways in which digital twins distort human responses. First, we find that digital twins suffer from insufficient individuation, providing answers that remain underdifferentiated and underdispersed relative to human answers. Second, we find evidence of demographics-based stereotyping. Third, digital twin predictions suffer from representation bias, as they tend to be more accurate for certain demographic groups of participants with higher education levels, higher income, and moderate political views and religious attendance habits. Fourth, digital twins tend to display ideological biases, for example, they appear to be at the same time “pro-human” and “pro-technology.” Fifth, digital twins tend to display hyper-rationality, with answers reflecting higher rationality and knowledge compared with humans.

These results culminate in the conclusion that the digital twins of today may best be described as funhouse mirrors that systematically distort human behavior. Stakeholders deploying digital twins risk making consequential decisions on the basis of an illusion of personalization rather than genuine individual-level insight. These systems are better characterized as “comparative profiles” imbued with LLM biases, not faithful twins of human cognition. Consequently, the premature deployment of these twins risks smoothing out individual voices, mispredicting ignorance and irrationality, reinforcing stereotypes, and introducing previously unidentified biases, such as pro-technology attitudes and AI-favoritism.

### Future directions

The five types of distortions we document are consistent with previous research on synthetic data, suggesting that despite their promise, digital twins are currently unable to address limitations of synthetic data. By empirically consolidating findings scattered across the literature into a list of distortions in a single mega-study, we offer a unified evaluative framework: a roadmap with clear guidance on specific ways in which the behavior of digital twins should be corrected before this technology may be deployed safely, with a clear set of indicators on which future methodologies can be measured.

How might future research address these current distortions? Recall that leveraging digital twins involves making out-of-distribution predictions using a base LLM augmented with individual-level data (the twin’s “blueprint”). We compare digital twins to a traditional machine learning approach to explore the extent to which the modest performance of digital twins (and therefore, future potential improvements) lie in the blueprint data being insufficiently informative versus the LLM being unable to fully leverage these data for out-of-distribution predictions (this exploratory analysis was not preregistered). Unlike digital twins, which are able to make out-of-distribution predictions on completely new questions, traditional machine learning models require collecting responses from a subset of the sample, training a model that connects a set of personal characteristics to these responses, and then predicting out-of-sample responses for the rest of the sample (given their personal characteristics).

We consider a case in which an XGBoost model is trained separately for each outcome based on the value of that outcome for a subsample of participants and their full-persona information (i.e., the same information used for full-persona digital twins). We focus on the 106 outcomes for which sample size was greater than 650 and vary the size of the training sample from 50 to 650. We also set temperature to 0 for digital twins, as this improves their performance (see the SM for details). We also consider an XGBoost model trained on demographic variables only. Comparing the performance of a given model with full-persona information versus demographics-only information sheds light on the intrinsic value of the twins’ blueprint data. Comparing the performance of the XGBoost model with that of digital twins holding the persona information constant sheds light on the base model’s ability to leverage individual-level data for out-of-distribution predictions.

**Fig. 5.**
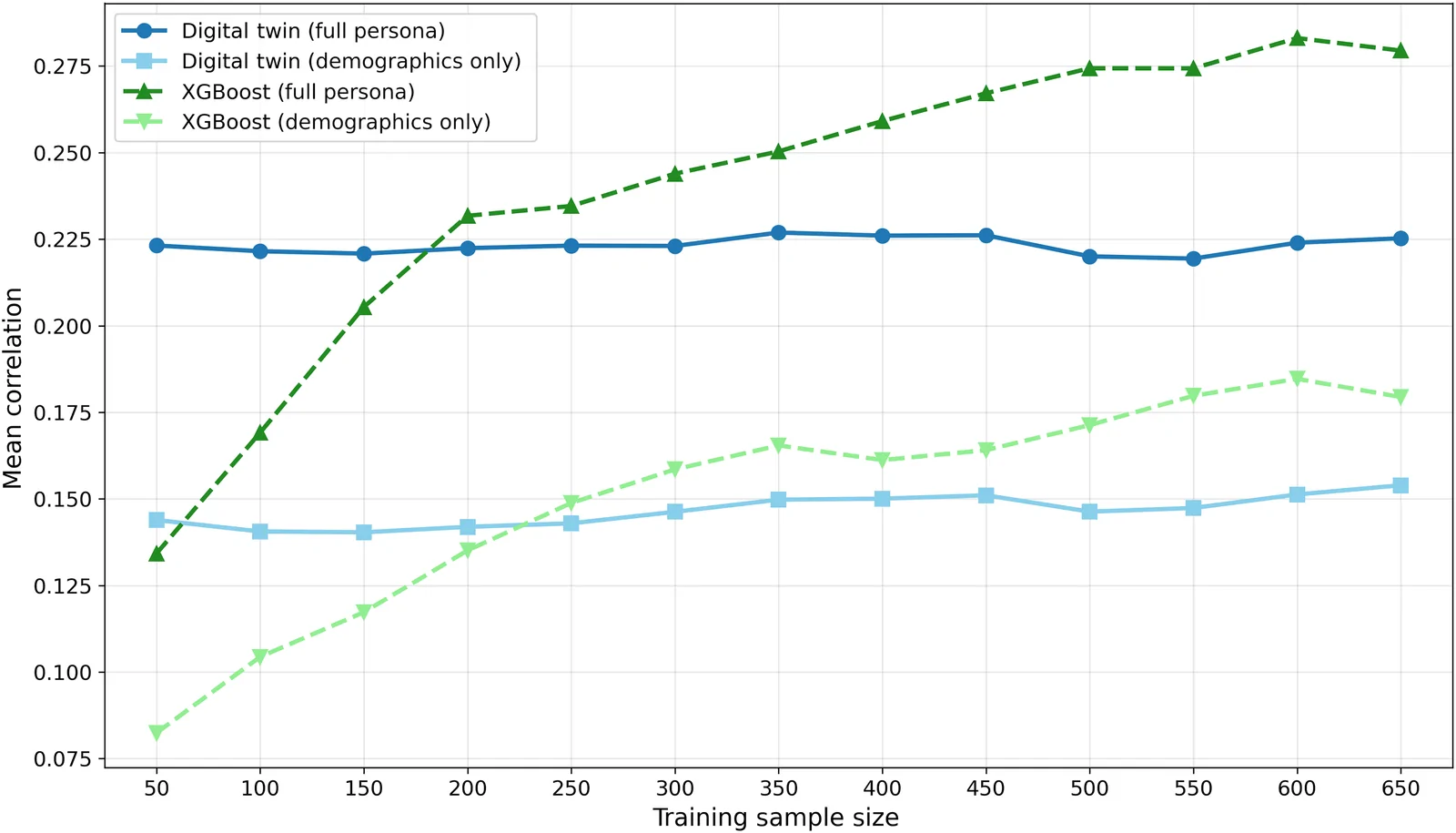
Comparison with traditional machine learning method: Correlation. We compare digital twins (plain lines) with an XGBoost model (dashed lines) trained for each outcome using additional data not needed or used by digital twins: responses for that outcome from a training subset of participants. We consider versions of each model with full-persona information (darker colors) versus demographics only (lighter colors). The full-persona information helps both digital twins and the XGBoost improve performance. However, even as the size of the training sample reaches 650, the predictive correlation achieved by the full-persona XGBoost model remains below 0.29. Holding the set of individual-level characteristics constant, the base LLM is able to make out-of-distribution predictions that achieve the same correlation with human answers as out-of-sample predictions made by an XGBoost model trained on approximately 200 human answers.

Looking first at correlation (see Fig. 5), comparing each model with full personas versus demographics-only personas reveals that the full-persona blueprint data helps both digital twins and the XGBoost improve performance. However, even as the size of the training sample reaches 650, the predictive correlation achieved by the full-persona XGBoost model remains below 0.29. This suggests that, despite being extensive and based on decades of social science research, the twins’ blueprint data has limited intrinsic potential to capture human behavior in our context. At the same time, we see that full-persona digital twins reach a predictive correlation equivalent to that achieved by the full-persona XGBoost model trained on approximately 180 participants. In the demographics-only case, the number increases to approximately 225 participants. In other words, given a set of individual-level characteristics, the base LLM is able to make out-of-distribution predictions that achieve the same correlation with human answers as out-of-sample predictions made by an XGBoost model trained on approximately 200 human answers (i.e., that require running the actual study on approximately 200 participants).

**Fig. 6.**
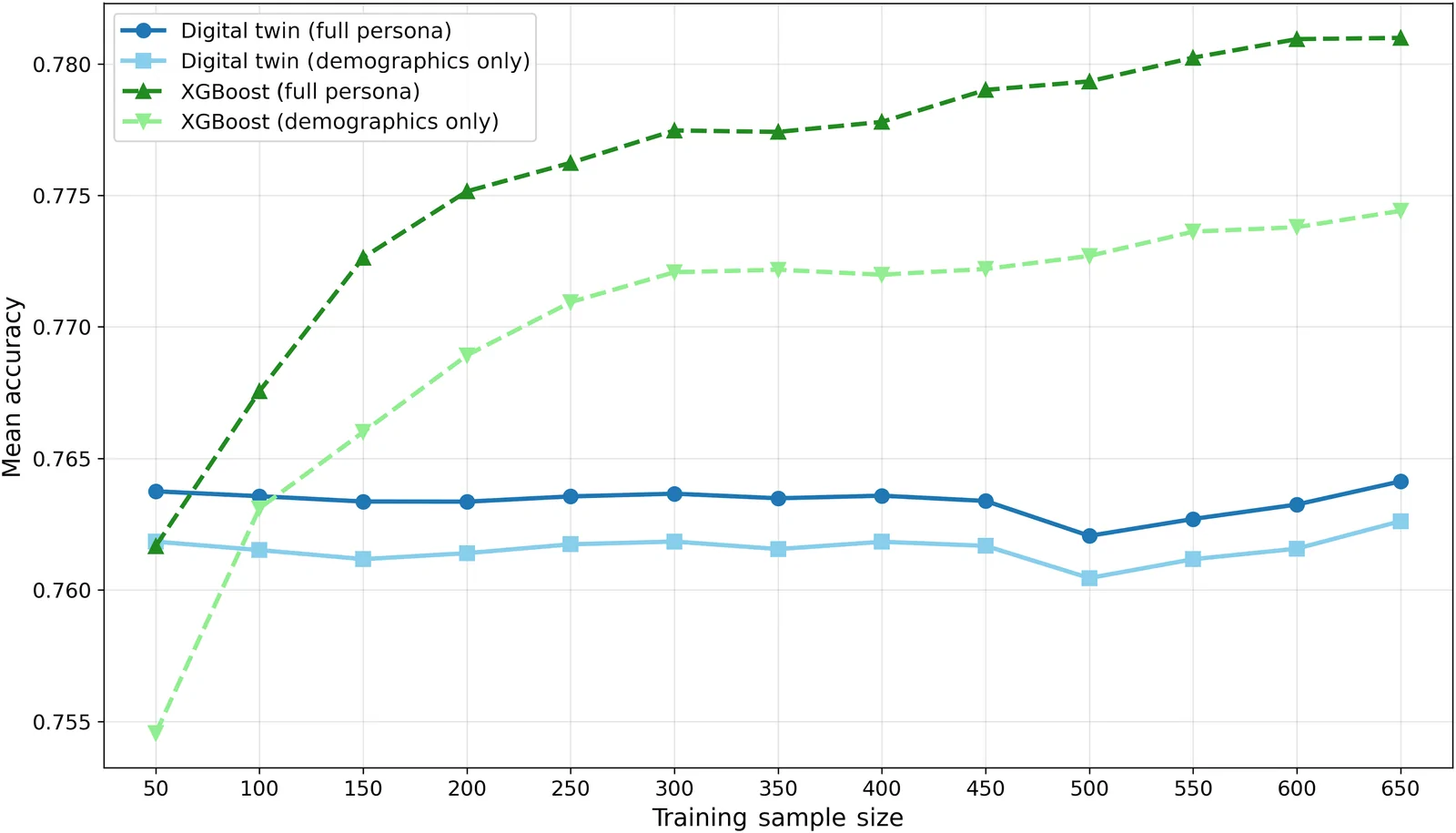
Comparison with traditional machine learning method: Accuracy. We compare digital twins (plain lines) with an XGBoost model (dashed lines) trained for each outcome using additional data not needed or used by digital twins: responses for that outcome from a training subset of participants. We consider versions of each model with full-persona information (darker colors) versus demographics only (lighter colors). The full-persona information only marginally helps improve individual-level accuracy. Moreover, digital twins are only as good as XGBoost trained on approximately 75 human responses on average, suggesting that it is harder for the LLM to leverage these data to make out-of-distribution predictions that would be accurate at the individual level.

When it comes to accuracy (Fig. 6), the XGBoost model trained on the full data is only marginally better than the XGBoost model trained on demographics only (and similarly for digital twins), suggesting that the twins’ blueprint data are only marginally informative of the idiosyncratic patterns in responses. Moreover, digital twins are only as good as XGBoost trained on approximately 75 human responses on average, suggesting that it is harder for the LLM to leverage these data to make out-of-distribution predictions that would be accurate at the individual level.

In sum, this exploratory analysis suggests that the twins’ blueprint data, despite being based on a large number of scales and measures developed over decades of social science research and despite being the most comprehensive dataset for the creation of digital twins publicly available to date, are far from sufficient to fully align digital twins’ answers to those of their human counterparts. Predicting human behavior in our context is probably intrinsically challenging, and performance expectations need to be appropriately calibrated, and/or new scales and measures may need to be developed to better capture human cognition. This analysis further suggests that the blueprint data are relatively more informative of variations across people and that LLMs are able to leverage these data to make out-of-distribution predictions that capture some variation across people (e.g., whether one person will score higher or lower than another), while not being necessarily more accurate at the individual level (e.g., whether one person would answer a 6 versus a 7 on a scale).

Similarly, what role does the base LLM versus individual-level information play in shaping the behavior of digital twins and the distortions that we observe? One may speculate, and test in future research, that several of the distortions that we observe are due to properties of the base LLM being transferred to digital twins. For example, ideological biases and hyper-rationality likely stem from the base LLM powering the digital twins. It is similarly quite plausible that representation bias is linked to certain groups (e.g., higher education and moderate views) being better represented in the base model’s training data. In addition, the insufficient individuation of answers by digital twins is probably the result of the homogeneity of the base model not being sufficiently offset by the individual-level information contained in digital twins.

Of course, a base model is required to enable digital twins to process and generate unstructured data and to make out-of-distribution predictions without additional training data. However, future research might find optimal ways to combine the capabilities of one (or potentially an ensemble of) base LLM(s) with rich individual-level information. We hope our data will encourage the development of improved pipelines for digital twins that address some of the limitations uncovered in our analysis.

Last, our findings also raise the question of how we should reasonably think about digital twins. In particular, on the basis of our results, it may not be realistic to think about them as “clones” of humans, but rather as hyper-rational, quasi-omniscient versions of humans, with implicit values partly imbued by their base LLM. This perspective may influence which use cases are more promising for future research and practical applications. Perhaps use cases that consider digital twins as well-informed advisors are more promising than use cases that consider digital twins as carbon copies of their human counterparts.

In sum, digital twins hold genuine promise in revolutionizing how we study human behavior. However, realizing this promise requires rigorous empirical foundations. We hope that our evaluative framework, publicly available data and code, and detailed analysis of each study (see the SM) provide the data-driven scaffolding needed to reach digital twins’ full potential.

## MATERIALS AND METHODS

Each substudy was first run on Prolific according to its preregistered plan (https://researchbox.org/4145). All substudies were run from the same Prolific account to ensure consistency. Invitations were sent to Prolific participants from the Twin-2K-500 panel. Each substudy was approved by Columbia University’s Institutional Review Board (IRB). Informed consent was obtained for each participant in each substudy. All substudies were run from April to June 2025.

Next, each substudy was conducted on the digital twins of its participants as preregistered (AsPredicted no. 223920). To set up the simulation, we extracted twin information from the Twin-2K-500 dataset and provided it as prompt input. For each human participant, the completed survey was transformed into text, stripped of its answers, and incorporated into the user prompt. Survey questions varied across participants because of randomization; we preserved these differences when generating inputs for their twins. The LLM was then tasked with producing responses to these surveys. The creation of digital twins and the related computations were approved by Columbia University’s IRB under Protocol IRB-AAAV5832.

For each persona in each substudy, we constructed a user prompt using a template (see the SM) and queried the LLM via its application programming interface or API (OpenAI API for OpenAI models, OpenRouter API for others). The JSON outputs were then postprocessed to ensure all questions were answered in the required format (e.g., no missing values or out-of-range responses). If validation failed, the API call was retried. A complete version of the prompt template, including placeholders for persona profiles, new questions, and JSON-based response formatting, is provided in the SM.

## Supplementary Materials

### This PDF file includes:

Supplementary Text S1 to S10 Figs. S1 to S38 Tables S1 to S30 References
