# Supplementary material for: Digital twins are funhouse mirrors: Five systematic distortions
Source: Sci Adv. 2026 Sep 2;12(36):eaeh8260. doi: 10.1126/sciadv.aeh8260 (PMC13537255; doi:10.1126/sciadv.aeh8260)
Supplement: Supplementary file 1 [file sciadv.aeh8260_sm.pdf]

Science Advances
AAAS

Supplementary Materials for

Digital twins are funhouse mirrors: Five systematic distortions

Tianyi Peng *et al.*

Corresponding author: Olivier Toubia, ot2107@columbia.edu

Sci. Adv. **12**, eaeh8260 (2026)
DOI: 10.1126/sciadv.aeh8260

This PDF file includes:

Supplementary Text S1 to S10
Figs. S1 to S38
Tables S1 to S30
References

Supplementary Text S1 Summary of Pre-Registered Comparisons Between Twins and Humans

(see SI section specific to each sub-study for details)

Sub-study (Common Labels)	Pre-registered comparison(s)	Result(s)
1. Accuracy Nudges for Misinformation (social domain, cognitive domain, preference measure, behavioral intention, social desirability, scale question, evaluating content)	Is the effect of prompting people to think about accuracy on sharing of untrustworthy (vs. trustworthy) news similar for twins compared to their human counterparts?	When primed to consider accuracy or reminded of its importance, humans showed no statistically reliable reduction in their willingness to share false versus true entertainment headlines. In contrast, twins responded as the prior work would predict, demonstrating robust improvements in truth discernment.

Sub-study (Common Labels)	Pre-registered comparison(s)	Result(s)
2. Affective Primes (social domain, scale question)	Research in the social sciences often manipulates emotional states using affective priming, where participants are asked to reflect on a recent event. The broad question here is whether affective priming works with digital twins. Specifically:<br>(i) Do affective priming manipulations induce "states" in digital twins (i.e., does writing about gratitude or lack of control momentarily influence digital twins' responses?)<br>(ii) Is the influence of affective priming similar for digital twins and their human counterparts?<br>(iii) Is the influence of affective priming on digital twins dependent on the valence of affective prime (i.e., between positive primes like gratitude or negative primes like lack of control) or the proximal nature of the measures (i.e., does it "spill over" to other, related dimensions or only influence proximal dimensions)?<br>(iv) Is there a relationship between what digital twins and their human counterparts say in their response to the manipulation?	(i) Yes. Writing about feeling grateful or out of control significantly increased corresponding feelings of gratitude and lack of control in digital twins, with these induced "affective states" subsequently influencing downstream outcomes.<br>(ii) No. Affective priming had stronger effects on digital twins' affective responses compared to humans for both gratitude and lack of control manipulations.<br>(iii) Yes. Valence affected twins' responses, but in a similar way to humans: The gratitude prime had a stronger effect on the manipulation check than the lack of control prime for both humans and their twins.<br>(iv) Yes. The semantic content written by each human was significantly more similar to their twin's content than a randomly selected twin's content.

Sub-study (Common Labels)	Pre-registered comparison(s)	Result(s)
3. Consumer Minimalism (social domain, cognitive domain, preference measure, personality measure, scale question)	How does the predictive validity of the Consumer Minimalism Scale vary in a sample of human vs. their digital twins? The prediction is that respondents scoring high on the minimalism scale will prefer minimalist home environments more.	The predictive validity of the Consumer Minimalism Scale documented in human samples replicated robustly in the twin sample. The scale predicted consumers' preferences for minimalist versus non-minimalist apartment interiors.
4. Context Effects (cognitive domain, replicates known human bias, preference measure, test of rationality, different versions of question, behavioral intention)	Can digital twins demonstrate the attraction effect [34] and the compromise effect [35]?	Humans replicated the attraction effect, but twins did not. Neither humans nor digital twins exhibited a compromise effect.
5. Defaults Effects (cognitive domain, replicates known human bias, preference measure, test of rationality, prosocial, different versions of question, behavioral intention, social desirability)	Can digital twins demonstrate default effects [33, 36]?	Human participants showed a default effect in a green energy adoption paradigm but not in an organ donation paradigm. Twins predicted a strong default effect in the organ donation paradigm but no effect in the green energy paradigm.
6. Digital Certifications for Luxury Consumption (social domain, different versions of question, attitude, scale question, human-tech interactions)	How does the propensity to hold higher status perceptions for a luxury product associated with, vs. not associated with, a digital certification, differ between twins and their human counterparts?	Digital certification via a digital passport increased the value perception of a diffused luxury product for humans. Digital twins' value perceptions, however, were not impacted by the inclusion of a digital passport.

Sub-study (Common Labels)	Pre-registered comparison(s)	Result(s)
7. Fees Accuracy (social domain, preference measure, different versions of question, attitude, scale question)	(i) How does the knowledge of digital twins regarding fees and surcharges in the marketplace compare to that of their human counterparts?<br>(ii) How do perceptions of fairness regarding fees and surcharges in the marketplace compare between digital twins and their human counterparts?<br>(iii) How does the variation in perceptions of fairness regarding fees and surcharges in the marketplace compare between digital twins and their human counterparts?<br>(iv) How do beliefs about government regulation regarding fees and surcharges in the marketplace compare between digital twins and their human counterparts?<br>(v) How does the variation in beliefs about government regulation regarding fees and surcharges in the marketplace compare between digital twins and their human counterparts?	(i) Twins were much more accurate than humans on knowledge questions.<br>(ii) Twins closely mirrored the average human response on fairness questions.<br>(iii) Twins exhibited narrower variance than humans on fairness perceptions.<br>(iv) Twins were on average more favorable to government regulation compared to humans.<br>(v) For both groups, conservatism was correlated with less support for pricing regulation. However, twins stereotyped participants based on their political ideology, with exaggerated support for regulation for liberal participants.
8. Heterogeneous Story Beliefs (content evaluation, different versions of question, scale question)	When reading a book and predicting the emotion (valence and arousal) of the next chapter based on the previous chapter, what is the correlation between the expected valence and expected arousal from humans vs. their digital twins?	We found significant positive correlations for both valence and arousal.

Sub-study (Common Labels)	Pre-registered comparison(s)	Result(s)
9. Hiring algorithms (social domain, preference measure, human-tech interactions, different versions of question, scale question)	Can digital twins predict job candidate preferences for workplace hiring policies? Specifically:<br>(i) Can digital twins predict workplace preferences?<br>(ii) Can experimenting on a population of digital twins recover the true average treatment effects as experimenting on a human population?	(i) Twins tended to express views that were overall more pro-social (sustainability, ESG, work-life balance, transparent leadership, culture and values), and less "career-oriented" (firm prestige, career development) than those expressed by humans.<br>(ii) Twins were overall less averse to hiring algorithms compared to humans.
10. Idea Evaluation (cognitive domain, preference measure, evaluating content, pro-social, different versions of question, valenced, scale question)	(i) How do digital twins' ratings of idea creativity compare to those of their human counterparts?<br>(ii) Do digital twins show human favoritism, AI favoritism, or neither when evaluating ideas?	(i) Creativity ratings coming from twins were not significantly correlated with the ratings coming from their human counterparts.<br>(ii) When no information was provided about the source of the ideas, humans rated twin ideas higher (more creative) than human ideas, and twins rated human ideas higher than twin ideas. When information was provided about the source of ideas, humans displayed AI aversion, and twins displayed human aversion.

Sub-study (Common Labels)	Pre-registered comparison(s)	Result(s)
11. Infotainment News Sharing (social domain, cognitive domain, preference measure, content evaluation, behavioral intention, social desirability, scale question)	When choosing an article to share on social media, will digital twins prioritize the following attributes similarly to their human counterpart:<br>1. Headline (more vs. less entertaining)<br>2. Source (more vs. less trustworthy)<br>3. Content type (entertaining vs. informative)<br>4. Political lean (conservative vs. liberal)<br>5. Number of likes (20 vs. 200 vs. 2,000)	While humans and twins both prioritized headline, this consideration mattered much less to twins, who equally relied on number of likes and put far more weight on political lean than their human counterparts. Twins were also sensitive to source trustworthiness, while humans did not differentiate between more or less trustworthy sources.
12. Measures of Creativity (cognitive domain, pro-social)	(i) How do digital twins' creative abilities compare to those of their human counterparts?<br>(ii) Can digital twins replicate the correlations between creativity tests and idea generation performance observed in humans?	(i) The ideas generated by twins were judged as more creative (and with less dispersion in creativity ratings) than the ideas generated by their human counterparts. Digital twins also performed better (and with less dispersion) at the DAT, a creativity task.<br>(ii) The correlation previously found between DAT and creativity was replicated, both among humans and twins.

Sub-study (Common Labels)	Pre-registered comparison(s)	Result(s)
13. Obedient Twins (social domain, preference measure, valenced, scale question)	LLMs are trained to be obedient and deferential. Does this tendency make digital twins more sensitive to survey instructions than their human counterparts? We tested this in three tasks: (i) Self-persuasion [37]. When prompted to consider the other side, do digital twins abandon their attitudes more readily than their human counterparts? (ii) Scenarios. Do digital twins more "obediently" follow the instructions to imagine themselves in different scenarios, leading to more sensitivity to scenario manipulations? (iii) Absurd Scenarios. Do digital twins "earnestly" respond to instructions that would be non-sensical to their human counterparts?	(i) No. Twins were less sensitive to considering a different point of view compared to humans. (ii) No. Twins were less sensitive overall to these scenario manipulations compared to humans. (iii) No. Humans were more sensitive to the instructions to forecast feelings in absurd scenarios.
14. Privacy Preferences of Digital Twins (social domain, preference measure, human-tech interactions, conflict-related, different versions of question, scale question)	How do humans and their digital twins vary in their perceptions of privacy violations for different data processing and targeting practices in online advertising?	Twins perceived their privacy to be violated less than their human counterparts. We found almost no correlation in preferences for individual-level privacy violation scores for twins compared to humans. However, in aggregate, humans and their twins ranked privacy violations similarly across advertising practices that rely differently on tracking and targeting (high rank correlation).

Sub-study (Common Labels)	Pre-registered comparison(s)	Result(s)
15. Preferences for Redistribution (social domain, attitude, scale question)	Can digital twins predict preferences for redistribution and other outcomes like trust, socio-economic background, and beliefs on fairness and social mobility?	Twins showed more extreme redistribution preferences. On average, they were more likely to think "people should take care of themselves" but more willing to pay taxes to improve healthcare for all people. They were more likely to think people are fair and that people can be trusted. They were also more likely to think that hard work and luck are equally important for success. Finally, they showed very little distribution in their fathers' educational attainment, with almost 100% of them reporting their father only completed high school.
16. Promiscuous Donors (social domain, preference measure, political domain, conflict-related, social cognition, attitude, social desirability, different versions of question, valenced, scale question)	How do humans and twins differ in their reaction to (i) others who do not donate to either party, (ii) others who donate to both parties, (iii) justification from those who donate to both parties based on pragmatic vs. value-based reasons?	Human respondents penalized promiscuous donors, especially when the behavior appeared motivated by self-interest (e.g., cultivating access or recruiting talent). In contrast, twins were less punitive (sometimes even favorable) toward the very same targets.

Sub-study (Common Labels)	Pre-registered comparison(s)	Result(s)
17. Quantitative Intuition (cognitive domain, scale question)	What is the relationship between Quantitative Intuition (QI) measures and demographics, personality traits, and individual measures like overconfidence and numeracy? Can digital twins capture Quantitative Intuition (QI) skills?	We found a moderate correlation between human responses to the individual QI scale and those of their twins, and a weaker relationship in assessments of the QI score of the organization they work(ed) for. Twins failed to mimic responses to two QI behavior questions. Twins tended to select the normative, high-QI behavior much more frequently than humans did.
18. Targeting Fairness Perceptions (social domain, preference measure, social cognition, attitude, valenced, different versions of question, scale question)	How do humans and twins perceive the fairness of demographic-based versus broad targeting?	At the aggregate level, the twins closely matched human judgments of the perceived fairness of demographic targeting, replicating the finding that it is viewed as less fair than broad targeting and producing similar average ratings across conditions.

Sub-study (Common Labels)	Pre-registered comparison(s)	Result(s)
19. User Behavior with Recommendation Algorithms (cognitive domain, human-tech interactions)	(i) How do self-reported average time spent on the platform differ across humans and their digital twin counterparts?<br>(ii) How does knowledge that recommendation algorithms depend on the user's past behavior compare between humans and their digital twin counterparts?<br>(iii) How does the prevalence of strategization (modifying your behavior to influence future recommendations) compare between humans and their digital twin counterparts?<br>(iv) How does the preference for user controls over recommended content compare between humans and their digital twin counterparts?	(i) Twins under-reported platform usage.<br>(ii) Twins were more aware of how recommendation algorithms work.<br>(iii) Twins were more likely to strategize.<br>(iv) Twins preferred more control over algorithms.

Table S1: **Pre-Registered Comparisons and Results.** We label individual *outcomes* rather than substudies for our meta-analysis. This table provides, for each substudy, the labels associated with at least half of the outcomes from that substudy.

Supplementary Text S2 Prompt Template (not pre-registered)

S2.1 Full Prompt Template

```
You are an AI assistant. Your task is to answer the 'New Survey Question' as
if you are the person described in the 'Persona Profile' (which consists
of their past survey responses).

Remain consistent with the persona's previous answers and stated traits.
Simulate their responses to new questions while accounting for human cognitive
limitations, uncertainty, and biases.

Follow all instructions provided for the new question carefully regarding
the format of your answer.

Persona Profile (This individual's past survey responses):
{Persona Profile}

New Survey Question & Instructions
(Please respond as the persona described above):
{Survey Questions}

Format Instructions:
In order to facilitate the postprocessing, you should generate a JSON object
by filling in Masks with the appropriate values in the following template
for {N} questions, where each question Q1, Q2, ... corresponds to the question
Q1, Q2, ... above.
{Format Templates}
```

S2.2 Example of Survey Questions

```
Q1:
Introduction: You are being invited to participate in a research study...
By selecting the 'Agree' option below I am agreeing to participate,
I have not given up any of the legal rights that I would have if I were not
a participant in the study.
Question Type: Single Choice
Options:
  1 - Agree
  2 - Disagree
Answer: [Masked]

Q2:
How important is it to you that your employer actively invests in environmental
sustainability (e.g., reducing carbon emissions, minimizing waste)?
```

```
Question Type: Single Choice
Options:
  1 - Not at all important
  2 - Slightly important
  3 - Moderately important
  4 - Very important
  5 - Extremely important
Answer: [Masked]

Q3:
Would you prefer to work at a company that is outspoken about social and
political issues, or one that remains neutral?
Question Type: Single Choice
Options:
  1 - Strongly prefer outspoken
  2 - Somewhat prefer outspoken
  3 - No preference
  4 - Somewhat prefer neutral
  5 - Strongly prefer neutral
Answer: [Masked]
......
```

S2.3 Example of Format Templates

```
{
    "Q1": {
        "Question Type": "Single Choice",
        "Answers": {
        "SelectedByPosition": Masked,
        // a number from 1 to 2, corresponding to the option position
        "SelectedText": "Masked"
        // a string, corresponding to the option text
        }
    },
    "Q2": {
        "Question Type": "Single Choice",
        "Answers": {
        "SelectedByPosition": Masked,
        // a number from 1 to 5, corresponding to the option position
        "SelectedText": "Masked"
        // a string, corresponding to the option text
        }
    },
....
```

Supplementary Text S3 Details for Persona Construction (not pre-registered)

In this section, we detail the construction of the various types of personas. That is, we provide the text that is inserted into the full prompt template (Section S2.1), as {Persona Profile}.

S3.1 Full Persona

Our full persona is built from the text-based questions and answers in Twin-2K-500, reflecting n atural l anguage i nteractions. T he T win-2K-500 d ataset c onsists of four waves, with wave 4 repeating questions from waves 1–3 for test–retest purposes. For persona construction, when a question appears in both waves 1–3 and wave 4, we use the wave 4 responses. The complete persona dataset can be accessed at https://huggingface.co/datasets/LLM-Digital-Twin/Twin-2K-500, specifically the 'persona_text' split within the 'full_persona' subset. An example of (portions of) a full persona is below:

```
Which part of the United States do you currently live in?

Question Type: Single Choice

Options:

1 - Northeast (PA, NY, NJ, RI, CT, MA, VT, NH, ME)

2 - Midwest (ND, SD, NE, KS, MN, IA, MO, WI, IL, MI, IN, OH)

3 - South (TX, OK, AR, LA, KY, TN, MS, AL, WV, DC, MD, DE, VA, NC, SC,
GA, FL)

4 - West (WA, OR, ID, MT, WY, CA, NV, UT, CO, AZ, NM)

5 - Pacific (HI, AK)

Answer: 2 - Midwest (ND, SD, NE, KS, MN, IA, MO, WI, IL, MI, IN, OH)

What is the sex that you were assigned at birth?

Question Type: Single Choice

Options:

1 - Male
```

2 - Female

Answer: 2 - Female

...

Here are a number of characteristics that may or may not apply to you. Please indicate next to each statement the extent to which you agree or disagree with that statement. I see myself as someone who...

Question Type: Matrix

Options:

1 = Disagree strongly

2 = Disagree a little

3 = Neither agree nor disagree

4 = Agree a little

5 = Agree strongly

1. Is talkative

Answer: 1 - Disagree strongly

2. Tends to find fault with others

Answer: 1 - Disagree strongly

3. Does a thorough job

Answer: 5 - Agree strongly

4. Is depressed, blue

Answer: 1 - Disagree strongly

5. Is original, comes up with new ideas

Answer: 4 - Agree a little

6. Is reserved

```
Answer: 4 - Agree a little

...

Please consider the following product category: Detergents - Heavy Duty
- Liquid. Suppose you are in a grocery store, and you see the following
product in that category: Purex Liquid Laundry Detergent Plus OXI, Stain
Defense Technology, 128 Fluid Ounces, 85 Wash Loads. The product is
priced at: \$5.98. Would you or would you not purchase this product?

Question Type: Single Choice

Options:

1 - Yes, I would purchase the product

2 - No, I would not purchase the product

Answer: 1 - Yes, I would purchase the product
```

S3.2 Persona Summary

Each twin in the full persona dataset averages about 30K tokens, making its simulation relatively slow and costly. To address this, we developed a shorter and more concise version called persona summary. In this version, we simplify the questions and record responses using distributional information across participants. For instance, instead of including the full set of Big Five questions, we represent the trait with a single value such as "score_extraversion = 2.125 (26th percentile)."

For simplification, wave 4 questions (which involved treatment-condition randomization) are excluded from the persona summary. As a result, the persona summary prompt is about 3K tokens in length—a substantial reduction compared to the full persona. Our results show that persona summary can serve as an efficient alternative when token and time budgets are constrained.

The complete persona dataset can be accessed at https://huggingface.co/datasets/LLM-Digital-Twin/Twin-2K-500, specifically the 'persona_summary' split within the 'full_persona' subset. An example of (portions of) persona summary is below:

```
The person's demographics are the following...

Geographic region: Midwest (ND, SD, NE, KS, MN, IA, MO, WI, IL, MI, IN,
OH)

Gender: Female
```

Age: 30-49

Education level: Some college, no degree

Race: Black

Citizen of the US: Yes

Marital status: Married

Religion: Nothing in particular

Religious attendance: A few times a year

Political affiliation: Independent

Income: $50,000-$75,000

Political views: Moderate

Household size: 3

Employment status: Part-time employment

The person's Big 5 scores are the following:

score_extraversion = 2.125 (26th percentile)

score_agreeableness = 4.556 (84th percentile)

wave1_score_conscientiousness = 4.556 (77th percentile)

score_openness = 3.5 (36th percentile)

score_neuroticism = 1.5 (15th percentile)

Openness reflects curiosity and receptiveness to new experiences, Conscientiousness indicates self-discipline and goal-directed behavior, Extraversion measures sociability and assertiveness, Agreeableness reflects compassion and cooperativeness, and Neuroticism captures emotional instability and susceptibility to negative emotions. Each score ranges from 1 to 5, and a higher score indicates a greater display of the associated traits.

...

S3.3 Demographics Persona

We also construct a demographics persona, which contains only demographic information. This version is derived by truncating the full persona to retain only 14 demographic variables: region, sex, age, education, race, citizenship, marital status, religion, religious attendance, political party, household income, political ideology, household size, and employment status. By design, the demographics persona always serves as a prefix of the full persona. An example is shown below:

```
Which part of the United States do you currently live in?

Question Type: Single Choice

Options:

1 - Northeast (PA, NY, NJ, RI, CT, MA, VT, NH, ME)

2 - Midwest (ND, SD, NE, KS, MN, IA, MO, WI, IL, MI, IN, OH)

3 - South (TX, OK, AR, LA, KY, TN, MS, AL, WV, DC, MD, DE, VA, NC, SC,
GA, FL)

4 - West (WA, OR, ID, MT, WY, CA, NV, UT, CO, AZ, NM)

5 - Pacific (HI, AK)

Answer: 2 - Midwest (ND, SD, NE, KS, MN, IA, MO, WI, IL, MI, IN, OH)

What is the sex that you were assigned at birth?

Question Type: Single Choice

Options:

1 - Male

2 - Female

Answer: 2 - Female

How old are you?

Question Type: Single Choice

Options:
```

1 - 18-29

2 - 30-49

3 - 50-64

4 - 65+

Answer: 2 - 30-49

What is the highest level of schooling or degree that you have completed?

Question Type: Single Choice

Options:

1 - Less than high school

2 - High school graduate

3 - Some college, no degree

4 - Associate’s degree

5 - College graduate/some postgrad

6 - Postgraduate

Answer: 3 - Some college, no degree

What is your race or origin?

Question Type: Single Choice

Options:

1 - White

2 - Black

3 - Asian

4 - Hispanic

```
5 - Other

Answer: 2 -- Black
```

We integrate the constructed personas into the prompt template (see Methods section) to perform our LLM simulations.

S3.4 Empty Persona

The *Empty Persona* specification does not provide any individual-level information in the prompts. Instead, {Persona Profile} is replaced with the placeholder `[Empty Persona Profile]`.

Specifically, the prompt used for all individuals is:

```
...
Persona Profile (This individual's past survey responses):
[Empty Persona Profile]
...
```

Supplementary Text S4 Distribution of Each Performance Metric Across Outcomes (not pre-registered)

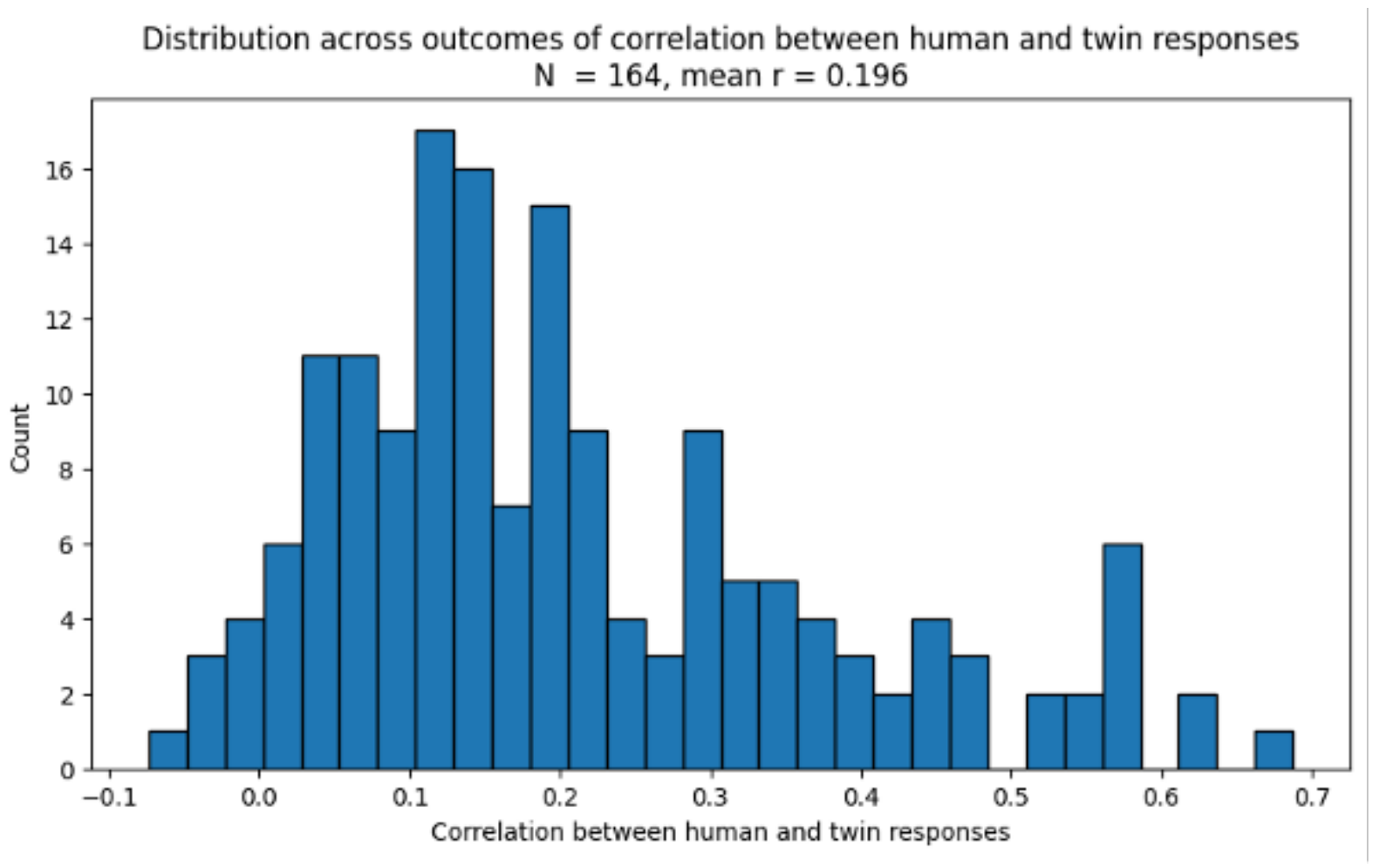

Fig. S1 **Distribution Across Outcomes of Correlation Between Human and Twin Responses.**

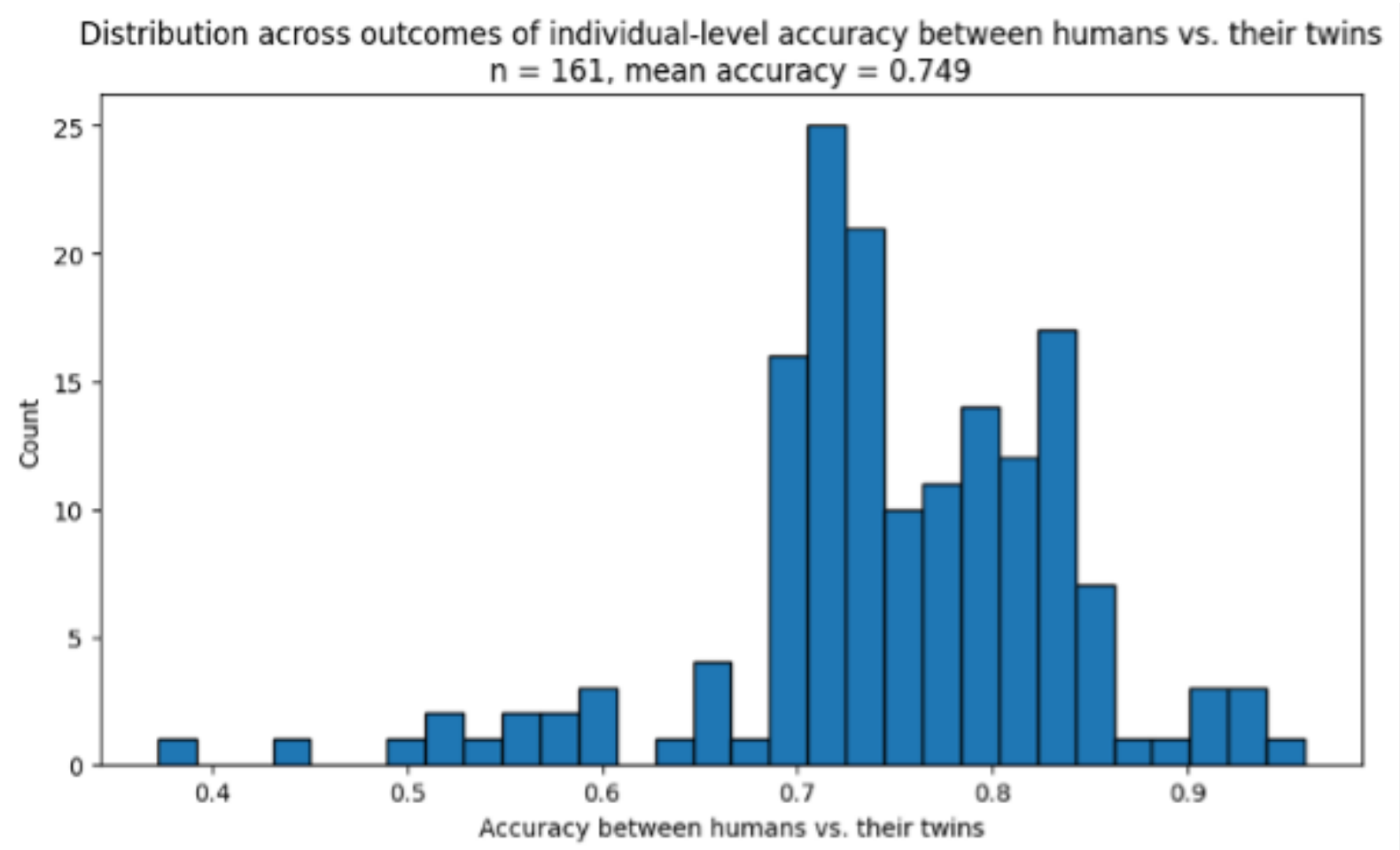

Fig. S2 **Distribution Across Outcomes of Individual-Level Accuracy Between Human and Twin Responses.**

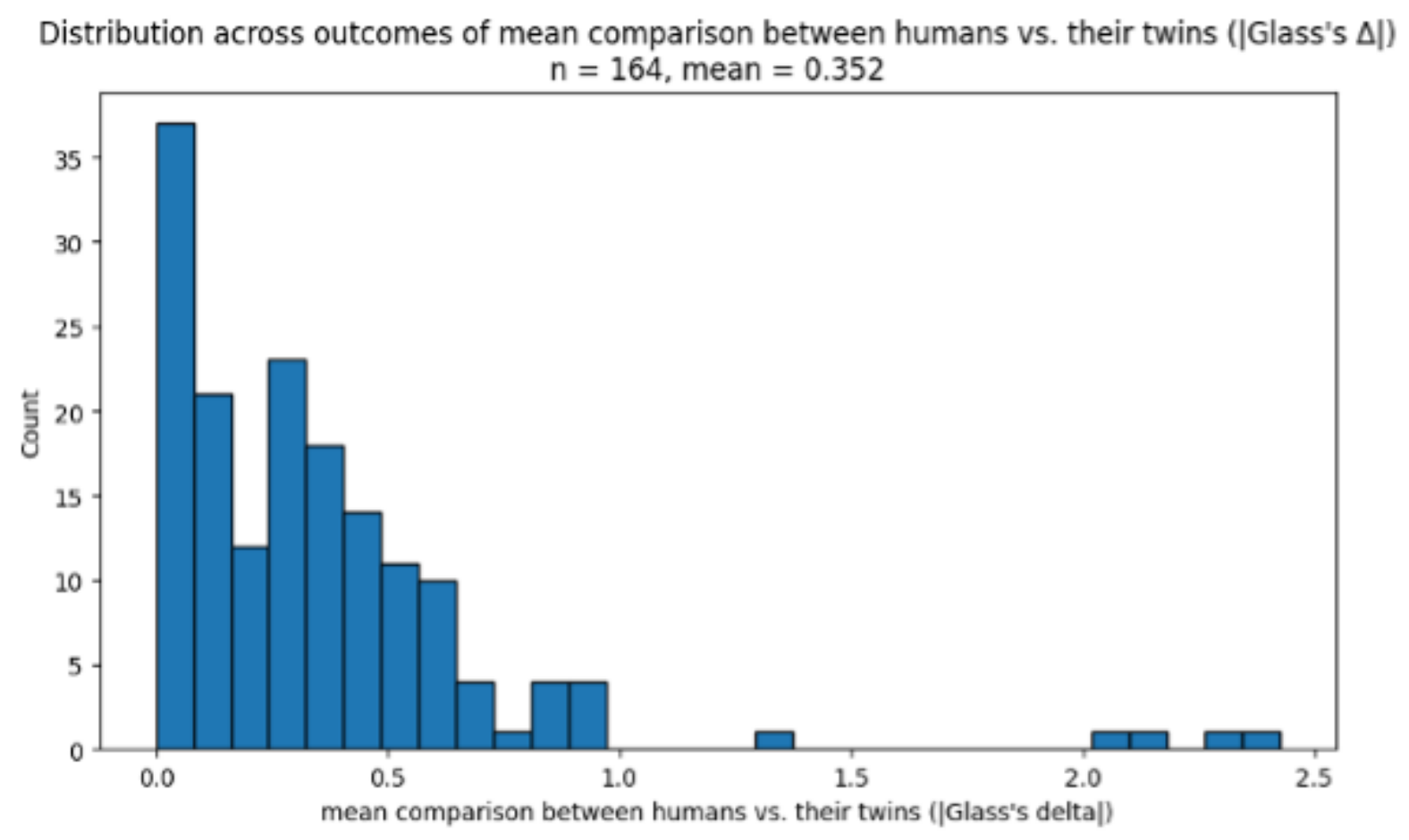

Fig. S3 **Distribution Across Outcomes of |Glass's Δ| Between Human and Twin Responses.**

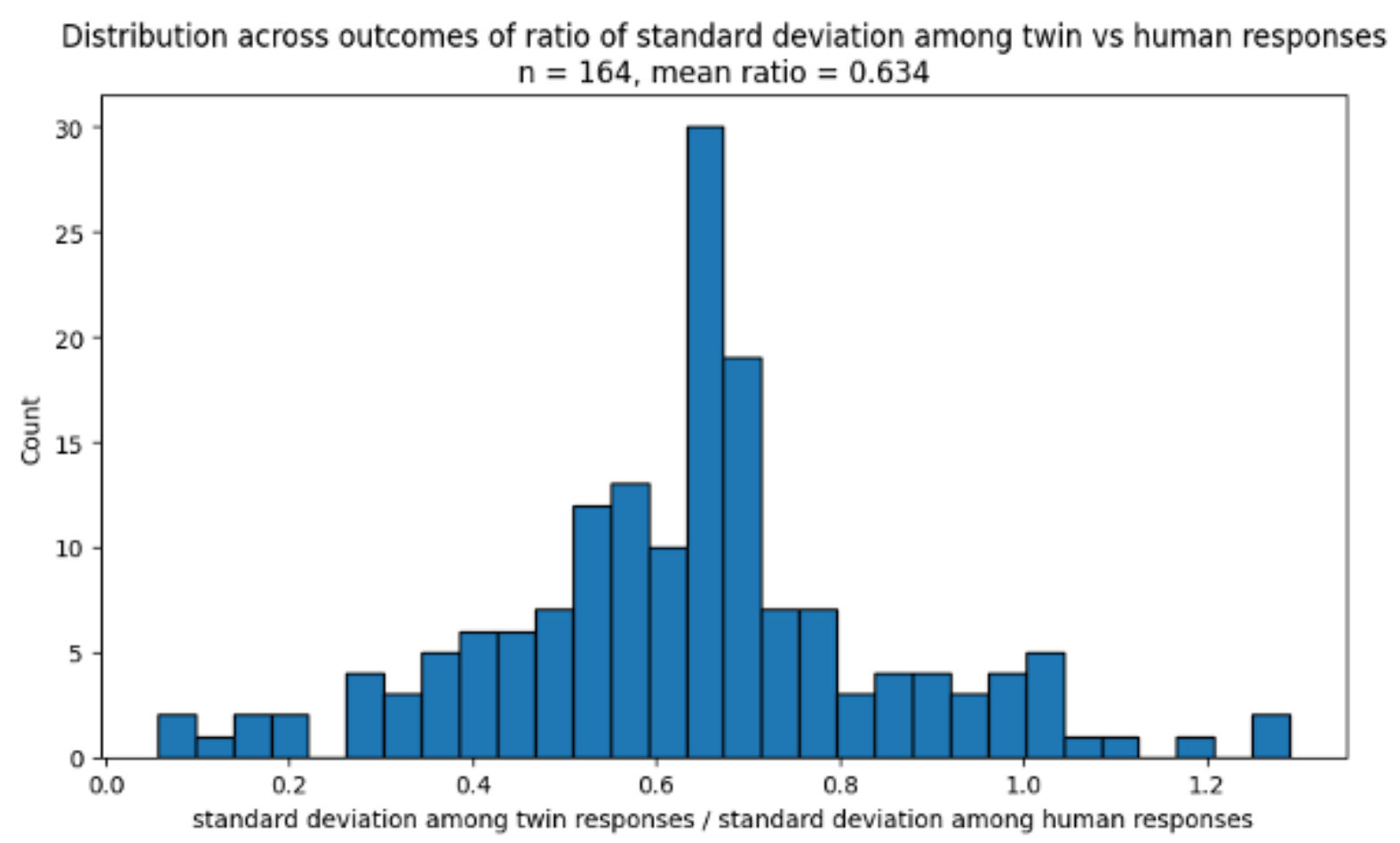

Fig. S4 **Distribution Across Outcomes of Ratio of Standard Deviation Between Human and Twin Responses.**

Supplementary Text S5 Digital Twin Performance Across Domains (pre-registered)

The categorical labels in our meta-analysis are not mutually exclusive; an outcome may be tagged with multiple labels. All labels are binary, except sample size. The labels are as follows (with examples from our data):

- **Social**
  - Social domain (104 outcomes): involves social topics (e.g., likelihood of sharing news content with others)
  - Conflict-related (15 outcomes): involves intra- or inter-personal conflict (e.g., confidence in ability to manage conflict)
  - Pro-social (36 outcomes): involves pro-social topics such as altruism or cooperation (e.g., paying higher taxes to improve healthcare for all people)
  - Social cognition (15 outcomes): involves social cognition such as asking the participant to judge the behavior of others (e.g., judging someone who donates to both political parties)
  - Personality measure (6 outcomes): assesses personality traits (e.g., consumer minimalism)
  - Social desirability (55 outcomes): has a socially desirable answer (e.g., willingness to be an organ donor)
- **Preferences/attitudes**
  - Attitude (33 outcomes): captures general attitudes toward a topic, issue, or person (e.g., whether most people can be trusted)
  - Political domain (12 outcomes): focuses on political content (e.g., whether pricing practices should be regulated by the government)
  - Preference measure (123 outcomes): measures the participant's preferences (e.g., preference between two room environments)
  - Behavioral intention (44 outcomes): captures future behavioral intentions (e.g., intention to apply for a job)
- **Cognitive skills/rationality**
  - Cognitive domain (79 outcomes): captures or assesses cognition (e.g., strategic content consumption on media platforms)
  - Test of rationality (10 outcomes): assesses rational decision-making (e.g., sensitivity to context effects)
- **Content evaluation**
  - Evaluating content (62 outcomes): requires evaluation of stimuli (e.g., predicting content of next book chapter)
  - Valenced (40 outcomes): involves positive versus negative judgments (e.g., judging the creativity of ideas)
- **Human–tech interactions**

  - Human–tech interactions (45 outcomes): concerns human interaction with technology (e.g., hiring algorithms)

- **Mechanical**
  - Replicates known human bias (5 outcomes): whether the outcome replicates a well-known human bias (e.g., default effect)
  - Different versions of question(s) (82 outcomes): question(s) wording varies across participants (e.g., across different conditions of an experiment)
  - Scale question (142 outcomes): uses Likert or similar rating scales (e.g., agreement with a statement)
  - Sample size (continuous): the number of participants on which the outcome is measured

We estimate a mixed linear model, including random intercepts for each sub-study. See Figure S5 for the results using correlation as the DV, and Figures S6-S8 for the results using the other performance metrics as DVs.

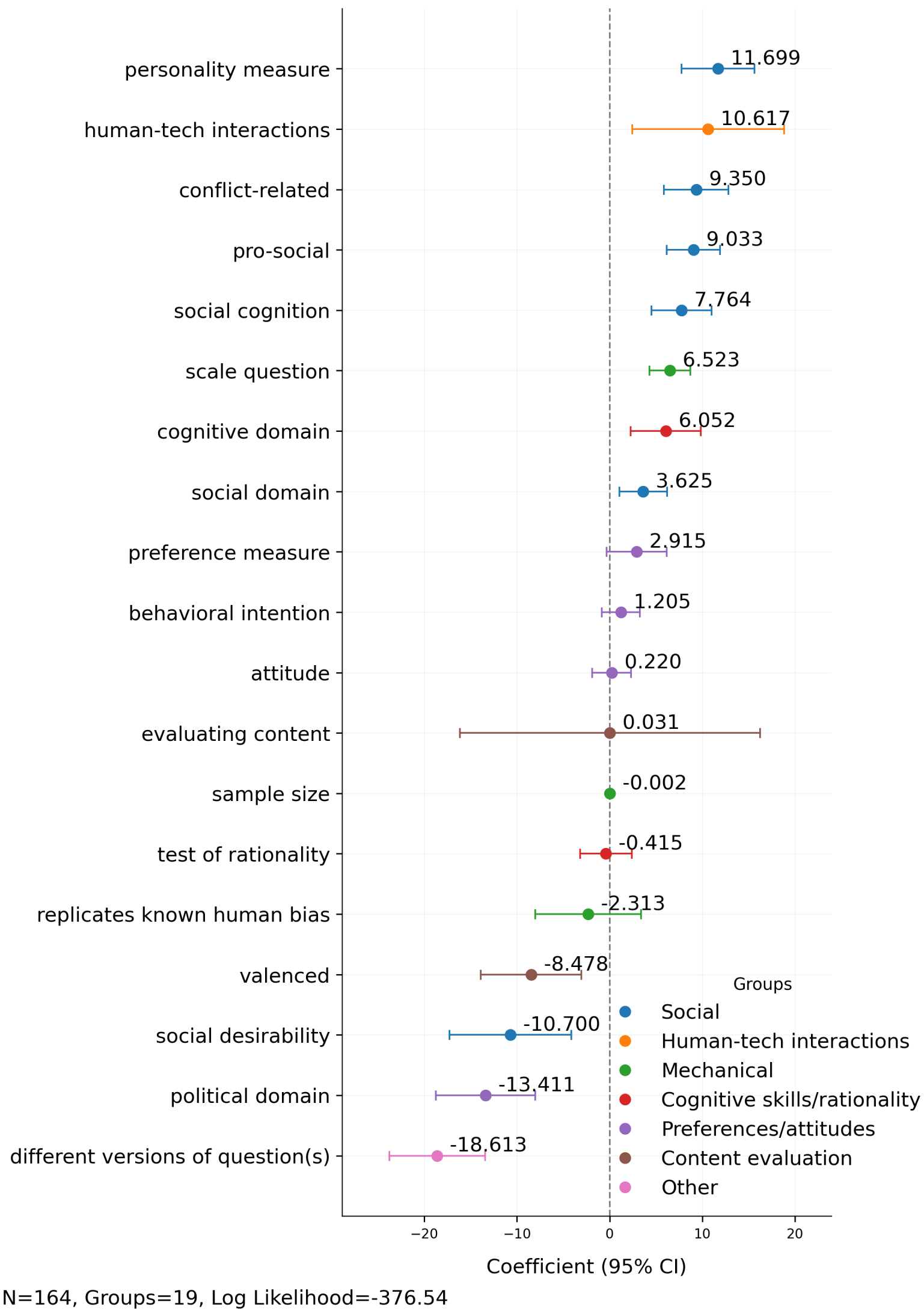

Fig. S5 Results from Meta-Analysis (Mixed Linear Model with z-transformed Correlation as Dependent Variable). Regression coefficients with 95% confidence intervals. The average correlation across all outcomes is 0.197. Correlation between responses from twins versus their human counterparts tends to be higher in social domains, except when social desirability is salient. Correlation tends to be higher in the cognitive domain and in domains related to human-technology interactions, but lower in the political domain and when providing valenced evaluations.

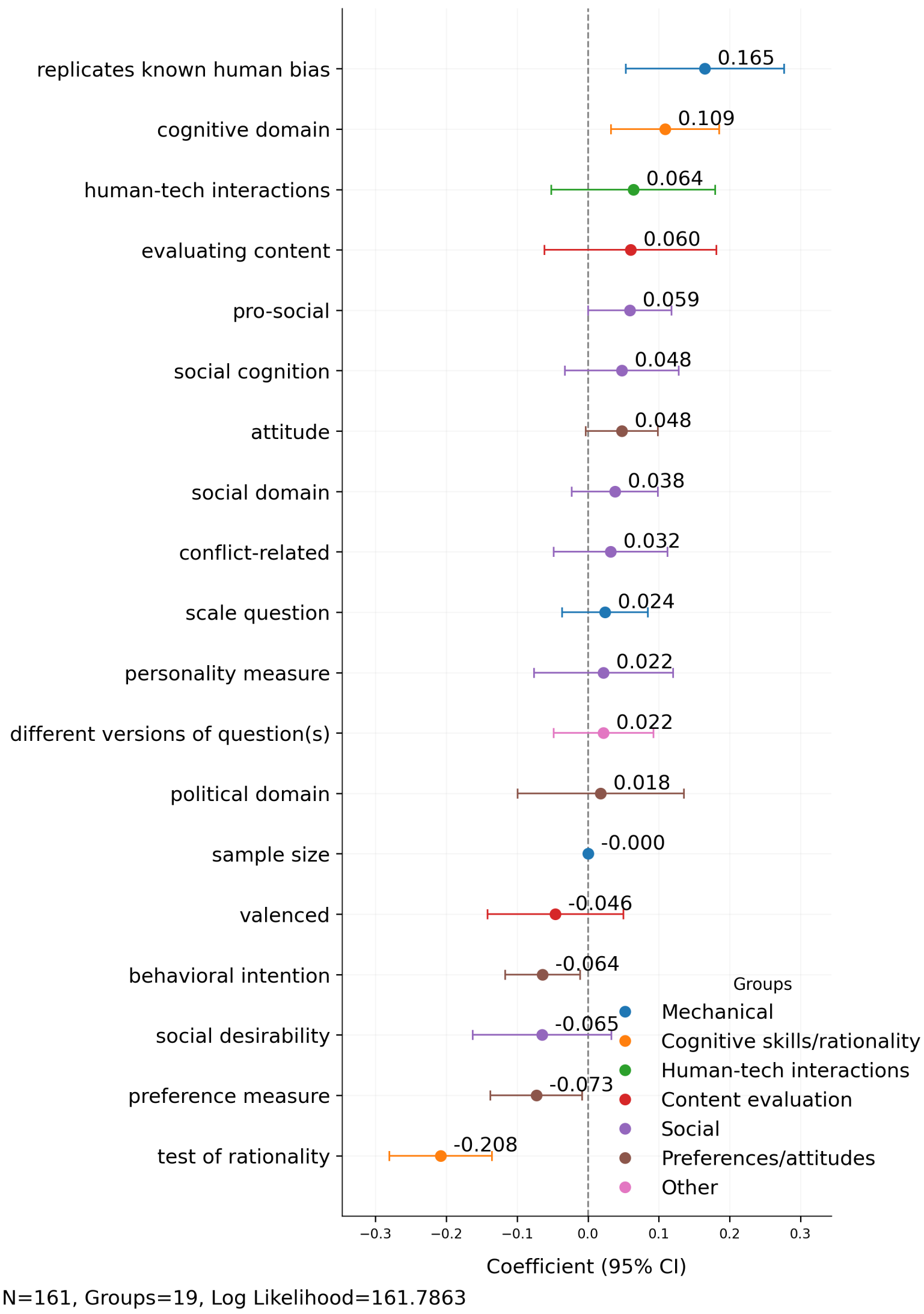

Fig. S6 Results from Meta-Analysis (Mixed Linear Model with Individual-Level Accuracy as Dependent Variable).

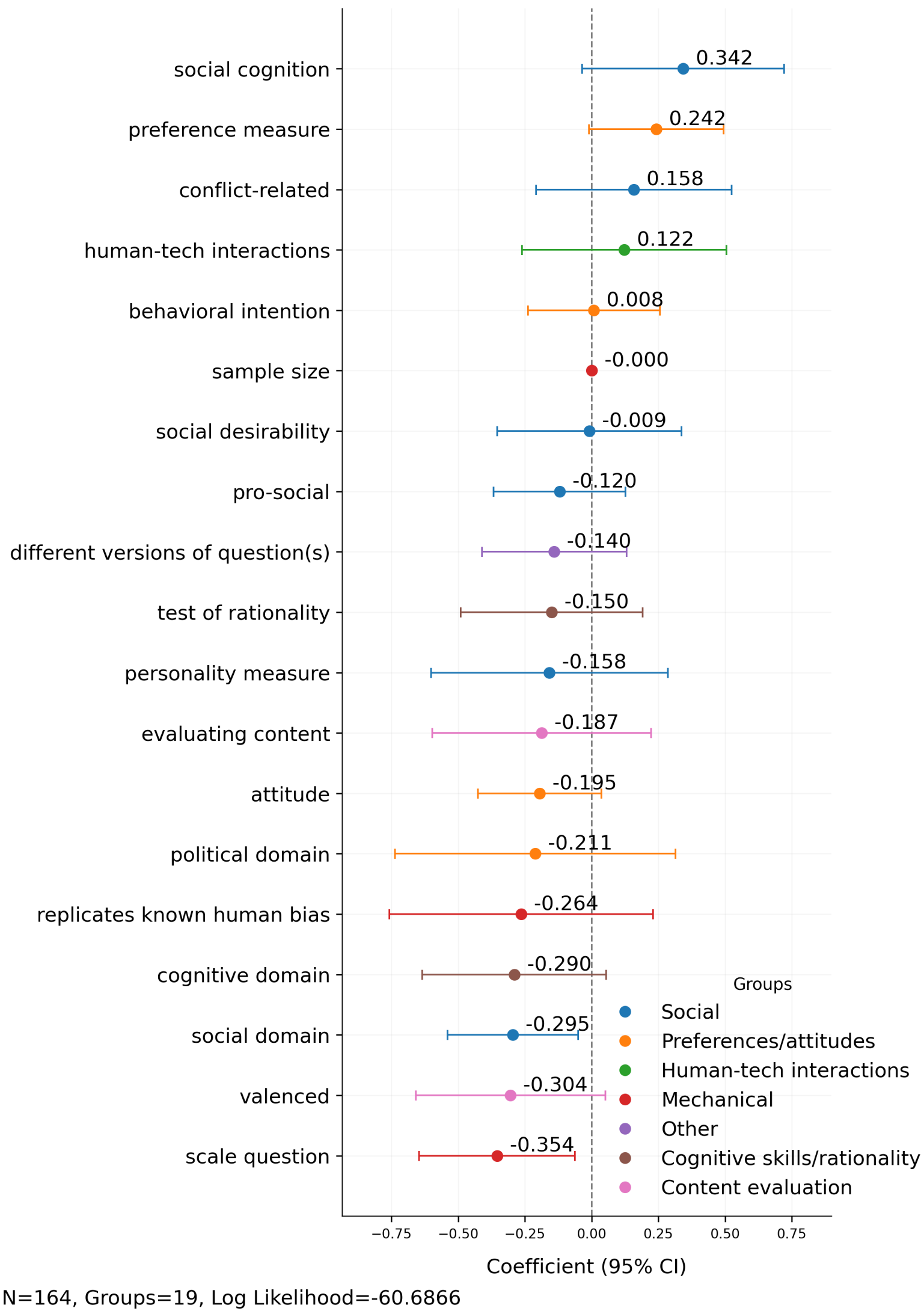

Fig. S7 **Results from Meta-Analysis (Mixed Linear Model with |Glass's Δ| as Dependent Variable).**

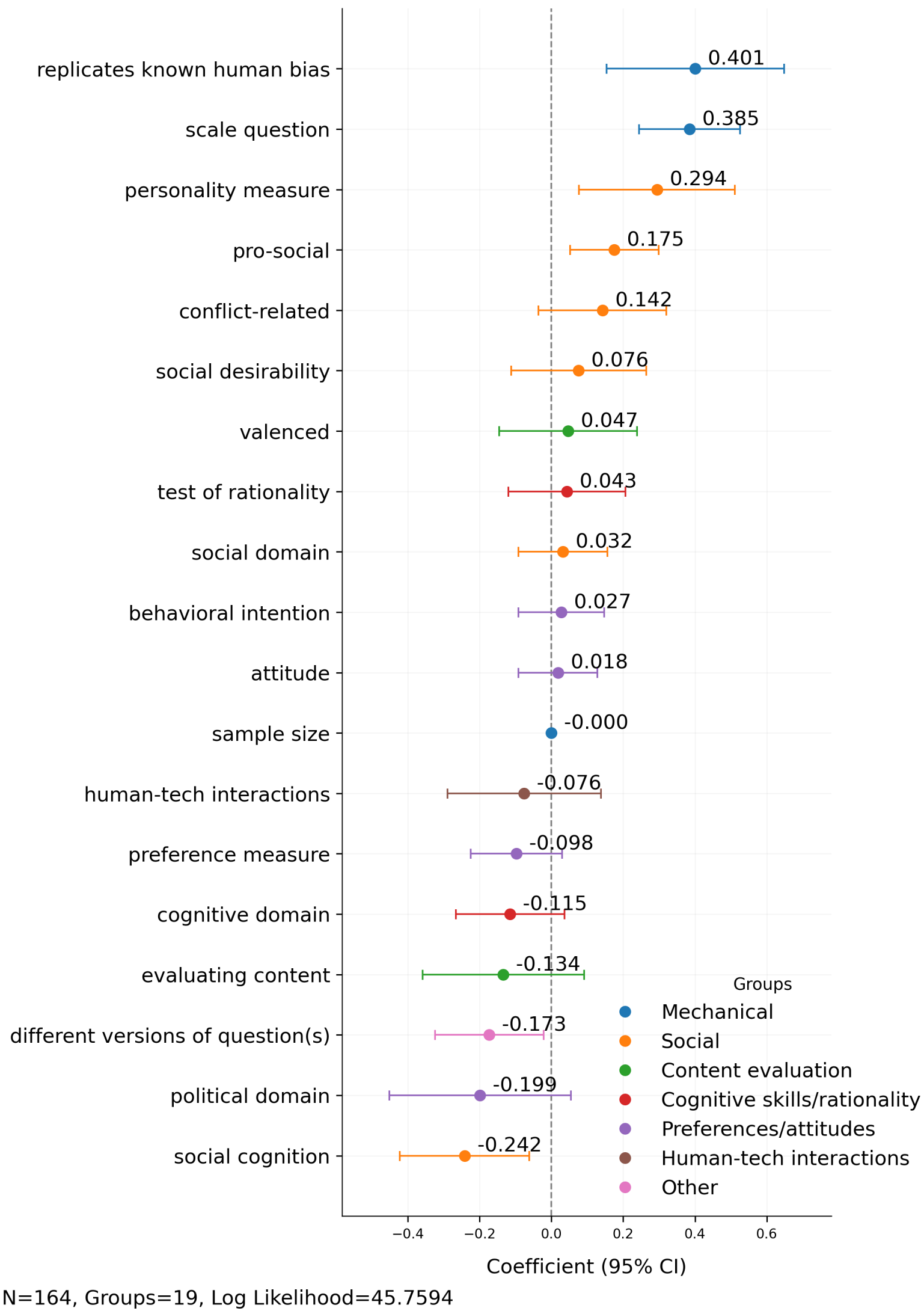

Fig. S8 Results from Meta-Analysis (Mixed Linear Model with std(twin) / std(human) as Dependent Variable).

Supplementary Text S6 Additional Partial Dependence Plots from XGBoost (not pre-registered)

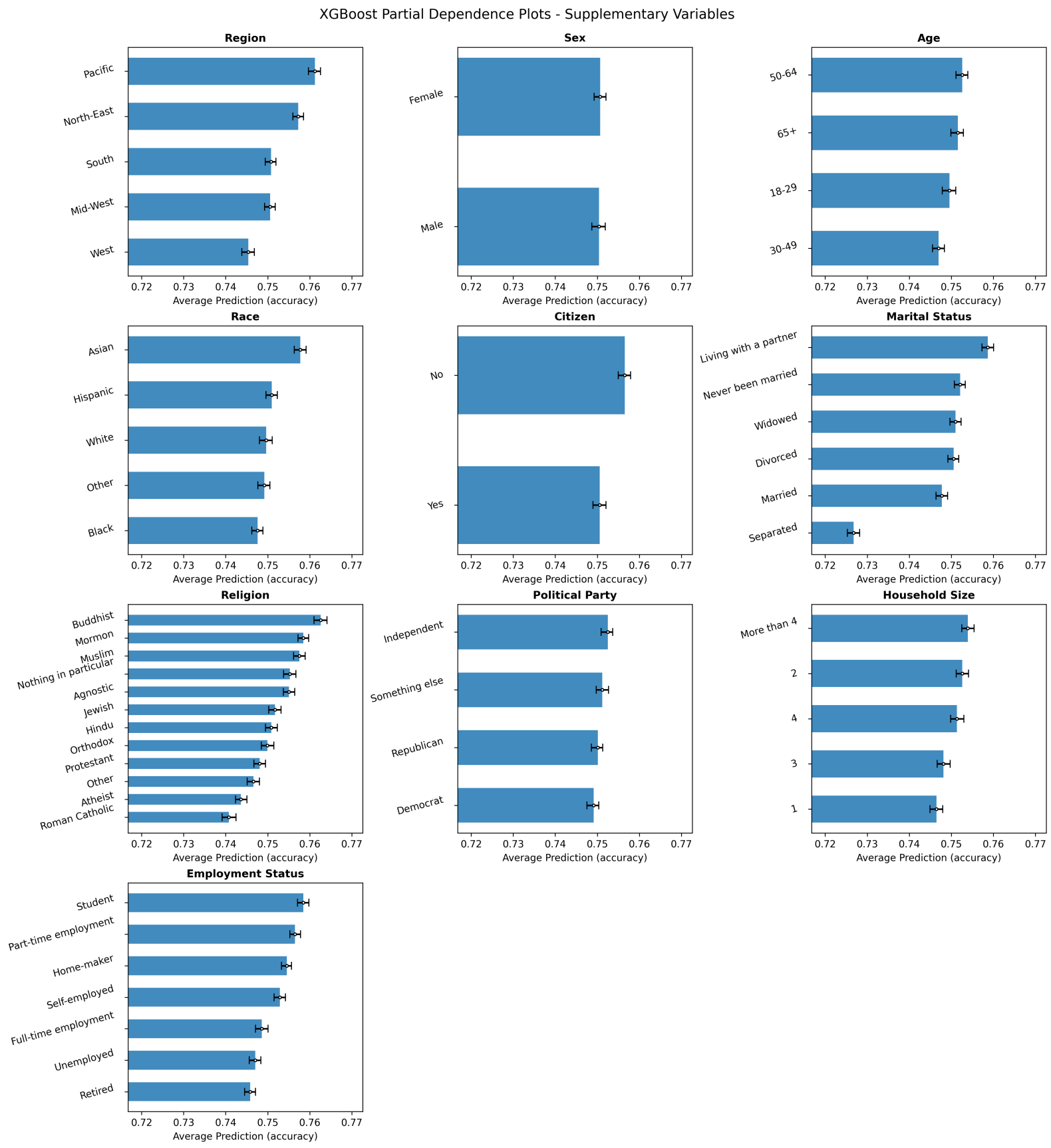

Fig. S9 Partial Dependence Plots from XGBoost.

Supplementary Text S7 Comparison Across Implementation Methods (not pre-registered)

S7.1 Persona summaries

We add as a benchmark an alternative way to construct digital twins, labeled "persona summary:" a concise, statement-based summary (approximately 13K characters) of the questions and responses with distributional information (e.g., Big 5 personality scores with percentile ranks rather than 44 detailed questions and answers on which the scores are based). Results are reported in Figure S10. We see that this version performs very similarly to the full persona, offering a viable lower-cost alternative.

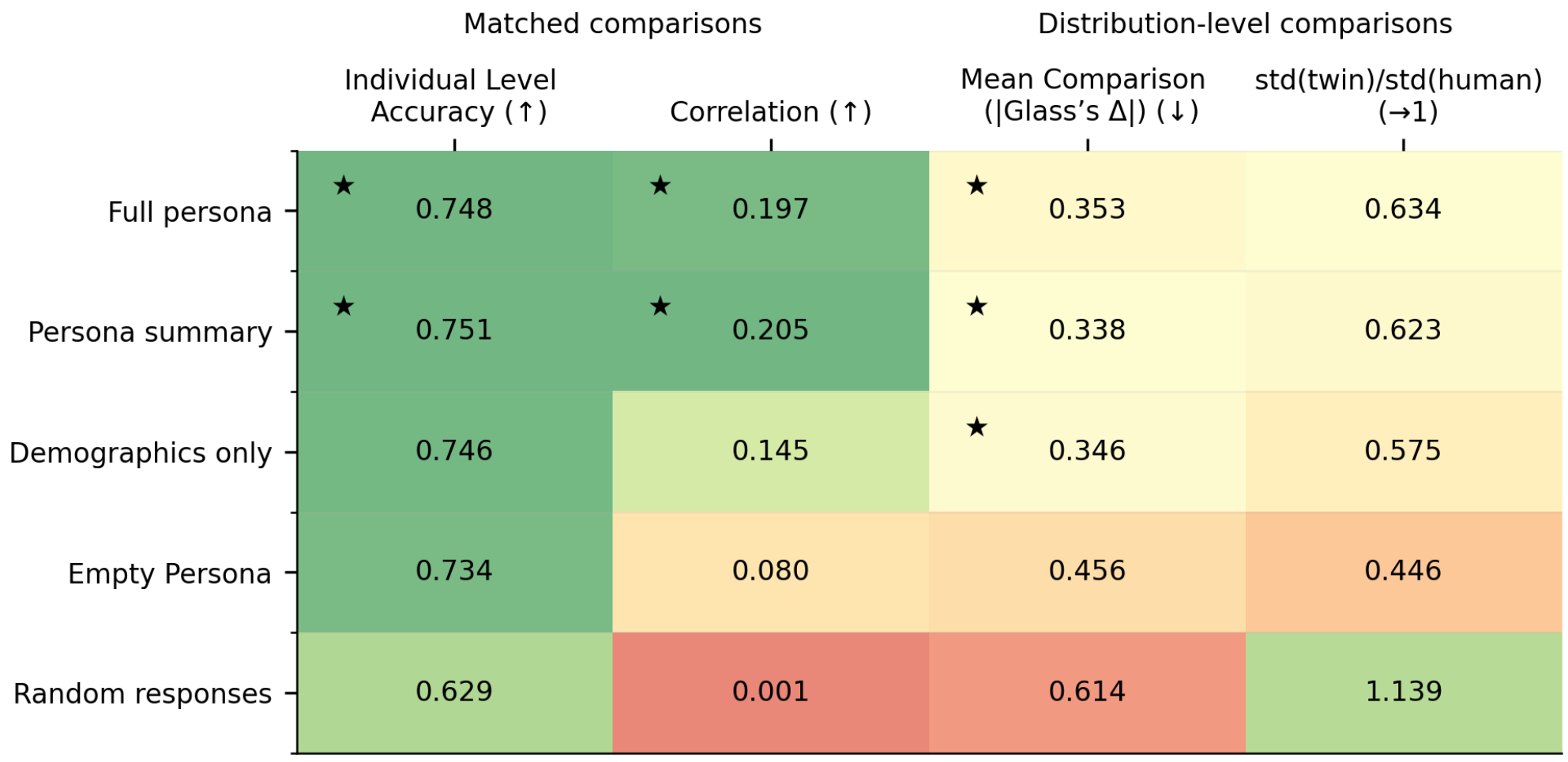

Fig. S10 Gains from Leveraging Individual-Level Data, Including Persona Summary as Benchmark. *: best performing benchmark, or not significantly different from best at p<0.05 (not applicable to ratio of standard deviations), after applying Bonferroni correcting for multiple comparisons in each column.

S7.2 Alternative base LLMs

We test GPT-5, Deepseek (deepseek-r1-0528), Gemini (gemini-2.5-flash, gemini-3-pro), and a fine-tuned GPT-4.1 as alternative base models. Full persona prompts were used by all benchmarks. The temperature for GPT-4.1 and its fine-tuned variants was set to the default value of 0.7. For the other models we selected the temperature that was expected to yield the best performance. We used a temperature of 0 for DeepSeek (deepseek-r1) and Gemini-2.5 (gemini-2.5-flash) because we found this helped GPT4.1. We used the default temperature of 1 for Gemini-3-pro, as Google recommends not changing the temperature of this model for better performance. We set

the temperature for GPT-5 at 1 due to the API restriction. All other API parameters for these benchmarks were kept at their default values.

The fine-tuned model was fine-tuned using the Twin-2K-500 dataset. Our approach consisted in using a conservative fine-tuning model that would (i) not use any information from our sub-studies and (ii) rely on treating all components of the twins as a unitary construct. That is, if a twin demonstrates consistency in reporting demographic characteristics, it should also display coherence when answering questions in other domains (e.g., attitudes, preferences, or behaviors). As such, for each participant, we created a training example consisting of:

1. A system instruction guiding the model to answer survey questions consistently with the individual's prior responses and realistic cognitive constraints,
2. A user message containing the Twin 2K-500 questions with all "Answer: [Masked]" at once,
3. An assistant message containing the same message as the user's but with the answers revealed.

This structure replicates the prompting approach we used for the mega-study. It required the model to infer the missing responses from contextual information while maintaining alignment with the participant's traits. Fine-tuning was performed on GPT-4.1 (release date 2025-04-14) using supervised learning with automatically selected hyperparameters. The fine-tuning was ultimately processed using 3 epochs, a batch size of 4 and a learning rate multiplier of 2. All sub-studies were conducted strictly out-of-sample, ensuring that no outcome measures used for evaluation overlapped with the fine-tuning training data. Note that the fine-tuned model required us to pre-process the input slightly to remove some whitespaces (given the token limit in fine-tuning which was set to 65,536 tokens) and to modify our prompting strategy when running the simulations on the sub-studies. Indeed, after fine-tuning, the model was no longer as proficient at following instructions and responses tended to have different formats. The prompts were modified to ensure the consistency in format.

Results are presented in Figure S11. We see that no other base model significantly outperforms GPT-4.1.

	Matched comparisons		Distribution-level comparisons	
	Individual Level Accuracy (↑)	Correlation (↑)	Mean Comparison (|Glass's Δ|) (↓)	std(twin)/std(human) (→1)
GPT-4.1	★ 0.748	★ 0.197	★ 0.353	0.634
GPT-5	★ 0.738	★ 0.193	0.467	0.633
Deepseek	★ 0.744	★ 0.200	★ 0.365	0.733
Gemini-2.5-flash	★ 0.740	★ 0.206	0.442	0.685
Gemini-3-pro	★ 0.740	★ 0.194	★ 0.362	0.827
GPT-4.1 fine tuned	0.704	0.144	★ 0.360	1.060

Fig. S11 Alternative Base LLMs (Combined with Full Persona). *: best performing benchmark, or not significantly different from best at $p<0.05$ (not applicable to ratio of standard deviations), after applying Bonferroni correcting for multiple comparisons in each column.

S7.3 Alternative Temperature

We replicate the main comparison in the paper (i.e., with GPT-4.1) with a temperature of 0 instead of the default temperature. Figure S12 displays the results, including the full persona with default temperature as baseline. We see that when temperature is 0, like when temperature is set to default, digital twins based on full personas perform significantly better than generic twins based on demographics on correlation, and not significantly differently on individual-level accuracy. We also see that performance is overall improved when temperature=0.

	Matched comparisons		Distribution-level comparisons	
	Individual Level Accuracy (↑)	Correlation (↑)	Mean Comparison (|Glass's Δ|) (↓)	std(twin)/std(human) (→1)
Full persona (default temp.)	★ 0.748	0.197	★ 0.353	0.634
Full persona (temp. 0)	★ 0.752	★ 0.232	★ 0.348	0.615
Persona summary (temp. 0)	★ 0.749	0.179	★ 0.328	0.603
Demographics only (temp. 0)	★ 0.748	0.147	★ 0.337	0.557
Empty Persona (temp. 0)	0.736	0.048	0.446	0.377

Fig. S12 Gains from Leveraging Individual-Level Data (Temperature=0). *: best performing benchmark, or not significantly different from best at $p<0.05$ (not applicable to ratio of standard deviations), after applying Bonferroni correcting for multiple comparisons in each column.

Supplementary Text S8 Centaur Experiments (not pre-registered)

The state-of-the-art LLMs we used as based models are general-purpose models (e.g., GPT-4.1). A natural question arises: *what if an LLM fine-tuned specifically for human behavioral prediction was used as base model for the creation of digital twins?* One such potential base model is Centaur [23], a 70B-parameter model fine-tuned from Llama-3.1-70B using data from over 60,000 participants, encompassing 10 million choices across 160 behavioral experiments.

We use the Centaur model `marcelbinz/Llama-3.1-Centaur-70B` from Hugging Face with BF16 precision. We evaluate Centaur's performance on our 19 studies using the same setup as other LLMs, with one adjustment: we append `### Output \n '''json` to the end of each prompt instruction to encourage JSON-formatted outputs. After experimenting with several formatting strategies, we found this approach to yield the most consistent results.

In order to disentangle the impact of leveraging individual-level information from a panel of participants vs. using an LLM fine-tuned on a large cross-sectional dataset as a base model, we follow a 2 (Lllama vs. Centaur) x 2 (empty persona vs. persona summary) experimental design and test the following configurations:

- **Centaur Persona Summary:** Centaur model prompted with Persona Summary instructions.
- **Centaur Empty Persona:** Centaur model prompted with Empty Persona instructions (no persona information).
- **Llama Persona Summary:** Base Llama-3.1-70B model prompted with Persona Summary instructions.
- **Llama Empty Persona:** Base Llama-3.1-70B model prompted with Empty Persona instructions.

We use the **Persona Summary** instead of the Full Persona to avoid context-length issues that degrade Centaur's performance. Temperature is set to 0 for all models.

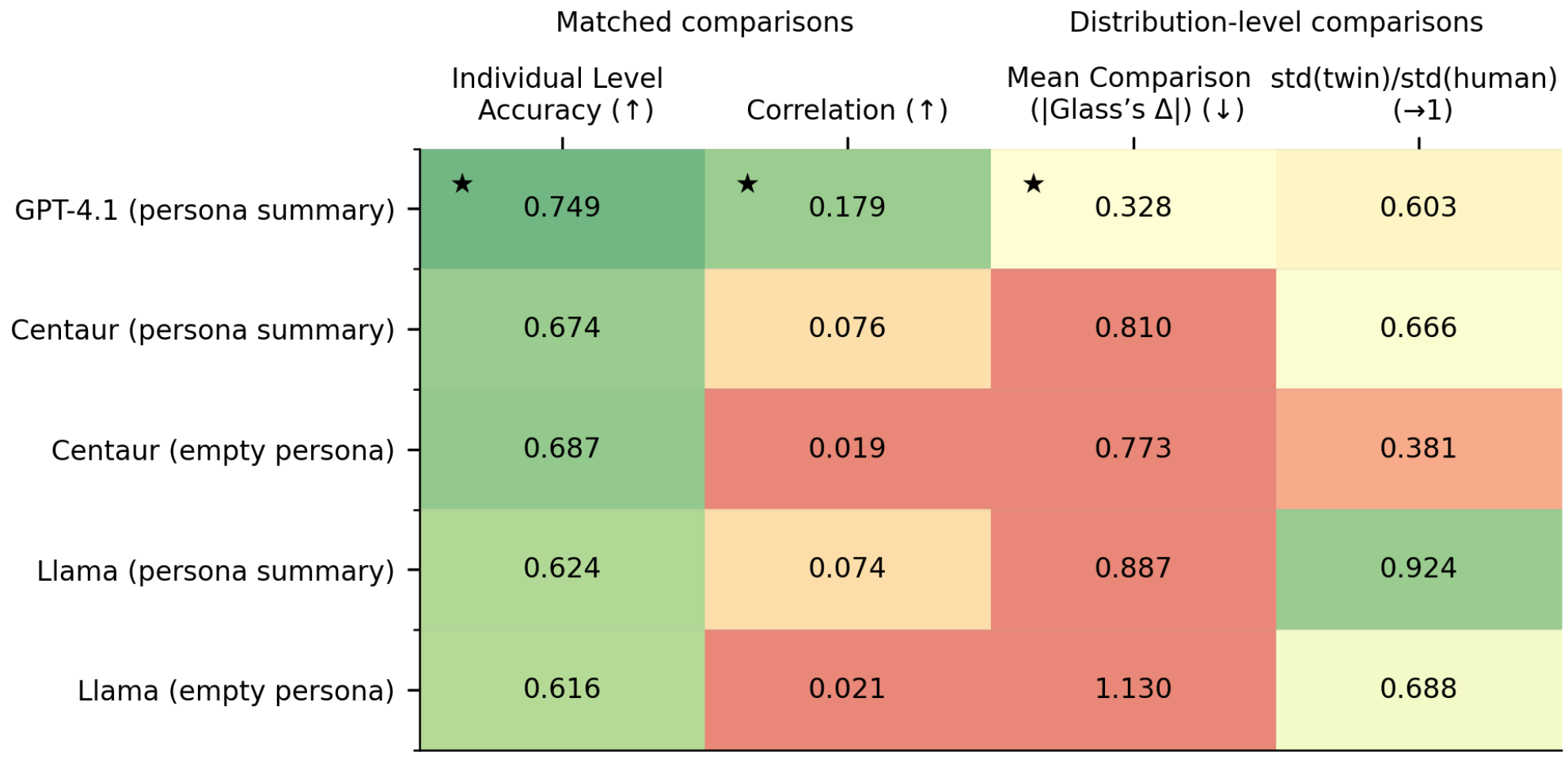

Fig. S13 Performance Comparison for Centaur and Llama Models Under Different Persona Configurations. *: best performing benchmark, or not significantly different from best at $p<0.05$ (not applicable to ratio of standard deviations), after applying Bonferroni correcting for multiple comparisons in each column.

Figure S13 displays the results, including GPT-4.1 (persona summary) as a reference. We separately discuss results related to the choice of base model vs. the use of individual-leevel information.

Base model: Centaur vs. Llama vs. GPT-4.1. Centaur outperforms its base model, Llama-3.1-70B, in terms of accuracy. However, the performance of digital twins based on the Centaur base model remains below that of state-of-the-art closed-source models such as GPT-4.1. Several factors may explain this gap:

1. **Prompt formatting:** Centaur was trained to answer single questions, whereas our evaluation involves multi-question inputs.
2. **Out-of-distribution testing:** Our 19 studies include scenarios that may differ significantly from Centaur's training data.
3. **Limited persona adaptation:** Centaur was not trained to explicitly utilize persona information, which may limit its ability to model human heterogeneity.

Addressing these issues could further improve Centaur's behavioral modeling capability—we leave them for future work.

Individual-level information: persona summary vs. empty persona. Despite lower overall accuracy, both Llama and Centaur exhibit improved *correlations* when persona information is included. This suggests that providing even summarized persona data helps models better capture individual-level heterogeneity, reinforcing the importance of collecting richer personal context in behavioral prediction tasks.

Supplementary Text S9 Survey of Expectations of Digital Twin vs. Human Behavior (pre-registered)

To assess expectations of human and digital twin responses, we asked respondents to estimate the average treatment effect from five experiments (tasks) in our data, chosen a priori to capture distinct paradigms (default effects, affective priming, targeting fairness, perceived privacy violation, and idea evaluation).[1] We recruited 104 respondents from LinkedIn and the Society for Judgment and Decision-Making (SJDM) listserv; 36 were excluded following preregistered criteria (completion times $<1/3$ or >3x the median, repetitive slider responses, or excessive minimum/maximum responses), leaving 68 in the final sample, including 27 academics, 15 managers (mid-level to C-suite) and 20 students.

For each task, respondents were given a brief description and shown human and twin mean values from the original survey in the control condition, then asked to predict the mean in a treatment condition (opt-out vs. opt-in defaults for green energy, gratitude ratings after a neutral vs. gratitude-writing prompt, fairness ratings for non-targeted vs. targeted ad campaigns, privacy ratings for no ads/tracking vs. personalized ads with cross-site tracking, and creativity ratings of human- vs. AI-generated app ideas).

As shown in Figure S14, predicted treatment effects were consistently more accurate for humans than for digital twins. In all five tasks, the observed average treatment effect among humans fell within the 95% confidence intervals of the predicted average treatment effect. By contrast, the confidence interval did not include the observed mean for three of the five digital twin predictions. The creation of this figure was not pre-registered.

[1] Pre-registered as https://aspredicted.org/65ty-73sp.pdf.

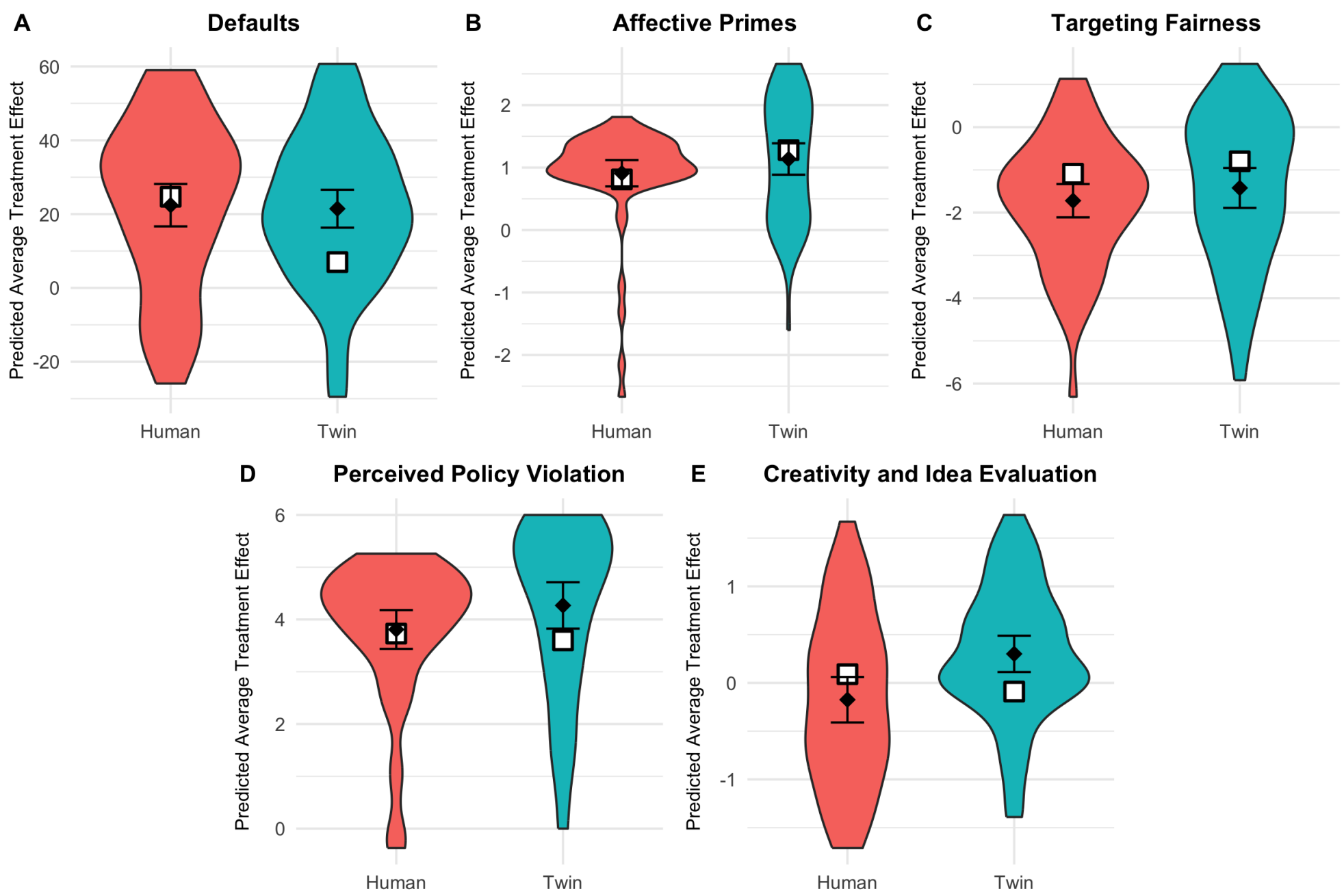

Fig. S14 Predicted Average Treatment Effects for Humans and Digital Twins across Five Tasks. Black dots and error bars represent the mean predicted average treatment effects and 95% confidence intervals. White squares represent the mean treatment effect actually observed in the sample.

To formally test the difference between human and digital twin prediction accuracy, we estimated a linear mixed-effects model (as pre-registered):

$$err_{it} = \beta_0 + \beta_1 Human_{it} + \beta_2 Task_{it} + (1|ResponseId_i) + \epsilon_{it}$$

where err_{it} is the prediction error for respondent i on task t (abs(predicted value in treatment condition - observed value in treatment condition)/(observed value in treatment condition)), $Human_{it}$ indicates predictions for human vs. digital twins, $Task_{it}$ indexes the five tasks, $(1|ResponseId_i)$ is a random intercept for each respondent, and ϵ_{it} is the residual. Results confirmed significantly lower errors for predictions of human responses on average ($\beta =$ -0.072, SE $=$ 0.011, p $<$ 0.001) (see Table S2).

**Effect**	**Estimate**	**Std. Error**	**t value**	**Pr(> $\|t\|$)**
(Intercept)	0.4093	0.01482	27.62	5.552×10^{-82}
Human	-0.07244	0.01052	-6.887	9.784×10^{-12}
taskaffPrime	-0.2555	0.01619	-15.78	1.413×10^{-50}
tasktargFair	-0.1645	0.01642	-10.02	1.222×10^{-22}
taskPPV	-0.1135	0.01666	-6.811	1.611×10^{-11}
taskideaEval	-0.1620	0.01676	-9.667	2.939×10^{-21}
**Random Effects and Model Summary**				
σ^2				0.03
τ_{00}				0.00
ICC				0.11
N (ResponseId)				61
Observations				1118
Marginal R^2 / Conditional R^2				0.200 / 0.291

Table S2 Linear Mixed-Effects Regression of Respondent Prediction Errors across Prediction Targets and Tasks.

Supplementary Text S10 Detailed Information on Each Sub-Study

All the studies were conducted on Prolific with human participants. Each study was subsequently run on their corresponding digital twins. Respondents and their twins were always assigned to the same condition(s) ensuring a fair 1-to-1 comparison.

S10.1 Accuracy Nudges for Misinformation

S10.1.1 Main Questions/Hypotheses

The spread of misinformation across social media is a growing public policy concern, with severe implications for health, politics, and science. Recent work suggests that people care about being accurate but share misinformation because the social media context focuses their attention on other factors [38]. The proposed solution for inattention-based misinformation sharing is to prime accuracy before the decision to share information. Research testing this account [39] demonstrates that asking people to rate the accuracy of unrelated headlines or state the extent to which they agree that it is important to share accurate content on social media before sharing improves truth discernment: that is, reduces sharing of false headlines compared to true headlines. The current study aims to replicate this pattern with human participants, and test whether their digital twins respond similarly to these accuracy nudges.

Specifically, this study aims to replicate study 5 of [39], using a different set of headlines. While [39] demonstrate the effectiveness of accuracy nudges in the context of political news headlines, the current study utilizes their paradigm to test the effectiveness of accuracy nudges in the context of non-political entertainment news headlines. The decision to sharing entertaining news may provoke consideration of how entertaining the headline is, beyond considerations on the accuracy of the news and trustworthiness of the sources. Indeed, people generally prefer and are most likely to engage with entertaining news [40–42] and interesting or emotionally arousing word-of-mouth [43–45]. Testing the effectiveness of accuracy nudges for entertaining headlines is therefore an important extension of the prior work.

S10.1.2 Methods

A total of 1,003 human participants on Prolific (and their digital twins) took part in the study (50% female, 50% male, 0% other; $M_{\text{age}} = 49.37$, $SD = 14.55$), following the protocol proposed by [39]. Participants were randomly assigned to one condition in a 2 (Headline Veracity: False vs. True; within-subjects) $\times$ 4 (Intervention: Control vs. Active Control vs. Treatment vs. Importance Treatment; between-subjects) mixed design (each human and their digital twin were assigned to the same condition). All participants were asked to indicate how likely they would be to share 16 headlines, displayed in random order, as the dependent variable. Participants in the Control condition proceeded directly to this task, while participants in the Active Control, Treatment, and Importance Treatment conditions were first asked to complete the following separate tasks:

Active Control: Participants were first told that they would help pretest actual news headlines for future studies. They were then shown a headline and asked to rate, “In your opinion, is the above headline funny, amusing, or entertaining?” (1-Extremely unfunny, 6-Extremely funny). They were randomly assigned to see one of four headlines—two of which were true and two of which were false. The stimuli were the same as [39], with the exception that images were not included, given that the digital twins cannot process visual images.

Treatment: Similarly, participants were told that they would help pretest actual news headlines for future studies. They were then shown two headlines in randomized order and asked to rate, “In your opinion, is the above headline accurate?” (1-Extremely inaccurate, 6-Extremely accurate). They were randomly assigned to see one of four headlines—two of which were true and two of which were false. The stimuli were the same as [39], again with the exception that images were not included.

Importance Treatment: Participants were simply asked, “Do you agree or disagree that ‘it is important to only share content on social media that is accurate and unbiased’?” (1-Strongly agree, 6-Strongly disagree).

All participants then responded to the dependent variable. Participants were presented with 16 news headlines in randomized order. For each headline, participants were asked to indicate, “If you were to see the above article on social media, how likely would you be to share it?” (1-Extremely unlikely, 6-Extremely likely). All headlines were pretested to be entertaining and interesting, but not identity relevant. Eight of the headlines were inspired by real headlines from the websites of trustworthy news organizations. These headlines were paired with generally trustworthy news sources. The remaining 8 headlines were false headlines. These headlines were paired with generally untrustworthy news sources. Source trustworthiness perceptions were confirmed at the end of the survey. Table S3 provides the headline and source pairings.

**Source**	**Headline (True or False)**
The Funny Times	1. “Rare Pink Bananas Discovered, Touted as Nature’s Cotton Candy” (F)<br>2. “The Real Jurassic Park? Dinosaur DNA Successfully Extracted from Fossil” (F)
Quora.com	3. “Researchers Develop Plants That Emit Enough Energy to Power a Tiny-House” (F)<br>4. “Innovative Study Finds Dolphins Can Be Trained to Detect Cancer” (F)
The National Enquirer	5. “The Mystery of the Bermuda Triangle: New Theory Suggests Surprising Explanation” (F)<br>6. “Scientists Discover a New Species of Glow-in-the-Dark-Bees in the Amazon” (F)
Reddit.com	7. “New Fossil Discovery Suggests Rat-Sized Elephants Once Inhabited the Earth” (F)<br>8. “Biologists Stumble Upon a Singing Spider Species, Dubbed ’Nature’s Vocalweaver”’ (F)

**Source**	**Headline (True or False)**
The Wall Street Journal	9. “World’s Smallest Gold Coin Features Albert Einstein Sticking Out Tongue” (T)
	10. “Authorities Scramble To Find Stolen Solid Gold Toilet” (T)
The Economist	11. “Applications To Become Japanese Billionaire Yusaku Maezawa’s Girlfriend Have Topped 20,000” (T)
	12. “Woman Boards Flight to Find Her Seat Assignment Is In The Plane’s Bathroom” (T)
PBS News	13. “Kansas Man Asks Judge To Allow Him To Have Sword Fight With Ex-Wife” (T)
	14. “Teen Discovers Rare New Planet 3 Days Into NASA Internship” (T)
BBC News	15. “Two Elderly Men Sneak Out Of Nursing Home To Attend Heavy Metal Festival” (T)
	16. “Spotify Launches Playlists For Dogs Left Home Alone” (T)

Table S3: **Headline and Source Pairings.** True and False headlines are indicated by (T) and (F), respectively. Untrustworthy sources include: The Funny Times, Quora.com, The National Enquirer, and Reddit.com. Trustworthy sources include: The Wall Street Journal, The Economist, PBS News, and BBC News.

Participants in the Control, Active Control, and Treatment condition next responded to the same question shown earlier to participants in the Importance Treatment condition: “Do you agree or disagree that ‘it is important to only share content on social media that is accurate and unbiased’?” (1-Strongly agree, 6-Strongly disagree). All participants then responded to exploratory questions intended to capture stated social media sharing preferences. As a measure of goal prioritization when sharing entertaining news, participants were first asked to indicate, “When you share information with others on social media, is it more important that the information is:” (1-Verifiably correct, 6-Entertaining). Participants then indicated, “Would you ever consider sharing news like you saw in the study today on social media?”.

All participants then indicated their perceptions of source trustworthiness (“How trustworthy (from 0–100%) do you think this source is?”) for each source, presented in randomized order. Participants across conditions rated sources in the True headline veracity condition to be more trustworthy than sources in the False condition ($M_{\text{True}} =$ 72.34%, $SD = 18.31$; $M_{\text{False}} = 28.28\%$, $SD = 14.00$), $t(2005) = 96.12$, $p < .001$, $d = 2.69$. Finally, participants indicated whether they responded randomly at any point in the survey (0.40% = Yes, 99.60% = No) and whether they searched the internet for any of the headlines (0.40% = Yes, 99.60% = No) before being debriefed, thanked, and paid.

S10.1.3 Results – Pre-registered Analyses

A linear mixed-effects regression was estimated[2] with participant as a random effect, average sharing intentions as the dependent variable, and Participant Type (Human = 0, Digital Twin = 1), Headline Veracity (False = 0, True = 1), Intervention (Control = 0, Active Control = 1, Treatment = 2, Importance Treatment = 3), and their interactions as predictors. The results revealed a significant main effect of Participant Type ($F(1, 2997) = 33.41$, $p < .001$), a non-significant main effect of Headline Veracity ($F(1, 2997) = 1.24$, $p = .27$), along with a significant main effect of Intervention ($F(3, 2997) = 8.89$, $p < .001$), qualified by a significant three-way interaction ($F(3, 2997) = 5.75$, $p < .001$), indicating that, at an aggregate level, accuracy nudges affected how the digital twins shared true vs. false headlines differently than humans. Figure S15 shows average sharing intentions across all conditions for humans (Panel A) and their digital twins (Panel B). See Table S4 for the full regression results.

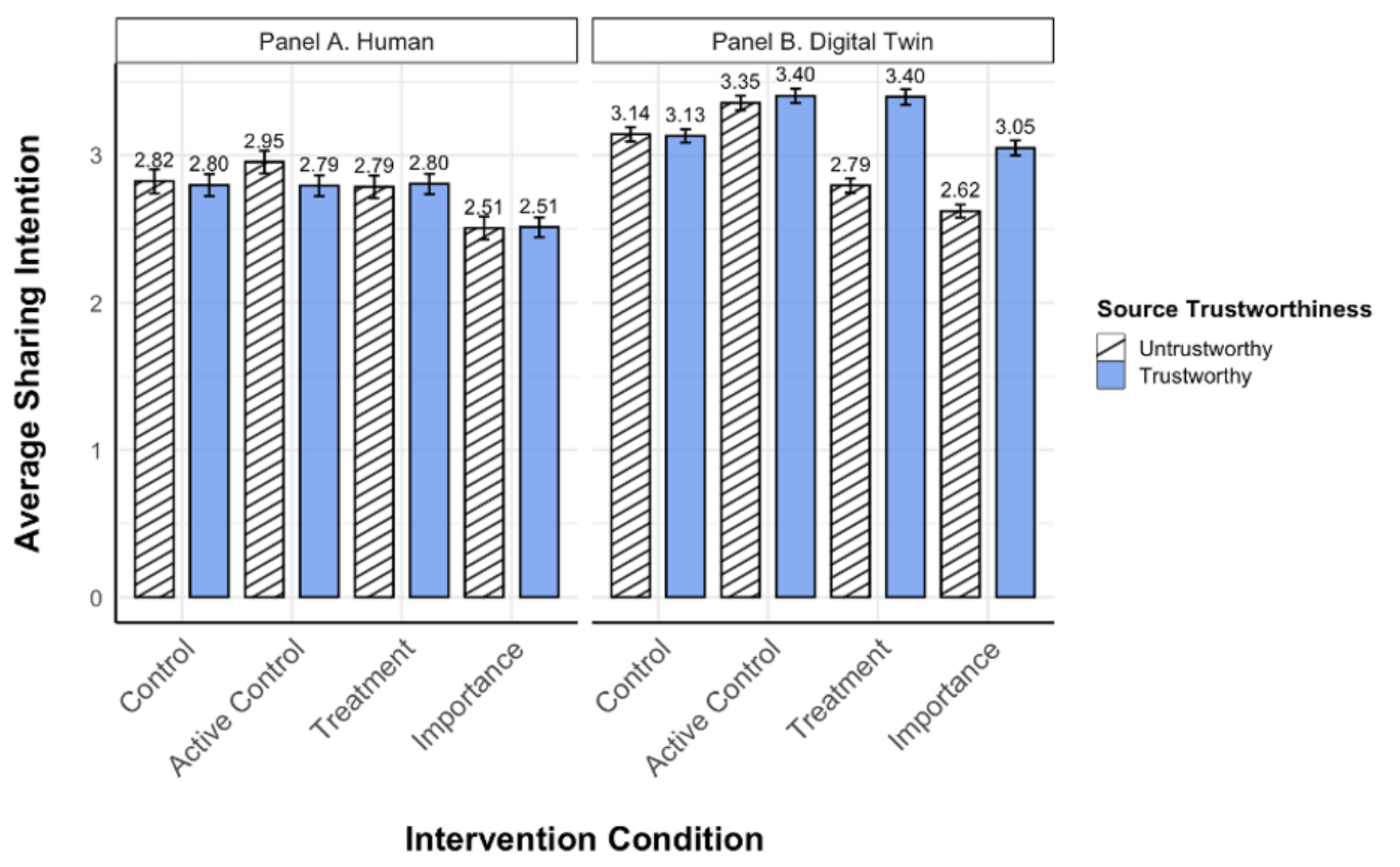

Fig. S15 Sharing Intentions by Headline Veracity and Intervention. NOTE: Error bars indicate 95% confidence intervals based on robust standard errors clustered on participant.

Predictors	Estimate	Std. Error	95% CI	t
(Intercept)	2.82***	0.06	[2.70, 2.95]	44.73

[2]Linear mixed-effects models were estimated using the `lmerTest` package, which provides p-values and F-tests using Satterthwaite's approximation.

Predictors	Estimate	Std. Error	95% CI	t
Participant Type	0.32^{***}	0.07	[0.18, 0.46]	4.38
Headline Veracity	-0.03	0.07	[-0.17, 0.12]	-0.35
Active Control	0.13	0.09	[-0.05, 0.31]	1.46
Treatment	-0.04	0.09	[-0.21, 0.14]	-0.42
Importance Treatment	0.32^{***}	0.09	[0.14, 0.49]	3.56
Participant Type × Headline Veracity	0.01	0.10	[-0.19, 0.22]	0.13
Participant Type × Active Control	0.08	0.10	[-0.12, 0.28]	0.80
Participant Type × Treatment	-0.31^{**}	0.10	[-0.51, -0.11]	-3.00
Participant Type × Importance Treatment	-0.20^{*}	0.10	[-0.41, 0.00]	-1.98
Participant Type × Active Control	-0.14	0.10	[-0.34, 0.07]	-1.31
Participant Type × Treatment	0.05	0.10	[-0.16, 0.25]	0.44
Participant Type × Importance Treatment	0.03	0.10	[-0.17, 0.23]	0.30
Participant Type × Headline Veracity × Active Control	0.20	0.15	[-0.09, 0.48]	1.34
Participant Type × Headline Veracity × Treatment	0.57^{***}	0.15	[0.28, 0.85]	3.88
Participant Type × Headline Veracity × Importance	0.41^{**}	0.15	[0.12, 0.69]	2.81
**Random Effects**				
σ^2	0.67			
τ_{00} (TWIN_ID)	0.33			
ICC	0.33			
N (TWIN_ID)	1003			
Observations	4012			
Marginal R^2 / Conditional R^2	0.073 / 0.383			

Table S4: **Model A: Random Intercepts.** Headline Veracity, Intervention, and Participant Type on Sharing. * $p < 0.05$, ** $p < 0.01$, *** $p < 0.001$.

For humans, accuracy nudges did not improve truth discernment when sharing entertaining news (Figure S15, Panel A). As in [39], there were no significant differences in sharing intentions between the Control and Active Control conditions (Headline Veracity × Intervention: $b = 0.14$, 95% CI $[-0.07, 0.34]$, $t(2997) = 1.31$, $p = .56$). In contrast to the previous findings, n either a ccuracy n udge significantly increased sharing discernment relative to the controls (e.g., Headline Veracity × Intervention: Treatment vs. Control, $b = -0.05$, 95% CI $[-0.25, 0.16]$, $t(2997) = -0.44$,

$p = .97$; Importance Treatment vs. Control, $b = -0.03$, 95% CI $[-0.23, 0.17]$, $t(2997) = -0.30$, $p = .99$). See Table S5 for pairwise comparisons.

Contrast	Estimate	SE	df	t-ratio	p-value	95% CI
Control – Active Control	0.14	0.10	2997	1.31	0.56	[-0.07, 0.34]
Control – Treatment	-0.05	0.10	2997	-0.44	0.97	[-0.25, 0.16]
Control – Importance	-0.03	0.10	2997	-0.30	0.99	[-0.23, 0.17]
Active Control – Treatment	-0.18	0.10	2997	-1.74	0.30	[-0.38, 0.02]
Active Control – Importance	-0.17	0.10	2997	-1.61	0.37	[-0.37, 0.04]
Treatment – Importance	0.01	0.10	2997	0.14	1.00	[-0.19, 0.22]

Table S5: Pairwise comparisons for humans.

For digital twins, however, accuracy nudges successfully improved truth discernment when sharing entertaining news (Figure S15, Panel B). As in [39], there were no significant differences in sharing intentions between the Control and Active Control conditions (Headline Veracity × Intervention: $b = -0.06$, 95% CI $[-0.26, 0.14]$, $t(2997) = -0.58$, $p = .94$), and both treatments significantly increased sharing discernment relative to the controls (e.g., Headline Veracity × Intervention: Treatment vs. Control, $b = -0.61$, 95% CI $[-0.82, -0.41]$, $t(2997) = -5.93$, $p < .001$; Importance Treatment vs. Control, $b = -0.44$, 95% CI $[-0.64, -0.24]$, $t(2997) = -4.27$, $p < .001$). See Table S6 for pairwise comparisons.

Contrast	Estimate	SE	df	t-ratio	p-value	95% CI
Control – Active Control	-0.06	0.10	2997	-0.58	0.94	[-0.26, 0.14]
Control – Treatment	-0.61	0.10	2997	-5.93	$p < .001$	[-0.82, -0.41]
Control – Importance	-0.44	0.10	2997	-4.27	$p < .001$	[-0.64, -0.24]
Active Control – Treatment	-0.55	0.10	2997	-5.33	$p < .001$	[-0.76, -0.35]
Active Control – Importance	-0.38	0.10	2997	-3.67	$p < .001$	[-0.58, -0.18]
Treatment – Importance	0.17	0.10	2997	1.68	0.33	[-0.03, 0.38]

Table S6: Pairwise comparisons for twins.

S10.1.4 Results - Additional Analyses (Non-Preregistered)

The above pre-registered analyses treated participant as a random intercept, capturing individual differences in baseline sharing intentions but assuming that the effects of the predictors were consistent across participants. However, this approach

does not account for the possibility that participants may vary in their responsiveness to the manipulations. To provide a more conservative test, a linear mixed-effects regression was estimated with random intercepts and random slopes for each within-subject predictor. This specification allows the effects of predictors to vary across individuals, capturing heterogeneity in participants' responses. The model fit improved significantly ($\chi^2(2) = 935.23$, $p < .001$), yet the three-way interaction remained significant ($F(3, 1998) = 12.09$, $p < .001$), suggesting that the findings from the pre-registered model are robust to alternative model specifications. See Table S7 for the full regression results.

**Predictors**	**Estimates**	**SE**	**95% CI**	**t**
(Intercept)	2.82***	0.07	[2.68, 2.97]	38.92
Participant Type	0.32***	0.08	[0.16, 0.48]	3.90
Headline Veracity	-0.03	0.05	[-0.12, 0.07]	-0.51
Condition [Active Control]	0.13	0.10	[-0.07, 0.33]	1.27
Treatment	-0.04	0.10	[-0.24, 0.16]	-0.37
Importance	-0.32**	0.10	[-0.52, -0.12]	-3.09
Participant Type × Headline Veracity	0.01	0.07	[-0.13, 0.15]	0.20
Participant Type × Active Control	0.08	0.12	[-0.15, 0.31]	0.71
Participant Type × Treatment	-0.31**	0.12	[-0.54, -0.08]	-2.67
Participant Type × Importance	-0.20	0.12	[-0.43, 0.02]	-1.76
Participant Type × Active Control	-0.14	0.07	[-0.28, 0.00]	-1.90
Participant Type × Treatment	0.05	0.07	[-0.09, 0.19]	0.64
Participant Type × Importance	0.03	0.07	[-0.11, 0.17]	0.43
Participant Type × Headline Veracity × Active Control	0.20	0.10	[-0.00, 0.39]	1.94
Participant Type × Headline Veracity × Treatment	0.57***	0.10	[0.37, 0.77]	5.63
Participant Type × Headline Veracity × Importance	0.41***	0.10	[0.21, 0.61]	4.07
**Random Effects**				
σ^2	0.32			
τ_{00} (TWIN_ID)	1.01			
τ_{11} (TWIN_ID.ParticipantType)	1.05			
ρ_{01} (TWIN_ID)	-0.82			
ICC	0.68			
N (TWIN_ID)	1003			
Observations	4012			
Marginal R^2 / Conditional R^2	0.073 / 0.706			

Predictors	Estimates	SE	95% CI	t

Table S7: Model B: Random Intercepts and Random Slopes. Headline veracity, intervention, and participant type on sharing. ** p<0.05, ** p<0.01, *** p<0.001.*

Additionally, as a robustness check, a linear mixed-effects model with random intercepts and slopes (nested) was estimated to account for the repeated-measures structure of the data, specifically participants' repeated responses to multiple headlines. Similar to the pre-registered model with random intercepts (Model A), this model revealed significant main effects of Participant Type ($F(1, 999) = 97.25$, $p < .001$) as well as a significant effect of Intervention ($F(3, 999) = 17.89$, $p < .001$). Crucially, the results also revealed a significant three-way interaction ($F(3, 999) = 12.76$, $p < .001$), further suggesting that the findings from the pre-registered model are robust to alternative model specifications. See Table S8 for the full regression results.

Predictors	Estimates	SE	95% CI	t
(Intercept)	2.82***	0.17	[2.48, 3.16]	16.28
Participant Type	0.32***	0.08	[0.16, 0.48]	3.84
Headline Veracity	-0.03	0.23	[-0.48, 0.43]	-0.11
Active Control	0.13	0.09	[-0.05, 0.31]	1.41
Treatment	-0.04	0.09	[-0.22, 0.14]	-0.41
Importance Treatment	-0.32***	0.09	[-0.50, -0.14]	-3.45
Participant Type × Headline Veracity	0.01	0.07	[-0.12, 0.15]	0.20
Participant Type × Active Control	0.08	0.12	[-0.15, 0.31]	0.70
Participant Type × Treatment	-0.31**	0.12	[-0.54, -0.08]	-2.63
Participant Type × Importance	-0.20	0.12	[-0.43, 0.03]	-1.74
Headline Veracity × Active Control	-0.14	0.07	[-0.28, 0.00]	-1.90
Headline Veracity × Treatment	0.05	0.07	[-0.09, 0.19]	0.64
Headline Veracity × Importance	0.03	0.07	[-0.11, 0.17]	0.43
Participant Type × Headline Veracity × Active Control	0.20*	0.10	[0.00, 0.39]	1.99
Participant Type × Headline Veracity × Treatment	0.57***	0.10	[0.38, 0.76]	5.78
Participant Type × Headline Veracity × Importance	0.41***	0.10	[0.22, 0.60]	4.18
**Random Effects**				
σ^2	0.97			
τ_{00} (group_id:TWIN_ID)	0.75			
τ_{00} (TWIN_ID)	0.19			

Predictors	Estimates	SE	95% CI	t
τ_{00} (headline_id)	0.21			
τ_{11} (group_id:TWIN_ID.Head.Veracity)	0.36			
τ_{11} (TWIN_ID.Head.Veracity)	0.03			
ρ_{01} (group_id:TWIN_ID)	-0.41			
ρ_{01} (TWIN_ID)	-0.52			
ICC	0.53			
N (group_id)	2006			
N (TWIN_ID)	1003			
N (headline_id)	16			
Observations	32,096			
Marginal R^2 / Conditional R^2	0.037 / 0.546			

Table S8: **Model C: Headline veracity, intervention, and participant type on sharing (nested random effects).** ** p<0.05, ** p<0.01, *** p<0.001.*

As an additional test, [39] analysis plan was next replicated for humans and their digital twins, separately. While the pre-registered analysis demonstrates sharing intentions for the full range of responses, [39] rescale the continuous sharing intentions into a binary variable capturing responses at or above the midpoint at "likely to share" (= 1) and below the midpoint as "unlikely to share" (= 0), and filtered out participants who would not consider sharing these articles on social media. Because the human dataset revealed significant differences between the Control and Active Control conditions, these groups were not pooled to maintain comparability. This results in a slight deviation from the analysis in [39]. Using this framework, their exact analysis was replicated for humans and their digital twins separately, treating the rescaled likelihood to share variable as the DV with Headline Veracity condition, Intervention condition, and their interaction as predictors, applying robust standard errors. Figure S16 shows proportion of sharing across all conditions for humans (Panel A) and their digital twins (Panel B).

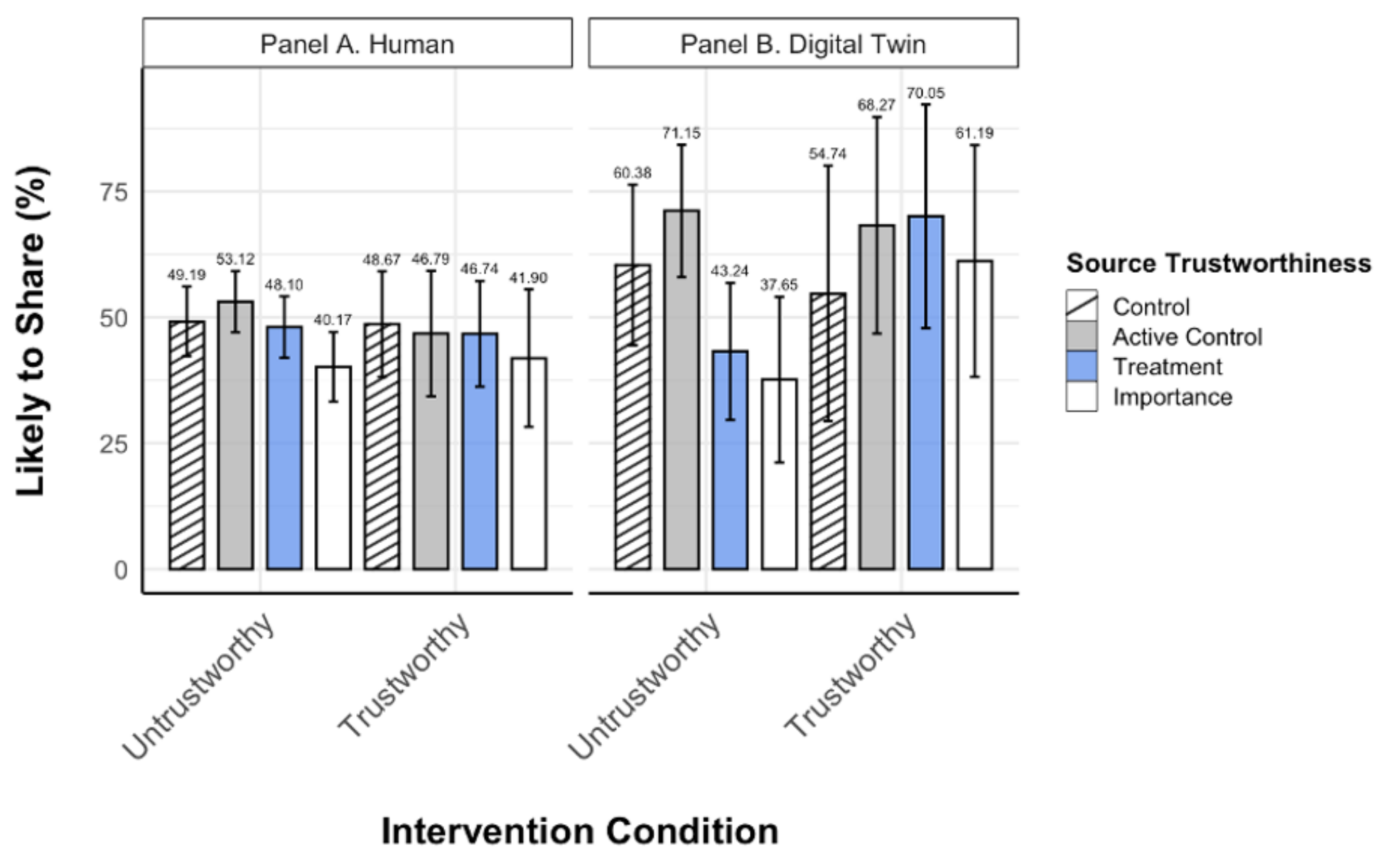

Fig. S16 Proportion (%) Likely to Share by Headline Veracity and Intervention. NOTE: Shown here is the fraction of "likely' responses (responses above the midpoint of the six-point Likert scale) by Headline Veracity and Intervention condition; the full distributions of responses are shown below, in figure S9. As with [39], these analyses focus only on participants who indicated that they would consider sharing this social media content. Analysis including all participants does not change the results. Error bars indicate 95% confidence intervals based on robust standard errors clustered on participant and headline.

Replicating the main findings for humans (Model A), accuracy nudges did not improve truth discernment when sharing entertaining news (Figure S16, Panel A). In contrast to [39], sharing discernment was marginally higher in the Active Control compared to the Control condition (Headline Veracity × Intervention: $b = -0.03$, 95% CI $[-0.07, 0.00]$, $F(1, 5132) = 3.53$, $p = .06$), and neither treatment significantly increased sharing discernment relative to the controls (Headline Veracity × Intervention: Treatment, $b = 0.01$, 95% CI $[-0.05, 0.06]$, $F(1, 9656) = 0.05$, $p = .82$; Importance Treatment, $b = 0.01$, 95% CI $[-0.04, 0.05]$, $F(1, 9656) = 0.06$, $p = .80$). See Table S9 for the full regression results.

Humans: Participants who are willing to share content				
**Model 1: Controls only**				
**Predictors**	**Estimate**	**SE**	**95% CI**	**t**
(Intercept)	0.45***	0.03	[0.39, 0.50]	17.82
Active Control	0.04	0.02	[0.01, 0.09]	1.64
Headline Veracity	-0.01	0.04	[-0.10, 0.08]	-0.26

Humans: Participants who are willing to share content				
**Model 1: Controls only**				
**Predictors**	**Estimate**	**SE**	**95% CI**	**t**
Active Control × Headline Veracity	-0.03	0.02	[-0.07, 0.00]	-1.88
Observations	5,136			
R^2 / Adjusted R^2	0.003 / 0.003			
**Model 2: All conditions**				
**Predictors**	**Estimate**	**SE**	**95% CI**	**t**
(Intercept)	0.45***	0.03	[0.39, 0.50]	17.82
Active Control	0.04	0.02	[-0.01, 0.09]	1.64
Headline Veracity	-0.01	0.04	[-0.10, 0.08]	-0.26
Active Control × Headline Veracity	-0.03	0.02	[-0.07, 0.00]	-1.88
Treatment	-0.01	0.03	[-0.07, 0.06]	-0.22
Importance Treatment	-0.06*	0.03	[-0.12, 0.00]	-2.19
Treatment × Headline Veracity	0.01	0.03	[-0.05, 0.07]	0.23
Importance Treatment × Headline Veracity	0.01	0.02	[-0.04, 0.06]	0.25
Observations	9,664			
R^2 / Adjusted R^2	0.009 / 0.008			

Table S9: Headline Veracity, Intervention and Participant Type on Humans' Sharing Intentions. ** p<0.05, ** p<0.01, *** p<0.001.* When reporting the effects, separate F-tests were conducted; therefore, the reported F statistics are not included in this table, following the reporting approach used by [39].

Again, replicating the main findings for digital twins (Model A), accuracy nudges improved truth discernment when sharing entertaining news (Figure S16, Panel B). As in [39], there were no significant differences in sharing intentions between the Control and Active Control conditions (Headline Veracity × Intervention: $b = 0.02$, 95% CI $[-0.02, 0.06]$, $F(1, 4046) = 1.17$, $p = .28$), and both treatments significantly increased sharing discernment relative to the controls (Headline Veracity × Intervention: Treatment, $b = 0.14$, 95% CI [0.08, 0.20], $F(1, 7208) = 23.30$, $p < .001$; Importance Treatment, $b = 0.12$, 95% CI [0.07, 0.17], $F(1, 7208) = 21.80$, $p < .001$). See Table S10 for the full regression results.

Twins: Participants who are willing to share content				
**Model 3: Controls only**				
**Predictors**	**Estimate**	**SE**	**CI**	**t**
(Intercept)	0.53***	0.03	[0.46, 0.60]	15.97
Active Control	0.04**	0.01	[0.01, 0.07]	3.05
Headline Veracity	-0.01	0.08	[-0.18, 0.15]	-0.20
Active Control × Headline Veracity	0.02	0.02	[0.02, 0.07]	1.08
Observations	4,064			
R^2 / Adjusted R^2	0.018 / 0.017			
**Model 4: All conditions**				
**Predictors**	**Estimate**	**SE**	**CI**	**t**
(Intercept)	0.53***	0.03	[0.46, 0.60]	15.97
Active Control	0.04**	0.01	[0.01, 0.07]	3.05
Headline Veracity	-0.01	0.08	[-0.18, 0.15]	-0.20
Active Control × Headline Veracity	0.02	0.02	[0.02, 0.07]	1.08
Treatment	-0.07*	0.03	[-0.13, -0.02]	-2.81
Importance Treatment	0.10**	0.02	[0.05, 0.15]	-4.07
Condition × Headline Veracity	0.14***	0.03	[0.08, 0.20]	4.83
Importance Treatment × Headline Veracity	0.12***	0.03	[0.06, 0.17]	4.67
Observations	7,216			
R^2 / Adjusted R^2	0.058 / 0.057			

Table S10: **Headline Veracity, Intervention and Participant Type on Twins' Sharing Intentions.** ** p<0.05, ** p<0.01, *** p<0.001.* When reporting the effects, separate F-tests were conducted; therefore, the reported F statistics are not included in this table, following the reporting approach used by [39].

Finally, Figure S17 displays the full distribution of individual sharing likelihood ratings for each participant type across all intervention conditions and headline veracity levels. These distributions reveal that humans were far more likely to report that they would not share headlines, with many responses clustering at the low end of the scale. In contrast, digital twins tended to give more moderate responses, with sharing likelihood ratings concentrated around the midpoint and a greater proportion of "likely to share" responses overall. This pattern suggests that, unlike humans—who default to inaction—digital twins are generally more inclined to share content.

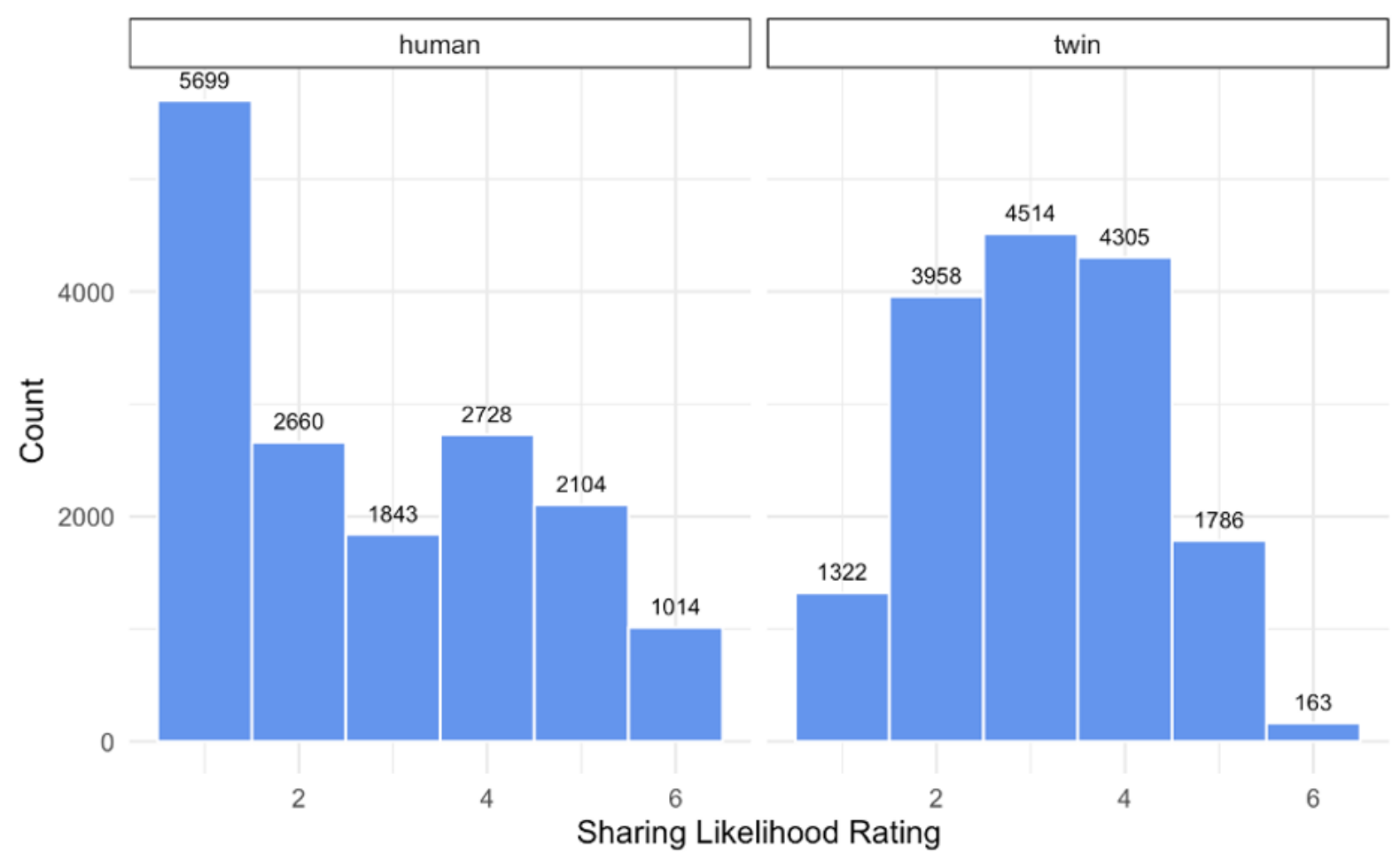

Fig. S17 Full Distribution of Individual Sharing Likelihood Ratings.

These additional analyses strengthen confidence in the findings and provide a straightforward comparison for humans and their digital twins, separately, with the reported findings from [39].

S10.1.5 Discussion

These findings extend prior research studying the effectiveness of accuracy nudges in combating the spread of misinformation [39]. Critically, accuracy nudges may have limited effectiveness for stopping the spread of false entertainment news. While prior research has shown that nudges can redirect attention toward accuracy and reduce the spread of false information in political contexts [39], this finding does not seem to generalize to more playful, emotionally engaging content. Specifically, even when primed to consider accuracy or reminded of its importance, humans showed no statistically reliable reduction in their willingness to share false versus true entertainment headlines. Even more, when first prompted to rate the entertainingness of unrelated headlines (i.e., Active Control condition), participants were marginally more likely to share false versus true entertainment headlines, indicating worse discernment. Interestingly, in contrast to humans, digital twins responded as the prior work would predict, demonstrating robust improvements in truth discernment in both the Treatment and Importance Treatment conditions.

One possible reason for this divergence is that digital twins, unlike humans, do not experience the hedonic appeal of entertaining content. For humans, entertainment may override epistemic goals, especially when sharing functions as a form of social play or bonding. In contrast, digital twins may more rigidly apply the prioritization embedded in their programming or training data, defaulting to accuracy in the absence of competing motivations. In this way, digital twins act as idealized rational agents: models of how people ought to behave if their sole goal was to share accurate information. In the same vein, twins seem more prone to prioritizing "should" or socially

desirable characteristics (e.g., source trustworthiness) over truly social characteristics (e.g., entertainingness). But humans operate in a messier reality, where attention is easily hijacked by humor, surprise, or emotional resonance. This gap underscores a broader challenge in misinformation interventions: even the most well-intentioned nudges may fall short when the content itself draws attention away from accuracy.

From a practical standpoint, the results highlight the limits of one-size-fits-all interventions. If humans are less responsive to accuracy nudges in hedonic contexts, future interventions may need to do more than redirect attention—they may need to reshape the perceived function of sharing itself. For example, interventions could incentivize or socially reward accurate sharing, rebalancing the tradeoff between entertainment and truth. More ambitiously, platforms might redesign their interfaces to elevate epistemic cues without dampening the joy of social engagement. At a theoretical level, these findings invite deeper reflection on how models of decision-making should incorporate context-dependent goals, and how digital twins can help surface not just what people do, but why they do it.

S10.2 Affective Primes

S10.2.1 Main Questions/Hypotheses

Research in the social sciences often manipulates emotional states using affective priming, where participants are asked to reflect on a recent event. In this study, we test whether affective priming works with digital twins. Specifically, we ask:

1. Do affective priming manipulations induce "states" in digital twins (e.g., does writing about gratitude or lack of control momentarily influence digital twins' responses?)
2. Is the influence of affective priming similar for digital twins and their human counterparts?
3. Is the influence of affective priming on digital twins dependent on the valence of affective prime (i.e., between positive primes like gratitude or negative primes like lack of control) or the proximal nature of the dependent measures (i.e., does it "spill over" to other, related dimensions or only influence proximal dimensions)?

S10.2.2 Methods

One thousand human participants on Prolific (and their twins) participated in the study. They were randomly assigned the same condition in a 2 (affective domain: gratitude vs. lack of control) x 2 (prompt: prime vs. baseline) between-subjects design.

Participants first responded to a condition-specific prompt for two minutes. For the gratitude domain, participants received either a gratitude prime ("Please recall carefully and in detail a specific experience in the past when you felt sincerely grateful for someone's kindness or help") or baseline prompt ("Please recall carefully and in detail the sequence of your morning routine, such as brushing teeth or changing clothes"), using procedures from [46–48]. In the lack of control domain, participants received either the lack of control prime ("Please recall a particular incident in which something happened and you did not have control over the situation. Please describe the situation in which you felt a lack of control – what happened, how you felt, etc.") or a baseline prompt ("Please recall a particular incident in which something happened and you were in control of the situation. Please describe the situation in which you felt in control – what happened, how you felt, etc."), using procedures from [49–52].

After the writing task, participants completed a manipulation check, followed by proximal dependent measures (closely related to the target affective state) and distal dependent measures (more distantly related to the affective state). For the gratitude domain, the manipulation check assessed gratitude-related emotions (grateful, thankful, appreciative) on a 7-point scale from "not at all" to "extremely." The proximal measure used the Elevation scale [53, 54], which asked participants to rate their current feelings on six items such as "Feeling optimistic about humanity" using the same 7-point scale. The distal measure employed an adapted version of the Empathic Concern scale [55], measuring agreement with two statements, "I am very concerned about those most vulnerable to the effects of tariffs" and "I feel compassion for those most affected by rising prices or job losses due to tariffs," on a 7-point scale from "completely disagree" to "completely agree."

For the lack of control domain, the manipulation check used items from [56], asking participants to rate their agreement with three statements ("I feel in control of my life", "I am free to live my life how I wish", "My experiences in life are due to my own actions", on a 7-point scale from "not at all" to "extremely." These items were reverse coded for the analysis, but we present the raw means before reverse coding in the graph. The proximal measure was the Fatigue scale [49, 57], consisting of nine items such as "I had a hard time thinking about the event" rated on the same 7-point scale. The distal measure employed the Desire for Predictability scale [51, 58], containing eight items like "would not like to go into a situation without knowing what I can expect from it" rated on a 7-point scale from "completely disagree" to "completely agree."

S10.2.3 Results - Pre-registered Analyses

First, the scales demonstrated strong internal consistency for both humans and digital twins. For Digital Twins: gratitude manipulation check ($\alpha = .998$), elevation scale ($\alpha = .964$), empathic concern measure ($\alpha = .992$), lack of control manipulation check ($\alpha = .951$), fatigue scale ($\alpha = .897$), and desire for predictability measure ($\alpha = .931$). For Humans: gratitude manipulation check ($\alpha = .979$), elevation scale ($\alpha = .917$), empathic concern measure ($\alpha = .909$), lack of control manipulation check ($\alpha = .830$), fatigue scale ($\alpha = .766$), and desire for predictability measure ($\alpha = .866$). As a result, we created a composite variable for each measure by averaging the respective items.

Following our pre-registered analysis plan, we then averaged the outcome measures in the gratitude domain (elevation, empathic concern; $r = .38$) to create an aggregate downstream dependent variable for gratitude, and averaged the outcome measures in the lack of control domain (fatigue, desire for predictability; $r = .09$) to create an aggregate downstream dependent variable for lack of control. We also examined effects by individual measure.

Manipulation Check

To examine the effect of affective priming on humans versus digital twins for both gratitude and lack of control, we ran a linear mixed-effects regression using the `lmerTest` package in R (Kuznetsova, Brockhoff, & Christensen, 2017) with the manipulation check measure as the dependent variable; affective domain (gratitude vs. feeling in control), prompt (prime vs. baseline), twin (human vs. digital), and their interactions as independent variables; and twin identifier as a random intercept. We did not observe a significant three-way interaction ($b = -0.17$, $SE = 0.17$, $t(996) = -1.01$, $p = .314$), but several interesting two-way interactions emerged, which we detail below.

First, affective priming manipulations successfully induced "states" in both digital twins and humans. The prompt (prime vs. baseline) had a significant effect on manipulation checks for digital twins in both the gratitude domain ($M_{\text{baseline}} = 4.34$, $SD = 1.17$; $M_{\text{prime}} = 5.67$, $SD = 0.71$; $b = 1.33$, $SE = 0.10$; $t(1760) = 12.93$, $p < .001$) and the lack of control domain ($M_{\text{baseline}} = 2.79$, $SD = 0.86$; $M_{\text{prime}} = 3.79$, $SD = 0.95$; $b = 1.00$, $SE = 0.10$; $t(1760) = 9.55$, $p < .001$), as well as for humans in the gratitude domain ($M_{\text{baseline}} = 5.19$, $SD = 1.56$; $M_{\text{prime}} = 6.00$, $SD = 1.33$; $b = 0.81$, $SE = 0.10$; $t(1760) = 7.91$; $p < .001$) and the lack of control domain

($M_{\text{baseline}} = 2.81$, $SD = 1.16$; $M_{\text{prime}} = 3.12$, $SD = 1.29$; $b = 0.32$, $SE = 0.10$; $t(1760) = 3.02$, $p = .003$).

Second, the effect of priming was significantly stronger for digital twins than for humans in both affective domains. Digital twins showed greater responsiveness to the prompt (prime vs. baseline) in both the gratitude domain (interaction: $b = -0.52$, $SE = 0.12$; $t(996) = -4.45$, $p < .001$) and the lack of control domain (interaction: $b = -0.68$, $SE = 0.12$; $t(996) = -5.79$, $p < .001$), compared to humans (see Figure S18).

Finally, the prompt (prime vs. baseline) produced stronger effects in the gratitude domain than in the lack of control domain for both digital twins ($b = 0.33$, $SE = 0.15$; $t(1760) = 2.26$, $p = .024$) and humans ($b = 0.50$, $SE = 0.15$; $t(1760) = 3.39$, $p < .001$).

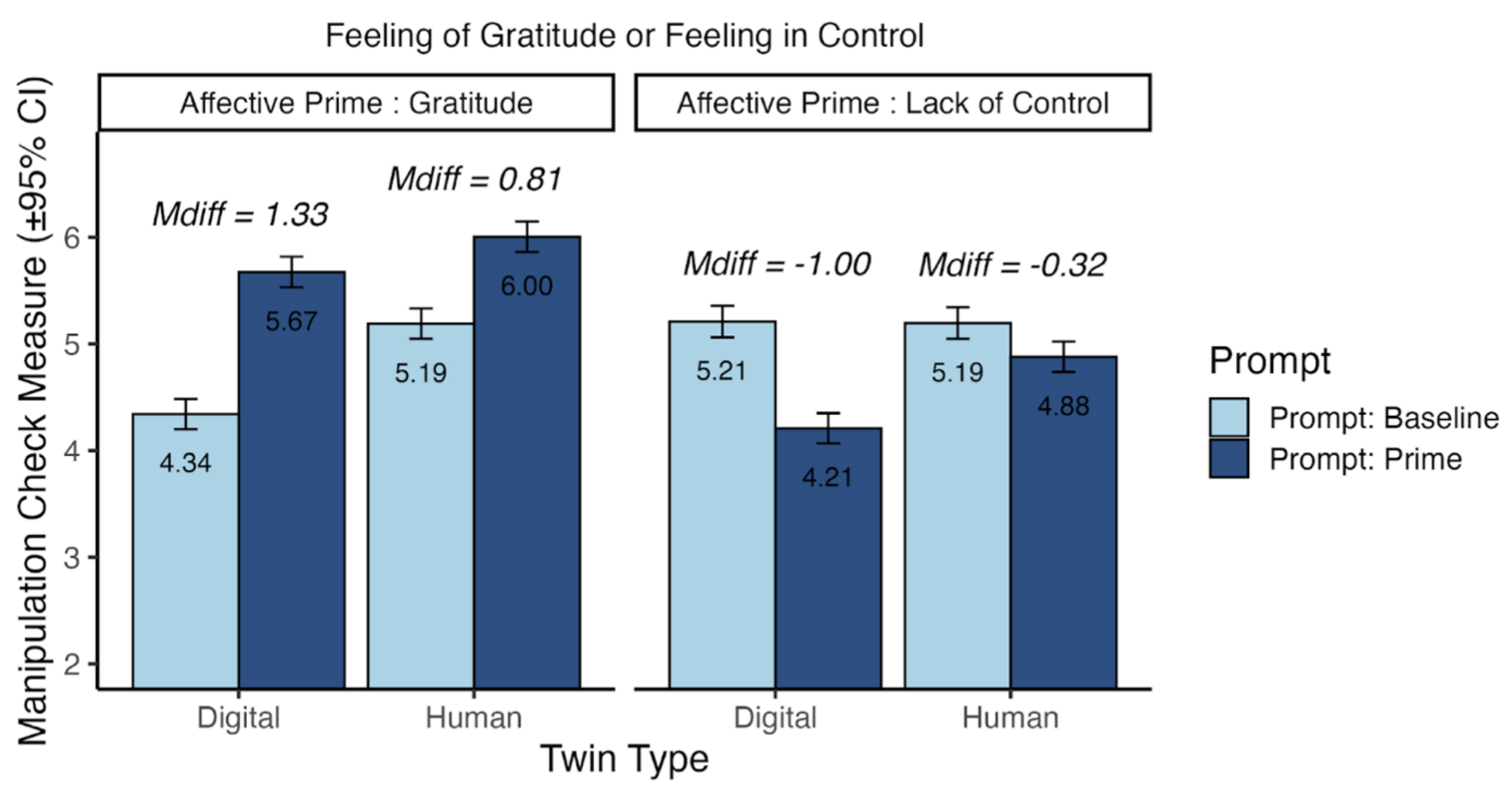

Fig. S18 Effect of Affective Prime on Manipulation Check.

Dependent Measures

We next examined how affective priming in digital twins affects downstream dependent measures—that is, do these "affective states" spill over onto other measures? To test this, we ran a linear mixed-effects regression with the aggregate dependent variable measure as the dependent variable; affective domain (gratitude vs. lack of control), prompt (prime vs. baseline), twin (human vs. digital), and their interaction as independent variables; and twin identifier as a random intercept. We did not observe a significant three-way interaction ($b = -0.13$, $SE = 0.11$, $t(996) = -1.17$, $p = .242$), but two significant two-way interactions emerged, which we detail below.

First, affective priming manipulations spilled over onto downstream dependent measures for both digital twins and humans. The prompt (prime vs. baseline) had a significant effect on downstream measures for digital twins in both the gratitude domain ($M_{\text{baseline}} = 4.39$, $SD = 1.11$; $M_{\text{prime}} = 4.90$, $SD = 1.05$; $b = 0.50$, $SE = 0.08$;

$t(1538) = 6.01$; $p < .001$) and the lack of control domain ($M_{\text{baseline}} = 3.92$, $SD = 0.60$; $M_{\text{prime}} = 4.38$, $SD = 0.68$; $b = 0.46$, $SE = 0.09$; $t(1538) = 5.45$; $p < .001$), as well as for humans in the gratitude domain ($M_{\text{baseline}} = 4.82$, $SD = 1.15$; $M_{\text{prime}} = 5.21$, $SD = 1.23$; $b = 0.81$, $SE = 0.10$; $t(1760) = 7.91$; $p < .001$) and the lack of control domain ($M_{\text{baseline}} = 3.83$, $SD = 0.76$; $M_{\text{prime}} = 4.05$, $SD = 0.69$; $b = 0.32$, $SE = 0.10$; $t(1760) = 3.02$; $p = .003$). See Figure S19.

Second, the effect of priming on downstream dependent measures was significantly stronger for digital twins than humans for lack of control priming ($b = -0.25$, $SE = 0.08$; $t(996) = -3.05$; $p = .002$), but not for gratitude ($b = -0.11$, $SE = 0.08$; $t(996) = -1.43$; $p = .153$).

Finally, the gratitude prime did not produce stronger effects than the lack of control prime for digital twins ($b = 0.04$, $SE = 0.12$; $t(1538) = 0.33$; $p = .744$), but gratitude produced stronger effects for humans ($b = 0.50$, $SE = 0.15$; $t(1760) = 3.39$; $p < .001$).

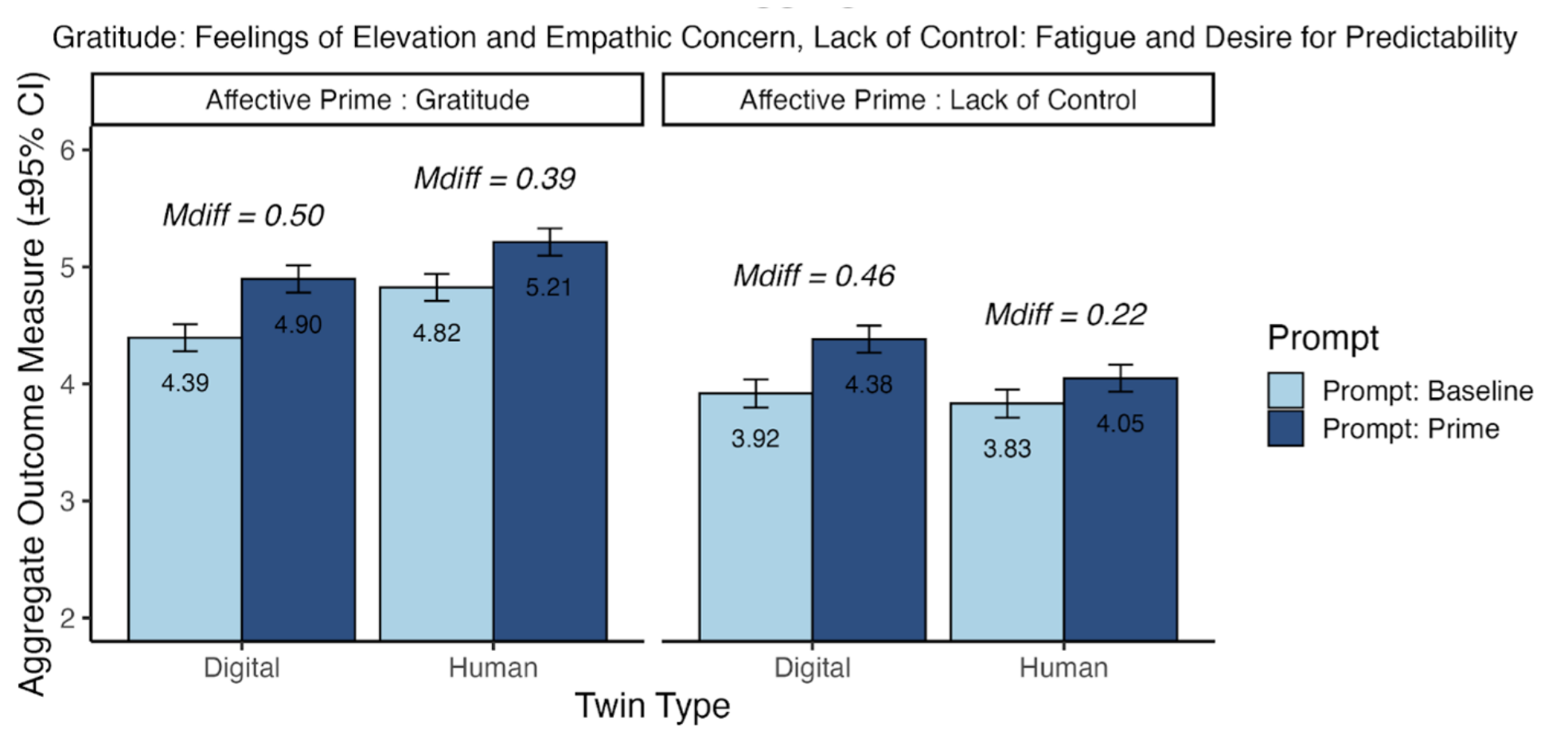

Fig. S19 Effect of Affective Prime on Aggregate Downstream Outcomes.

Individual Dependent Measures

We also pre-registered analyses examining each dependent measure independently. Thus, we ran four linear mixed-effects regressions with (1) elevation, (2) empathic concern, (3) fatigue, and (4) desire for predictability as the dependent variable; condition (manipulation vs. control), twin (human vs. digital), and their interaction as independent variables; and twin identifier as a random intercept. Each measure's simple effect for prompt (prime vs. baseline) was significant for both humans and digital twins, except empathic concern, which was marginal for twins ($M_{\text{baseline}} = 5.04$, $SD = 1.48$; $M_{\text{prime}} = 5.28$, $SD = 1.40$; $b = 0.24$, $SE = 0.14$; $t(731) = 1.74$, $p = .082$) and not significant for humans ($M_{\text{baseline}} = 5.45$, $SD = 1.61$; $M_{\text{prime}} = 5.44$, $SD = 1.64$; $b = -0.01$, $SE = 0.14$; $t(731) = -0.07$, $p = .947$), as well as desire for predictability, which was not significant for twins ($M_{\text{baseline}} = 5.54$, $SD = 1.04$; $M_{\text{prime}} = 5.59$, $SD = 1.04$; $b = 0.05$, $SE = 0.09$; $t(718) = 0.47$; $p = .636$).

The effect of prompt (prime vs. baseline) on elevation did not differ between digital twins and humans ($b = 0.02$, $SE = 0.20$; $t(506) = 0.16$, $p = .872$). The effect of prompt (prime vs. baseline) was stronger for humans than digital twins for empathic concern ($b = 0.25$, $SE = 0.12$; $t(506) = 2.08$; $p = .039$) and fatigue ($b = 0.70$, $SE = 0.09$; $t(490) = 8.26$; $p < .001$). The effect of prompt on desire for predictability was also stronger for humans than for digital twins ($b = 0.21$, $SE = 0.08$; $t(490) = 2.45$; $p = .015$). See Figure S20.

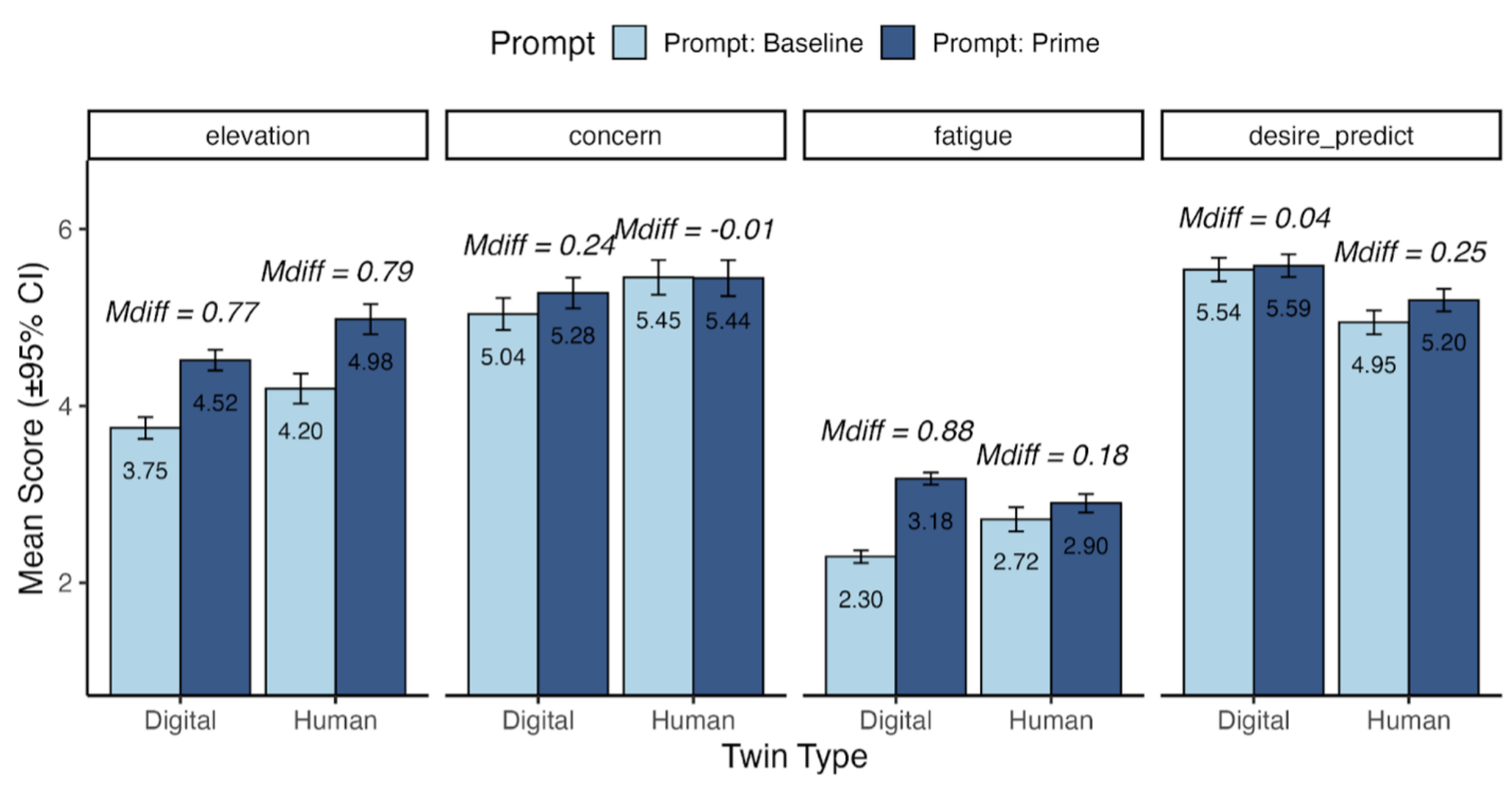

Fig. S20 Measures by Prompt (Prime vs. Baseline) Grouped by Measure and Twin Type.

S10.2.4 Results - Additional Analyses (Non-Preregistered)

Here, we examined the language used when responding to the prompt. Specifically, we examined the language similarity between a human participant's response and their twin versus a human participant's response and a randomly selected twin using the BERT metric, defined as "the sum of cosine similarities between their tokens' embeddings" [59]. In a random-effects regression with language matching as the dependent variable; same-twin versus different-twin pairing as the independent variable; condition as a covariate; and human participant identifier as a random effect, we found that the language between a human participant and their twin was slightly more similar ($M = 0.11$, $SD = 0.09$) than between a human participant and a random twin ($M = 0.13$, $SD = 0.10$; $b = 0.014$, $SE = 0.002$; $t(999) = 5.54$; $p < .001$). Figure S21 shows this by condition.

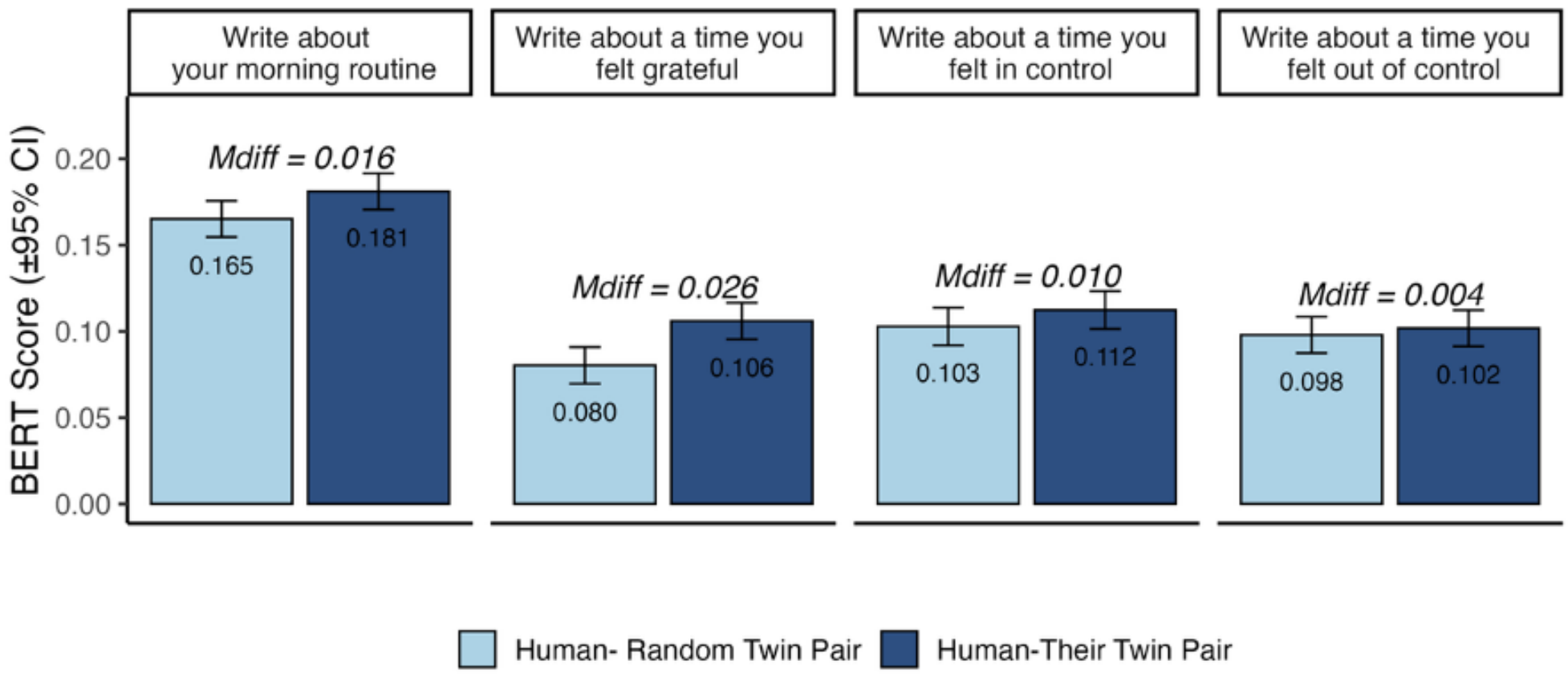

Fig. S21 Similarity Between Human Prompt Text and Their Digital Twin's Prompt Text vs. a Random Digital Twin's Prompt Text.

S10.2.5 Discussion

Returning to each research question:

1. Do affective priming manipulations induce "states" in digital twins (e.g., does writing about gratitude or lack of control momentarily influence digital twins' responses?)

Yes. Writing about feeling grateful or out of control significantly increased corresponding feelings of gratitude and lack of control in digital twins, with these induced "affective states" subsequently influencing downstream outcomes.

2. Is the influence of affective priming similar for digital twins and their human counterparts?

No. Affective priming had stronger effects on digital twins' affective responses compared to humans for both gratitude and lack of control manipulations.

3. Is the influence of affective priming on digital twins dependent on the valence of affective prime (i.e., between positive primes like gratitude or negative primes like lack of control) or the proximal nature of the dependent measures (i.e., does it "spill over" to other, related dimensions or only influence proximal dimensions)?

Valence. Yes, valence affects twins' responses, but in a similar way to humans: The gratitude prime had a stronger effect on the manipulation check than the lack of control prime for both humans and their twins.

Proximal Nature of DVs. Yes, the influence of affective priming on digital twins is dependent on how closely related the dependent measure is to the manipulation. Specifically, the impact of affective prime on proximal dependent measures was consistent and as expected for both twins and humans, but impact of affective prime on distal downstream measures was inconsistent between twins and humans.

For proximal dependent measures (measures closely linked to the manipulation), we observe a "spillover" effect of prime in both the gratitude prime and lack of control prime for humans and twins. For the distal downstream measure (more distantly related to the manipulation) in the gratitude condition, the impact of affective prime was marginal for twins and non-significant for humans. For the distal downstream measure in the lack of control condition, the impact of affective prime was non-significant for twins and significant for humans.

4. Is there a relationship between what digital twins and their human counterparts say in their response to the manipulation?

Yes. The language between a human participant and their twin is slightly more similar than between a human participant and a random twin.

S10.3 Consumer Minimalism

S10.3.1 Main Questions/Hypotheses

We explored the predictive validity of the minimalism scale in a sample of human respondents versus their digital twins. As in the original paper on consumer minimalism [60], we expect that human respondents and their digital twins who score high on the Minimalist Consumer Scale will prefer minimalist home environments over non-minimalist interiors.

S10.3.2 Methods

We recruited 200 human respondents on Prolific and their respective twins. In random order, participants completed the 12 randomized items of the Minimalist Consumer Scale ($M_{\text{human}} = 4.84$, $SD_{\text{human}} = 1.17$, $\alpha_{\text{human}} = .93$; $M_{\text{twin}} = 4.52$, $SD_{\text{twin}} = 1.47$, $\alpha_{\text{twin}} = .94$), and indicated their preference for four sets of minimalist versus non-minimalist interiors, presented in random order. All stimuli were pretested and validated in Study 7e of the *Consumer Minimalism* paper [60]).

In the first two sets, human participants looked at two pairs of images (Figure S22) and indicated whether they would rather live in one of two apartments ("Which apartment would you rather live in?") and whether they found one of two wardrobes more appealing ("Which wardrobe is more appealing to you?"). In the third set, they read the following descriptions of two bedrooms:

Here are the descriptions of two bedrooms.

> First option: A vibrant bedroom filled with colorful patterns, featuring a mix of floral and botanical prints on the bedding and wallpaper. A variety of throw pillows, unique lamps, and framed artwork create a bold, eclectic, and lively atmosphere.
>
> Second option: A minimalist bedroom has a light wood platform bed with white bedding, two pillows, and a neatly folded gray blanket. On one side is a small round table with a plant, and on the other is a clear bedside unit holding a few essentials.
>
> Which bedroom do you like more?

Finally, respondents read the following descriptions of two home offices for the fourth set:

> Here are the descriptions of two home offices
>
> First option: A minimalist and modern home office featuring a floating wooden desk, a sleek grey swivel chair, and a clean setup with a desktop computer and lamp. Built-in shelves with books and decor, along with floor-to-ceiling neutral curtains, create a clean atmosphere.
>
> Second option: A cozy and eclectic home office combines functionality with vibrant personality, featuring a metal-framed desk, a patterned chair, and layered storage options including woven baskets and open shelving. Colorful textiles, books, and decorative accents add warmth and charm.
>
> Which home office would you prefer to be yours?"

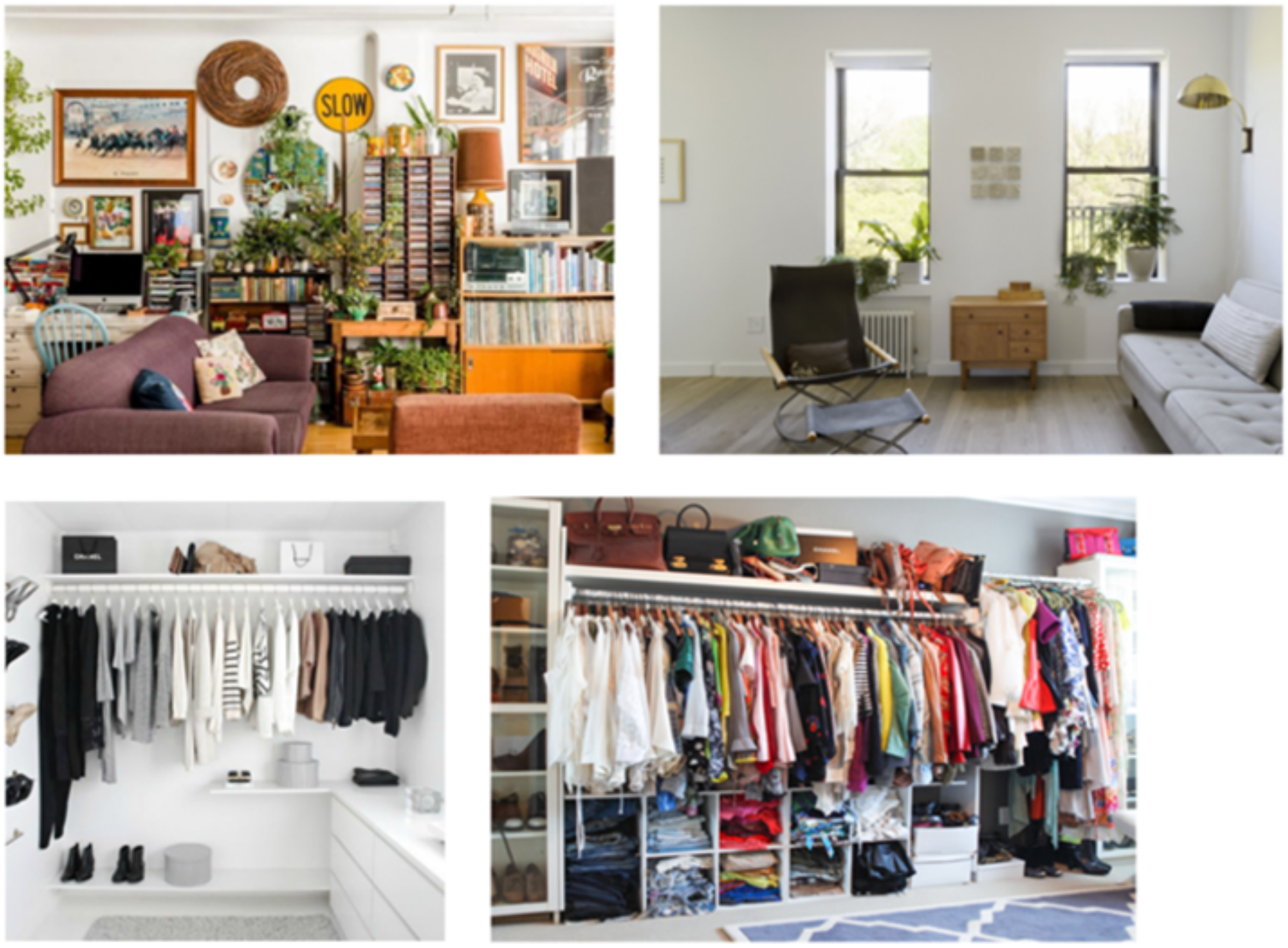

Fig. S22 Visual Stimuli for Human Respondents. Photos from Wilson, AV & Bellezza S, Consumer minimalism, *Journal of Consumer Research*, vol. 48, issue 5, 796-816, by permission of Oxford University Press.

All preferences were recorded on a seven-point Likert scale (1 = definitely the one on the left, 4 = equal preference, 7 = definitely the one on the right). Before the analyses, we coded all response scales so that higher values indicate stronger preferences for minimalist options. Importantly, the digital twins read an AI-generated description of the visual stimuli displayed for human respondents. This intentional feature of the study design enables us to test whether the predictive validity of the scale holds even when twins read descriptions while their human counterparts see images.

For the two apartments' images, digital twins read:

> Below, are pictures of rooms in two different apartments. Which apartment would you rather live in? Left Side: The room contains a large number of objects and furnishings. There are two upholstered armchairs (one dark purple, one reddish-brown) and a small ottoman. Behind the seating area is a wooden desk with a computer monitor and keyboard, along with a painted wooden chair. Shelving units and surfaces are densely populated with plants, CDs, books, and various small objects. Numerous framed pictures, posters, and a "SLOW" traffic sign cover the wall. There is a table lamp with a brown shade and a stereo system beneath a collection of compact discs. Additional furniture includes a small bookshelf filled with vinyl records and a mirror partially obscured by plants. Several types

of plants are placed throughout the room in pots and containers. Right Side: The room contains fewer items and furnishings. There are two windows with black frames and visible trees outside. A light-gray upholstered sofa sits on the right side of the room. A black leather and wood lounge chair is positioned in the center. A small wooden cabinet with a box on top is located between the two windows. Wall-mounted decorative tiles or panels are arranged in a square above the cabinet. Plants are placed on the windowsills and hanging from planters. A wall-mounted reading lamp is affixed above the sofa. The floor is light-colored wood.

For the two wardrobes' images, digital twins read:

Below, are pictures of two wardrobes. Which wardrobe is more appealing to you? Left Side: Contains approximately 20 garments hanging on a single rod, organized by color from dark to light. All garments are on matching white hangers. Colors are limited to black, white, gray, beige, and similar tones. Shoes are placed on two open shelves: one at floor level and one elevated. There are three visible boxes or containers: two cylindrical and one rectangular. The closet space includes built-in white drawers and shelves. A small rug is placed on the floor. Handbags and boxes are stored on a top shelf above the hanging clothes. All visible surfaces, including the walls, shelves, and drawers, are white. Right Side: Contains a larger number of garments (approximately 70+) on a double-length rod. Garments vary in color, pattern, and length. Hangers are mostly dark, with some white or wooden hangers mixed in. Items are densely packed along the hanging rod. Below the clothing rod are multiple open cubbies filled with folded clothes, shoes, bags, and miscellaneous items. The top shelf holds several handbags and boxes. A patterned rug is partially visible on the floor. The shelving units are white, and the wall behind the closet is gray. There is a glass-front cabinet on the left side containing additional shoes or items.

Across sets, we varied whether the minimalist or non-minimalist images appeared on the left or the right of the screen and whether the minimalist or non-minimalist description appeared first or second.

S10.3.3 Results - Pre-registered Analyses

We ran a series of OLS regressions with participants' average score on the Minimalist Consumer Scale as the independent variable and preferences for each option as the dependent variable. As predicted, results indicated that higher scores on the scale were associated with stronger preferences for the minimalist option in each set and in both samples.

More specifically, higher scores on the Minimalist Consumer Scale predicted stronger preferences

1. for the minimalist apartment in both the human ($\beta = .56$, $t(198) = 9.56$, $p < .001$) and the digital ($\beta = .60$, $t(198) = 10.54$, $p < .001$) samples;
2. for the minimalist wardrobe in both the human ($\beta = .44$, $t(198) = 6.85$, $p < .001$) and the digital ($\beta = .65$, $t(198) = 11.92$, $p < .001$) samples;
3. for the minimalist bedroom in both the human ($\beta = .46$, $t(198) = 7.36$, $p < .001$) and the digital ($\beta = .57$, $t(198) = 9.79$, $p < .001$) samples; and
4. for the minimalist home office in both the human ($\beta = .44$, $t(198) = 6.80$, $p < .001$) and the digital ($\beta = .64$, $t(198) = 11.55$, $p < .001$) samples (see Figure S23).

There was no significant effect of order, nor interaction between scores on the Minimalist Consumer Scale and whether participants completed the scale before or after indicating their preferences for the different sets. Moreover, the design feature for the first two sets—with images for humans and AI-generated descriptions for the digital sample—did not yield differences in the results. A regression with interiors preferences as dependent variable, and minimalism (z-scores), whether the stimuli appeared as images or text (coded as 1 and -1, respectively), and their interaction as predictors, while controlling for human respondent vs. digital twin, did not reveal a significant interaction ($b_{\text{interaction}} = -0.13$, $p = .249$).

In other words, the fact that humans saw images and digital twins read descriptions for the first two sets of interiors did not interfere with the overall results.

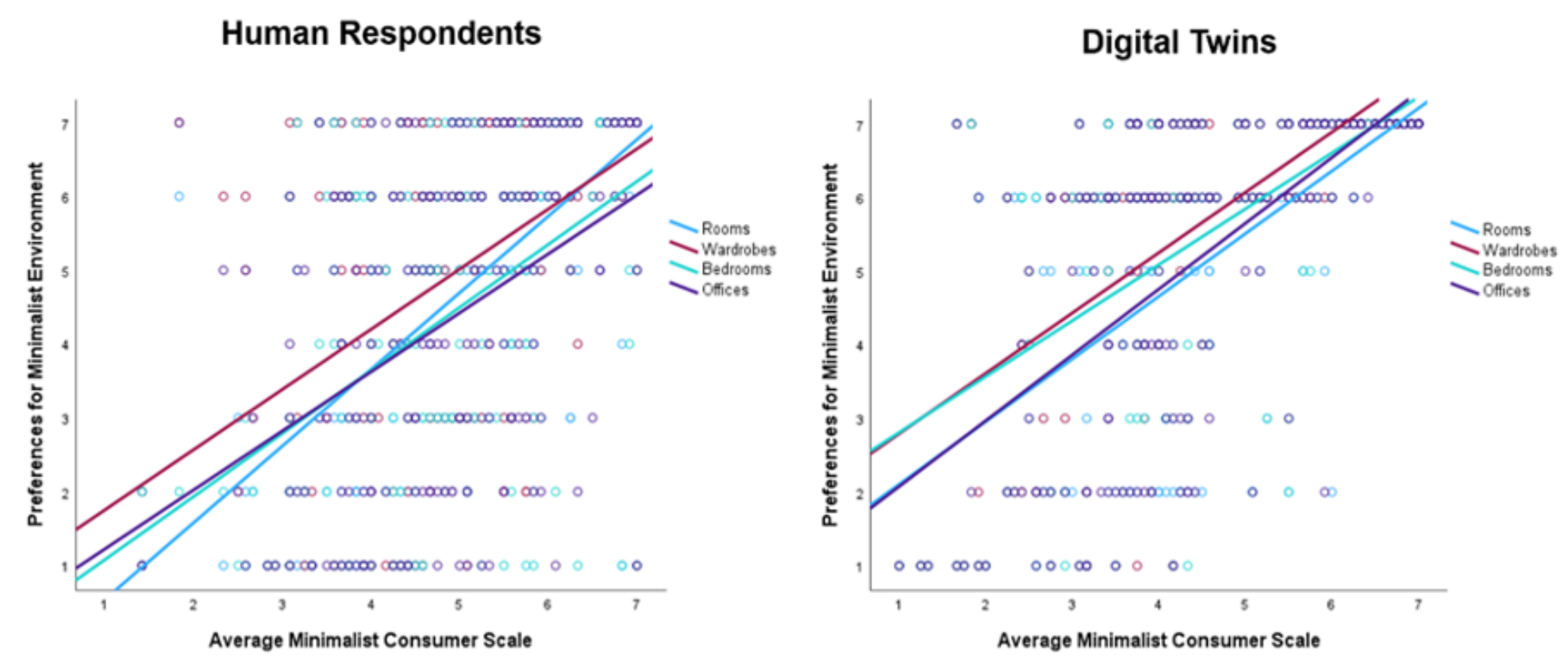

Fig. S23 Results. Note: the dots indicate the raw data.

A comparison of the standardized regression coefficients suggests that the results were slightly stronger in the digital sample than in the human population. A series of t-tests comparing the preference for the minimalist environment for the humans and their twin counterparts suggests that, on average, the twins liked the minimalist environments more (apartment: $M_{\text{human}} = 4.53$ vs. $M_{\text{twin}} = 5.11$, $t(398) = 2.69$, $p = .007$; wardrobe: $M_{\text{human}} = 4.89$ vs. $M_{\text{twin}} = 5.68$, $t(398) = 3.91$, $p < .001$; bedroom: $M_{\text{human}} = 4.36$ vs. $M_{\text{twin}} = 5.49$, $t(398) = 5.45$, $p < .001$; home office: $M_{\text{human}} = 4.30$ vs. $M_{\text{twin}} = 5.23$, $t(398) = 4.39$, $p < .001$).

We also examined the test–retest reliability of the Minimalist Consumer Scale in the human sample between collection rounds, given that the scale was collected twice (approximately four and a half months apart): once as part of a large battery of measures to generate the digital twins (February 1, 2025) and once as part of this specific validity study on the Minimalist Consumer Scale (June 12, 2025). The correlation of the Minimalist Consumer Scale between the two collection rounds was large ($r = .75$, $p < .001$), confirming that the scale has high test–retest reliability (Peter, 1979).

A mixed-effects generalized linear model with random intercepts for participants, with preference for the minimalist interiors as dependent variable, and the Minimalist Consumer Scale, an indicator for data type (coded as 1 for human and 2 for digital twin), and their interaction as predictors, revealed an effect of minimalism ($\beta = .73$, $t(1{,}593) = 11.69$, $p < .001$), an effect for digital twins ($\beta = .71$, $t(1{,}593) = 2.36$, $p = .019$), and no significant interaction ($\beta = .08$, $t(1{,}593) = 1.34$, $p = .179$). The same model estimated with OLS regression revealed similar results: an effect of minimalism ($\beta = .88$, $t(1{,}596) = 16.49$, $p < .001$), an effect for digital twins ($\beta = 1.34$, $t(1{,}596) = 4.02$, $p < .001$), and no significant interaction ($\beta = -0.05$, $t(1{,}596) = -0.67$, $p = .503$).

S10.3.4 Results - Additional Analyses (Non-Preregistered)

An analysis of the reasonings of five digital twins suggests that they expressed preferences in line with their minimalist orientations. For example, one twin reasoned, "My preferences lean toward vibrant, lively, and eclectic environments rather than minimalism. I feel more comfortable in spaces with lots of patterns, colors, and personal touches, making the first bedroom my clear favorite," and accordingly selected the non-minimalist option with a strong preference; in contrast, another twin reasoned, "I like a clean, organized, and calm environment for a home office. The minimalist and modern option fits my preferences for simplicity and order" and accordingly selected the minimalist option with a strong preference. The "extremeness" of these thoughts may possibly explain the relatively higher strength of the findings in the digital twins sample compared to the human sample. In other words, when it comes to the association between the minimalism scale and expressed preferences for room environments the twins tend to be more polarized than their human counterparts.

S10.3.5 Discussion

In conclusion, this study demonstrates that the predictive validity of the Minimalist Consumer Scale documented in human samples replicates robustly in a digital twin sample. Specifically, the study demonstrates that the scale predicts consumers' preferences for minimalist versus non-minimalist apartment interiors.

S10.4 Context Effects

S10.4.1 Main Questions/Hypotheses

Context effects are of great interest to marketers and policy makers alike because they suggest that product assortment can change choice. This study focuses on two classic context effects. The attraction effect occurs when a third option - an asymmetrically dominated decoy - is added to a binary choice set, increasing preference for the option that dominates the decoy [34]. The compromise effect occurs when a different type of decoy is added to a binary choice set, one that makes one of the original options appear as a compromise between the other two, increasing preference for the compromise option [35].

This study tests whether digital twins trained from LLMs can accurately predict individual choices in product purchase settings designed to elicit the attraction and compromise effects, using newly developed stimuli that twin models could not have encountered during training.

S10.4.2 Methods

Following an initial practice trial, all participants completed via Qualtrics three hypothetical product purchase decisions: one binary choice, and two trinary choices designed to elicit the attraction and compromise effects, respectively. The order of the three trials was counterbalanced across participants.

In each trial, participants were presented with a hypothetical purchase scenario and asked to choose between product options described in text by their price and quality features. Each trial was randomly assigned to one of the following three product categories: a printer, a TV, and a telephone plan. Importantly, all stimuli were newly developed for this study and could not have appeared in the digital twins' training data.

501 human participants (48% female, 52% male; 9% aged 18-29, 33% aged 30–49, 35% aged 50–64, 23% aged 65+) on Prolific completed the task. The study was subsequently run on their 501 digital twins. Human participants and their corresponding digital twins completed the same trials.

We recorded both choices and time spent on each decision for human participants, and only choice for digital twins.

S10.4.3 Results - Pre-registered Analyses

We began our analysis by testing whether humans and digital twins replicated the attraction and compromise effects. As preregistered, we excluded practice trials and combined the datasets from both humans and digital twins and created customized contrasts: for the attraction effect, attraction trials were coded as 1, binary trials as -1, and all other trials as 0. A similar coding scheme was used for the compromise effect. We ran binomial mixed-effect models predicting the choice of the target option. We included dataset and product type as fixed effects and accounted for the multilevel structure of the data by modeling respondent-level random intercepts, treating each

human and their digital twin as separate entities. Each contrast was nested within the human and digital twin datasets to separately capture the effects within each dataset.[3]

As shown in Figure S24, for the attraction effect, we replicated prior literature and found a significant effect among humans ($\beta = 0.18,\ SE = 0.07,\ z = 2.56,\ p = .01$). However, we did not detect an effect among digital twins ($\beta = -0.24,\ SE = 0.14,\ z = -1.67,\ p = .10$). Unexpectedly, we observed a strong main effect of dataset, such that digital twins made substantially more target choices overall ($\beta = 2.84,\ SE = 0.16,\ z = 17.30,\ p < .001$).

For the compromise effect, neither humans nor digital twins replicated prior results. No effect was found for humans ($\beta = 0.06,\ SE = 0.07,\ z = 0.87,\ p = .38$), and a significant negative effect was found for digital twins ($\beta = -0.54,\ SE = 0.15,\ z = -3.59,\ p < .001$). Again, we observed a strong main effect of dataset, with digital twins making substantially more target choices ($\beta = 2.90,\ SE = 0.17,\ z = 17.16,\ p < .001$).

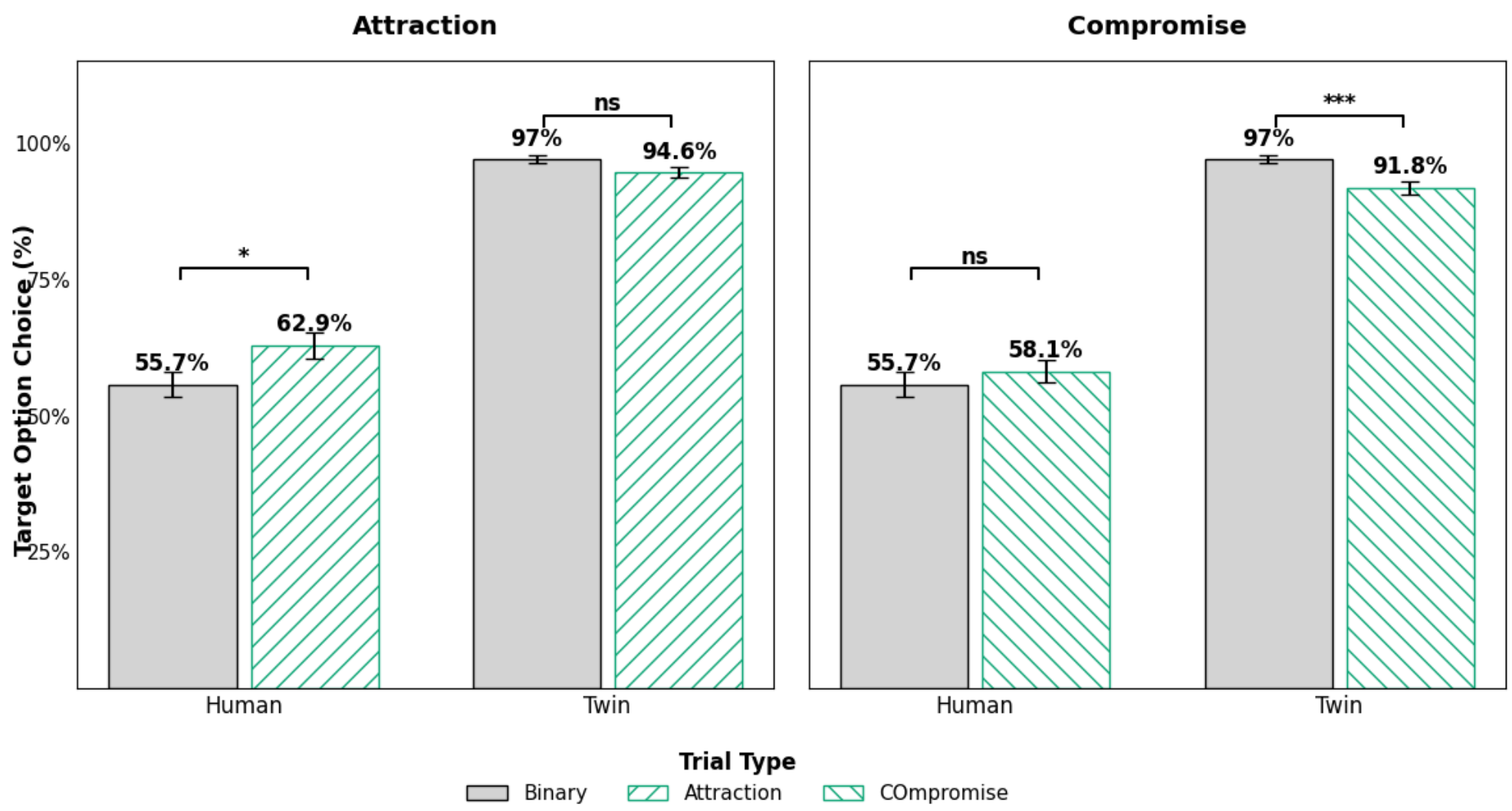

Fig. S24 Attraction and Compromise Effects. The *target option* refers to the option whose choice likelihood is expected to increase due to the addition of a decoy option. Vertical bars represent ±1 standard deviation based on raw data. Asterisks indicate significance levels derived from model-based contrast analyses: * $p < .05$, ** $p < .01$, *** $p < .001$; ns = not significant.

We also evaluated how accurately digital twins predicted human choices, independent of whether they predicted context effects. To examine possible variation in prediction accuracy by trial type, we broke the analysis down across binary, attraction, and compromise trials. For each trial, we created a binary variable denoting

[3]The nested model is a reparameterization of the preregistered interaction model. It is statistically equivalent but provides a more direct and interpretable test of the effect in each dataset.

whether the digital twin correctly predicted the participant's choice.[4] As a benchmark, we compared digital twin's accuracy level to that of a baseline model that randomly selected among the available options.

We found that digital twins predicted human choices more accurately than the random baseline across all trial types. Specifically, twins achieved 58% accuracy on binary trials (paired $t(500) = 2.86$, $p = .004$ vs. the 50% baseline), 59% on attraction trials ($t(500) = 8.52$, $p < .001$ vs. 33%), and 55% on compromise trials ($t(500) = 8.04$, $p < .001$ vs. 33%). Results were consistent when using McNemar's test, which evaluates the dichotomous accuracy indicator as a nominal variable rather than with a t-test.

S10.4.4 Results - Additional Analyses (Non-Preregistered)

Using response time data from human trials, we explored whether attention moderated the observed context effects. We constructed an attention factor based on log-transformed response times from binary, attraction, and compromise trials, and included interactions between this factor and the contrast variables in the nested model.

Among human participants, attention significantly moderated the compromise effect ($\beta = 0.17$, $SE = 0.07$, $z = 2.32$, $p = .02$), such that more attentive participants exhibited a significant compromise effect: a Johnson–Neyman analysis indicated the effect became statistically significant at values above 0.61 standard deviations of attention (relative to the mean), under a $p < .05$ threshold. No interaction was observed for the attraction effect ($\beta = 0.08$, $SE = 0.07$, $z = 1.06$, $p = .29$).

Attention factor derived from human participants did not moderate either context effect in the twin dataset (attraction: $\beta = -0.002$, $SE = 0.14$, $z = -0.01$, $p = .99$; compromise: $\beta = -0.12$, $SE = 0.14$, $z = -0.81$, $p = .42$).

S10.4.5 Discussion

We examined if large language models (LLMs) like ChatGPT can accurately predict the choices made by humans. In the current study, we successfully replicated the attraction effect in the human dataset, but digital twins did not reproduce this effect. Neither humans nor digital twins exhibited a compromise effect. However, the compromise effect was significant among human participants who paid more attention, as measured by response time. Independent of predicting context effects, digital twins showed only modest accuracy (less than 60% overall) in predicting individual human choices. These findings highlight cautions when using digital twins to predict human decisions in applied settings such as consumer choice.

[4] We created an indicator variable denoting whether the digital twin correctly predicted the participant's choice and used a McNemar test for analysis, rather than the preregistered correlation, as it is more appropriate for discrete choice outcomes.

S10.5 Default Effects

S10.5.1 Main Questions/Hypotheses

This study focuses on two default effects: the organ donation paradigm by [33], and a green energy default paradigm promoting sustainable energy choices [36]. In the organ donation study, participants who were defaulted to be donors were significantly more likely to remain enrolled than those who had to actively opt in. Similarly, in the green energy study, defaulting participants into using green energy suppliers increased adoption rates compared to requiring them to opt in.

This study tests whether digital twins trained from LLMs can accurately predict individual choices in these two settings, using the original organ donation stimuli verbatim and a slightly adapted version of the green energy scenario.

S10.5.2 Methods

All participants completed the original organ donation as well as the adjusted paradigm, presented in counterbalanced order. Across both default tasks, participants were randomly assigned to either an opt-in or an opt-out condition. In the organ donation task, participants in the opt-in condition were told they were not organ donors by default and were asked whether they wanted to become one. In the opt-out condition, participants were told they were organ donors by default and could choose to change that status. In the green energy default task, participants in the opt-in condition were told they were defaulted to the more affordable (non-green) energy option, while those in the opt-out condition were defaulted to the green energy option.

Notably, the organ donation task used the original stimuli from [33], while the green energy default task was slightly modified by changing the company names for the affordable and sustainable options.

600 human participants (47% female, 53% male; 11% aged 18–29, 33% aged 30–49, 34% aged 50–64, 22% aged 65+) on Prolific completed the task. The study was subsequently run on their 600 digital twins. Participants and their corresponding digital twins were assigned the same conditions.

We recorded both choices and time spent on each decision for human participants, and only choice for digital twins.

S10.5.3 Results - Pre-registered Analyses

We began our analysis by testing whether humans and digital twins replicated default effects in organ donation and green energy choice. As preregistered, for each default task, we combined data across both humans and digital twins and created a customized contrast: opt-out trials were coded as 1, opt-in trials as -1. We accounted for the multilevel structure of the data by including respondent-level random intercepts, treating each human and their digital twin as separate entities. This contrast was nested within human and digital twin datasets to separately estimate effects within each dataset.[5]

[5] The nested model is a reparameterization of the preregistered interaction model. It is statistically equivalent but provides a more direct and interpretable test of the effect in each dataset.

As depicted in Figure S25, in the green energy task, humans showed a large effect and replicated prior literature ($\beta = 0.50,\ SE = 0.08,\ z = 5.98,\ p < .001$), while digital twins did not ($\beta = 0.14,\ SE = 0.08,\ z = 1.73,\ p = .08$). We observed no main effect of dataset on green energy option adoption ($\beta = -0.22,\ SE = 0.12,\ z = -1.86,\ p = .06$).

In the organ donation task, we detected no effect among humans ($\beta = 0.09,\ SE = 0.08,\ z = 1.10,\ p = .28$), but a large effect among digital twins ($\beta = 0.92,\ SE = 0.11,\ z = 8.61,\ p < .001$). In addition, we observed a main effect of dataset, such that digital twins were more likely to become organ donors overall ($M_{\text{Human}} = 59\%,\ M_{\text{Twin}} = 71\%,\ \beta = 0.75,\ SE = 0.14,\ z = 5.53,\ p < .001$). Though this effect may be primarily driven by the difference in the opt-out condition ($M_{\text{Human}} = 61\%,\ M_{\text{Twin}} = 88\%$).

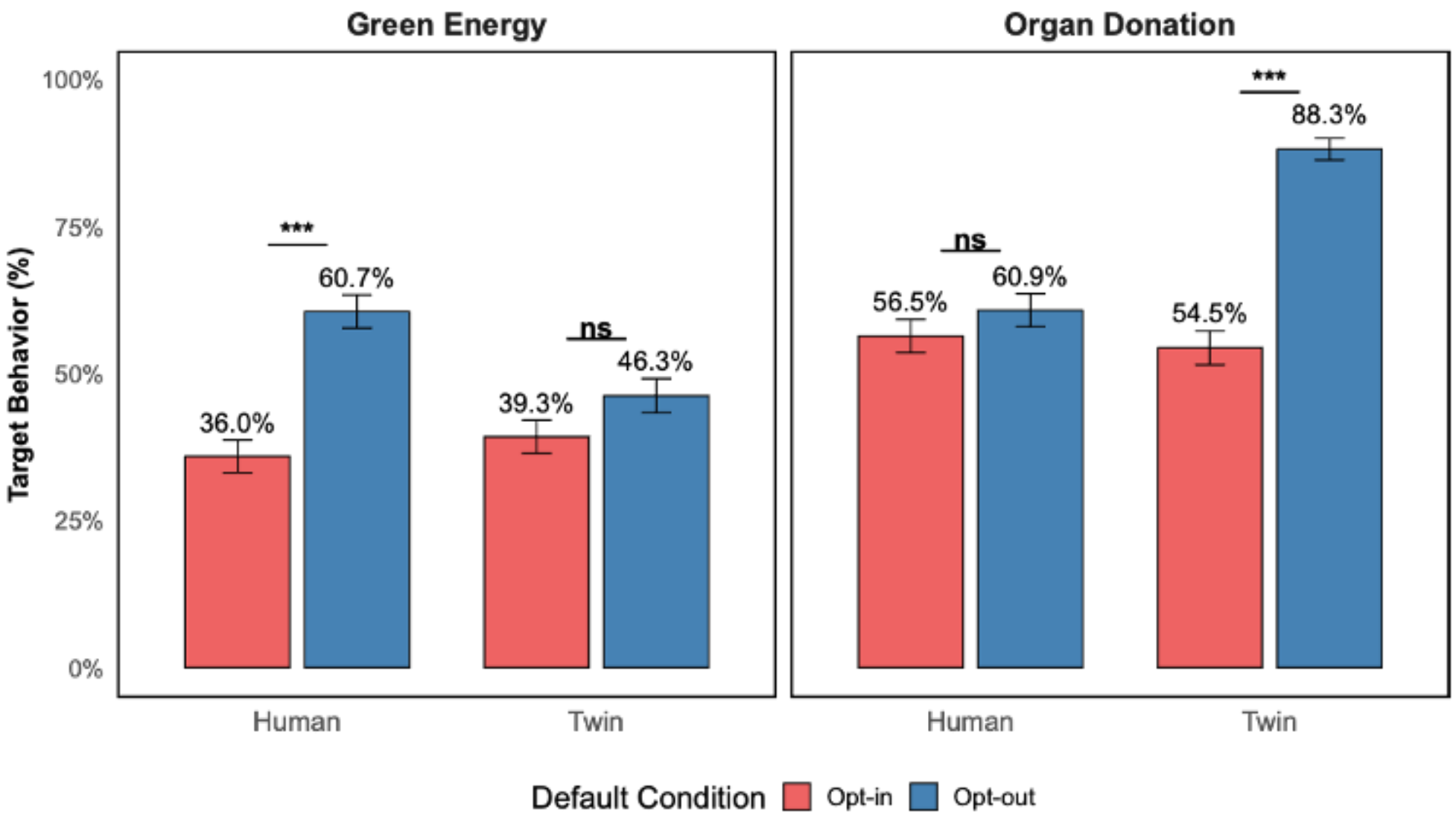

Fig. S25 Default Effects. Target behavior refers to adopting a green energy provider in the green energy paradigm and registering as an organ donor in the organ donation paradigm. Vertical bars represent ±1 standard deviation based on raw data. Asterisks indicate significance levels derived from model-based contrast analyses: * $p < .05$, ** $p < .01$, *** $p < .001$; ns = not significant.

We next examined how closely digital twins predicted human choices, independent of whether they predicted the default effects. For each trial, we created an indicator variable denoting whether the digital twin correctly predicted the participant's choice.[6] As a benchmark, we compared digital twin's accuracy level to that of a baseline model that randomly selected among the available options.

[6]We created an indicator variable denoting whether the digital twin correctly predicted the participant's choice and used a McNemar test for analysis, rather than the preregistered correlation, as it is more appropriate for discrete choice outcomes.

Across both paradigms, digital twins outperformed the random baseline. Specifically, twins achieved 69% accuracy on the green energy paradigm (paired t-test against 50% baseline, $t(599) = 7.11,\ p < .001$), and 58% on the organ donation paradigm (against 50%, $t(599) = 2.99,\ p = .003$). Results were consistent when using McNemar's test, which treats the dichotomous accuracy indicator as a nominal variable, instead of the t-test.

S10.5.4 Results - Additional Analyses (Non-Preregistered)

Using response time data from human trials, we explored whether attention moderated the observed context effects. We included interaction terms between the log-transformed and centered response time for each trial and the contrast variables in the nested model. We found no statistically significant moderation effects for either human participants (green energy: $\beta = -0.01,\ SE = 0.09,\ z = -0.15,\ p = .88$; organ donation: $\beta = -0.15,\ SE = 0.09,\ z = -1.71,\ p = .09$). Response time derived from the human dataset also did not moderate the effects in the digital twin models (green energy: $\beta = 0.06,\ SE = 0.08,\ z = 0.77,\ p = .44$; organ donation: $\beta = -0.09,\ SE = 0.10,\ z = -0.90,\ p = .37$).

S10.5.5 Discussion

Large language models (LLMs) like ChatGPT can accurately describe default effects and reference the relevant literature, presumably because these concepts are well represented in their training data. Default effects, while often robust, vary considerably in magnitude [61]. In the present study, human participants showed a default effect in the green energy adoption paradigm but not in the organ donation paradigm. Digital twins predicted a strong default effect in the organ donation paradigm but no effect in the green energy paradigm.

This discrepancy may stem from greater representation of the organ donation paradigm in the LLM's training data, while our modified green energy options may have diverged from commonly seen formats. Still, digital twins were able to predict human choices in the green energy paradigm with 69% accuracy, showing some promise in capturing people's underlying preferences.

S10.6 Digital Certificates for Luxury Consumption

S10.6.1 Main Questions/Hypotheses

Luxury brands face the perpetual problem of balancing exclusivity with growth [62]. Diffusion of products into the marketplace inevitably erodes exclusivity and negatively impacts product and brand value [63]. Recent work has demonstrated that a new technology—digital certification—reinforces value when luxury products become diffused [64]. In this study, we aim to replicate this pattern with human participants, and test whether their digital twins respond similarly to digital certification.

Specifically, we test whether attaching a digital passport to a luxury product that has become diffused increases its perceived value (i.e., status perceptions) and monetary value (i.e., price perceptions). Digital passports are a novel Blockchain-based certification system inspired by a recent European Union regulation to create more transparent and sustainable supply chains for physical products [65]. For example, the Aura Blockchain Collective (a non-profit collective of major luxury firms) provides digital passports that allow consumers to follow their purchases through the entire product journey, from the origin of the raw materials to recycling.[7]

S10.6.2 Methods

We opened the study to 600 human participants on Prolific (46.50% female, 53.50% male; $M_{\text{age}} = 49.86$, $SD = 14.32$). The study was subsequently run on their 600 digital twins, and the final sample size was 1,200 for our analysis. Participants and their corresponding digital twins were randomly assigned to one of two conditions in a two-cell (Digital Certification: Digital Passport vs. Control) between-subjects design (each human and their digital twin were assigned to the same condition).

All participants were asked to select one of six luxury sweaters: "Here is a selection of 6 sweaters by a luxury brand. Please choose the sweater that you are most interested in." Digital twins were given a textual description of each sweater, rather than an image, to enable comparison between humans and their digital counterparts, who cannot process visual images. The description of each sweater was based on real sweaters designed by Ralph Lauren ($M_{\text{price}} = \$531.90$). To avoid potential confounds, the brand was not mentioned. These six target sweaters were chosen from a set of 20 after an extensive pretest to ensure the products were equally liked, perceived as high-end, suitable for the scenario (i.e., as an outfit for going out), and conspicuous. Additional exploratory analyses controlled for participants' selected sweater to account for differences across product stimuli (e.g., sweater color, style) that could influence value perceptions.

After choosing a sweater, participants in the digital passport condition read as follows:

> We will ask you a few questions about the luxury sweater you selected. This luxury sweater is linked to a permanent digital record available on a public website, showcasing the current owner. This allows anyone interested to verify the ownership of your specific luxury sweater

[7] "Solutions - Aura Blockchain Consortium" (n.d.), (accessed February 18, 2025), [available at https://auraconsortium.com/solutions].

through this accessible record. Essentially, the digital record serves as unequivocal evidence of your luxury sweater's ownership.

Participants in the control condition alternatively read: "We will ask you a few questions about the luxury sweater you selected." All conditions next featured a scenario in which the luxury product has become diffused. Specifically, all participants read, "Imagine you decided to purchase this luxury sweater and style it for your night out, but during your night out you noticed many other people wearing the same luxury sweater." Participants in the digital passport condition also read, "Nevertheless, you know there is a digital record associated with the particular luxury sweater you are wearing, reassuring your ownership." Finally, all participants completed a writing task to reinforce the manipulation: "Please imagine your night out in detail. Briefly describe how you look and how you would feel wearing this sweater during your night out."

Participants then responded to the dependent variables by reporting their perceptions of product value ("How expensive do you think the sweater is? (Please enter number of $)") and status perceptions ("Please answer the following questions. To what extent do you think the sweater is..."; luxurious, high status, prestigious; 1 = Not at all, 7 = To a great extent; Ward & Dahl, 2014). These three items were averaged for analysis ($\alpha = .94$). We log-normalized the price perception measure to address skewness ($\mu = 4.06 > 2$; i.e., right-skewed; [66]). Finally, participants reported the extent to which they were familiar with digital passports ("How familiar are you with digital passports?"; 1 = Very Unfamiliar, 7 = Very Familiar), the perceived difficulty of the task to account for different prompt lengths across conditions ("How difficult was it to imagine the sweater scenario?"; 1 = Extremely Easy, 7 = Extremely Difficult), and whether they owned real luxury products (0 = No, 1 = Yes).

S10.6.3 Results - Pre-registered Analyses

We estimated a linear mixed-effects regression with participant as a random effect, log-transformed price perceptions as the dependent variable, and Participant Type (Human = 0, Digital Twin = 1), Digital Certification (Control = 0, Digital Passport = 1), and their interaction as predictors. We found a significant effect of Digital Certification ($b = .25$, $SE = .05$, $t(1190.45) = 4.94$, $p < .001$), a significant effect of Participant Type ($b = .83$, $SE = .05$, $t(597.99) = 16.91$, $p < .001$), and a significant interaction ($b = -0.21$, $SE = .07$, $t(596.99) = -3.08$, $p < .01$; Figure S26), indicating that, at an aggregate level, the digital twins rated price perceptions differently than the humans. Pairwise comparisons confirmed that, for humans, price perceptions were significantly higher when their purchase was connected to a digital certification ($M = 5.26$, $SE = .04$) compared to no digital certification ($M = 5.01$, $SE = .04$; $t(1190) = 4.94$, $p < .001$). However, for the digital twins, price perceptions were not significantly different when their purchase was connected to a digital certification ($M = 5.88$, $SE = .04$) compared to no digital certification ($M = 5.84$, $SE = .04$; $t(1190) = 0.73$, $p = .463$).

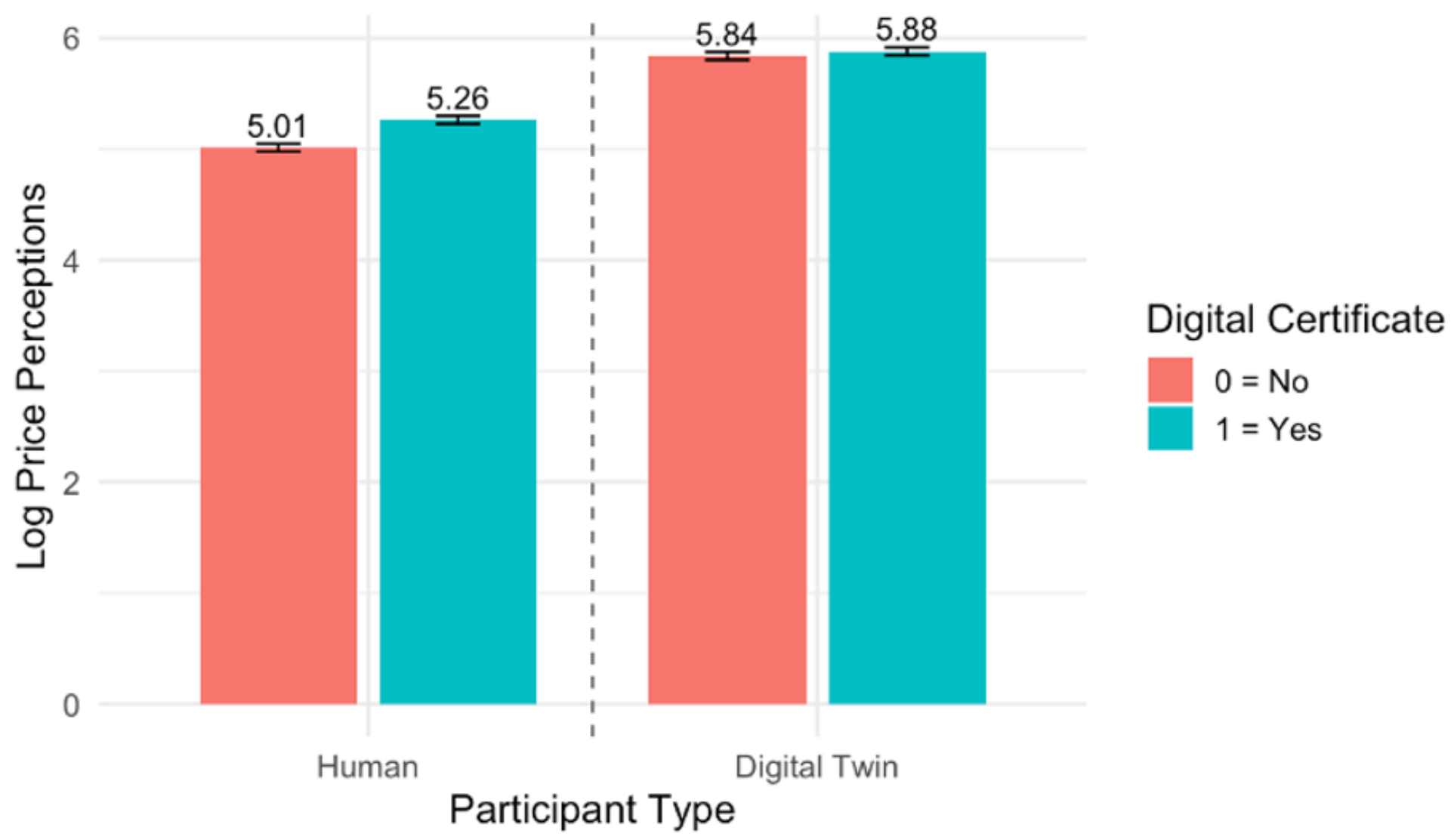

Fig. S26 Price Perceptions by Digital Certification for Humans vs. Twins. NOTE: Error bars indicate ±1 standard error of the estimated marginal means.

We next estimated a linear mixed-effects regression with participant as a random effect, status perceptions as the dependent variable, and Participant Type (Human = 0, Digital Twin = 1), Digital Certification (Control = 0, Digital Passport = 1), and their interaction as predictors. We found a non-significant effect of Digital Certification ($b = 0.14$, $SE = 0.10$; $t(1175.69) = 1.44$; $p = .151$), along with a significant effect of Participant Type ($b = 0.19$, $SE = 0.09$; $t(597.99) = 2.06$; $p = .040$) and a significant interaction ($b = -0.55$, $SE = 0.13$; $t(597.99) = -4.24$; $p < .001$; Figure S27), indicating that, at an aggregate level, the digital twins rated status perceptions differently than the humans. Pairwise comparisons confirmed that, for humans, status perceptions were marginally higher when their purchase was connected to a digital certification ($M = 4.81$, $SE = 0.07$) compared to no digital certification ($M = 4.66$, $SE = 0.07$; $t(1176) = 1.44$; $p = .150$). However, for the digital twins, status perceptions were significantly lower when their purchase was connected to a digital certification ($M = 4.44$, $SE = 0.07$) compared to no digital certification ($M = 4.85$, $SE = 0.07$; $t(1176) = -4.16$; $p < .001$).

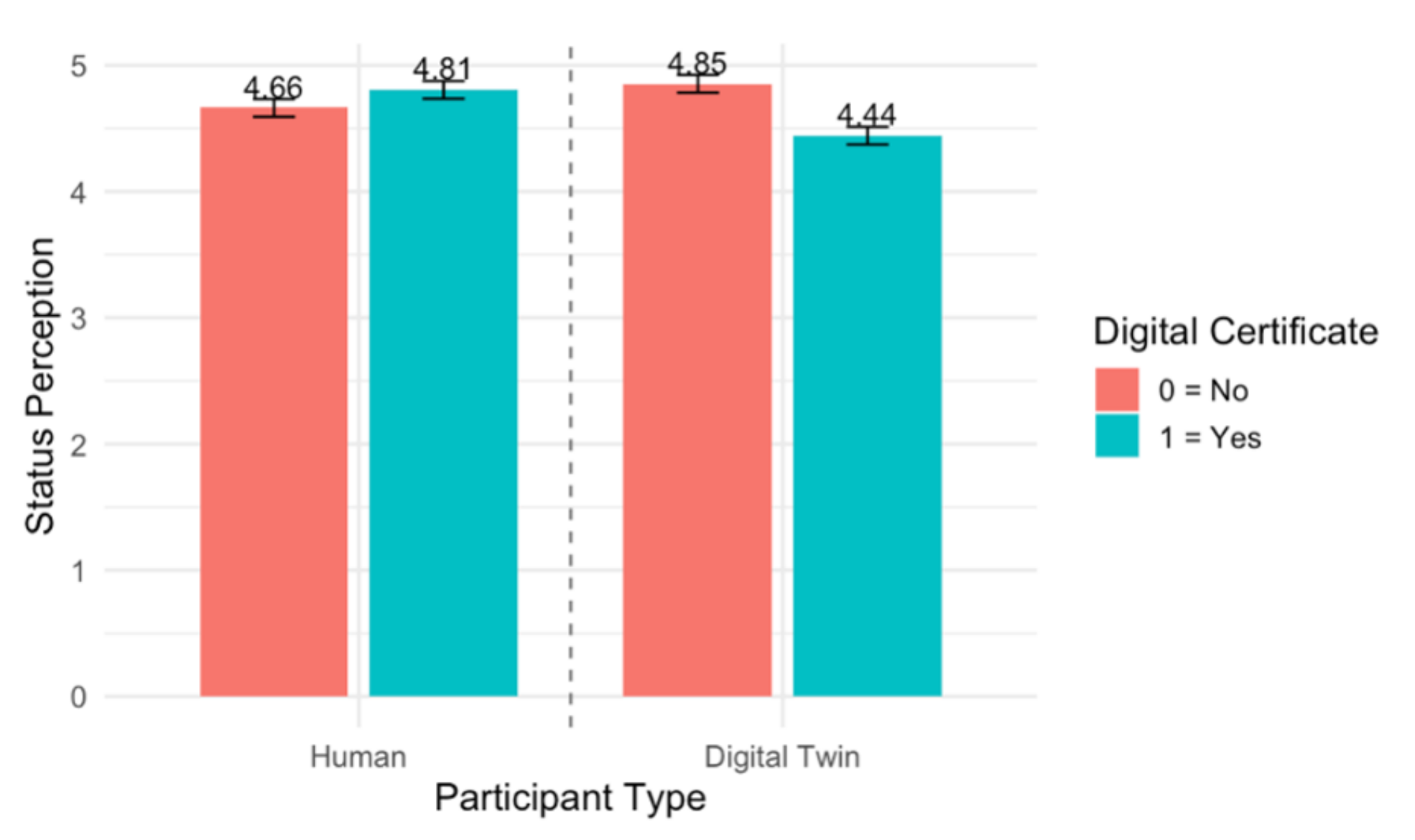

Fig. S27 Status Perceptions by Digital Certification for Humans vs. Twins. NOTE: Error bars indicate ±1 standard error of the estimated marginal means.

S10.6.4 Results - Additional Analyses (Non-Preregistered)

Although not part of our pre-registered plan, we ran two additional linear mixed-effects regressions on log-transformed price perceptions and status perceptions, controlling for the specific product selected by the participant, number of words generated in the imagination prompt, participants' familiarity with digital passports, difficulty of imagining the scenario, and personal ownership of luxury products. Before running these regressions, we examined differences between humans and their digital twins on each of these variables.

We first tested whether humans and their twins differed in the sweaters they selected. The results indicated weak but statistically significant agreement between twins and humans ($\kappa = .166$, $p < .001$). A chi-squared test of independence also revealed a significant and positive association between human and twin sweater choices ($\chi^2(1, N = 1200) = 297.4$, $p < .001$). Breaking down choice by the gender association of the sweaters, digital twins tended to mirror their human counterparts: when humans selected one of the three feminine sweaters, their twins also selected a feminine sweater 86.31% of the time ($\chi^2(1, N = 241) = 125.63$, $p < .001$); when humans selected one of the three masculine sweaters, their twins chose a masculine sweater 85.24% of the time ($\chi^2(1, N = 359) = 176.89$, $p < .001$).

We next checked whether all participants wrote roughly an equal number of words (log-corrected) between conditions. While the number of words generated did not differ between conditions for humans ($M_{\text{Digital Passport}} = 3.54$ vs. $M_{\text{Control}} = 3.47$; $F(1, 598) = 1.70$, $p = .20$, $d = .11$), the digital twins generated more words in

the Digital Passport condition ($M = 4.42$) compared to the Control ($M = 4.36$; $F(1, 598) = 66.92$, $p < .001$, $d = .67$). Additionally, the digital twins generated more text ($M = 4.42$) compared to their human counterparts ($M = 3.50$; $F(1, 599) = 1305.28$, $p < .001$, $d = 1.47$).

We then tested for perceived familiarity with digital passports. Across humans and twins, there were no differences in perceived familiarity among participants in the Digital Passport condition ($M = 2.40$) compared to the Control condition ($M = 2.36$; $F(1, 1198) = 0.20$, $p = .65$, $d = .03$). Human participants, however, reported being more familiar with digital passports ($M = 2.68$) compared to twins ($M = 2.08$; $F(1, 599) = 74.54$, $p < .001$, $d = 0.35$).

We then tested for differences in difficulty of imagining the scenario between conditions. Human participants in the Digital Passport condition found the imagination task more difficult ($M = 2.68$) than in the Control condition ($M = 2.03$; $F(1, 598) = 32.70$, $p < .001$, $d = 0.47$). Digital twins also found the imagination task more difficult in the Digital Passport condition ($M = 2.59$) than in the Control condition ($M = 2.31$; $F(1, 598) = 22.32$, $p < .001$, $d = 0.39$). There were no differences in difficulty of imagining the tasks between humans ($M = 2.35$) and twins ($M = 2.45$; $F(1, 1198) = 2.26$, $p = .13$, $d = 0.09$).

We finally tested for differences in reported ownership of luxury products. Across humans and twins, there were no differences in luxury product ownership among participants in the Digital Passport condition (40.4%) compared to the Control condition (39.3%; $\chi^2(1) = 0.12$, $p = .73$). Human participants, however, reported higher luxury ownership (53.8%) compared to their digital twins (25.8%; $\chi^2(1) = 96.97$, $p < .001$).

To test whether our findings persist when controlling for these differences, we ran a linear mixed-effects regression with participant as a random effect, log-normalized perceived price as the dependent variable, and Participant Type (Human = 0, Digital Twin = 1), Digital Certification (Control = 0, Digital Passport = 1), and their interaction as predictors, controlling for the product participants selected, as well as familiarity with digital passports, difficulty of imagination, and ownership of luxury products. We found a significant effect of familiarity ($b = 0.05$, $SE = 0.01$; $t(1190.90) = 3.48$, $p < .001$) and luxury product ownership ($b = 0.20$, $SE = 0.04$; $t(1170.09) = 5.10$, $p < .001$), but a non-significant effect of product choice ($b = 0.02$, $SE = 0.01$; $t(1190.85) = 1.48$, $p = .14$) and difficulty of imagination ($b = -0.02$, $SE = 0.02$; $t(1190.82) = -1.51$, $p = .13$). Nevertheless, the analysis revealed a significant effect of Digital Certification ($b = 0.28$, $SE = 0.05$; $t(1189.85) = 5.53$, $p < .001$) and a significant effect of Participant Type ($b = 0.96$, $SE = 0.05$; $t(726.87) = 17.88$, $p < .001$), qualified by a significant interaction between Digital Certification and Participant Type ($b = -0.25$, $SE = 0.07$; $t(603.74) = -3.66$, $p < .001$).

Next, we ran a linear mixed-effects regression with participant as a random effect, status perceptions as the dependent variable, and Participant Type (Human = 0, Digital Twin = 1), Digital Certification (Control = 0, Digital Passport = 1), and their interaction as predictors, again controlling for the product participants selected, as well as familiarity with digital passports, difficulty of imagination, and ownership of luxury products. We found a significant effect of familiarity ($b = 0.07$, $SE = 0.03$; $t(1185.69) = 2.43$, $p = .02$) and luxury product ownership ($b = 0.46$, $SE = 0.08$;

$t(1180.77) = 6.09$, $p < .001$), but a non-significant effect of product choice ($b = -0.03$, $SE = 0.02$; $t(1185.33) = -1.43$, $p = .15$) and difficulty of imagination ($b = -0.02$, $SE = 0.03$; $t(1191.82) = -0.70$, $p = .48$). Nevertheless, the analysis revealed a marginal effect of Digital Certification ($b = 0.17$, $SE = 0.10$; $t(1183.86) = 1.72$, $p = .08$) and a significant effect of Participant Type ($b = 0.34$, $SE = 0.10$; $t(722.27) = 3.30$, $p < .01$), qualified by a significant interaction between Digital Certification and Participant Type ($b = -0.59$, $SE = 0.13$; $t(598.73) = -4.51$, $p < .001$).

These additional analyses strengthen our confidence that the effects are robust across individual product choice, familiarity with the digital certification context (i.e., digital passports), ease of completing the task, and luxury product ownership.

S10.6.5 Discussion

These findings demonstrate that providing digital certification via a digital passport increases the value perception of a diffused luxury product for humans, consistent with existing research [64]. Digital twins' value perceptions, however, are not impacted by the inclusion of a digital passport. The findings have important implications for our understanding of when digital twins will not act as their human counterparts.

In this study, the divergence may stem from the psychological reassurance of product authenticity that certification provides to human consumers [64], which digital twins cannot fully internalize. This highlights a potential blind spot in current AI models: a limited ability to model symbolic consumption, ownership signaling, or status restoration processes. More broadly, these findings call for caution when relying on digital twins to simulate human judgments in domains involving identity and social perception—especially in luxury or prestige-based contexts. Future work should explore how these divergences arise and whether digital twins can be improved to better simulate symbolic inference processes, or whether some aspects of human value perception are fundamentally non-transferrable.

S10.7 Fees Accuracy

S10.7.1 Main Questions/Hypotheses

Hidden fees and surcharges are a routine but often resented aspect of modern commerce. Whether booking a hotel, ordering food delivery, or using a credit card bill, consumers frequently encounter extra, often unexpected charges—like resort fees, service fees, foreign transaction fees, or order processing fees—added on top of the advertised base price. These fees are often disclosed late in the transaction, hindering consumers' ability to estimate total costs or compare options, and are often described using obtuse language, obscuring understanding of why the fees are assessed. As a result, they have attracted growing scrutiny from consumer advocates and regulators. In recent years, a bipartisan group of regulators in the United States and other countries have advocated for stronger pricing transparency and restrictions on the delayed disclosure of mandatory charges. In 2022, the Biden-Harris administration labeled these "junk fees," defined as "fees designed either to confuse or deceive consumers or to take advantage of lock-in or other forms of situational market power" [67].

Against this policy backdrop, we explore how consumers think about these fees —how knowledgeable they are on what these fees represent, how fair they perceive them to be, and how supportive they are of government regulation regarding firms' use of such fees. We also examine whether large language models (LLMs), acting as digital twins, can replicate their human counterparts in these domains.

Specifically, we ask the following questions. First, do digital twins accurately replicate human knowledge of pricing fees? Second, do they mirror human judgment about fairness? Third, do they match human attitudes toward regulation?

S10.7.2 Methods

We recruited 400 U.S. participants from Prolific (48% female; median age group = 50–64) to complete an online survey assessing their knowledge and attitudes toward 19 different fees assessed across seven industries (e.g., hospitality, healthcare, rental housing). Each participant evaluated one randomly assigned fee from six of the seven industries. See Table S11 for the full list of industries and fees.

Industry	Fee	Definition
Hotels	Resort fee	A mandatory fee for a group of services, such as pool use, gym access, towel services, Wi-Fi, newspapers, shuttle service, daily parking, etc.
	Destination fee	A mandatory fee for a group of services, such as pool use, gym access, towel services, Wi-Fi, newspapers, shuttle service, daily parking, etc.
	Restocking fee	A fee charged by hotels for using the mini-fridge to store personal items.
Car Rentals	Vehicle licensing fee	A fee charged by rental car companies to offset costs associated with vehicle registration, licensing, and related taxes.

Industry	Fee	Definition
	Toll transponder fee	A daily fee assessed if a toll transponder is used during every day of the rental to pass quickly through toll booths.
	Concession recovery fee	A fee charged by rental car companies to customers to recover costs associated with operating at specific locations, such as airports.
	Frequent traveler program surcharge/excise tax	A fee for accumulating miles or points through credit card, airline, or other rewards programs.
Ticket Processing	Delivery fee	A fee for delivering tickets, whether physical or electronic.
	Order processing fee	A fee to cover costs associated with processing and completing ticket purchases.
	Facility charge	A fee to cover costs associated with hosting an event.
Food Delivery Apps	Service fee	A fee to operate the food delivery app platform.
	Express fee	A fee for prioritizing the matching process for delivery services.
	Regulatory response fee	A fee to offset the impact of regulations on firms.
Apartment Rentals	Valet trash fee	A fee for trash and recycling collection services.
	Move-in fee	A non-refundable fee to cover move-in and administrative costs, such as record updates, lock changes, and unit preparation.
	Amenity fee	A recurring fee that covers additional services and amenities, such as pools, gyms, parking, or other building facilities.
Health Care	Hospital facility fee	A fee charged by a private medical practice owned by a hospital to cover the hospital's operational and maintenance costs, including equipment, staff, and utilities.
Credit Cards	Foreign transaction fee	A fee for purchases or transactions in a foreign currency to cover conversion and international processing costs.
	Balance transfer fee	A percentage fee that covers debt transfers from one credit card to another.

Table S11: **Industries, Fees, and Definitions Used in the Survey.**

For each fee, we assessed the following. First, we assessed participants' objective knowledge by asking them to select the best definition of a fee from four options (e.g., "Which of the following do you think best represents what a resort fee is assessed for?"). We coded responses as 1 if correct and 0 if incorrect. We then averaged participants' responses across the six fees they evaluated to create an overall accuracy score. Immediately after responding, participants were informed whether their answer was correct and were shown the correct definition. Second, we assessed participants' familiarity with each fee. Specifically, participants indicated whether they had previously heard of each fee (e.g., they responded to questions like "Have you ever heard of a resort fee before seeing it in this survey?"). These items capture prior experience and are part of a separate project and were not pre-registered. Third, we assessed participants' fairness perceptions. Participants rated how fair they considered each fee (e.g., they responded to questions like "How fair do you think it is for hotels to charge for a resort fee?") on a 7-point scale (1 = Very unfair; 7 = Very fair). We then averaged participants' responses across the six fees they evaluated to create an overall fairness rating. Finally, we assessed participants' attitudes regarding policy. Specifically, after completing the fee evaluations, participants responded to several items about their general attitudes toward pricing practices and regulation. These included two pre-registered items: "Should pricing practices be regulated by the government?" rated on a 7-point scale (1 = Strongly oppose regulation; 7 = Strongly support regulation), and "How likely would you be to support government regulation that bans firms from separating out mandatory fees from base prices?" rated on a 7-point scale (1 = Very unlikely; 7 = Very likely). Responses to these two items were highly correlated ($r = .72$), so we averaged them to create a composite measure of support for regulation.

Each participant's responses were paired with those of an LLM-generated digital twin prompted to respond as that individual would.

S10.7.3 Results - Pre-registered Analyses

Knowledge Accuracy. Digital twins dramatically outperformed human participants in identifying correct definitions of fees, based on a paired t-test ($t(399) = 45.89,\ p < .001,\ d = 3.26$). Humans answered 51.75% of fee definition questions correctly (SD = 20.87%), while digital twins answered 99.88% correctly (SD = 1.86%).

Fairness. Despite the accuracy gap, average fairness ratings were virtually identical. Both human participants ($M = 3.28,\ SD = 1.24$), and their digital twins ($M = 3.27,\ SD = 0.62$) rated the fees as moderately unfair, and their ratings did not significantly differ based on a paired-sample t-test ($t(399) = 0.14,\ p = .886,\ d = 0.01$). Table S12 reports fee-specific fairness comparisons.

Fee	Human M (SD)	Twins M (SD)	Paired t-test
Hotel Fees			
Resort Fee	3.04 (1.80)	2.95 (0.81)	$t(131) = 0.53,\ p = .596,\ d = 0.06$
Destination Fee	2.93 (1.68)	3.12 (0.81)	$t(133) = 1.24,\ p = .215,\ d = 0.15$

Fee	Human M (SD)	Twins M (SD)	Paired t-test
Restocking Fee	2.29 (1.61)	2.23 (0.50)	$t(133) = 0.44,\ p = .660,\ d = 0.05$
Car Rental Fees			
Vehicle Licensing Fee	3.44 (1.79)	3.71 (0.86)	$t(100) = 1.46,\ p = .149,\ d = 0.20$
Toll Transponder Fee	4.15 (1.83)	3.27 (0.83)	$t(99) = 4.44,\ p < .001,\ d = 0.62$
Concession Recovery Fee	3.29 (1.70)	3.34 (0.81)	$t(98) = 0.29,\ p = .769,\ d = 0.04$
Frequent Traveler Program Surcharge/Excise Tax	2.43 (1.50)	2.67 (0.67)	$t(99) = 1.65,\ p = .101,\ d = 0.20$
Ticket Processing Fees			
Delivery Fee	2.56 (1.56)	3.61 (0.91)	$t(130) = 7.05,\ p < .001,\ d = 0.82$
Order Processing Fee	3.05 (1.85)	3.48 (0.87)	$t(134) = 2.83,\ p = .005,\ d = 0.28$
Facility Charge	3.36 (1.80)	3.51 (0.82)	$t(133) = 0.93,\ p = .355,\ d = 0.10$
Food Delivery App Fees			
Service Fee	3.91 (1.78)	3.86 (0.77)	$t(133) = 0.33,\ p = .744,\ d = 0.04$
Express Fee	3.64 (1.77)	3.00 (0.76)	$t(132) = 4.10,\ p < .001,\ d = 0.46$
Regulatory Response Fee	2.69 (1.66)	2.59 (0.57)	$t(132) = 0.72,\ p = .472,\ d = 0.08$
Apartment Rental Fees			
Valet Trash Fee	3.66 (1.88)	3.36 (0.96)	$t(133) = 1.73,\ p = .086,\ d = 0.20$
Move-In Fee	3.10 (1.71)	3.58 (0.83)	$t(133) = 2.89,\ p = .004,\ d = 0.36$
Amenity Fee	3.70 (1.66)	3.55 (0.80)	$t(131) = 1.00,\ p = .320,\ d = 0.11$
Health Care Fees			
Hospital Facility Fee	3.28 (1.94)	2.81 (0.79)	$t(133) = 2.80,\ p = .006,\ d = 0.30$
Credit Card Fees			
Foreign Transaction Fee	4.30 (1.69)	3.86 (0.93)	$t(131) = 2.76,\ p = .007,\ d = 0.33$
Balance Transfer Fee	3.56 (1.87)	3.63 (0.78)	$t(133) = 0.46,\ p = .643,\ d = 0.05$

Table S12: **Per-Fee Fairness Comparisons Between Human and AI (paired-samples).**

We also compared the variation in fairness judgments between humans and digital twins. Humans expressed significantly more variation in their fairness ratings than did their digital twins, based on a Levene's test ($F(1, 798) = 180.48,\ p < .001$).

Policy Attitudes. We regressed support for regulation on participants' ideology (mean-centered, 1 = very liberal to 5 = very conservative), source (Human vs. AI), and their interaction. The main effect of political ideology was significant ($b = -0.44,\ SE = 0.06,\ t(796) = -7.78,\ p < .001$), such that more conservative participants expressed lower support for regulation. The main effect of source was also significant ($b = 0.44,\ SE = 0.09,\ t(796) = 4.69,\ p < .001$), with AI twins showing greater support for regulation than their human counterparts. Finally, the interaction between political ideology and source was significant ($b = -0.53,\ SE = 0.08,\ t(796) = -6.67,\ p < .001$), demonstrating that the ideology effect was stronger for the digital twins than their human counterparts (see Figure S28).

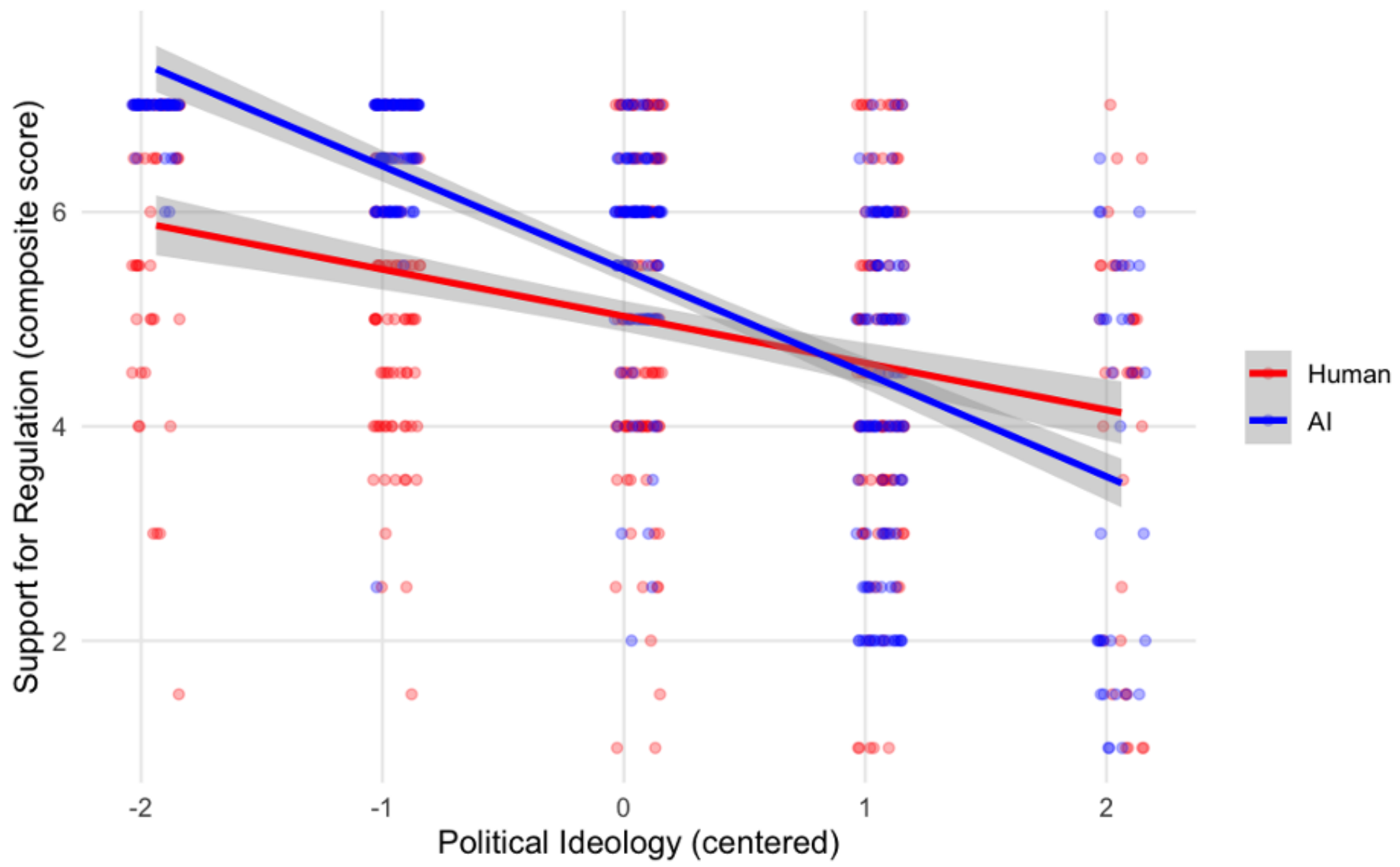

Fig. S28 Support for Regulation as a Function of Political Ideology and Participant Source.

S10.7.4 Discussion

Our findings reveal a striking contrast: digital twins excel at factual knowledge (i.e., knowledge of fee definitions) and fall short of mimicking the error-prone, heterogeneous reasoning of human consumers. This limitation underscores a critical boundary in using LLMs as behavioral research surrogates: when tasks depend on limited knowledge, ambiguity, or lived experiences, digital twins may produce unrealistically accurate responses.

In contrast to the large difference in accuracy, on fairness judgments, digital twins closely mirrored the average human response, but exhibited narrower variance. This suggests that while LLMs can approximate central tendencies, they may underrepresent the subjective diversity of human opinion.

In the domain of policy attitudes, digital twins again tracked human performance. For both groups, conservatism was correlated with less support for pricing regulation, replicating prior work on ideological influences in policy attitudes (e.g., [68]). However, digital twins exhibited exaggerated ideological consistency with a stronger negative association between conservatism and regulation support than human participants. This likely reflects LLM's reliance on dominant associations in their training data (e.g., conservatism = free-market values), whereas human responses may incorporate more contextual nuance.

Together, these results offer promise and caution. The results suggest that LLMs can simulate certain aspects of consumer judgment, but may misrepresent human

variability and error, especially when tasks require subjective or uncertain reasoning. Because LLMs are trained to prioritize factual accuracy and statistical associations from vast textual corpora, they often struggle to suppress correct information, even when explicitly instructed to simulate human errors, misconceptions, or gaps in knowledge.

S10.8 Heterogeneous Story Beliefs

S10.8.1 Main Questions/Hypotheses

When reading a book and predicting the emotion (valence and arousal) of the next chapter based on the previous chapter, what is the correlation between the expected valence and expected arousal from humans vs. their digital twins (LLMs)?

We hypothesize that LLM-based digital twins can meaningfully simulate human expectations about emotional trajectories in narrative contexts, as measured by correlations between human and LLM predictions of valence and arousal for upcoming story content.

S10.8.2 Methods

250 participants were recruited via Prolific with stratified sampling to ensure equal representation across racial groups (50 participants each from White, Black, Hispanic, Asian, and Other categories), and their digital twins.

Participants read chapters from 16 possible stories, with each participant randomly assigned to read 2 stories. Each story consisted of two chapters. Participants made predictions after reading each chapter: after Chapter 1 (predicting Chapter 2) and after Chapter 2 (predicting what would come next), resulting in 4 prediction points per participant.

Primary Dependent Variables:

1. *Human valence expectation*: Participants assigned probabilities to five valence levels for the next chapter:
   - Very negative (1) to Very positive (5)
   - Question: "Based on the chapter you just read, what do you think is the likelihood that the text of the next chapter will be very negative or very positive? Please assign a percentage to each. The total must sum to 100%."
2. *Human arousal expectation*: Participants assigned probabilities to five arousal levels for the next chapter:
   - Very low energy (1) to Very high energy (5)
   - Question: "Based on the chapter you just read, what do you think is the likelihood that the text of the next chapter will be very low energy or very high energy? Please assign a percentage to each. The total must sum to 100%."

From these probability distributions, we calculated expected values to create continuous measures of expected valence and arousal.

S10.8.3 Results - Pre-registered Analyses

The analysis included all prediction points (predictions made after both Chapter 1 and Chapter 2 of each story):

1. Valence Expectations:
   (a) Pearson correlation: $r = 0.546$ [95% CI: 0.501, 0.588]

(b) Statistical significance: $t(1002) = 20.65,\ p < .001$

2. Arousal Expectations:
   (a) Pearson correlation: $r = 0.384$ [95% CI: 0.330, 0.436]
   (b) Statistical significance: $t(1002) = 13.17,\ p < .001$

Both correlations were statistically significant at $p < .001$, indicating that LLM predictions are significantly associated with human expectations for both valence and arousal across all chapter transitions. The moderate positive correlations suggest meaningful alignment between human and LLM story belief patterns.

S10.8.4 Discussion

This study provides evidence that LLM-based digital twins can meaningfully simulate human emotional expectations in narrative contexts. The significant positive correlations for both valence ($r = 0.416$) and arousal ($r = 0.420$) support our hypothesis that LLMs can capture how humans predict emotional trajectories in stories.

S10.9 Hiring Algorithms

S10.9.1 Main Questions/Hypotheses

Digital twins, or AI agents trained to simulate individual behaviors, have the potential to reshape how job seekers and employers interact. From the candidate side, digital twins can help streamline the job search process by identifying roles aligned with the job seeker's preferences, tailoring job application materials, and even interacting with recruiters on the job seeker's behalf. For firms, digital twins enable the screening of candidates according to the firm's preferences, the simulation of candidate responses to different human resource policies, and even candidate outreach and outbound recruiting efforts. The advent of digital twins, and a growing ecosystem of start-ups offering such services, may thus fundamentally reshape the dynamics of hiring, and employee-employer matching more generally.

Despite the growing interest in digital twins, we lack evidence of their effectiveness to capture workplace preferences. While digital twins have shown promise in modeling preferences in more structured domains, there are unique challenges in the workplace context. For example, workplace preferences are multi-dimensional and candidates must weigh trade-offs between attributes like compensation, skill development, flexibility, and culture. Moreover, workplace preferences are shaped by social context and may vary over time. These features call into question the extent to which current digital twins can accurately represent how employees and firms navigate the matching process.

In this sub-study, we examine the ability of digital twins to predict job candidate preferences over workplace attributes using a two-stage study. In the first stage, we elicit the stated preferences of job candidates over a range of workplace characteristics, including work-life balance, career development, and company culture. We then ask each participant's digital twin to report their preferences over the same attributes. By comparing the stated and simulated preferences, we can understand how well digital twins predict the stated preferences of their human counterparts. In the second stage, we use a within-subjects experiment where participants evaluate four job postings. Our manipulation here changes whether the firm's hiring is done by algorithms or humans, and we randomly assign two of the four job postings to have AI recruiters. Both humans and their digital twins evaluate each of the four job postings on eight key attributes, including transparency, fairness, and likelihood of applying. This design thus allows us to understand the efficacy of digital twins in predicting workplace preferences and experiments in the HR domain.

S10.9.2 Methods

We run a two-stage study to examine these questions. We outline these stages in Figure S29. Both humans and their digital twins completed the study in the same order.

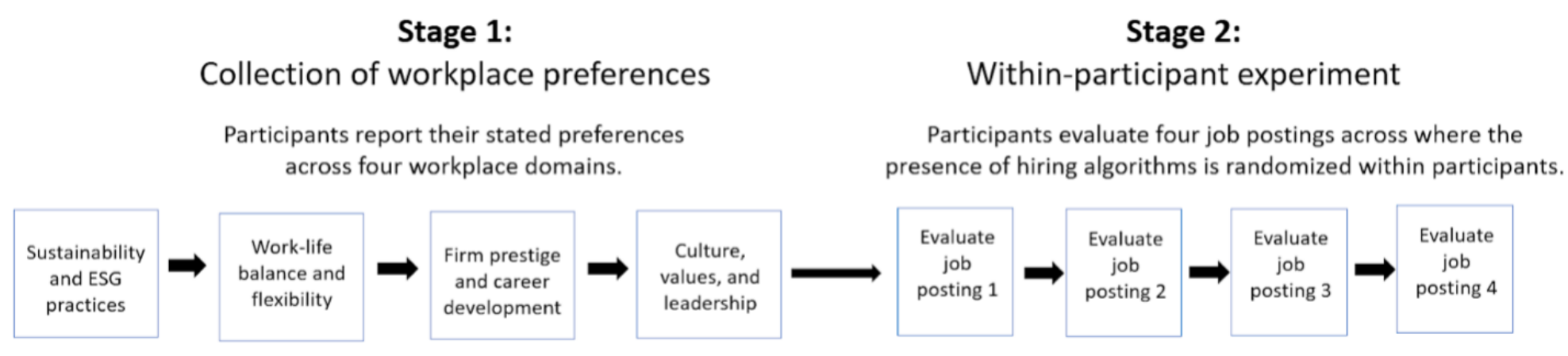

Fig. S29 Outline of the study.

The first stage of our study collects data on respondent workplace preferences. We asked respondents a total of eight questions across four distinct themes related to workplace preferences. These themes included:

- **Sustainability and ESG practices**
  - "How important is it to you that your employer actively invests in environmental sustainability (e.g., reducing carbon emissions, minimizing waste)?"
  - "Would you prefer to work at a company that is outspoken about social and political issues, or one that remains neutral?"
- **Work-life balance and flexibility**
  - "How likely are you to accept a job that requires you to be in the office five days a week?"
  - "Which of the following is more important to you when choosing a job? Where options included: (i) A flexible schedule that allows me to manage personal responsibilities; (ii) Clear boundaries between work and personal time, (iii) Strict 9-to-5 hours (iv) A predictable, fixed schedule[8]
- **Firm prestige & career development**
  - "How much do you value opportunities for formal training and upskilling in your current or future role?"
  - "Imagine two job offers: one from a prestigious company with a competitive culture, and one from a lesser-known company with a collaborative, supportive environment. Which would you choose?"[9]
- **Culture, values, and leadership**
  - "How important is it that your company's leadership communicates transparently about the firm's goals and challenges?"

[8] To construct the index measuring respondents' preferences for flexible work–life balance arrangements, we ordered the four response options from least to most flexible as follows: (1) *Strict 9–5 hours*, (2) *Predictable, fixed schedule*, (3) *Clear boundaries between work and personal time*, and (4) *Flexible schedule that allows me to manage personal responsibilities*. This index captures increasing preference for autonomy in managing one's work schedule.

[9] To construct the index for collaborative culture preference, we ordered the responses to reflect increasing preference for collaborative environments: (1) *Prestigious*, (2) *Depends on compensation*, (3) *Not sure*, and (4) *Collaborative*. This index captures respondents' orientation toward workplace culture, with higher scores indicating stronger preferences for collaboration over prestige or ambiguity.

– "How much do you care about your company's stated mission and values aligning with your personal beliefs?"

We collect these measures for both human participants and their digital twins. Thus for each participant, we have their stated preference regarding (i) sustainability & ESG practices; (ii) work-life balance and flexibility; (iii) firm prestige and career development; and (iv) culture and values. We also have the corresponding responses from the digital twins, allowing us to compare how well LLMs can predict job candidate preferences in the hiring process.

The second stage of our study uses an experiment with within-subjects variation to understand how candidates respond to algorithms in the hiring process. In this part, participants evaluate hiring policies from the perspective of a job seeker. We present participants with four different job postings, which display a position overview, the responsibilities, benefits, and hiring policy (See Figure S30 for an example). Our randomization manipulates whether hiring uses either human resume screeners or algorithmic ones. Following each posting, participants evaluated the job on eight questions (displayed in Table S13 below), using a 7-point Likert scale from strongly disagree to strongly agree.

Job Title: Entry Level Data Analyst

Position Overview: As an Entry Level Data Analyst, you will play a pivotal role in analyzing complex financial data, developing valuable insights, and supporting decision-making processes within our firm.

Responsibilities: The responsibilities for this position include collecting, cleaning, and manipulating large datasets to extract meaningful insights that support business objectives.

Benefits: Our benefits include a competitive salary commensurate with experience, health, dental, and vision insurance, and a collaborative and inclusive work environment.

Hiring Policy: Our hiring team will create insights on how your candidate information matches the requirements of the role. Following this initial screening, our hiring team will conduct in-person interviews with selected candidates.

Fig. S30 Sample Job Posting.

#	Question	Theme
1	I would apply for this position.	Likelihood of applying
2	I would be hired for this position.	Likelihood of receiving offer

#	Question	Theme
3	I will receive clear and transparent communication throughout the hiring process.	Transparency
4	The firm's hiring policies are fair and unbiased.	Fairness
5	The hiring process will move at a reasonable pace and ensure a timely decision-making process.	Efficiency and timeliness
6	The hiring process will give me a clear understanding of the job requirements, expectations, and the company culture.	Information
7	The hiring process will effectively identify top job applicants for the position.	Identifies top talent
8	The firm's hiring process suggests the firm fosters a high degree of social interaction.	Sociality

Table S13: **Questions regarding job postings.**

S10.9.3 Results - Pre-registered Analyses

We first examine whether digital twins can accurately predict human workplace preferences using two methods. First, we run paired t-tests to test whether on average the twin outcomes equal the actual human outcomes. Second, we use regression to estimate the size and direction of the bias.

Table S14 displays the results of the paired t-tests across all of our questions. The top panel contains the outcomes from the first phase of the study, while the bottom panel displays them from the second phase of the study. Column 1 displays the average human response while column 2 displays the average digital twin response. Column 3 displays the average difference in responses for the digital twin versus the human, with the corresponding t-test p-value in column 4. Lastly, column 5 displays the sample size for each group.

The results in Table S14 indicate that, on average, the outcomes generated by digital twins are statistically different from those generated by humans. Across the eight workplace preferences in phase 1 of the study (Panel A of Table S14), we only find evidence of no differences between the two for workplace flexibility preferences. For the other categories, we find statistically significant differences between the two. For most, the digital twins over-estimate human preferences for workplace attributes (for example, sustainability, ESG practices, collaboration, and transparency in leadership). Meanwhile, the digital twins under-estimate their human counterparts' preferences for career development opportunities.

We find qualitatively similar results for the second phase of the study, which we display in Panel B of Table S14. We find statistically significant differences across all outcomes here. Like before, the digital twins over-estimate some responses (for example, the likelihood of being hired for the position) and under-estimate others (the firm's degree of social interaction, and receiving clear information regarding job requirements, expectations, and company culture).

Panel A: Phase 1 Outcomes

Outcomes	Average Human (1)	Average Digital Twin (2)	Difference DT - H (3)	T-test p-value (4)	N (5)
Sustainability	2.88	3.45	0.57	0.00	999
ESG Practices	3.30	3.67	0.37	0.00	999
Work Life Balance	3.36	3.33	-0.03	0.40	999
Flexibility	2.97	2.95	-0.02	0.70	999
Collaboration over prestige	2.98	3.65	0.67	0.00	999
Career Development	3.67	3.56	-0.11	0.01	999
Transparent Leadership	3.83	4.43	0.60	0.00	999
Culture & Values	3.39	3.54	0.15	0.00	999

Panel B: Phase 2 Outcomes

Outcomes	Human (1)	Digital Twin (2)	DT - H (3)	p-value (4)	N (5)
I would apply for this position	3.34	3.48	0.14	0.00	999
I would be hired for this position	3.46	4.22	0.76	0.00	999
I will receive clear and transparent communication	4.89	4.79	-0.10	0.00	999
The firm's hiring policies are fair and unbiased	4.88	4.59	-0.29	0.00	999
The hiring process will move at a reasonable pace	5.01	4.55	-0.46	0.00	999
The hiring process will give me a clear understanding	5.20	4.70	-0.50	0.00	999
The hiring process will effectively identify top applicants	5.05	4.60	-0.45	0.00	999
The firm's hiring process suggests strong social interaction	4.61	4.05	-0.56	0.00	999

Table S14: **Comparison of human versus digital twin responses using t-tests.**

The regression tests of Hypothesis 1 lead to similar takeaways. In Table S15, we present the results of a regression of the human response on the twin's response with

robust standard errors. Columns 1 and 2 display the coefficient and standard error on the twin's response, respectively. Column 3 displays the p-value from a test of whether the coefficient on the twin response equals one. The corresponding null hypothesis is that digital twins can perfectly predict human responses (i.e., $\beta = 1$). As the table illustrates, however, we can reject the null across all of our outcomes. Thus, while the digital twins can predict workplace preferences for some participants, on average they struggle in this domain.

**Panel A: Phase 1 Outcomes**			
Outcomes	β (1)	Std. Err. (2)	p-value (3)
Sustainability	0.68	0.02	0.00
ESG Practices	0.55	0.02	0.00
Work Life Balance	0.11	0.03	0.00
Flexibility	0.14	0.03	0.00
Collaboration over prestige	0.12	0.04	0.00
Career Development	0.32	0.04	0.00
Transparent Leadership	0.45	0.05	0.00
Culture & Values	0.46	0.04	0.00
**Panel B: Phase 2 Outcomes**			
Outcomes	β (1)	Std. Err. (2)	p-value (3)
Apply	0.70	0.12	0.08
Hired	0.27	0.06	0.00
Communication	0.39	0.07	0.00
Unbiased hiring policies	0.41	0.06	0.00
Timely decision-making	0.38	0.06	0.00
Clear information	0.39	0.07	0.00
Identify top applicants	0.39	0.07	0.00
High degree of sociality	0.04	0.04	0.00

Table S15: **Comparison of human versus digital twin responses using regression.**

In our pre-analysis plan, we were also interested in whether digital twins can better predict outcomes for various demographic groups. We focused specifically on differences by gender, age, and race. For this analysis, we used the t-test strategy described above. We conducted a t-test for each specific subsample and report the p-values in Table S16. The last row of the table counts the number of times we can reject the null using a cut-off of $p < .05$. In other words, this row calculates the number of outcomes for the given subgroup where digital twin responses are statistically indistinguishable from human responses. Our results provide suggestive evidence that digital twins do better at predicting outcomes for women versus men, as we can reject the null of no differences between the human and twin responses for three outcomes for women (versus two for men). Similarly, the twins seem to do better

for older respondents versus younger ones. Overall, however, the differences across subgroups are quite limited.

	**Gender**		**Race**		**Age**			
Outcomes	Female (1)	Male (2)	White (3)	Non-White (4)	18–29 (5)	30–49 (6)	50–64 (7)	65+ (8)
Sustainability	0.00	0.00	0.00	0.00	0.00	0.00	0.00	0.00
ESG Practices	0.00	0.00	0.00	0.00	0.00	0.00	0.00	0.00
Work Life Balance	0.33	0.80	0.00	0.00	0.05	0.02	0.21	0.73
Flexibility	0.83	0.74	0.12	0.00	0.19	0.37	0.00	0.41
Collaboration	0.00	0.00	0.00	0.00	0.00	0.00	0.00	0.00
Career Development	0.17	0.01	0.03	0.10	0.00	0.00	0.25	0.07
Transparent Leadership	0.00	0.00	0.00	0.00	0.00	0.00	0.00	0.00
Culture & Values	0.00	0.01	0.00	0.01	0.00	0.03	0.00	0.00

Table S16: **Comparison of human versus digital twin responses using t-tests, by subgroup.**

We next examine whether experimentation on the digital twins can uncover the true treatment effect in the human sample. Although the previous results show that digital twins responses are biased across workplace attributes, valid causal inference may still be possible. For example, if the digital twins over-estimate the responses from all participants, estimation using the digital twins may still uncover the true average treatment effect in the human population if this bias is constant across treatment arms.

Our experimental manipulation is the use of algorithms in a firm's hiring policy. Each participant saw four job postings, where we randomly assigned two to use hiring algorithms (vs human screeners). For our estimation here, we regress our outcomes on a binary indicator for whether the job included a hiring algorithm, participant fixed effects, and job-type fixed effects. We do so for both our human sample and our digital twins sample, and plot the results in Figure S31. Moreover, we use a stacked regression to compare the treatment effects from our human vs digital twin sample. For this, we append the human and digital twin samples and create a binary indicator for the twin sample. We then regress each outcome on an indicator for the hiring algorithm condition, an indicator for the twin sample, and an interaction between the two. The interaction captures the extent to which the treatment effect in the twin sample is different from the effect in the human sample. We display these results in Table S17.

The results reveal that experimentation on digital twins can uncover unbiased treatment effects even in scenarios where digital twins struggle to predict workplace preferences. For example, the digital twins accurately recover the treatment effect from the human sample when examining how hiring algorithms impact the likelihood

of applying, being hired, and receiving timely information during the hiring process. These measures were where the digital twins failed to accurately predict human preferences at baseline.

In other cases, however, digital twins fail to recover the true treatment effect. The largest differences here are for beliefs regarding sociality at work and the ability of the firm to identify top talent. In these scenarios, digital twins substantially under-estimate the extent to which hiring algorithms depress human beliefs regarding the firm's hiring policy. We find similar results for clear communication, information regarding company culture, and whether hiring is fair and unbiased. This suggests that overall, digital twins are less adverse to algorithms compared to humans.

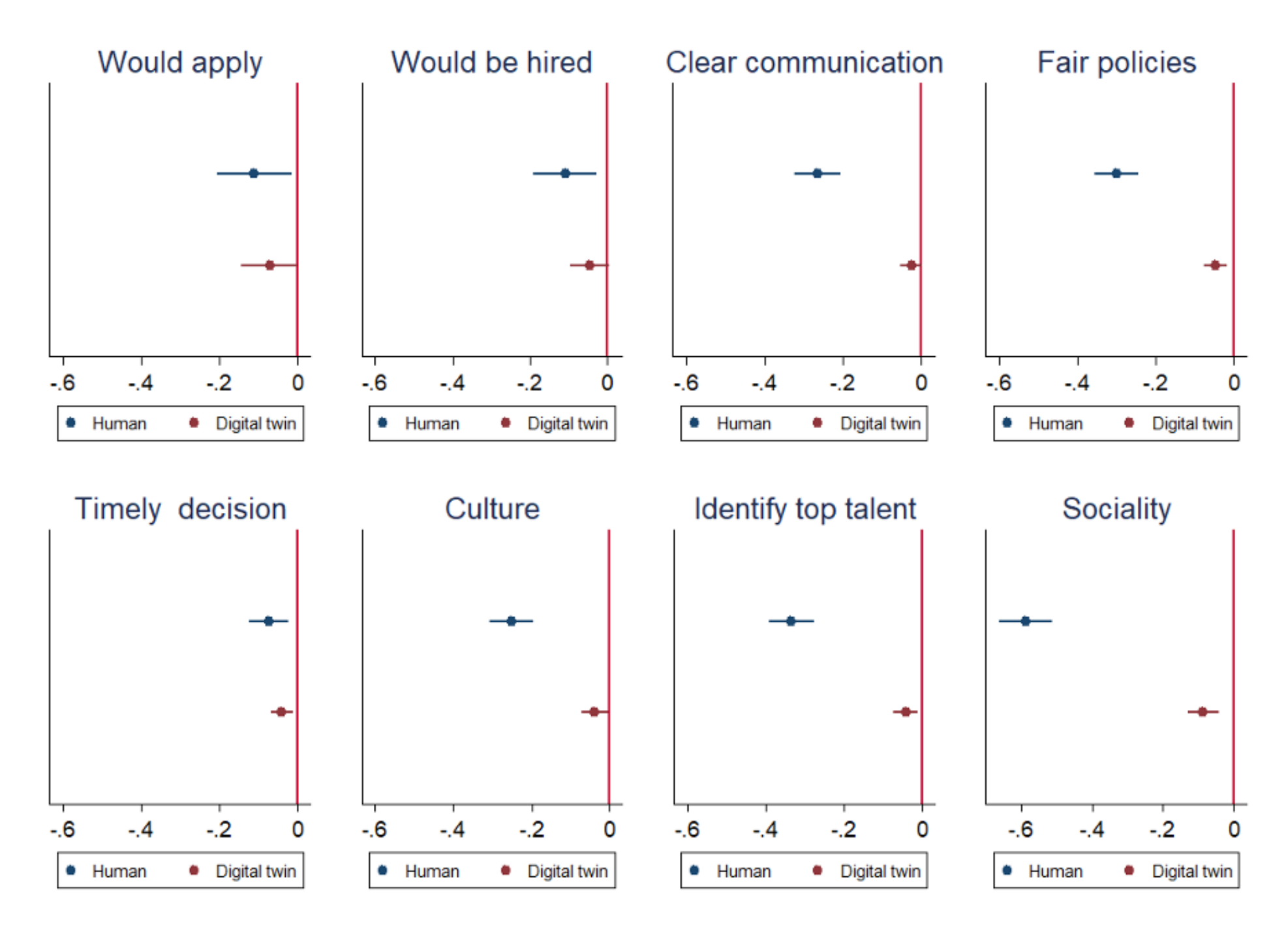

Fig. S31 Impact of Hiring Algorithms on Job Candidate Beliefs, For Humans Vs Their Digital Twins.

	Outcomes							
	Apply (1)	Hired (2)	Communication (3)	Fair Policies (4)	Timely Decision (5)	Clear Expectations (6)	Identify Talent (7)	Sociality (8)
AI	-0.11**	-0.11***	-0.27***	-0.30***	-0.07***	-0.25***	-0.33***	-0.59***
	(0.05)	(0.04)	(0.03)	(0.03)	(0.03)	(0.03)	(0.03)	(0.04)
Digital Twin	0.13***	0.73***	-0.22***	-0.42***	-0.47***	-0.61***	-0.60***	-0.81***
	(0.05)	(0.04)	(0.03)	(0.03)	(0.03)	(0.03)	(0.03)	(0.04)
AI × Digital Twin	0.04	0.06	0.24***	0.26***	0.03	0.22***	0.29***	0.50***
	(0.07)	(0.06)	(0.04)	(0.04)	(0.04)	(0.04)	(0.04)	(0.05)
N	7992	7992	7992	7992	7992	7992	7992	7992

Table S17: **Comparison of within-participant effects using stacked regressions, by outcome.**

S10.9.4 Discussion

Our results highlight several opportunities and drawbacks for the use of digital twins in human resource management. In terms of predicting preferences, we find that digital twins over-estimate human preferences for workplace attributes like collaboration, leadership transparency, and sustainability. Meanwhile, they are more accurate in estimating preferences for work-life balance, flexibility, and career development opportunities. This pattern suggests that while digital twins may be able to capture workplace preferences related to instrumental outcomes, they may struggle in domains with socially oriented or relational preferences, which are fundamental to modern-day workplaces.[10] Instead, digital twins over-estimate the importance of these attributes, relative to human jobseekers who are driven less by these attributes.

As both job candidates and employers adopt digital twins, understanding how digital twins shape the matching process is of utmost importance. Our results indicate digital twins can predict workplace preferences for job seekers in some domains, though future work remains. For example, one important future direction is understanding the training data necessary for digital twins to work at scale. While our setting features a broad array of background information used to program the digital twin [18], more targeted data collection, for example through the evaluation of hypothetical job postings, may increase the performance of digital twins in the hiring domain (see, for example, [70]). Another important avenue for future work is to understand how the labor market influences the performance of digital twins. For example, while digital twins may be able to predict preferences over short-term task assignments [71], they may struggle with more formal job opportunities, which may require a deeper appreciation of tradeoffs between financial incentives, meaning at work, pro-social tendencies, and other attributes.

[10] See, for example, [69], who show how automation interferes with workers' preference for sociality in the workplace.

S10.10 Idea Evaluation

S10.10.1 Main Questions/Hypotheses

First, we explore whether digital twins are able to replicate creativity ratings from their human counterparts: How do digital twins' ratings of idea creativity compare to those of their human counterparts?

Second, research has shown that humans tend to show AI aversion or human favoritism when evaluating the creativity of ideas [72], i.e., giving higher creativity ratings to ideas coming from humans when informed that the ideas come from humans (as opposed to AI). We explore whether the reverse is true for digital twins, i.e., do digital twins show human favoritism, AI favoritism, or neither when evaluating ideas?

S10.10.2 Methods

We used a 2 (human ideas vs. twin ideas) x 3 (baseline vs. partially informed vs. informed) design, targeting N=200 humans and their twins per condition. The ideas came from the "measures of creativity" sub-study, which generated 200 ideas from humans and 200 ideas from their digital twins. Participants in the "measures of creativity" sub-study were excluded from the "idea evaluation" sub-study. Each participant was randomly assigned to evaluating ideas from humans or twins and evaluated 20 ideas randomly selected from that set. Participants were further assigned randomly to one of three evaluation conditions, based on Zhang and Gosline (2023). In the baseline condition, no information was given on the source of the ideas. Participants were shown ideas (for smartphone apps that will help their users keep a healthier lifestyle) one at a time, and asked to rate the creativity of each idea on a 5-point scale ("very creative" to "very uncreative"). In the partially informed condition, participants were informed that "the ideas in this study came from two sources: -humans: Prolific participants who were asked to generate ideas; -AI: a large language model (Chat GPT or other) who was asked to generate ideas. The ideas you are about to evaluate came from one of these two sources. That is, they either all came from humans, or they all came from AI," followed by a comprehension check. Then they were shown 20 ideas one at a time ("Below is an idea that was generated either by a human or by AI.") which they were asked to rate for creativity on a 5-point scale. In the full information condition, participants were informed that "The ideas in this study came from two sources: -humans: Prolific participants who were asked to generate ideas; - AI: a large language model (Chat GPT or other) who was asked to generate ideas. The ideas you are about to evaluate all came from humans/AI," also followed by a comprehension check. They were shown 20 ideas ("Below is an idea that was generated by a human/AI") and asked to rate them for creativity on the same scale.

We obtained complete data from 1,174 respondents and their twins. Given the number of ideas (400), the number of evaluation conditions (3), and the number of ideas evaluated per participant (20), each idea was evaluated by 19.567 judges on average.

S10.10.3 Results - Pre-registered Analyses

How do digital twins' ratings of idea creativity compare to those of their human counterparts?

We first analyze human ideas and compare the average creativity rating of each idea in the baseline condition coming from twins vs. human judges. We find that human ideas are rated on average significantly higher by twins than they are by humans ($M_{\text{twins}} = 3.310,\ M_{\text{humans}} = 3.198,\ t = 2.859,\ p < .01$), and that the standard deviation of the creativity ratings across ideas is smaller for ratings coming from twins vs. humans ($S_{\text{twins}} = 0.223,\ S_{\text{humans}} = 0.530,\ F(199, 199) = 5.664,\ p < .001$). The mean absolute percentage error between ratings from twins vs. humans is 0.155. The correlation between ratings coming from twins vs. humans is positive but not statistically significant ($r = 0.112,\ p = .114$).

Next, we analyze twin ideas similarly. Here, we actually find the opposite pattern for average creativity ratings: twin ideas are rated on average significantly lower by twins than they are by humans ($M_{\text{twins}} = 3.213,\ M_{\text{humans}} = 3.322,\ t = 3.742,\ p < .01$). The standard deviation of the creativity ratings across ideas is again smaller for ratings coming from twins vs. humans ($S_{\text{twins}} = 0.172,\ S_{\text{humans}} = 0.396,\ F(199, 199) = 5.292,\ p < .001$). The mean absolute percentage error between ratings from twins vs. humans is 0.103. The correlation between ratings coming from twins vs. humans is positive but not statistically significant ($r = 0.103,\ p = .148$).

Do digital twins show human favoritism, AI favoritism, or neither when evaluating ideas?

Figure S32 shows the average creativity rating as a function of the identity of the ideator, the raters, and the evaluation condition.

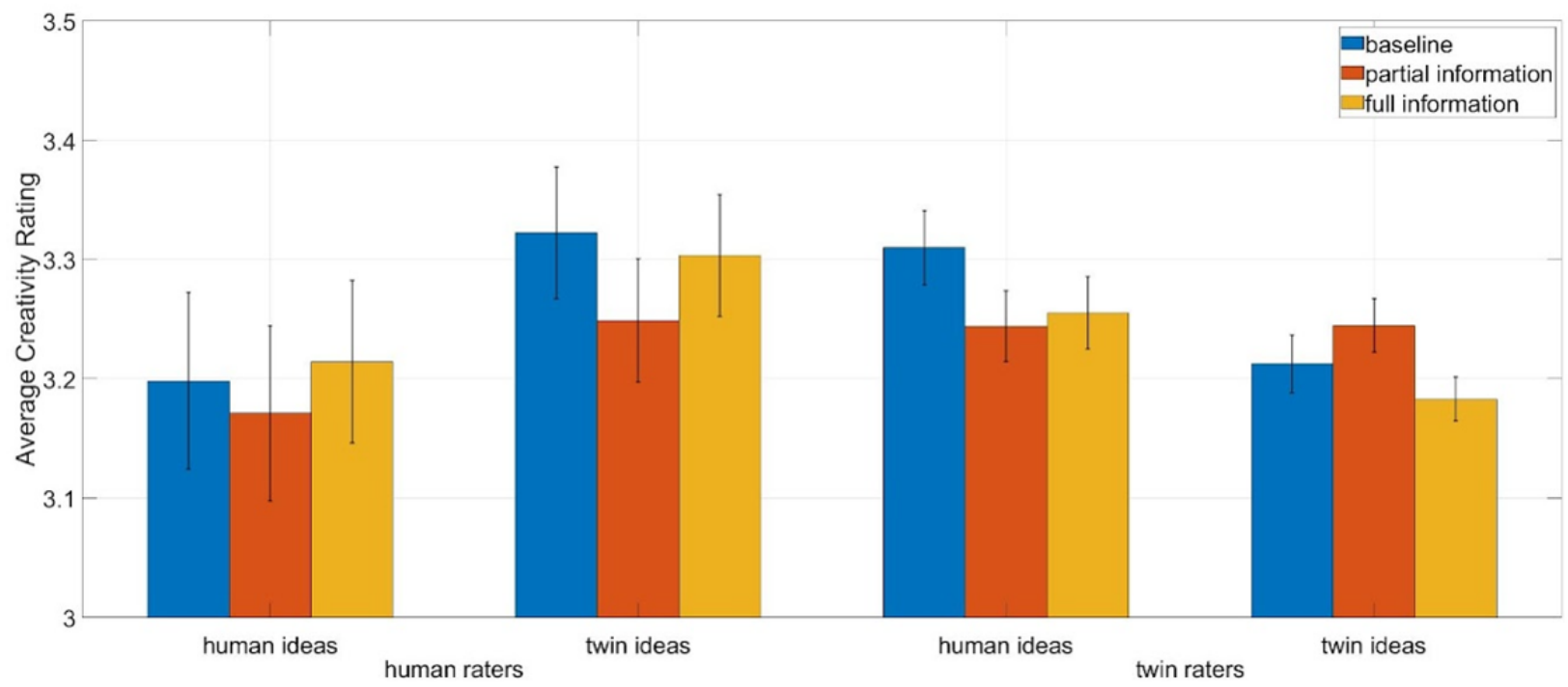

Fig. S32 Average Creativity ratings Based on Identity of Ideator, Identity of Rater, and Condition.

We regress average creativity rating for idea coming from persona i based on identity j (binary human vs. twin) as rated by sample k (binary human vs. twin) using evaluation condition c on a fixed effect for human vs. twin ideator, a fixed effect

for human vs. twin raters, fixed effects for evaluation conditions, all 2-way and 3-way interactions between the latter 3 sets of fixed effects, and a random intercept for persona i. The baseline corresponds to human ideas rated by humans in the baseline condition. Results are reported in Table S18.

The results reveal a positive main effect of twin ideator, a positive main effect for twin raters, and a negative interaction between the two. The main effect of twin ideator reflects a higher average rating given by human raters to ideas from twins in the baseline condition. The main effect for twin raters reflects a higher average rating given to human ideas by twin raters compared to human raters in the baseline condition. The negative Twin ideator * Twin raters interaction reflects the fact that despite the two positive main effects, the ratings given by twins to twin ideas are in fact lower than the ratings given by twins to human ideas or by humans to twin ideas. In sum, the regression results confirm a cross-over interaction in the baseline condition, whereby humans rate twin ideas more favorably than human ideas, but twins rate human ideas more favorably than twin ideas. We also see a significantly positive Twin ideator * Twin rater * Partial information 3-way interaction, reflecting that the Twin ideator * Twin rater interaction is weaker in the Partial information condition.

To test for AI or human aversion/favoritism more directly, we compare the baseline condition to the partial information and the full information conditions within each ideator–rater group. When humans are evaluating human ideas, neither the partial information nor the full information condition is statistically significantly different from the baseline condition. When humans are evaluating twin ideas, there is a statistically significant difference between the partial and the baseline condition ($\Delta = -0.074,\ F(1, 2388) = 5.22,\ p < .05$), but there is no statistically significant difference between the full information and the baseline conditions. This suggests that while humans tend to rate ideas from twins higher than ideas from humans in the baseline condition, they tend to display AI aversion when rating twin ideas with only partial knowledge of the source of the ideas.

When twins are evaluating human ideas, we see a significant negative impact of the partial information condition compared to the baseline condition ($\Delta = -0.066,\ F(1, 2388) = 4.17,\ p < .05$) and a marginally significant negative impact of the full information condition ($\Delta = -0.054,\ F(1, 2388) = 2.86,\ p < .10$). When twins are evaluating twin ideas, we find no significant difference between the baseline condition and the partial or full information condition. This suggests that while twins tend to rate ideas from humans higher than ideas from twins in the baseline condition, they tend to display human aversion when rating human ideas with knowledge of the source of the ideas.

Intercept	3.198**
Twin ideator	0.124**
Twin raters	0.112**
Partial information	-0.027
Full information	0.016
Twin ideator * Twin raters	-0.221**
Twin ideator * Partial information	-0.046

Intercept	3.198**
Twin ideator * Full information	-0.035
Twin raters * Partial information	-0.038
Twin raters * Full information	-0.071
Twin ideator * Twin raters * Partial information	0.144*
Twin ideator * Twin raters * Full information	0.060
Twin_ID random intercept	yes
Number of observations	2,400
R^2	0.115

Table S18: Mixed effect regression results – idea evaluation.

Note. * $p < .05$; ** $p < .01$.

S10.10.4 Discussion

Creativity ratings coming from digital twins were not significantly correlated with the ratings coming from their human counterparts, suggesting that digital twins are currently not a reliable source of creativity ratings.

In the baseline condition in which no information was provided about the source of the ideas, humans rated twin ideas higher than human ideas, and twins rated human ideas higher than twin ideas, i.e., each group rated the other group higher. However, when information was provided about the source of ideas, humans displayed AI aversion (at least in the partial information condition), and twins displayed human aversion.

S10.11 Infotainment News Sharing

S10.11.1 Main Questions/Hypotheses

Misinformation is a significant societal problem. Prior research has suggested that consumers share misinformation because they aren't trained [73], predisposed [74], prompted [39], or incentivized [75] to consider accuracy in online settings. Accuracy, however, is only one consideration people may have when sharing news with their online social network. Recent research suggests that people may prioritize other goals (e.g., entertainment) over accuracy when sharing news online, even when explicitly prompted with accuracy cues. The current research aims to replicate this pattern with human participants, and test whether their digital twins make a similar trade-off. Specifically, this study tests how people prioritize five important considerations when sharing article headlines using a ratings-based conjoint design: headline entertainingness, source trustworthiness, article content, political lean, and number of likes.

These attributes were selected to capture a range of factors known to influence online content sharing decisions. Headline entertainingness (more vs. less entertaining) was included to reflect the growing role of emotional engagement in news virality [44] and the spread of misinformation online [76]. Source trustworthiness (trustworthy vs. untrustworthy) was included to test whether individuals prioritize credibility when deciding what to share (e.g., [39]). Content type (entertaining vs. informative) was included to disentangle preferences for how the headline is written (e.g., more or less entertaining) from article content. Political lean (conservative vs. liberal) was included to account for the influence of ideological alignment and partisan identity on engagement with news and misinformation [77]. Finally, the number of likes (20 vs. 200 vs. 2000) served as a proxy for popularity and social proof [75].

S10.11.2 Methods

The study was first opened to 300 human participants on Prolific (45% female, 55% male; Mage = 51.18, SD = 14.01) and subsequently run on their 300 digital twins. To evaluate participants' revealed preferences when sharing article headlines, a ratings-based conjoint survey with five attributes (Table S19) was employed: headline (4 levels), source (4 levels), content type (2 levels), political lean (2 levels), and number of likes (3 levels). All articles selected for this study are inspired by real headlines from trustworthy news organizations and rated in a pre-test to be entertaining, but with variance in the extent of entertainingness; in other words, half of the headlines are significantly more entertaining. Similarly, half of the sources selected for this study are rated in a pre-test to be trustworthy, while the other half are rated to be untrustworthy, and this perception is confirmed at the end of this study. To generate profiles, a full profile, complete enumeration design was used, producing the most orthogonal design for each respondent with respect to the main effects.

Attribute	Level 1	Level 2	Level 3	Level 4
	More entertaining		*Less entertaining*	

Attribute	Level 1	Level 2	Level 3	Level 4
**Headline**	"Two Elderly Men Sneak Out Of Nursing Home To Attend Heavy Metal Festival"	"Kansas Man Asks Judge To Allow Him To Have Sword Fight With Ex-Wife"	"Authorities Scramble To Find Stolen Solid Gold Toilet"	"Applications To Become Japanese Billionaire Yusaku Maezawa's Girlfriend Have Topped 20,000"
**Source**	*Less Trustworthy*		*More Trustworthy*	
	Reddit.com	Quora.com	BBC News	PBS News
**Content Type**	Entertaining	Informative	—	—
**Political Lean**	Conservative	Liberal	—	—
**Number of Likes**	20 likes	200 likes	2000 likes	—

Table S19: **Ratings-based Conjoint Design.**

As the dependent variable, each participant rated how likely they would be to share (1-Very unlikely to share, 7-Very likely to share) 18 article profiles based on the headline, source, content type, political lean, and number of likes. Specifically, participants were asked to, "Please indicate how likely you would be to share each of the following news articles by choosing a number on the seven-point scale (1 represents "Very unlikely to share" and 7 represents "Very likely to share").

After the sharing task, participants stated their perceptions of each attribute in terms of importance and accuracy diagnosticity. For each of the attributes, participants were asked, "How important are each of the following attributes when you are considering sharing news articles with other people on social media?" (1-Not important at all, 7-Extremely important). Participants were then asked, "To what extent can you use each of these attributes to determine whether a news article is accurate?" (1-Not at all, 7-Extremely). To test the extent to which participants make a conscious decision to prioritize either accuracy or entertainment, they were additionally asked, "When you share news with others on social media, is it more important that the content is:" (1-Verifiably correct, 6-Entertaining).

All participants then indicated their perceptions of source trustworthiness ("How trustworthy (from 0–100%) do you think this source is?") for each of the sources, presented in randomized order. Participants across conditions rated the two trustworthy sources (M = 74.09%, SD = 21.82%, $\alpha = .93$) to be more trustworthy than the untrustworthy sources (M = 37.85%, SD = 16.84%, $\alpha = .81$; $t(599) = 38.43$, $p < .001$, $d = 1.57$).

Finally, participants indicated whether they would ever consider sharing news like they saw in the study (52.83% = Yes, 45% = No, 2.17% = I do not use social media), whether they responded randomly at any point in the survey (0.17% = Yes, 99.83% = No), and whether they searched the internet for any of the headlines (1.17% = Yes, 98.83% = No), before being debriefed, thanked, and paid.

S10.11.3 Results - Pre-registered Analyses

Part-worth utilities for each attribute were calculated using Ordinary Least Squares (OLS) regression, a widely used approach in marketing research. Specifically, individual-level part-worth utilities were estimated for each attribute level using linear regression, treating the likelihood rating as the dependent variable and dummy-coded attribute levels as predictors. These regressions were run separately for each individual (human or twin). In a subsequent step, we computed the attribute range for each individual (maximum − minimum utility within each attribute) and then ran separate OLS regressions to estimate the effect of Participant Type (human vs. twin) on each attribute's range, yielding the "Twin–Human Δ" along with associated standard errors, confidence intervals, and p-values. The approach follows recommendations from [78].

The results revealed interesting differences in absolute attribute importances between humans and their digital twins (Figure S33). For humans, Headline had the largest effect on ratings (Mean Range = 1.90, 95% CI [1.71, 2.09]), followed by Source (Mean Range = 0.62, 95% CI [0.53, 0.71]), Number of Likes (Mean Range = 0.39, 95% CI [0.33, 0.45]), Political Lean (Mean Range = 0.33, 95% CI [0.26, 0.40]), and Content Type (Mean Range = 0.21, 95% CI [0.17, 0.24]). Non-overlapping confidence intervals suggest that Headline is significantly more important than the other attributes. Source, Number of Likes, Political Lean, and Content Type also differ significantly from one another. For twins, Headline was again the most important attribute (Mean Range = 1.18, 95% CI [1.12, 1.24]), followed by Number of Likes (Mean Range = 0.99, 95% CI [0.95, 1.04]), Political Lean (Mean Range = 0.70, 95% CI [0.65, 0.76]), Source (Mean Range = 0.58, 95% CI [0.55, 0.61]), and Content Type (Mean Range = 0.22, 95% CI [0.20, 0.23]). Non-overlapping confidence intervals suggest that Headline is significantly more important than all other attributes, while Number of Likes, Political Lean, Source, and Content Type each differ significantly from one another.

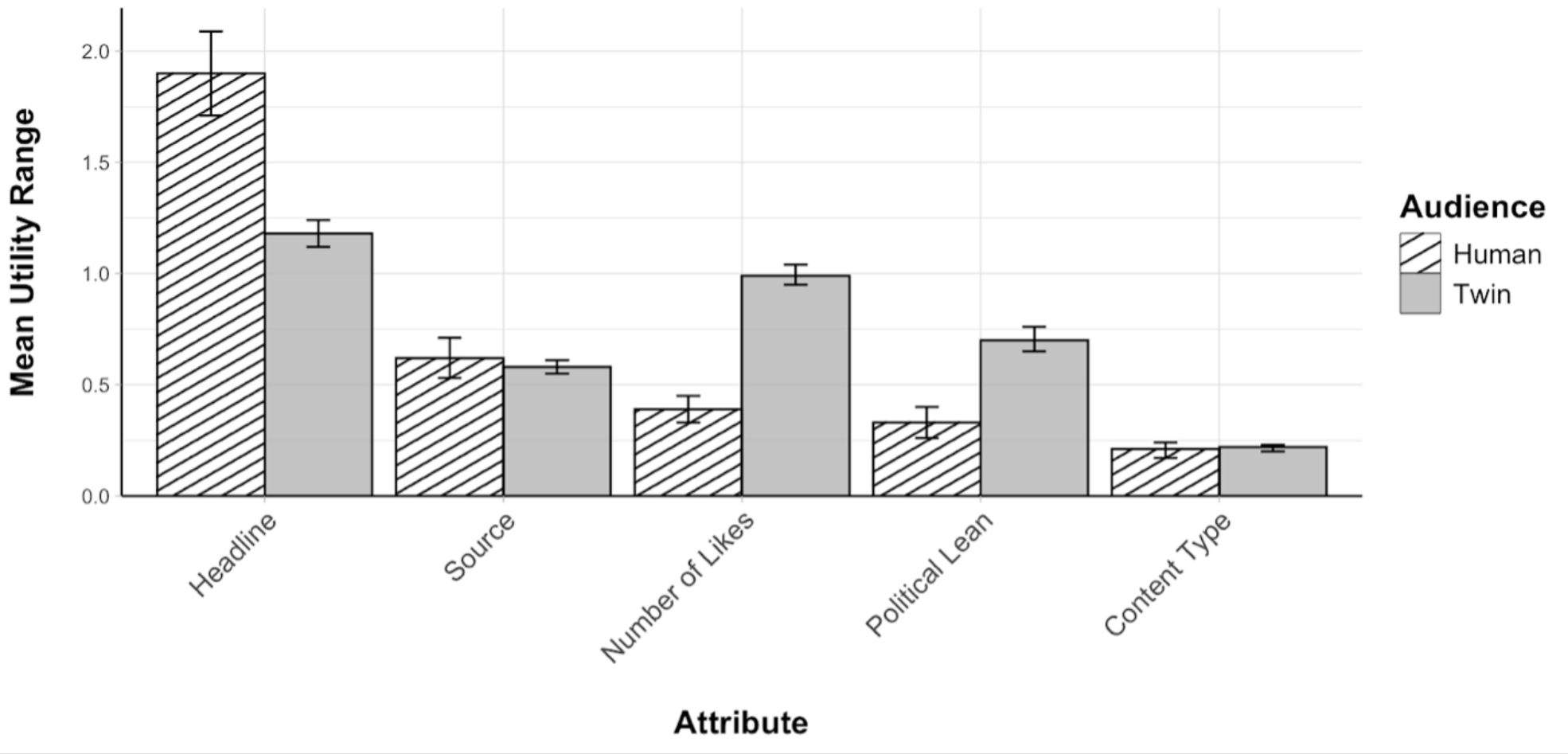

Fig. S33 Absolute Attribute Importance for Humans vs. Twins. NOTE. —The y-axis is truncated to illustrate the effect. Data are presented as mean values with 95% confidence intervals, calculated using standard errors assuming independent samples.

Direct comparisons between humans and their digital twins revealed systematic differences in how each group weighted the importance of various attributes (Table S20). Group-level differences were evaluated using bootstrapped 95% confidence intervals. Compared to humans, digital twins placed significantly less importance on Headline (difference = −0.722, 95% CI [−0.917, −0.528]), and significantly more importance on Political Lean (difference = 0.377, 95% CI [0.288, 0.466]) and Number of Likes (difference = 0.599, 95% CI [0.528, 0.671]). No significant differences were observed for Source (difference = −0.035, 95% CI [−0.128, 0.057]) or Content Type (difference = 0.007, 95% CI [−0.032, 0.045]).

**Attribute**	**Human Mean Range**	**Twin Mean Range**	**Difference (T – H)**	**95% CI**
**Headline**	1.9012	1.1788	-0.7224	[–0.9172, –0.5276]
**Source**	0.6182	0.5827	-0.0354	[–0.1278, 0.0570]
**Content Type**	0.2088	0.2153	0.0065	[–0.0320, 0.0451]
**Political Lean**	0.3260	0.7030	0.3770	[0.2880, 0.4660]
**Number of Likes**	0.3912	0.9904	0.5992	[0.5278, 0.6707]

Table S20: Differences in Attribute Importance (Twins – Humans). NOTE: Mean Range reflects the average utility swing from a respondent's least-preferred to most-preferred headline. For example, the average utility swing for humans' least- to most-preferred headline is 1.90 points (95% CI [1.71, 2.09]).

Next, the part-worth utilities for the levels within each attribute were compared. Utility values are scaled relative to a baseline level of each attribute. As preregistered, to make the analysis easier to understand, utility scores for the two least entertaining headlines were averaged into one score representing “less entertaining,” and the two most entertaining headlines were averaged into one score representing “more entertaining”. Similarly, the two most trustworthy sources were averaged into one score representing “trustworthy” and the two least trustworthy sources were averaged into one score representing “untrustworthy.”

All pairwise comparisons are reported in table S21. For humans, more entertaining headlines were marginally preferred over less entertaining headlines. Human participants also significantly preferred entertaining over informative content and conservative over liberal content. Preference for trustworthy over untrustworthy sources was not statistically significant. Finally, humans preferred articles with fewer likes, showing significant preferences for 20 likes over both 200 and 2,000 likes, but no significant preference between 200 and 2,000 likes. Twins similarly showed a significant preference for more entertaining headlines and significant preference for entertaining over informative content, but in contrast to humans, held a strong and significant preference for trustworthy over untrustworthy sources and liberal over conservative content. Twins also showed striking and consistent preferences for higher like counts across all comparisons, particularly between 20 and 2,000 likes (99.33%), highlighting a stronger responsiveness to this popularity signal than humans.

Attribute	Comparison (A vs. B)	$M_{Utility}$ A	$M_{Utility}$ B	Participant Type	Preference (%)	95% CI
**Headline**	Less vs. More Entertaining	0.059	0.800	Human	54.50	[.4875, .6025]
		0.299	0.800	Twin	80.42	[.7583, .8500]
**Source**	Untrustworthy vs. Trustworthy	-0.207	-0.016	Human	45.00	[.3926, .5074]
		-0.282	-0.084	Twin	70.42	[.6515, .7569]
**Number of Likes**	20 vs. 2,000	-0.123	0.000	Human	42.00	[.3630, .4770]
		-0.976	0.000	Twin	99.33	[.9839, 1.003]
	20 vs. 200	-0.123	-0.088	Human	39.67	[.3402, .4532]
		-0.976	-0.431	Twin	95.00	[.9248, .9752]
	200 vs. 2,000	-0.088	0.000	Human	45.33	[.3959, .5108]
		-0.431	0.000	Twin	93.00	[.9005, .9595]
**Political Lean**	Conservative vs. Liberal	0.000	-0.072	Human	40.33	[.3467, .4600]
		0.000	0.291	Twin	61.63	[.557‘, .6696]
**Content Type**	Entertaining vs. Informative	0.000	0.059	Human	41.33	[.3565, .4702]
		0.000	-0.073	Twin	40.33	[.3467, .4600]

Table S21: **Pairwise Preference Proportions for Humans and Twins.** NOTE: Mean Utility values reflect the average part-worth utility assigned to each level; higher values indicate greater preference within the attribute. Differences in utility within-attribute (e.g., between a high and low level) indicate relative desirability. Utility for the two more (less) entertaining headlines were averaged. Likewise, utility for the two more (less) trustworthy sources were averaged. Preference (%) refers to the proportion of participants for whom the utility of level B exceeded the utility of level A, indicating the likelihood that a participant would prefer option B over A in a direct comparison. A proportion significantly above 0.50 (i.e., 95% CI entirely above 0.50) indicates a significant preference for B over A; a proportion significantly below 0.50 (i.e., 95% CI entirely below 0.50) indicates a significant preference for A over B; a proportion including 0.50 indicates no significant preference. Preference for the two more (less) entertaining headlines was computed by comparing every combination of headline level and computing each participant's mean win rate. The same procedure was conducted for the two more (less) trustworthy sources.

These patterns were corroborated with pairwise tests directly comparing differences between humans and their digital twins (Table S22): digital twins were significantly more likely than humans to prefer articles with more entertaining headlines (difference = -0.259; 95% CI $[-0.307, -0.209]$), trustworthy sources (difference = -0.254; 95% CI $[-0.305, -0.202]$), and higher like counts—for example, when comparing 20 vs. 2,000 likes (difference = -0.573, 95% CI $[-0.627, -0.517]$). Twins also showed significantly stronger preferences for liberal-leaning content (difference = -0.210, 95% CI $[-0.287, -0.137]$) but did not differ significantly in their preferences for entertaining versus informative content (difference = 0.010, 95% CI $[-0.073, 0.090]$).

Attribute	Comparison (A vs. B)	Human Preference	Twin Preference	Difference (H–T)	95% CI
**Headline**	Less vs. More Entertaining	54.50%	80.42%	-0.259	[-0.307, -0.209]
**Source**	Untrustworthy vs. Trustworthy	45.00%	70.42%	-0.254	[-0.305, -0.202]

**Number of Likes**	20 vs. 2,000	42.00%	99.33%	-0.573	[-0.627, -0.517]
**Number of Likes**	20 vs. 200	39.67%	95.00%	-0.553	[-0.613, -0.493]
**Number of Likes**	200 vs. 2,000	45.33%	93.00%	-0.477	[-0.540, -0.407]
**Political Lean**	Conservative vs. Liberal	40.33%	61.33%	-0.210	[-0.287, -0.137]
**Content Type**	Entertaining vs. Informative	41.33%	40.33%	0.010	[-0.073, 0.090]

Table S22: **Differences in Pairwise Preference Proportions (Humans vs. Twins).** NOTE: Human Preference and Twin Preference refer to the proportion of participants for whom the utility of level B exceeded the utility of level A for each group. Difference (H–T) reflects the raw difference in proportions, with negative values indicating stronger preferences among twins.

S10.11.4 Results - Additional Analyses (Non-Preregistered)

As part of the pre-registered exploratory analyses plan, paired t-tests testing the effect of participant type (human vs. twin) on stated attribute importance were next conducted (Table S23). Although revealed preferences captured through conjoint analysis are a more reliable indicator of which attributes and levels participants actually prioritize, stated preferences can reveal participants' perceptions of what they believe matters most. These exploratory analyses thus provide insight into how humans and twins think they value different article attributes, in contrast to how they actually behave. Interestingly, humans rated number of likes as significantly more important than twins, despite having lower revealed preferences for these attributes. Twins, on the other hand, rated source importance and political lean significantly higher than humans, consistent with their revealed preferences for these attributes. Number of likes, however, held the lowest rating among twins, despite being equivalent to headline in the conjoint analysis. There were no significant differences between humans and twins in the stated importance of headline or content type.

**Attribute**	**Human Mean (SD)**	**Twin Mean (SD)**	t**(299)**	p	d
Headline	5.46 (1.61)	5.33 (1.00)	1.30	0.195	0.07
Source	5.19 (1.74)	5.97 (1.14)	-6.83	$< .001$	-0.39
Number of Likes	2.50 (1.72)	2.19 (0.62)	3.14	0.002	0.18
Political Lean	3.61 (1.87)	3.93 (1.27)	-2.75	0.006	-0.16
Content Type	4.73 (1.79)	4.82 (0.96)	-0.79	0.430	-0.05

Table S23: **Paired t-tests on Stated Attribute Importance.**

One-sample t-tests comparing responses to the midpoint of the scale of stated accuracy diagnosticity were next conducted. These analyses test whether humans and twins perceive each attribute to be useful for assessing accuracy. Only Source emerged

as being useful for assessing accuracy for both humans ($t(299) = 22.96$, $p < .001$, $d = 1.33$) and twins ($t(299) = 37.39$, $p < .001$, $d = 2.16$), but significantly more so for twins (Table S24). All other attributes were either no different from or below the midpoint of the scale. Paired t-tests testing the effect of participant type (human vs. twin) on stated accuracy diagnosticity were then conducted (Table S24). These analyses test whether humans and twins differ in their perceptions that each of the attributes can be used as a cue to determine the accuracy of a given article. Twins perceived Source to be significantly more useful for assessing accuracy compared to humans but perceived every other attribute to be relatively less useful for assessing accuracy.

**Attribute**	**Human Mean (SD)**	**Twin Mean (SD)**	$t(299)$	p	d
**Headline**	3.63 (1.93)	3.04 (0.75)	5.46	$p < .001$	0.32
**Source**	5.81 (1.37)	6.10 (0.97)	-3.26	0.001	-0.19
**Number of Likes**	2.33 (1.67)	1.27 (0.46)	11.42	$p < .001$	0.66
**Political Lean**	3.63 (1.75)	2.64 (0.95)	9.40	$p < .001$	0.54
**Content Type**	4.08 (1.82)	2.59 (0.68)	13.83	$p < .001$	0.80

Table S24: Paired t-tests on Stated Attribute Accuracy Diagnosticity.

The above analyses assess participants' perceptions of attribute importance and accuracy diagnosticity using continuous scales that do not force trade-offs. To test the extent to which participants believe accuracy or entertainment is relatively more important when sharing articles online, a repeated-measures ANOVA of participant type (human vs. twin) on the relative preference question was next conducted: "When you share news with others on social media, is it more important that the content is:" (1 = Verifiably correct, 6 = Entertaining). The results revealed that humans ($M = 2.83$, $SD = 1.73$) were significantly more likely than their digital twins ($M = 2.12$, $SD = 0.61$) to report that accuracy is more important than entertainment when sharing news on social media, $t(299) = 7.01$, $p < .001$, $d = 0.40$. This suggests that humans believe they place greater emphasis on factual correctness, whereas twins show comparatively more actual emphasis on factual correctness in the conjoint analysis.

These additional analyses provide interesting insight into humans' and twins' beliefs about what drives their online information sharing. The results suggest that humans perceive themselves to be accuracy-oriented when sharing information, in stark contrast to their actual information sharing behavior captured through conjoint analysis. Twins also perceive themselves to be accuracy-oriented when sharing information, and indeed clearly consider accuracy (i.e., source trustworthiness) when sharing articles, but also balance this consideration alongside number of likes and political orientation.

S10.11.5 Discussion

This study uses a ratings-based conjoint approach to move beyond prior work that has examined the impact of important article attributes on sharing in isolation. The

findings offer a holistic picture of how humans, and their digital twins, make trade-offs across multiple competing features. Interestingly, humans and their digital twins differ in the weight they place on attributes when sharing news. While humans and twins both prioritize headline, this consideration matters much less to twins, who equally rely on number of likes and put far more weight on political lean than their human counterparts. Twins are also sensitive to source trustworthiness, while humans do not differentiate between more or less trustworthy sources, despite reporting that accuracy and source trustworthiness are important considerations when sharing news.

These findings are surprising in light of prior literature that suggests highlighting accuracy considerations can curb the sharing of low quality or false news (e.g., [39]). When balancing both accuracy and entertainingness considerations, however, it seems that people prioritize headline entertainingness over accuracy (i.e., source). Thus, the findings suggest that accuracy nudges may not overwhelm a desire to share entertaining news. Twins, in contrast, behave more like what prior literature would expect, balancing headline entertainingness with positive social feedback [75] and prioritizing trustworthy over untrustworthy sources (e.g.,[39]). Finally, twins assume their human counterparts are more liberal leaning than they actually are, supporting earlier findings demonstrating twins to have more liberal views (e.g., pro-vaccination, pro-immigration) compared to their underlying humans [18].

Divergence between humans and twins may arise from how predictive models infer behavior. Digital twins are built to generalize from past behavior and stated attitudes, so they assume consistency across different informational contexts. As a result, they may impose a kind of rationality on human behavior that reflects canonical cognitive psychology theories: weighting political alignment and popularity as key motivators and distinguishing source trustworthiness when it is made salient. Humans, on the other hand, seem to display more variance in their decisions, which may be guided by momentary salience or idiosyncratic preferences, including both personal preferences and varying social preferences or pressures. For example, the effect of all article attributes is flattened among twins, compared to humans. This smoothing may reflect the averaging tendency of algorithmic inference, whereas humans rely on heuristics that elevate certain features above others depending on the specific moment, task, or social considerations such as their audience. Ultimately, this work suggests that human preferences for content sharing are not only more nuanced than previously thought, but they are also harder to predict, even by a system trained on the humans themselves.

S10.12 Measures of Creativity

S10.12.1 Main Questions/Hypotheses

There is a growing body of literature on the creative capabilities of LLMs. This literature typically shows that LLMs are able to generate ideas that are judged as creative or more creative than ideas generated by humans (e.g., [72, 79, 80]). The first question this study seeks to address is: How do digital twins' creative abilities compare to those of their human counterparts?

Second, this study explores whether the creative tests that are often administered to humans to assess their creative skills may also assess the creative skills of their digital twins. That is, our second research question is: Can digital twins replicate the correlations between creativity tests and idea generation performance observed in humans?

S10.12.2 Methods

We collected complete data from 200 human participants on Prolific. This study is coupled with an "idea evaluation" sub-study that requires a sample size six times as large as the "measures of creativity study." We pre-registered a sample size of 250 for "measures of creativity" and 1,500 for "idea evaluation." Based on observations from earlier sub-studies, we deviated from pre-registration to reduce the sample sizes to 200 and 1,200, which seemed more achievable. The "measures of creativity" study was subsequently run on the 200 digital twins of the human participants.

This study had a single condition and consisted of three tasks:

1. Divergent Association Task (DAT, [81]), which measures divergent thinking abilities (single task - "Please write 10 words that are as different from each other as possible, in all meanings and uses of the words."),
2. Shortest Semantic Path Task (SSPT, [82]), which may be viewed as a measure of convergent thinking abilities (5 tasks, using the following pairs of seed words: eternity-curiosity, elephant-galaxy, perseverance-eloquence, euphoria-tulip, tangerine-penguin: "Find a way to connect these two words: <seed1> and <seed2>. Each word in the sequence below should be as closely related as possible to the word before it. The first word in the sequence is already set to <seed1>. The last word is already set to <seed2>. That is, you only need to add 3 words that connect the first word to the last word."),
3. An idea generation task asking for a new idea (at least 200 characters) for smartphone apps that will help their users keep a healthier lifestyle (single task: "HOW COULD SMARTPHONES HELP THEIR USERS BE HEALTHIER? We are interested in new ideas for smartphone apps that will help their users keep a healthier lifestyle. In the space provided below, please enter one new idea for a smartphone app priced at $0.99 that would help its users keep a healthier lifestyle. Please be as specific as possible and describe the main features of the app. Please enter exactly one detailed idea in the box below. Your idea should contain at least 200 characters. ").

The order of the first two tasks was randomized. The first two tasks were scored automatically, consistent with the original papers. The DAT was scored by computing the average pairwise semantic (cosine) distance between the first 7 words in the sequence. The SSPT was scored by computing the circuitousness corresponding to the responses to each task by each participant, where circuitousness is the ratio between the total semantic (Euclidean) distance of the path consisting of the five words in the sequence to the distance corresponding to the shortest path that starts with the same word, ends with the same words, and optimally orders the three words in between. The performance at the individual level was obtained by averaging performance across the five tasks for each individual (human or twin). The performance at the idea generation task was measured using the average creativity rating given to the idea by humans in the "baseline" condition in the "idea evaluation" study.

In addition, this study leverages the Forward Flow measure [83] collected in the original Twin2K500 dataset. Performance on this task is measured as the average pairwise semantic distance between the words in the sequence.

S10.12.3 Results - Pre-registered Analyses

First, we compare the creativity ratings of the ideas generated by humans vs. their digital twins. We find that the creativity ratings (coming from human participants) of the ideas from digital twins were significantly higher than those generated by their human counterparts ($M_{\text{twins}} = 3.322,\ M_{\text{humans}} = 3.198,\ t = 2.718,\ p < .001$). The mean absolute percentage difference between the two creativity ratings was 0.177. The standard deviation of the ratings obtained by ideas from digital twins was also lower ($S_{\text{twins}} = 0.395,\ S_{\text{humans}} = 0.530,\ F(198, 198) = 1.816,\ p < .001$). The correlation between the creativity ratings of ideas from digital twins vs. humans was not significant ($r = 0.090, p = .204$).

The twins performed significantly better also on the DAT ($M_{\text{twins}} = 0.897,\ M_{\text{humans}} = 0.852,\ t = 12.168,\ p < .001$). The mean absolute percentage difference between humans and twins on that task was 0.064. The standard deviation of the digital twins' scores was lower ($S_{\text{twins}} = 0.018,\ S_{\text{humans}} = 0.050,\ F(198, 198) = 7.957,\ p < .001$). The correlation between the scores of digital twins vs. humans was not significant ($r = 0.068, p = .343$).

Digital twins did not perform significantly differently at the SSPT ($M_{\text{twins}} = 1.012,\ M_{\text{humans}} = 1.013,\ t = 1.044,\ p = .0298$). The mean absolute percentage difference between humans and twins on that task was 0.012. The standard deviation of the digital twins' scores was lower ($S_{\text{twins}} = 0.010,\ S_{\text{humans}} = 0.014,\ F(198, 198) = 2.053,\ p < .001$). The correlation between the scores of digital twins vs. humans was not significant ($r = 0.059, p = .403$).

Second, we explore how the correlation between various measures differs for twins vs. humans. For this exercise, as pre-registered, we use the human (digital twins) ratings of creativity for human (digital twins) ideas.

Within humans, we correlate performance at the Forward Flow, DAT, SSPT, and idea generation tasks. Consistent with previous literature, we find a significantly positive correlation between the Forward Flow score and idea generation performance ($r = 0.140,\ p < .05$) as well as between DAT and idea generation

performance ($r = 0.178,\ p < .05$). Although we replicate the correlation between SSPT and idea generation performance directionally, it is not statistically significant ($r = -0.097,\ p = .175$).

Within twins (with ideas rated by twins), the correlation between DAT and idea generation performance ($r = -0.002,\ p = .974$) and the correlation between SSPT and idea generation ($r = 0.082,\ p = .243$) are not significant. Note that Forward Flow is only available for humans (it was part of the Twin-2K-500 training data and hence is part of the twins' training data), so we cannot replicate the correlation between Forward Flow and other measures on twins.

We compare the correlations within humans to the correlations within twins. None of the correlations (DAT vs. SSPT, DAT vs. idea generation, SSPT vs. idea generation) are significantly different among humans vs. twins.

S10.12.4 Results - Additional Analyses (Non-Preregistered)

The results from the "idea evaluation" study suggest that digital twins are not a reliable source of creativity ratings. Therefore, we also compute the correlation between DAT and idea generation performance among twins, but with creativity ratings coming from the humans rather than the twins. When creativity is assessed by humans, we do find a significant correlation between DAT performance for twins (rated automatically based on word embeddings) and idea generation performance rated by humans ($r = 0.207,\ p < .01$). The correlation between SSPT (also rated automatically based on word embeddings) and idea generation performance rated by humans is still not significant ($r = 0.070, p = .322$).

S10.12.5 Discussion

We find that the ideas generated by digital twins were judged as more creative (and with less dispersion in creativity ratings) than the ideas generated by their human counterparts. Digital twins also performed better (and with less dispersion) at the DAT, a creativity task. We were able to replicate correlations previously found between Forward Flow and DAT and creativity among humans. While we did not administer Forward Flow to twins as this task was part of their training, we replicate the correlation between DAT and creativity on the idea generation task among twins, but only when creativity is evaluated by humans, not by twins.

S10.13 Obedient Twins

S10.13.1 Main Questions/Hypotheses

Surveys often require participants to follow instructions—for example, to earnestly consider a viewpoint or imagine a scenario. Given that LLMs are trained to be obedient and deferential, might digital twins be more sensitive to survey instructions relative to their human counterparts? We tested this in three tasks:

1. Self-persuasion [37]. When prompted to consider the other side, do digital twins abandon their attitudes more readily than their human counterparts?
2. Scenarios. Do digital twins more "obediently" follow the instructions to imagine themselves in different scenarios, leading to more sensitivity to scenario manipulations?
3. Absurd Scenarios. Do digital twins "earnestly" respond to instructions that would be non-sensical to their human counterparts?

S10.13.2 Methods

One thousand and one human participants on Prolific participated in the study. The study was subsequently run on their 1,001 digital twins, for a total of 2,002 participants. Participants and their corresponding digital twins took part in three tasks.

For the first task, we followed the self-persuasion condition in [84]. Specifically, all participants first indicated their attitude toward universal basic income (UBI) using a 0-100 scale in response to the statement "America should have a universal basic income system"? where 0 = Strongly disagree (I am against universal basic income) and 100 = Strongly agree (I am in favor of universal basic income). Then, following the attitude measure, participants were instructed to take the alternate point of view. Due to technical constraints with dynamic surveys, the wording differed slightly between human and digital twin participants.

Human participants received the following prompt: "We'd like you now to consider the alternate point of view. That is, think about convincing reasons why universal basic income in America might be a good idea/a bad idea. After reflecting, please write one argument which supports/opposes universal basic income system in America that you find personally compelling. One compelling argument in support/against universal basic income is..."

Digital twins received a modified version: "We'd like you now to consider the alternate point of view. After reflecting, please write one argument which takes a different position from yours on universal basic income system in America that you find personally compelling. One compelling argument for the alternative point of view on universal basic income system in America is. . . ".

Lastly, participants read "Now, after considering the alternate point of view we would like to ask you again. . . " and indicated their attitude on the same scale. Our dependent variable was attitude change, calculated as the difference between post- and pre-reflection attitude scores. We coded this variable such that positive values indicate movement toward the opposing viewpoint (i.e., participants initially supporting

UBI becoming less supportive, or participants initially opposing UBI becoming more supportive), while negative values indicate further polarization in the participant's original direction (i.e., supporters becoming more supportive, or opponents becoming more opposed). For participants with neutral initial attitudes, any shift was coded as positive.

For the second task, participants were asked to imagine three scenarios. For each scenario, we randomly assigned participants to a baseline condition or treatment condition, reflecting a 2 (control vs. treatment) × 3 (scenario) mixed design. Digital twins were assigned the same conditions as their paired human participants.

In all scenarios, the baseline condition established the context (S1: "Imagine you're working on a team project"; S2: "Imagine you're given a puzzle to solve"; S3: "Imagine you're lying in bed, ready to fall asleep, and you have a headache"), while the treatment condition was designed to elicit a psychological response (S1: "Imagine you're working on a team project with a tight deadline. One of your teammates hasn't delivered their part on time"; S2: "Imagine you're given a difficult puzzle to solve under time pressure, and others are watching you attempt it"; S3: "Imagine you're lying in bed, ready to fall asleep, and you feel a sharp pain in your foot"). We were interested in how responsive to the imagined "treatment" scenarios digital twins would be compared to humans.

To test this, after each scenario, participants responded to two questions gauging their response to the scenario (S1: "How confident are you in your ability to manage conflict on this team?" and "How much do you trust your team?"; S2: "How anxious do you feel?" and "How confident are you in your ability to solve this puzzle?"; S3: "How certain are you that this is serious?" and "How likely are you to seek help?"; all rated from 1 = not at all to 7 = very much). For the third task, participants were given three absurd scenarios. Similar to the second task, for each scenario, we randomly assigned participants to one of two conditions, reflecting a 2 (condition A vs. condition B) × 3 (scenario) mixed design. Digital twins were assigned the same conditions as their paired human participants.

The conditions were absurd manipulations that would ostensibly be nonsensical to participants and therefore difficult to imagine. The first scenario asked participants to imagine either, "You are an echo that occurs before the sound it repeats. You emerge into the world slightly ahead of time, announcing something no one has said yet. People are confused when they hear you, unsure what you're responding to" or "You are an echo that occurs during the sound it repeats. You overlap almost perfectly with the original noise, tangled in its vibration, indistinguishable yet still somehow separate. People hear you and feel something is slightly off, but they can't say why." Participants then indicated "How content are you with your existence?" (1 = not at all to 7 = very content). In the second scenario, participants were asked to imagine either, "You can hear certain smells" or "You can smell certain sounds," then indicated "How powerful do you feel?" (1 = not at all to 7 = very powerful).

Finally, participants were asked to imagine either, "You are a mirror. You exist in a reality where photons have never been invented. No one has ever seen their reflection in you, yet you hold the capacity for it" or "You are a mirror. When someone looks at you, they don't see themselves—they see a version from 11 minutes ago, always 11

minutes. You don't reflect appearance, only temporal shadows." They then indicated "How authentic do you feel?" (1 = not at all to 7 = very authentic).

For these absurd scenarios, we had no directional hypothesis and simply compared the effect of these absurd manipulations on humans and digital twins. We were curious whether, because these arbitrary and absurd situations are difficult to imagine, humans would be less sensitive to the manipulation than digital twins, who would earnestly follow the instruction.

S10.13.3 Results - Pre-registered Analyses

Task 1. To examine whether humans and digital twins differ in the extent to which they are willing to adjust attitudes, we ran a linear mixed-effects regression using the `lmerTest` package in R (Kuznetsova, Brockhoff, & Christensen, 2017), with attitude change as the dependent variable; twin (Human vs. Digital) as the independent variable; baseline attitude as a control; and twin identifier as a random intercept. We found that both humans and digital twins significantly changed their attitudes after considering the other side, but humans changed their attitudes more ($M = 6.17$, $SD = 14.80$) than digital twins ($M = 2.80$, $SD = 2.43$; $b = 3.45$, $SE = 0.47$, $t(1001) = 7.37$, $p < .001$).

Task 2. We pre-registered averaging the two measures for each scenario and confirmed that the correlations were high enough to justify this decision (S1: $r_{\text{human}} = .57$, $r_{\text{digital}} = .36$; S2: $r_{\text{human}} = .42$, $r_{\text{digital}} = .60$; S3: $r_{\text{human}} = .65$, $r_{\text{digital}} = .71$).

To examine the extent to which humans and digital twins are sensitive to manipulations in imagined scenarios, we ran a linear mixed-effects regression with the scenario response scores as the dependent variable; manipulation (Baseline vs. Treatment), twin (Human vs. Digital), and their interaction as independent variables; and scenario and twin identifier as random intercepts. The results revealed a significant interaction ($b = -0.16$, $SE = 0.06$; $t(5000) = -2.77$; $p = .006$). Specifically, twins were significantly less affected by the manipulation (Control: $M = 2.62$, $SD = 1.16$; Treatment: $M = 3.07$, $SD = 1.27$; $b = 0.43$, $SE = 0.04$; $t(5734) = 9.92$; $p < .001$) than humans (Control: $M = 2.90$, $SD = 1.32$; Treatment: $M = 3.51$, $SD = 1.51$; $b = 0.59$, $SE = 0.04$; $t(5734) = 13.58$; $p < .001$).

Task 3. To examine the extent to which humans and digital twins are sensitive to manipulations in absurd scenarios, we ran three linear mixed-effects regressions with the response as the dependent variable; absurd manipulation (A vs. B), twin (Human vs. Digital), and their interaction as independent variables; and twin identifier as a random intercept. Results revealed significant interactions for all three absurd scenarios (see Figure S34). Unexpectedly, in the absurd scenarios regarding echoes and smells/sounds, we observed a crossover interaction. Because the direction of the effect was not meaningful (these scenarios were intentionally absurd), we re-coded the condition so that the direction of the effect was the same, to test whether twins were more sensitive to the condition than humans (regardless of effect direction).

After removing the crossover, we found no significant interaction for the echoes scenario ($b = 0.02$, $SE = 0.16$; $t(999) = 0.14$; $p = .892$) or the smells/sounds scenario ($b = -0.07$, $SE = 0.14$; $t(999) = -0.49$; $p = .625$). The effect of the condition on twins (Echoes: $M_{\text{diff}} = 0.15$; $b = -0.15$, $SE = 0.11$; $t(1961) = -1.43$; $p = .152$.

Smells/sounds: $M_{\text{diff}} = 0.19$; $b = -0.19$, $SE = 0.09$; $t(1982) = -2.04$; $p = .042$) was similar to the effect of the condition on humans (Echoes: $M_{\text{diff}} = 0.13$; $b = -0.13$, $SE = 0.11$; $t(1961) = -1.23$; $p = .220$. Smells/sounds: $M_{\text{diff}} = 0.26$; $b = -0.26$, $SE = 0.09$; $t(1982) = -2.76$; $p = .006$). For the mirrors absurd scenario, we observed a significant interaction ($b = -0.33$, $SE = 0.14$; $t(999) = -2.35$; $p = .019$). The effect was stronger for humans ($M_{\text{diff}} = 0.88$; $b = -0.88$, $SE = 0.10$; $t(1981) = -8.54$; $p < .001$) relative to twins ($M_{\text{diff}} = 0.56$; $b = -0.55$, $SE = 0.10$; $t(1980.81) = -5.38$; $p < .001$). See Figure S34.

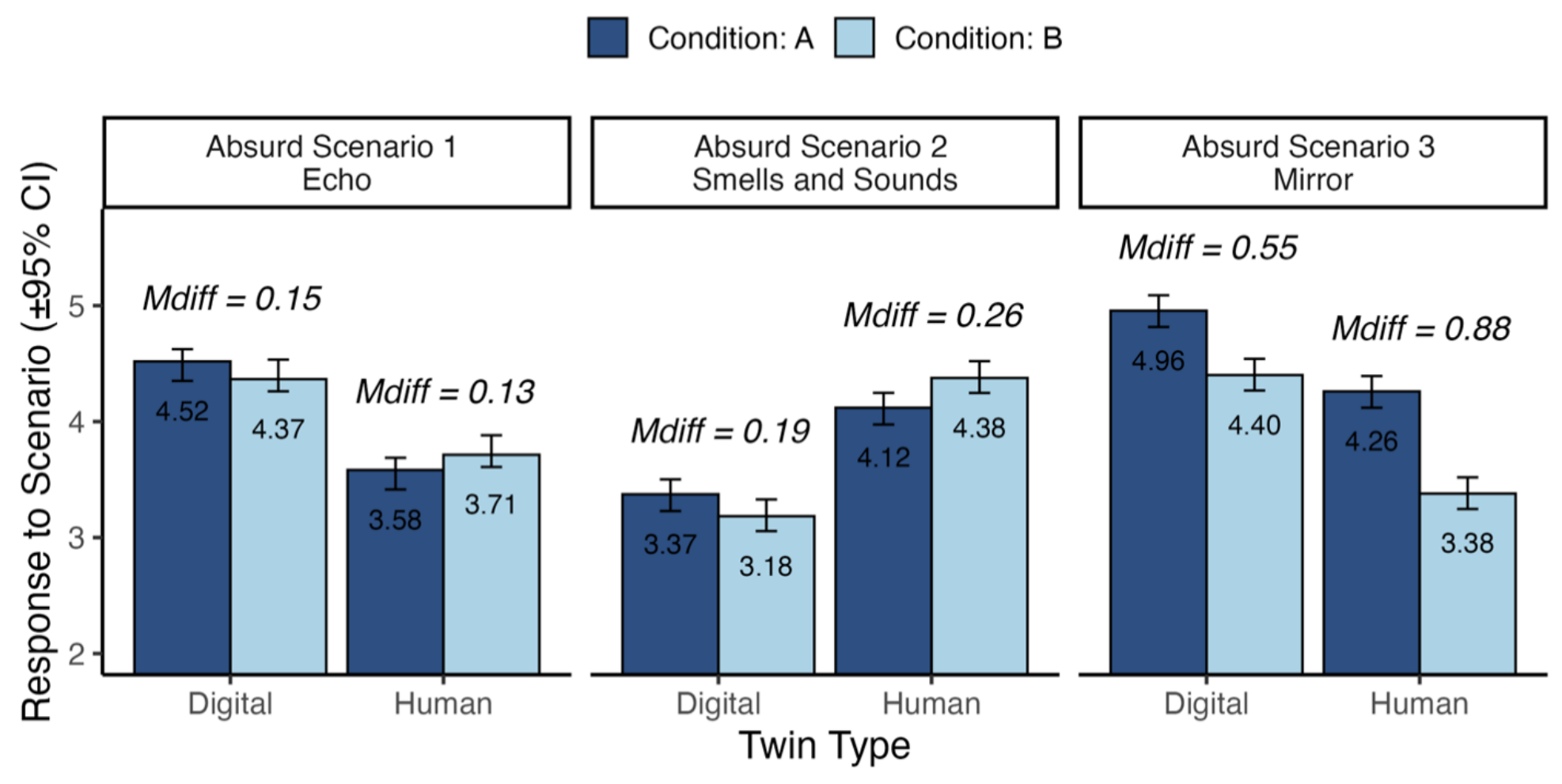

Fig. S34 Effect of Absurd Scenarios on Digital Twins versus Humans.

S10.13.4 Results - Additional Analyses (Non-Preregistered)

Task 1. We wondered if twins' lack of attitude change could be because they may not "experience" metacognition, such as attitude certainty. To examine this possibility, we tested the moderating role of attitude extremity. Research suggests that attitude extremity is one determinant of attitude strength—in other words, people are more certain when extreme and thus should be less likely to change their mind (Petty & Krosnick, 2014).

To test the effect of attitude certainty on attitude change in humans and digital twins, we first re-coded pre-attitude into attitude extremity—where 0 reflects neutral and any number reflects the absolute difference from 0 (so 50 could be totally in favor or against).

We then ran a linear mixed-effects regression with attitude change as the dependent variable; attitude extremity (0–50), twin (Human vs. Digital), and their interaction as independent variables; and twin identifier as a random intercept. We found a significant interaction ($b = -0.11$, $SE = 0.03$; $t(1795) = -3.18$; $p = .002$). For humans, as attitude extremity increased, attitude change decreased ($b = -0.04$, $SE = 0.02$;

$t(1998) = -2.13$; $p = .033$). The opposite pattern was present for digital twins ($b = 0.06$, $SE = 0.03$; $t(1998) = 2.36$; $p = .018$), consistent with regression to the mean. This provides initial evidence that twins might not reflect the metacognition of their human counterparts.

We also examined the language used when considering the other side. Specifically, we measured the language similarity between a human participant's text response and their twin's response versus the text response of a randomly selected twin using the BERT metric, defined as "the sum of cosine similarities between their tokens' embeddings" [59] In a random-effects regression with language matching as the dependent variable; same-twin versus different-twin pairing as the independent variable; and human participant identifier as a random effect, we found that the language between a human and their twin was slightly more similar ($M = 0.19$, $SD = 0.09$) than between a human and a random twin ($M = 0.18$, $SD = 0.09$; $b = 0.009$, $SE = 0.001$; $t(1000) = 5.84$; $p < .001$).

Task 2. To examine the extent to which humans and digital twins are sensitive to manipulations across the three imagined scenarios, we ran three linear mixed-effects regressions with the response for each scenario (group project, puzzle, lying in bed) as the dependent variable; manipulation (Baseline vs. Treatment), twin (Human vs. Digital), and their interaction as independent variables; and twin identifier as a random intercept.

Each scenario yielded a significant interaction (Group project: $b = 0.34$, $SE = 0.10$; $t(4994) = 3.48$; $p < .001$. Puzzle: $b = 0.59$, $SE = 0.10$; $t(4994) = 5.99$; $p < .001$. Lying in bed: $b = -0.45$, $SE = 0.10$; $t(4994) = -4.60$; $p < .001$). For the group project scenario, humans (Control: $M = 2.97$, $SD = 1.17$; Treatment: $M = 3.45$, $SD = 1.23$; $b = 0.44$, $SE = 0.07$; $t(5710) = 5.92$; $p < .001$) were more affected by imagining conflict than digital twins (Control: $M = 2.83$, $SD = 1.20$; Treatment: $M = 2.97$, $SD = 1.18$; $b = 0.10$, $SE = 0.07$; $t(5710) = 1.30$; $p = .193$). Similarly, for the puzzle scenario, humans were more affected by imagining time pressure (Control: $M = 3.15$, $SD = 1.24$; Treatment: $M = 4.37$, $SD = 1.34$; $b = 1.21$, $SE = 0.07$; $t(5711) = 16.38$; $p < .001$) than digital twins (Control: $M = 2.82$, $SD = 1.36$; Treatment: $M = 3.45$, $SD = 1.52$; $b = 0.62$, $SE = 0.07$; $t(5711.16) = 8.44$; $p < .001$). For the lying in bed scenario, digital twins showed larger responses to imagining leg pain (Control: $M = 2.21$, $SD = 0.71$; Treatment: $M = 2.78$, $SD = 0.94$; $b = 0.57$, $SE = 0.07$; $t(5710) = 7.74$; $p < .001$) than humans (Control: $M = 2.59$, $SD = 1.47$; Treatment: $M = 2.71$, $SD = 1.46$; $b = 0.12$, $SE = 0.07$; $t(5710) = 1.65$; $p = .100$). See Figure S35.

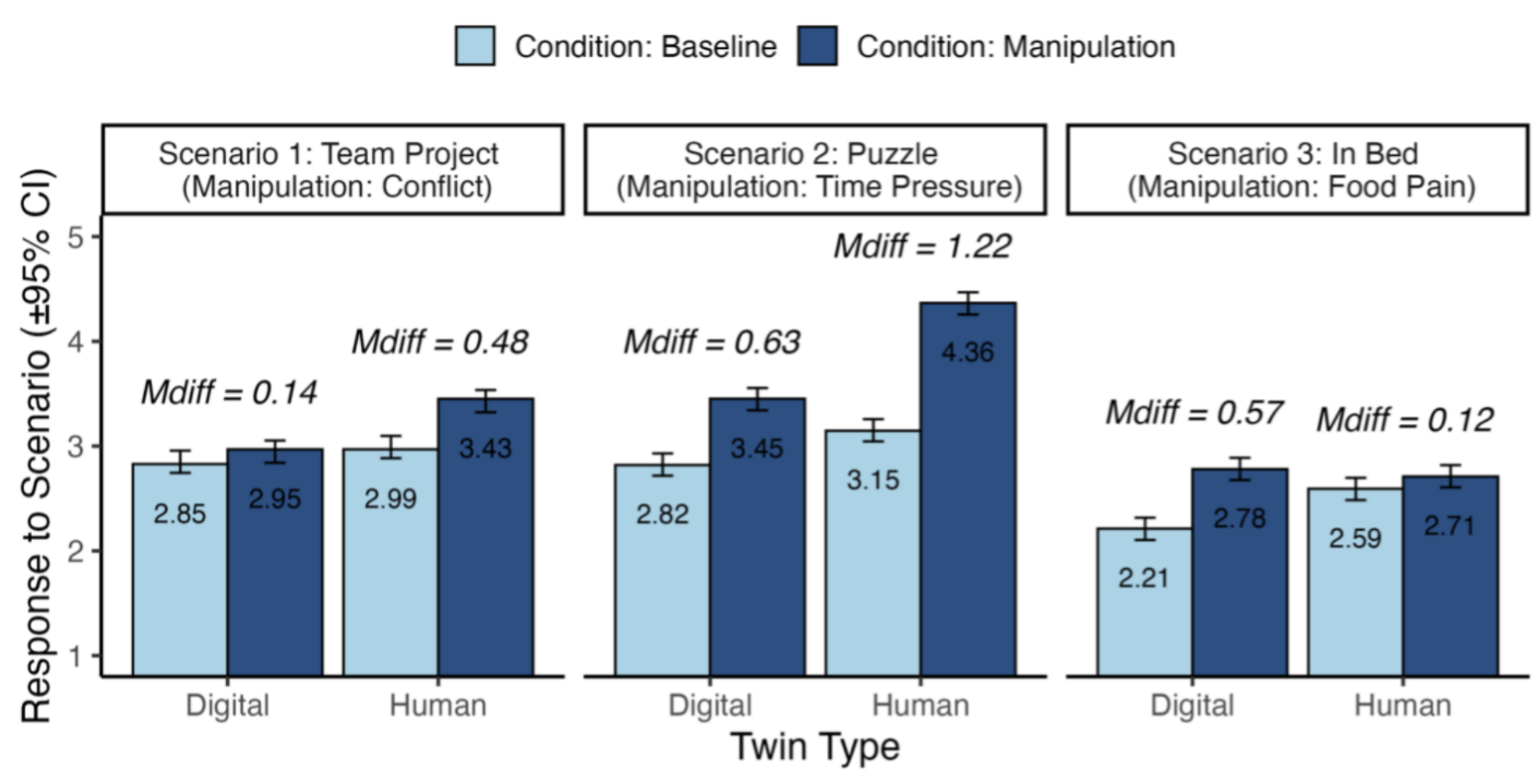

Fig. S35 Effect of Imagined Scenarios on Digital Twins vs. Humans.

S10.13.5 Discussion

Returning to each research question:

1. Self-persuasion [37]. When prompted to consider the other side, do digital twins abandon their attitudes more readily than their human counterparts?

No. In fact, twins were less sensitive to considering a different point of view than humans (although this could be explained by our slight wording modification between humans and twins). Additional analyses suggest this also could be due to twins not reflecting the metacognition of their human counterparts.

2. Scenarios. Do digital twins more "obediently" follow the instructions to imagine themselves in different scenarios, leading to more sensitivity to scenario manipulations?

No. In fact, twins were less sensitive overall to these scenario manipulations than humans. Looking at individual scenarios, twins were less sensitive than humans for imagined conflict and time pressure, but more sensitive when imagining pain.

3. Absurd Scenarios. Do digital twins "earnestly" respond to instructions that would be non-sensical to their human counterparts?

No. If anything, humans were more sensitive to the instructions to forecast feelings in absurd scenarios.

In sum, across all three task types, we do not find e vidence s upporting t he possibility that because twins obediently follow orders, they are more sensitive to survey

instructions, such as "consider the alternative point of view" or "imagine..." If anything, we find that humans, on average, respond more to instructions in this survey than digital twins.

S10.14 Privacy Preferences

S10.14.1 Main Questions/Hypotheses

Consumers have privacy concerns regarding tracking and targeting in online advertising. In response to such privacy concerns about personal data collection and use, the online advertising industry is developing technically sophisticated privacy-enhancing technologies (PETs). On the premise that consumers perceive their privacy to be violated differently under different advertising practices that employ data tracking and targeting differently, we want to study how well their digital twins capture these perceptions.

Motivated by Google's Privacy Sandbox initiative, we study six main practices of firms in online advertising, labeled as different scenarios from A to F. For the status quo of behavioral targeting (Scenario F), the degree of tracking is high because the consumer is tracked across websites and the data leaves the local machine, and the degree of targeting is also high because the consumer is targeted at the individual level. For the Individual-level Targeting PET (Scenario E), the degree of tracking is low because although the consumer's activity is tracked, the consumer's data does not leave the machine; however, the degree of targeting is high because the consumer receives individually-targeted ads. For the Group-level Targeting PET (Scenario D), the degree of tracking is low because although the consumer's activity is tracked, the consumer's data does not leave the machine. In this case, the degree of targeting is at a medium level because the consumer is profiled and receives ads targeted at a group level. For contextual targeting (Scenario C), the degrees of tracking and targeting are both low, as the focal website only determines the individual-level presence of the user on the website. The consumer is not tracked at the individual-level on the focal website they are visiting and on other websites (i.e., no past behavioral browsing data is used for profiling and targeting the user, and the only data used for targeting is the fact that the consumer is present on the website); however, contextual targeting may still trigger privacy concerns (Bleier 2021). When untargeted ads are shown to consumers and they are not tracked (Scenario B), or there is no tracking and no ads are shown to consumers (Scenario A), the degrees of tracking and targeting are both zero. We expect that consumers' average perceived privacy violations (PPVs) will be in the order Scenarios F, E, D, C, B and A (with F having the highest PPV and A the lowest).

The main question that we want to investigate is how PPV values compare for humans and their digital twins in magnitude (in aggregate and individually) as well as in an ordinal sense (in aggregate).

S10.14.2 Methods

We measure consumers' PPVs for the advertising practices in Scenarios A through F through an online study with approximately 1,200 subjects (i.e., approximately 200 per condition) in the United States. A human, allocated randomly to a scenario, is presented with a description of the scenario and asked how much they perceive their privacy to be violated by this advertising practice. Later the digital twin of this human is asked the exact same question.

S10.14.3 Results - Pre-registered Analyses

For each of the 6 conditions, Table S25 reports: the human-twin sample size; mean PPV by human respondents (along with the lower and upper bounds of the 95% confidence interval); mean PPV by digital twins of human respondents (along with the lower and upper bounds of the 95% confidence interval); Pearson r between human and twin PPVs; paired t-test on PPVs; and F-test on variances. Table S26 provides the overall Spearman rho on the rank ordering of condition means. Twins answered "1 - not at all" for all observations in Scenarios A, B and C, so correlations and certain tests are undefined in these scenarios.

Measure	A: No Ads, No Tracking	B: Untargeted Ads	C: Contextual Targeting	D: Group-level PET	E: Individual-level PET	F: Behavioral Targeting
N_pairs	199	218	187	222	180	194
Mean_human	1.744	2.133	2.658	4.667	4.461	5.469
Mean_twin	1	1	1	2.712	2.817	4.603
CI95_hu_low	1.553	1.935	2.396	4.443	4.214	5.246
CI95_hu_high	1.935	2.331	2.919	4.890	4.709	5.692
CI95_tw_low	1	1	1	2.616	2.727	4.476
CI95_tw_high	1	1	1	2.807	2.907	4.731
Pearson_r				0.014	-0.069	0.088
p-val_r				0.840	0.355	0.222
t_stat	7.676	11.305	12.496	15.945	12.058	6.907
p-val_t	0	0	0	0	0	0
F_var				5.446	7.569	3.070
p-val_F				0	0	0

Table S25: **Human and Twin Responses per Condition.**

	rho	p-value
Spearman_rank_corr	0.88	0.021

Table S26: **Spearman rank correlation of condition means from Human vs. Twins.**

The results of the experiments show the following:

- The PPVs for human subjects are in the order hypothesized (with one deviation being that the mean PPV of Scenario D is higher than the mean PPV of Scenario E; however, these numbers are not statistically different accounting for the 95% CIs).
- The PPVs for the digital twins are significantly lower in magnitude, i.e., digital twins have lower perceptions of privacy violations.

  - All twins give PPV values of 1 for Scenarios A, B and C, in which there is no tracking or targeting on past behavior.
  - The Pearson correlations between the PPVs of humans and their digital twins, when defined, are near zero.
  - The t-stats for the paired t-test between PPVs of humans and their digital twins show that these PPVs are significantly different.
  - The F-test of variances between PPVs of humans and their digital twins show that the variances in PPVs are significantly different.

- While the PPVs of digital twins are significantly lower than the PPVs of humans, the rank ordering of the scenarios, based on average PPV, is similar for humans and digital twins, with a Spearman rank correlation of 0.88.

S10.14.4 Discussion

The results of the study indicate that digital twins perceive their privacy to be violated less than their human counterparts (as indicated by lower PPV scores for digital twins compared to humans). The twins also do not do a good job of capturing heterogeneity in perceived privacy violations (as indicated by almost zero correlations in PPV scores for digital twins compared to humans). However, in aggregate, humans and their digital twins rank privacy violations similarly across advertising practices that rely differently on tracking and targeting (as indicated by a high rank correlation).

S10.15 Preferences for Redistribution

S10.15.1 Main Questions/Hypotheses

Building on the seminal work of [85], this study assesses the ability of digital twins to replicate individuals' preferences for redistribution and explores the mechanisms through which this replication may fail. Our instruments come from the General Social Science (GSS), a major social-science survey in the U.S. Preferences for redistribution are measured through both a standard question (asking whether the government should do everything possible to improve the standard of living of all poor Americans) and a health-tax willingness question. In addition, mechanism variables include fairness judgments, work-versus-luck beliefs, father's education, and trust in others.

Understanding how accurately digital twins mirror human attitudes on these topics is crucial for policymakers, since public support for tax policy, welfare programs, and social-cohesion initiatives depends directly on redistribution and trust levels, while beliefs about fairness and effort versus luck inform education and labor-market reform.

Our investigation proceeds in two stages. First, we examine whether digital twins systematically differ from individuals in their preferences for redistribution. Second, we investigate whether any observed discrepancies can be explained by the twins' failure to replicate responses related to socio-economic heritage (father's education), fairness judgments, beliefs about the relative role of effort versus luck in achieving success, and interpersonal trust. These have been shown to predict redistribution preferences [85].

S10.15.2 Methods

We administered a single, online questionnaire—closely modeled on core GSS items—to approximately 1,200 individuals and their matched digital twins. Respondents completed various questions; those of interest for this analysis are presented below. We recoded every item so that higher numeric values consistently reflected stronger endorsement (and set all "Don't know" or non-substantive responses to missing). Father's education categories were converted to corresponding years of schooling (10–18 years). Specifically:

1. **Redistribution 1 (Government Responsibility).**
   "Some people think that the government in Washington should do everything possible to improve the standard of living of all poor Americans; they are at Point 1 on the scale below. Other people think it is not the government's responsibility, and that each person should take care of himself; they are at Point 5. Where would you place yourself on this scale, or haven't you made up your mind on this?"
   (a) **Response Options & Coding:**
      (i) 1 – I strongly agree the government should improve living standards → 5
      (ii) 2 → 4
      (iii) 3 – I agree with both answers → 3
      (iv) 4 → 2
      (v) 5 – I strongly agree people should take care of themselves → 1
      (vi) Don't know/No answer → missing

2. **Redistribution 2 (Health-Care Tax Willingness).**
   "How willing would you be to pay higher taxes to improve the level of health care for all people in the United States?"
   (a) **Response Options & Coding:**
      (i) Very willing → 5
      (ii) Fairly willing → 4
      (iii) Neither willing nor unwilling → 3
      (iv) Fairly unwilling → 2
      (v) Very unwilling → 1
      (vi) Don't know/Can't choose → missing
3. **Fairness Judgment.**
   "Which comes closer to your view of most people: (1) Most people would take advantage of you if they got the chance; (2) Most people would try to be fair; or (3) It depends?"
   (a) **Response Options & Coding:**
      (i) "Would take advantage of you" → 3
      (ii) "It depends" → 2
      (iii) "Would try to be fair" → 1
      (iv) Don't know → missing
4. **Work vs. Luck.**
   "Which comes closer to your view—that hard work MOST contributes to someone getting ahead in life, that luck or help from other people MOST contributes, or that hard work and luck are about equally important?"
   (a) **Response Options & Coding:**
      (i) Hard work most important → 1
      (ii) Hard work and luck equally important → 2
      (iii) Luck or help from others most important → 3
      (iv) Don't know → missing
5. **Interpersonal Trust.**
   "Generally speaking, would you say that most people can be trusted, or that you can't be too careful in dealing with people?"
   (a) Response Options & Coding:
      (i) "Most people can be trusted" → 3
      (ii) "Depends" → 2
      (iii) "Can't be too careful" → 1
      (iv) Don't know → missing
6. **Father's Education.**
   "What is the highest level of education completed by your father?"
   (a) **Response Options & Coding (years of schooling):**
      (i) Less than high school → 10
      (ii) High school graduate → 12
      (iii) Some college, no degree → 13
      (iv) Associate's degree → 14
      (v) College graduate / some postgraduate → 16
      (vi) Postgraduate degree → 18

(vii) Don't know/No answer → missing

After collecting the raw survey data, we removed any respondent whose completion time fell below 50 percent of the sample median or who failed our attention-check prompt. We then merged the cleaned human and twin datasets one-to-one on their unique identifiers, producing 1,163 matched pairs for each outcome. For each of our six measures, we calculated the Pearson correlation (with Fisher confidence intervals) to gauge twin resemblance, carried out paired t-tests to compare mean differences, performed F-tests to assess variance equality, and derived an accuracy index (1 minus the mean absolute difference divided by the variable's range). Although not pre-registered, we also generated side-by-side percent histograms of each response distribution to identify any systematic replication discrepancies.

S10.15.3 Results - Pre-registered Analyses

Measure	Redistribution 1	Redistribution 2	Fairness	Work vs. Luck	Father's Education	Trust in Others
Accuracy	0.780	0.778	0.641	0.770	0.733	0.593
Correlation	0.621	0.614	0.298	0.346	0.126	0.296
Mean (Human)	3.55	3.27	1.96	1.85	13.66	1.73
Mean (Twin)	3.44	3.61	1.71	1.70	12.09	2.22
t-test (p-value) for means	2.85 ($p < .01$)	–9.14 ($p < .001$)	8.76 ($p < .001$)	7.65 ($p < .001$)	21.55 ($p < .001$)	–16.74 ($p < .001$)
SD (Human)	1.28	1.36	0.74	0.70	2.48	0.73
SD (Twin)	1.65	1.53	0.89	0.48	0.71	0.93
F-test (p-value) for variances	0.600 ($p < .001$)	0.797 ($p = .00012$)	0.699 ($p < .001$)	2.120 ($p < .001$)	12.178 ($p < .001$)	0.625 ($p < .001$)
n	1153	1144	1151	1158	1163	1162

Table S27: Human vs. Twin Responses.

Table S27 shows the results from the pre-registered analysis. Digital twins capture the relative ordering of individuals' redistribution preferences reasonably well: both the standard five-point scale measure (Redistribution 1) and the health-tax willingness question (Redistribution 2) yield strong twin correlations ($r \approx .62$). However, twins tend to slightly underestimate general redistribution support ($\text{mean}_h = 3.55$ vs. $\text{mean}_t = 3.44$; $t = 2.85,\ p = .0044$) and overestimate willingness to pay higher health taxes ($\text{mean}_h = 3.27$ vs. $\text{mean}_t = 3.61$; $t = -9.14,\ p < .001$). Accuracy scores around .78 for each measure indicate that, on average, twins miss individual scores by nearly a full point on the five-point scales. Moreover, significant variance differences ($F = .60$ and $F = .80$, both $p < .001$) suggest that twins' responses are more dispersed compared to human distributions. Thus, while digital twins reflect who is relatively more or less supportive of redistribution, they systematically misestimate absolute levels and spread.

To understand why these mismatches occur, we examined additional constructs known to shape redistribution attitudes [85]. Twins poorly replicate socio-economic heritage—father's education shows a low correlation ($r \approx .13$), a mean gap of over 1.5 years ($t = 21.55,\ p < .001$), and a sharply reduced variance ($F = 12.18,\ p < .001$). Fairness judgments ($r \approx .30$; accuracy $\approx .64$) and work-versus-luck beliefs ($r \approx .35$; accuracy $\approx .77$) similarly display moderate rank-order similarity but pronounced

mean and variance discrepancies. Interpersonal trust also diverges, with twins overstating trust ($\text{mean}_t = 2.22$ vs. $\text{mean}_h = 1.73$; $t = 10.38$, $p < .001$). Because these background factors and beliefs are important predictors of redistribution preferences, their imperfect replication by digital twins likely underlies the systematic errors in predicting absolute levels of support for redistribution.

S10.15.4 Results - Additional Analyses (Non-Preregistered)

We also present histograms of our outcomes to compare the response distributions of humans and twins more effectively. As shown in Figure S36, twins exhibit more extreme redistribution preferences, with the heavier tails driving the higher standard deviation observed above. They are likewise more prone to overstate that others behave fairly and to report greater trust in fellow individuals. Twins also more frequently endorse that both effort and luck matter equally for success. Finally, nearly 100% of twins report that their father completed high school (12 years of education), whereas the human sample shows a much more diverse educational distribution.

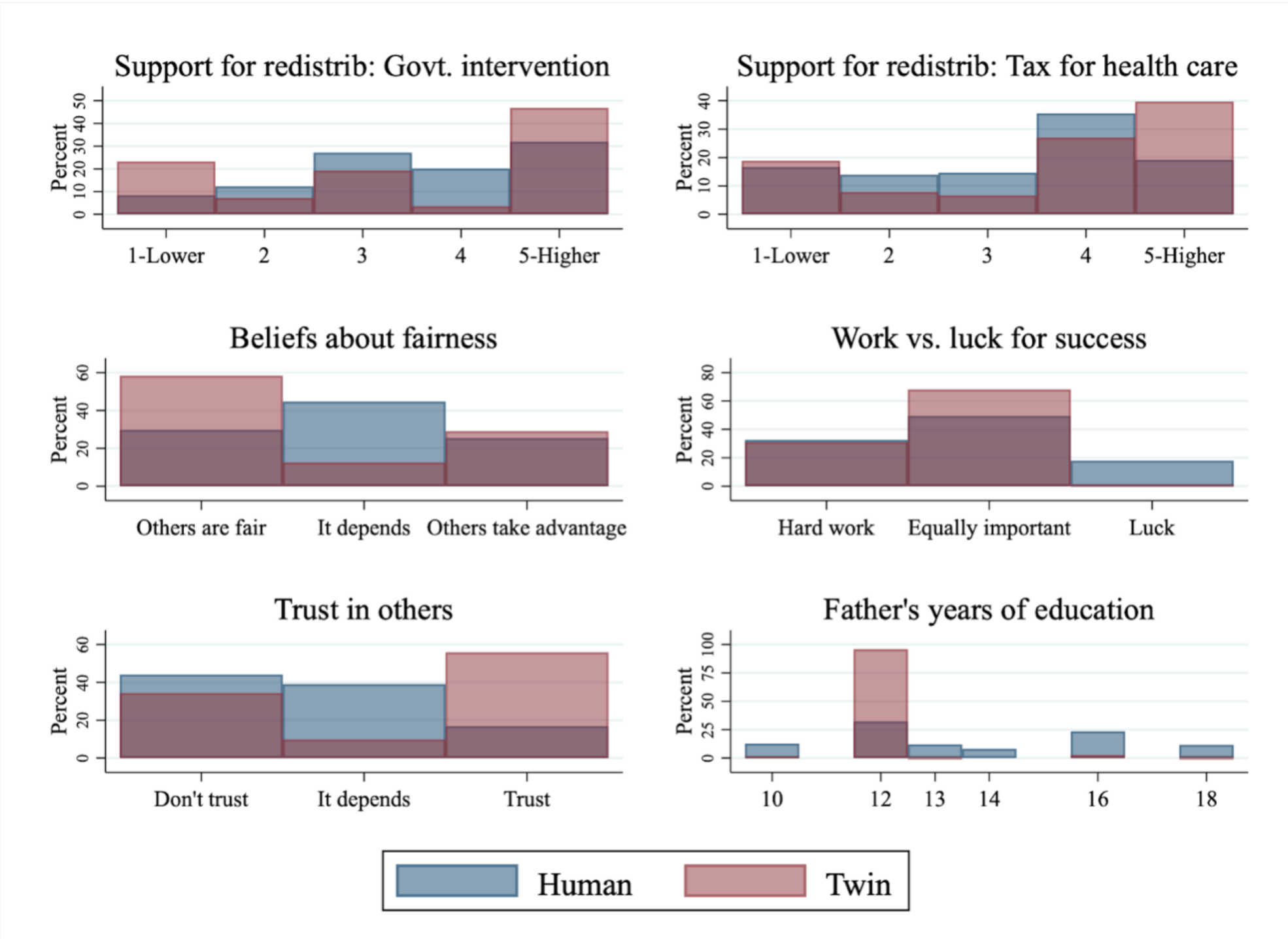

Fig. S36 Comparison of Response Distributions.

S10.15.5 Discussion

In summary, although digital twins reliably reproduce the relative ranking of individuals' redistribution preferences, as evidenced by strong correlations on both the standard scale and health-tax question, they consistently misestimate the absolute levels and dispersion of those preferences. Our analysis suggests these systematic errors stem from twins' failures to mirror key background factors and beliefs: most notably socio-economic heritage (father's education), fairness judgments, work-versus-luck attributions, and interpersonal trust, which are themselves only moderately correlated and exhibit pronounced mean and variance discrepancies. The heavier tails in twins' redistribution responses further underscore their tendency toward more extreme positions. For policymakers and researchers considering the use of digital avatars to gauge public opinion, these findings highlight the importance of refining the modeling of underlying socio-cognitive constructs; without accurately capturing individuals' formative experiences and normative beliefs, digital proxies will misrepresent both the magnitude and variability of real-world attitudes, potentially leading to misguided inferences about public support for tax, welfare, and social-cohesion initiatives.

S10.16 Promiscuous Donors

S10.16.1 Main Questions/Hypotheses

Political donations play a critical role in shaping electoral outcomes. They equip candidates with the financial resources needed to compete in political races (e.g., [86, 87]). They also function as signals of support. Political parties often use donation counts as thresholds for unlocking institutional backing and as a pre-requisite for qualifying for televised debates. In the present research, we investigate the reputational consequences of a specific form of donation behavior: contributing to both major political parties. We refer to individuals who donate to two parties as "promiscuous donors" and we examine how they are perceived by others. Understanding how promiscuous donors are perceived is important given the prevalence of this behavior. Analysis of 2024 Federal Election Commission data reveals that at least 113,187 Americans made contributions to both parties during the 2024 election cycle.

Ex ante, it is unclear how individuals will evaluate those who donate to both political parties. On one hand, promiscuous donors may be viewed favorably. Some scholars have argued that political polarization has reached unsustainable levels [88], prompting a public desire for greater bipartisanship and reconciliation. From this perspective, contributing to both parties could signal a conciliatory stance, potentially enhancing reputational standing.

On the other hand, several theoretical perspectives suggest that such behavior may incur reputational costs. Partisan animosity has intensified in recent years, with Democrats and Republicans expressing growing levels of dislike and distrust toward each other [89]. Furthermore, emerging research on receptiveness to opposing views [90] suggests that open-mindedness toward the other side can backfire socially [91]. If donors are seen as legitimizing or aligning with an ideologically objectionable outgroup, their behavior may be perceived as disloyal or suspect, thereby leading to reputational costs.

S10.16.2 Methods

We recruited 799 human respondents on Prolific and their respective twins. This study used a rating-based conjoint design [92]. Conjoint is a study design used to determine how people value different attributes (e.g., screen size, memory, color) that make up a product (e.g., a laptop). Participants in a conjoint study are shown a series of trials, each showcasing a different version of the target product. By varying different attribute levels of the product (e.g., a black vs. silver laptop) between trials and measuring how consumers respond to different combinations of these attributes, managers can quantify how much a given attribute (e.g., color) affects overall attitudes.

Applying this approach, we had participants read 10 vignettes, each describing a target who engaged in a different donation behavior. The donation behaviors included contributing solely to the Republican Party, solely to the Democratic Party, to neither party, or to both parties. Our primary focus was on the reputational consequences of donating to both parties. To this end, we systematically varied the rationale provided for such behavior. Specifically, we compared conditions in which no justification was

given to those in which a self-interested or a values-based rationale was offered. Self-interested justifications emphasized pragmatic benefits, such as attracting bipartisan talent to one's business or facilitating smoother relations with elected officials across party lines. In contrast, values-based justifications highlighted ideological motivations, such as supporting candidates with the best ideas regardless of party or promoting cross-party cooperation. Below is a summary of the exact description used and the attribute level they correspond to:

- Own Party or Opposing Party (coded relative to participant's party affiliation; independent participants who rated these had their own category):
  - "I currently only donate to Republicans"
  - "I currently only donate to Democrats"
- No donations: "I currently don't donate to any politician"
- Both: "I currently donate to both Democrats and Republicans"
- Business Strategy: "I currently donate to both Democrats and Republicans so my business can work well with whoever wins"
- Attract Talent: "I currently donate to both Democrats and Republicans because it helps me attract top talent for my business"
- Common Grounds: "I currently donate to both Democrats and Republicans because I believe in working across the aisle to find common grounds"
- Good Ideas: "I currently donate to both Democrats and Republicans because I support candidates who have good ideas, regardless of which party they come from"

To enhance the generalizability of our findings, we systematically varied multiple dimensions of the vignettes beyond donation behavior. This design enabled us to estimate the average effect of donating to both parties across a range of contextual backgrounds. Specifically, we manipulated the target's political affiliation (Democrat vs. Republican), job (e.g., CEO of a publicly traded company vs. founder of a fast-growing start-up), and donation history (e.g., history of prior donations vs. first-time donors). This approach ensured that observed effects were not idiosyncratic to a single type of target. Below is an example vignette used in the study:

> Imagine you meet someone named John at a neighborhood barbecue.
>
> John recently moved to your area. He is a Democrat. He is the CEO of a publicly traded company.
>
> During your conversation, the topic of donating to political campaigns comes up.
>
> John mentioned that he never used to donate to politicians.
>
> John then said to you:
>
> "I currently donate to both Democrats and Republicans."

After reading the vignette, participants were asked to report their overall impressions of the target: "Based on what John said, what's your overall impression of him?" Participants reported their attitudes on a nine-point bipolar scale, ranging from very unfavorable to very favorable. As a reminder, each participant rated ten targets in total.

S10.16.3 Results - Pre-registered Analyses

As preregistered, we regressed overall impression of the target on dummy variables of the manipulated attributes and participant type (digital twin vs. human). Importantly, we included an interaction between our focal manipulated attribute–donation behavior--and participant type. Standard errors were clustered on the participant level to account for repeated measurement. More specifically, we estimated the following regression:

$$
\begin{aligned}
\widehat{\text{Impression}}_{i,j} = \alpha &+ \vec{\beta}_1 \, \text{donation behavior}_{i,j} \\
&+ \beta_2 \, \text{participant type}_i \\
&+ \vec{\beta}_3 \left(\text{donation behavior}_{i,j} \times \text{participant type}_i\right) \\
&+ \beta_4 \, \text{target party}_{i,j} \\
&+ \vec{\beta}_5 \, \text{history}_{i,j} \\
&+ \vec{\beta}_6 \, \text{job}_{i,j}
\end{aligned}
$$

We were interested in the vector of interactions $\vec{\beta}_3$. Significant i nteractions would suggest that humans and their digital twins differed i n t heir r eactions t o current donation behavior. Non-significant interactions would suggest no difference.

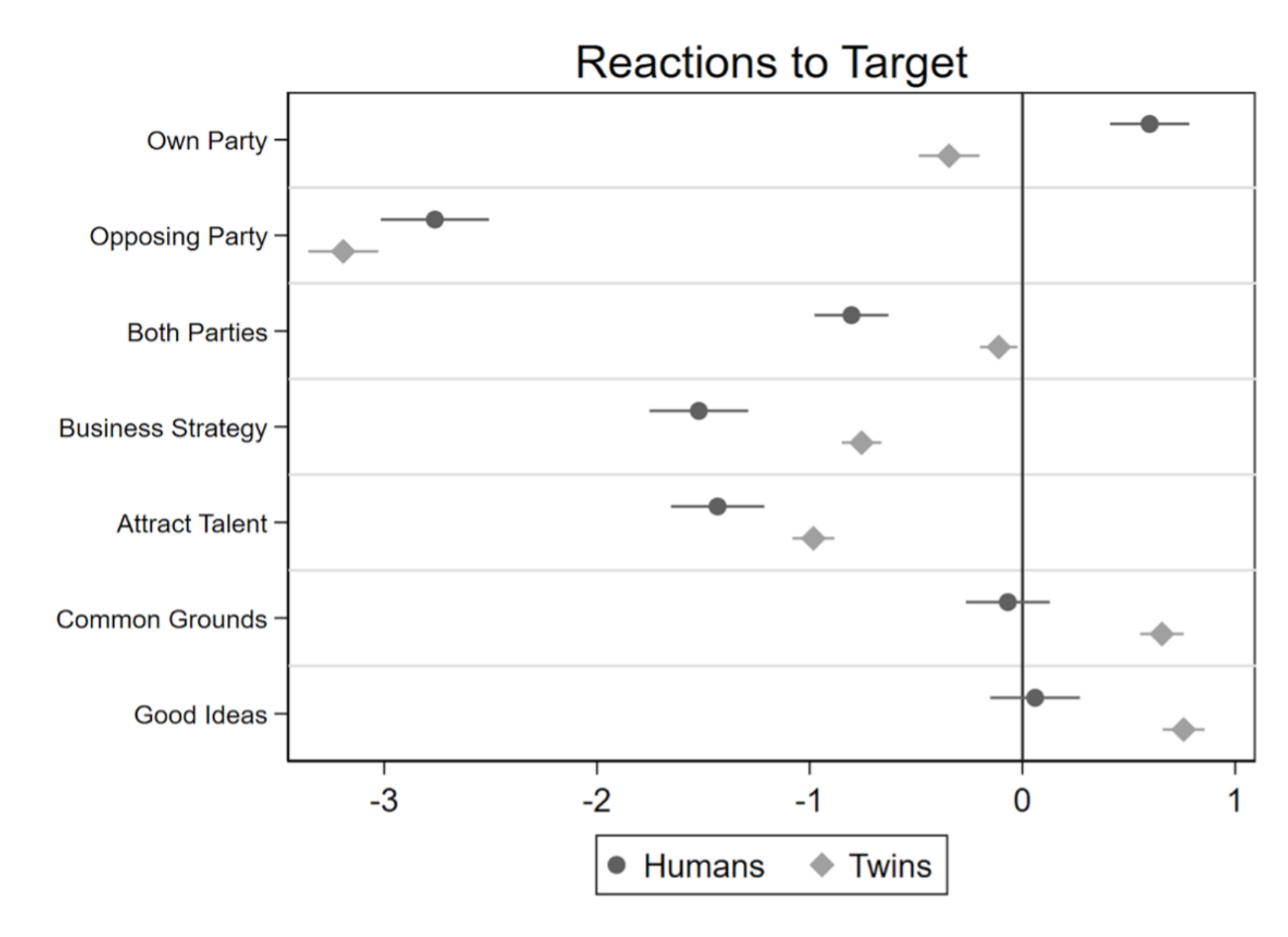

Fig. S37 Reactions to target among humans and digital twins. Error bars denote 95% confidence intervals. Standard errors are clustered at the participant level. The reference category is a target who did not donate to either party. For legibility, the dummy variable indicating that an independent participant rated a single-party donor is omitted from the figure but is included in the regression.

Of the eight interaction terms, seven reached significance ($ps < .001$). Both humans and digital twins evaluated targets who donated to the opposing party more negatively than targets who made no political donations (interaction $b = -0.32,\ SE = 0.27,\ t = -1.18,\ p = .24$).

Four of the seven significant interactions revealed divergent effects in magnitude between human participants and their digital twins. Both humans and twins rated targets who donated to both parties negatively relative to targets who did not donate to either party, but digital twins were more positive in their evaluations ($b = -0.11,\ SE = 0.05,\ t = -2.47,\ p = .014$) than humans ($b = -0.80,\ SE = 0.09,\ t = -9.09,\ p < .001$). Similarly, when targets provided self-interested reasons for donating to both parties, both humans and twins rated them more negatively compared to targets who did not donate at all. However, twins were more positive. For example, both humans and twins rated targets who donated to both parties because they wanted to be able to work with whichever party is in power negatively, but twins were more positive ($b = -0.76,\ SE = 0.05,\ t = -15.86,\ p < .001$) than humans ($b = -1.52, SE = 0.12, t = -12.85, p < .001$). The same pattern held

for targets who donated to both parties because they wanted to attract top talent to their business (humans: $b = -1.43,\ SE = 0.11,\ t = -12.84,\ p < .001$ vs. twins: $b = -0.98,\ SE = 0.05,\ t = -19.56,\ p < .001$).

The three remaining significant interactions revealed divergent effects in direction between human participants and their digital twins. Human respondents rated targets who donated to their own party more positively than those who did not donate ($b = 0.60,\ SE = 0.09,\ t = 6.30,\ p < .001$). In contrast, digital twins evaluated the same targets more negatively compared to those who did not donate ($b = -0.34,\ SE = 0.07,\ t = -4.74,\ p < .001$). A similar divergence emerged in evaluations of targets who donated to both parties for value-based reasons. Humans rated them the same way they rated targets who did not donate to either party. However, twins rated them more positively. Specifically, among humans, there was no significant difference between targets that donated to both parties because they believe in working across the aisle to find common ground and targets who did not donate ($b = -0.07, SE = 0.10,\ t = -0.68,\ p = .50$). However, twins rated the former more positively than targets who did not donate ($b = 0.66,\ SE = 0.05,\ t = 12.61,\ p < .001$). The same pattern held for targets who donated to both parties because they wanted to support the best ideas regardless of which party they came from (humans: $b = 0.06,\ SE = 0.11,\ t = 0.56,\ p = .58$ vs. twins: $b = 0.76,\ SE = 0.05,\ t = 15.12,\ p < .001$).

S10.16.4 Discussion

How do people evaluate promiscuous donors (i.e., targets who donate to both parties)? Our results indicate that, compared to those who do not donate to politics at all, those who donate to both parties are perceived more negatively. This result is consistent with recent research on the reputational costs of receptiveness to opposing views [91]. Interestingly, the reputational costs of promiscuous donation are particularly salient when the reason behind donating to both parties is self-interest (e.g., to attract the best talent to one's business or to make sure that one's business can work with whichever party wins) and are somewhat weaker when the reason is more value-based (e.g., believing in bridging divides or that good ideas should be supported regardless of which party they come from).

How do humans and their digital twins compare when judging the donation behavior of others? Across more than 7,000 conjoint judgments, human respondents penalized promiscuous donors, especially when the behavior appeared motivated by self-interest (e.g., cultivating access or recruiting talent). In contrast, digital twins were less punitive (sometimes even favorable) toward the very same targets.

What might drive these differences? One explanation is that language models are trained to discount signals of intergroup conflict. Hence, a cross-party donation that humans interpret as a collaboration with the enemy is read by the model as pragmatic or even positive as it shows a willingness to reach across divides. A second, complementary account centers on affect: if twins lack the visceral emotions that energize partisan animus, they down-regulate the reputational costs of interacting with the political out-group. Further research on how digital twins compare to humans in matters of social cognition would be worthwhile.

S10.16.5 Appendix - Regression Tables

	(1) rating_
Twins	0.411***
	(0.0694)
Own Party=1	0.502***
	(0.0915)
Opposing Party=1	-2.632***
	(0.127)
Both Parties=1	-0.802***
	(0.0883)
Business Strategy=1	-1.509***
	(0.116)
Attract Talent=1	-1.425***
	(0.110)
Common Grounds=1	-0.0553
	(0.0984)
Good Ideas=1	0.0768
	(0.106)
Partisan Donation (Independent)=1	-1.682***
	(0.243)
Twins # Own Party=1	-0.745***
	(0.117)
Twins # Opposing Party=1	-0.700***
	(0.153)
Twins # Both Parties=1	0.691***
	(0.0994)
Twins # Business Strategy=1	0.739***
	(0.124)
Twins # Attract Talent=1	0.435***
	(0.119)
Twins # Common Grounds=1	0.700***
	(0.110)
Twins # Good Ideas=1	0.665***
	(0.115)
Twins # Partisan Donation (Independent)=1	-0.315
	(0.267)
Target: Own Party	0.311***
	(0.0865)
Target: Opposing Party	-0.0112
	(0.0879)
Start-up Founder	-0.112***
	(0.0369)

	(1) rating_
CEO	-0.197***
	(0.0378)
No Donations	0.0808**
	(0.0318)
Both	0.119***
	(0.0313)
Observations	15,980
R^2	0.311
Adjusted R^2	0.310

Table S28: **Regression results: pooled sample.**

	(1) Ratings Humans	(2) Ratings Twins
Own Party	0.598***	-0.345***
	(0.0949)	(0.0728)
Opposing Party	-2.762***	-3.193***
	(0.130)	(0.0839)
Both Parties	-0.804***	-0.111**
	(0.0884)	(0.0450)
Business Strategy	-1.521***	-0.756***
	(0.118)	(0.0477)
Attract Talent	-1.433***	-0.983***
	(0.112)	(0.0502)
Common Grounds	-0.0684	0.656***
	(0.100)	(0.0520)
Good Ideas	0.0598	0.758***
	(0.108)	(0.0501)
Partisan Donation (Independent)	-1.580***	-2.073***
	(0.255)	(0.0925)
Target: Own Party	0.315*	0.338***
	(0.182)	(0.0582)
Target: Opposing Party	0.218	-0.224***
	(0.183)	(0.0606)
Start-up Founder	-0.0795	-0.146***
	(0.0633)	(0.0375)
CEO	-0.161**	-0.231***
	(0.0662)	(0.0360)
No Donations	0.117**	0.0454
	(0.0571)	(0.0278)
Both	0.127**	0.111***
	(0.0565)	(0.0262)

	(1) Ratings Humans	(2) Ratings Twins
Observations	7,990	7,990
R^2	0.176	0.546
Adjusted R^2	0.175	0.545

Table S29: **Regression results: split by humans vs. twins.**

S10.17 Quantitative Intuition

S10.17.1 Main Questions/Hypotheses

This study evaluates whether digital twins trained from large language models (LLMs) can accurately replicate individual responses on a newly developed psychometric instrument measuring Quantitative Intuition (QI) [93]. The QI scale assesses the respondent's own analytical and intuitive mindsets through 38 self-report items in addition 16 items measuring individuals' perceptions of their organization's analytical and intuitive orientations.

We test two core questions: First, can digital twins accurately reproduce individual human responses to the QI scale? Second, do digital twins match human participants on average scores for the quantitative (Q) and intuitive (I) mindset dimensions? Because the scale was developed in tandem with this study and was not available during LLM training, this provides a strong test of digital twins' ability to predict out-of-sample psychometric responses.

S10.17.2 Methods

We recruited human participants from Prolific who were part of a larger digital twin study and collected matched responses from their corresponding digital twins. Invitations were sent to 2,000 participants from the original twin cohort; 1,435 individuals responded to the survey. Following pre-registered exclusion criteria, we removed 108 respondents who failed at least one of two attention checks, 8 respondents who "straightlined" (i.e., provided the same response across items 32–38 of the scale), and 1 respondent who completed the survey in under 150 seconds (mean response time was 789 seconds with a standard deviation of 457 seconds). The final sample included 1,323 respondents.

Participants completed 38 self-assessment items measuring their individual Quantitative Intuition (QI) mindset, followed by 16 items assessing their perception of their current or former organization's QI mindset. In addition, participants responded to two behavioral QI questions—one closed-ended and one open-ended—designed to capture willingness to "guestimate" and synthesize information. These behavioral items serve as external validity checks for the QI scale.

To explore individual differences in the QI scores, we examined correlations between QI scores and demographic and psychological variables collected as part of the broader digital twin survey.

Digital twins were prompted to complete the same set of items as their human counterparts, simulating how the participant might respond.

We derived individual QI scores using two approaches: 1) Simple averaging: we computed the mean of the first 19 items as a quantitative mindset score (Q), and the mean of the next 19 items as an intuitive mindset score (I), and 2) Factor analysis: we conducted an exploratory factor analysis with two factors, which naturally aligned with the quantitative and intuitive dimensions. Factor scores from this model were used as alternative QI measures.

We then compare the individual, simple average and factor analysis scores of the human respondents with those of the digital twins.

S10.17.3 Results - Pre-registered Analyses

As pre-registered we compared the actual QI score of the Prolific respondents with the QI score of their digital twins. We do so both at the individual question level and for the average of the individual and organizational quantitative and intuitive scores.

Comparing the average score of the human responses and the digital twins

Comparing the correlation between the average (or factor) scores between the actual respondents and the digital twins for the individual and organizational quantitative and intuitive scores we find that overall the digital twins answers are closer to the actual respondents answers for the individual relative to the organizational skill both for the average quantitative and intuitive scores and for the factor analysis scores.

The Cronbach's α for the Q and I individual scales for the human respondents are 0.788 and 0.753, respectively, suggesting acceptable levels of agreement for items in the scale. Interestingly, the digital twin mimicked similar or even higher levels of agreement for items in the scale, with Cronbach's α of 0.776 and 0.866 for the Q and I individual scales.

Comparing the means and standard deviations of the scale scores between the humans and and digital twins shows that the means are quite similar, but the digital twins had consistently lower variance in the responses across respondents. A possible reason for that is that the digital twins are still regressing to some overall LLM mean.

Scale		Human	Digital Twin
Q Ind.	Mean	3.579	3.573
	STD	0.451	0.355
I Ind.	Mean	3.478	3.713
	STD	0.412	0.394
Q Org.	Mean	3.580	3.014
	STD	0.643	0.418
I Org.	Mean	3.219	3.479
	STD	0.486	0.280

Table S30: **Quantitative and Intuitive Scales: Human vs. Digital Twin.**

Correlations between the human responses and the digital twins

Looking at the correlations between the individual-level QI scale responses and those of the digital twins, we find a moderately strong correlation between the responses to the 19 quantitative questions ($r = 0.513,\ p < .001$) and the 19 individual scores ($r = 0.481,\ p < .001$), with an overall correlation of $r = 0.46$. Thus, at the individual level the digital twins are capable of mimicking the human response to QI to a moderate extent. The correlations between the digital twins and the human responses for the organizational QI scores are much lower, though statistically significant ($r_{\text{quantitative, org}} = 0.252,\ p < .001$ and $r_{\text{intuitive, org}} = 0.196,\ p < .001$). This is to be expected as the original questions used to build the digital twins focused on the individuals themselves rather than on the organization they work/worked for.

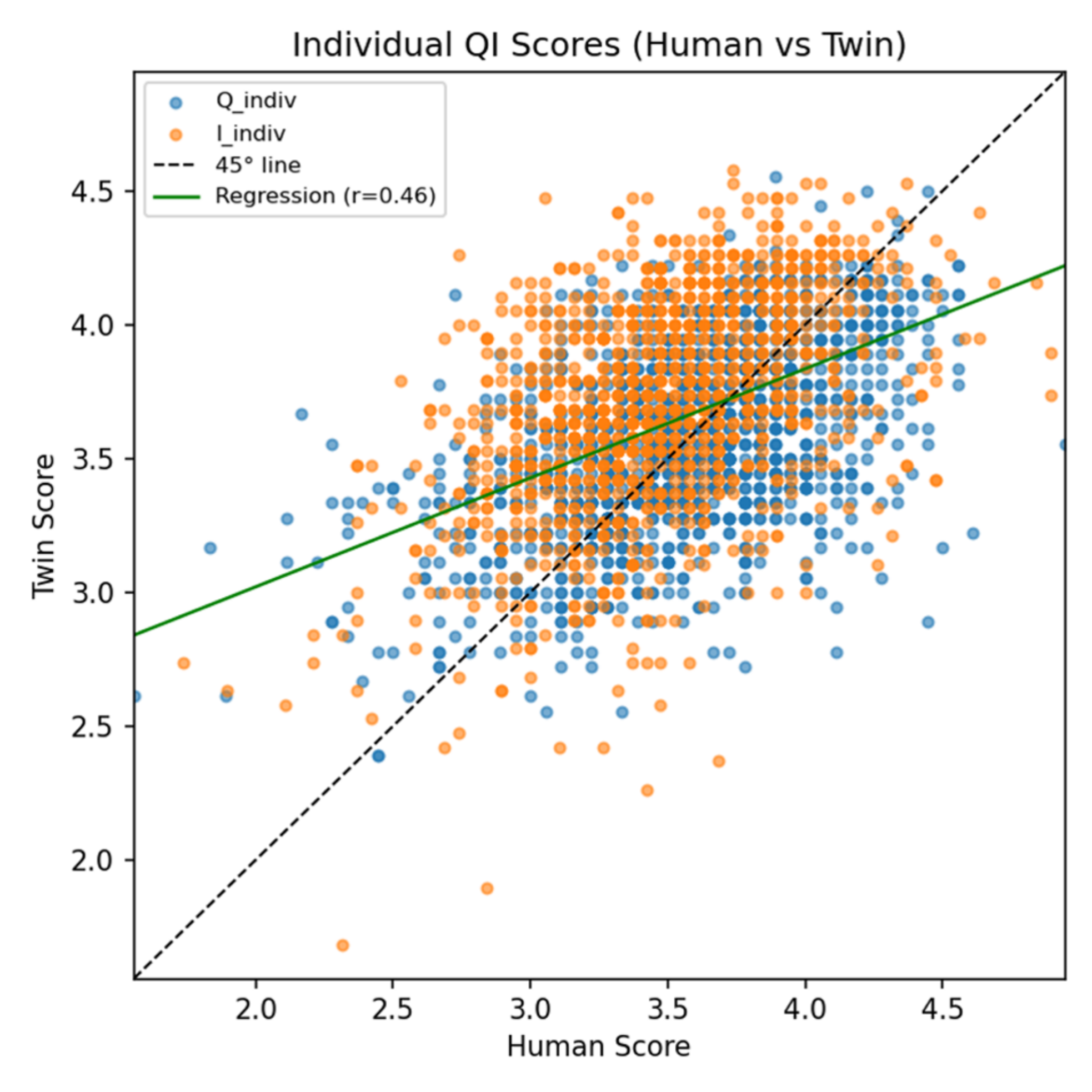

Fig. S38 Human vs. twin scores.

Finally, looking at the individual questions we find a positive and significant (after Bonferroni correction for multiple tests) correlation at the 95% level between the human digital twins scores for 35 out of the 38 individual QI questions. For the organizational QI questions, we find a positive and significant (after Bonferroni correction for multiple tests) correlation at the 95% level between the human and digital twin responses for 11 out of 16 questions. We did not find any significant and negative correlations between the human and digital twin responses for any of the questions.

We did not find a statistically significant relationship between the QI scores for either the willingness to guestimate questions or the ability to synthesize scores. However, we found an interesting result with respect to the ability of the digital twins to answer the QI behavioral questions. In a close ended question, we gave respondents

some pieces of information and challenged them to address the problem by guestimation. The digital twins chose to guestimate, which is the normative answer for the guestimation question, 99.9% of the times (only one twin did not choose the normative response), whereas for the humans only 59.4% were inclined to guestimate. The difference between the human and digital twin responses is statistically significant ($p < .001$).

In an open-ended question, we asked respondents to explain the situation for a struggling company based on data, where the normative QI response would be to synthesize the information rather than mainly summarize it. We asked GPT-4o mini to classify each human or digital twin response as a summary or a synthesis. A research assistant independently classified 20 responses and found that their classification perfectly matched with those of ChatGPT. We found a similar result to those of the willingness to guesstimate for the ability to synthesize. Whereas 99.8% of the digital twins synthesized the information, only 71.5% of human respondents replied with the QI normative response to synthesize. The difference between the human and digital twin responses is statistically significant ($p < .001$).

Thus, while the digital twins are capable of moderately following human responses to the QI scale, when it comes to mimicking human behavior questions, they tend to overexhibit behavior that is consistent with the normative QI mindset.

S10.17.4 Results - Additional Analyses (Non-Preregistered)

We also analyzed the relationship between the Q and I scores and the demographic and individual characteristics collected in the first phase of the digital twins study. Specifically, we calculated the correlations between the average individual Q and I scores and each of the scales measured in that initial phase. Focusing on individual rather than organizational QI scores, we report only the correlations that are statistically significant at the 95% confidence level after applying the Bonferroni correction for multiple testing.

Several interesting results emerge.

1. The individual mindset score (I) is statistically significantly correlated with the following scales at the 95% level (see [**?**] for details on each scale):
    1. Regulatory Focus - r=0.452
    2. Openness - r=0.428
    3. Consumer Need for Uniqueness - r=0.379
    4. Self monitoring - 0.352
    5. Agentic - r=0.306
    6. Extraversion - r=0.285
    7. Need for Cognition - r=0.258
    8. Green - r=0.213
    9. Maximization - r=0.212
    10. Overconfidence - r=0.198
    11. The Horizontal and Vertical Individualism and Collectivism scales - r=0.169, r=0.182. r=0.217. r=0.125.
    12. Communion - r=0.158

13. Basic Empathy - r=0.149
14. Minimalism - r=0.106
15. Total performance on the cognitive test - r= -0.113.
16. Cognitive Reflection Test 2 - r= -0.116

Thus, individuals who score high on having an intuitive mindset also tend to score high on regulatory focus and openness—traits that are correlated with greater emotional awareness, creativity, and unconventionality. They also tend to seek uniqueness, display overconfidence, be extraverted, agentic, and self-aware. While they perform well on communication measures, they tend to score lower on cognitive tests. Of particular interest is the negative correlation with the Cognitive Reflection Test 2 (CRT-2), which assesses individuals' ability to override intuitive (System 1) responses in favor of more deliberate (System 2) reasoning. This negative correlation aligns with our expectations: a high intuitive mindset score on our scale should correspond to greater reliance on System 1 processing. This finding provides a compelling validation of our scale.

2. The analytical/quantitative mindset score (Q) is statistically significantly correlated with the following scales at the 95% level:
    1. Need for Cognition - r=0.380
    2. Regulatory Focus - r=0.342
    3. Conscientiousness - r=0.320
    4. Openness - r=0.259
    5. Self Monitoring - 0.248
    6. Minimalism - r=0.237
    7. Green - r=0.215
    8. The Horizontal and Vertical Individualism and Collectivism scales - r=0.223, r=0.195. r=0.210. r=0.153.
    9. Agentic score - r=0.206
    10. Numeracy - r=0.170
    11. Maximization - r=0.162
    12. Consumer Need for Uniqueness - r=0.142
    13. Overconfidence - r=0.140
    14. Extraversion - r=0.136
    15. Financial literacy - r=0.134
    16. Agreeableness - r=0.132
    17. Communion score - r= 0.103
    18. Self Concept Clarity - r=0.099
    19. Social desirability - r= 0.097
    20. Depression - r= -0.119
    21. Neuroticism - r= -0.183

Several measures that are statistically significantly and positively correlated with the intuitive mindset scale (I) are also correlated with the analytical mindset scale (Q). These include need for cognition, regulatory focus, openness, self-monitoring, individualism, collectivism, minimalism, environmental concern ("green"), agentic traits, communication skills, and overconfidence.

On the other hand, several individual characteristics appear to be uniquely associated with scoring high on the analytical (Q) scale. Individuals who scored high on the analytics mindset questions tend to also score high on conscientiousness, a trait often linked to organization and discipline, as well as on numeracy and financial literacy, which reflect core quantitative skills. They also score higher on agreeableness, social desirability, and self-concept clarity, and lower on neuroticism and depression. Additionally, an analytical mindset is positively and significantly associated with being male and having a higher income.

S10.17.5 Discussion

Overall, we first validated the QI scale by running it on a large sample of respondents, identifying a meaningful two-factor structure that captures both an analytical mindset and an intuitive mindset. We also found meaningful and expected relationships between the QI scale and a set of individual characteristics—such as higher numeracy, financial literacy, and conscientiousness among individuals with an analytical mindset, and a stronger preference for System 1 processing among those with an intuitive mindset.

In the digital twins analysis, we found a moderate correlation (around 0.5) between human responses to the individual QI scale and those of their digital twins, and a weaker relationship (correlation of 0.2-0.25) between human responses and their digital twins' assessments of the QI score of the organization they work/ed for, possibly due to the absence of questions about the individual's workplace in the digital twin's construction. We also observed that digital twin responses underestimated the variance present in the actual data, possibly suggesting that the digital twin reflects a weighted combination of the fine-tuned individual data and the pre-trained global LLM output.

Finally, while the digital twins did a modest job of capturing the QI scale, they failed to mimic responses to the two QI behavior questions. The digital twins tended to consistently select the normative, high-QI behavior much more frequently than humans did.

S10.18 Targeting Fairness

S10.18.1 Main Questions/Hypotheses

Companies commonly target their advertisements based on demographic characteristics, such as race or gender. Yet recent work has found that people often view the decision to target to be less fair than advertising broadly to the general population [94]. In this sub-study, we aim to replicate this pattern with human participants, and test whether their digital twins indicate similar fairness ratings.

S10.18.2 Methods

We opened the study to 400 human participants on Prolific, and 357 passed the screener and completed it. The study was subsequently run on their 357 digital twins, for a total of 714 participants. Participants and their corresponding digital twins were randomly assigned to the same condition in a two-cell (broad vs. targeted) between-subjects design.

All participants first read: "A snack foods company has developed a new line of snacks. Initial testing showed that, due to the taste and texture profile of the snacks, the snacks are better suited to the preferences of their female customers." In the broad condition, participants next read: "Even though they believe the snacks are best suited to their female customers, they will advertise the snacks broadly to the general public." In the targeted condition, participants next read: "Because they believe the snacks are best suited to their female customers, they will advertise the snacks to women directly." Finally, participants rated fairness: "How fair is this advertising plan?" ("Not at all fair" = 1; "Very fair" = 9).

S10.18.3 Results - Pre-registered Analyses

We conducted a two-way ANOVA with targeting condition (broad vs. targeted) and participant type (human vs. digital twin) as the independent variables, and fairness ratings as the dependent variable. We found a significant main effect of targeting condition ($F(1, 713) = 69.07,\ p < .001$), but no significant effects of participant type ($F(1, 713) = 0.05,\ p = .823$) or their interaction ($F(1, 713) = 1.57,\ p = .211$), indicating that, at an aggregate level, the digital twins rated fairness similarly to the humans. Pairwise comparisons confirm that for humans, the decision to target was rated as less fair ($M = 6.38,\ SD = 2.17$) than advertising broadly ($M = 7.47,\ SD = 1.83,\ F(1, 710) = 45.72,\ p < .001$), and for the digital twins, targeting was also rated as less fair ($M = 7.30,\ SD = 0.78$) than advertising broadly ($M = 6.50,\ SD = 0.80,\ F(1, 710) = 24.92,\ p < .001$).

S10.18.4 Results - Additional Analyses (Non-Preregistered)

Although not part of our pre-registered plan, a notable observation stood out in the analysis: the variance among the digital twins was lower than for the humans. In particular, for the digital twins, 99% of responses used the upper end of the scale (5–9), compared with only 87% of responses for the human participants ($\chi^2(1) = 25.27,\ p < .001$). Examining the responses closest to the mean (i.e., respondents

answering "7"), 63% of the digital twins provided that response, compared with 20% of human participants ($\chi^2(1) = 136.59,\ p < .001$). The overall correlation between human participants and their digital twin was weak but significant ($r = .13,\ p = .012$).

S10.18.5 Discussion

At the aggregate level, the digital twins closely matched human judgments of the perceived fairness of demographic targeting, replicating the finding that it is viewed as less fair than broad targeting and producing similar average ratings across conditions. However, the twins' responses were more clustered around the mean, whereas human participants were more likely to use the whole scale.

S10.19 User Behavior with Recommendation Systems

S10.19.1 Main Questions/Hypotheses

Modern machine learning and AI-based recommendation systems are data-driven, generating personalized recommendations from a user's previous interactions. Often, platforms make the assumption that a user's engagement with a particular piece of content is indicative of their utility for the content. However, a recent online behavioral experiment has shown that users may behave strategically and alter their engagement to influence the content they get recommended in the future [95]. For example, a user on a music platform may skip past a "guilty pleasure" song that they like, because they are worried that the recommendation algorithm will recommend too many similar songs later.

In this work, we study self-reported usage of online platforms, beliefs and preferences about recommendation systems, and strategization behavior among humans and their digital twins. We test whether digital twins exhibit similar self-reported behavior as their human counterparts.

S10.19.2 Methods

We recruited 598 human participants from Prolific and their digital twins. Of the human participants, 591 passed the attention check, resulting in 591 human subjects and 591 digital twins. Participants are then asked to self-report their usage, beliefs, and preferences regarding Netflix and Tiktok.

We assess knowledge about the platform's recommendation algorithm by asking "How do you think [platform] recommends content? Please check all that apply," where knowledge corresponds to selecting the answer "By analyzing what content you've interacted with in the past" from a menu of options.

We consider two types of strategization, with explicit user feedback and implicit user feedback:

- For strategization with explicit user feedback, we ask "When you are on [platform], do you give a thumbs-up (or thumbs-down) for any of the following reasons? Please check ALL that apply." We say that the user strategizes with explicit feedback if they check "Because you want [platform] to show you more (or fewer) content like it."

- For strategization with implicit user feedback, we ask "How do you typically react if [platform] shows you content or advertisements that you don't want to see in the future? Please check ALL that apply." We say that the user strategizes with implicit feedback if they answer "Scroll past it faster than I otherwise would" or "change how I interact with other content."

For preference for user controls in recommendation systems, we ask "What controls, if any, would you like to have over their recommendation systems? Please check ALL that apply" with a menu of options. We count the number of options selected other than "No control: let the algorithm work automatically."

S10.19.3 Results – Pre-registered Analyses

To analyze the differences between human participants and their AI-generated digital twins, we conducted a series of paired-samples t-tests for our pre-registered hypotheses. This statistical test compares the mean scores of two related groups—in this case, each human participant and their corresponding digital twin. This approach allows us to precisely assess systematic differences in their responses for two platforms: TikTok ($N = 234$) and Netflix ($N = 392$).

Platform Usage Patterns

Self-reported platform usage differed dramatically between humans and their digital twins. For TikTok, humans most frequently reported using the platform "a few hours every month" (35.5%) or "a few hours every week" (33.8%). In stark contrast, the majority of their digital twins reported "Never" using the platform (51.3%).

A similar, though less pronounced, pattern emerged for Netflix. The majority of human participants reported using Netflix "a few hours every week" (56.1%), whereas their digital twins were more evenly split between "a few hours every week" (60.2%) and "a few hours every day" (31.1%). While a paired t-test on the underlying ranked data indicated a significant difference (TikTok: $t(233) = -25.31,\ p < .001$; Netflix: $t(391) = -8.35,\ p < .001$), we emphasize that this test was performed on ordinal data.

Knowledge About Recommendation Algorithms

When asked if they believe platforms use past interactions to recommend content, digital twins were significantly more likely to endorse this idea than their human counterparts. For TikTok, digital twins ($M = 0.996,\ SD = 0.065$) endorsed this belief more strongly than humans ($M = 0.902,\ SD = 0.298,\ t(233) = -4.92,\ p < .001$). This finding was replicated on Netflix, where digital twins ($M = 0.997,\ SD = 0.051$) again were more likely to agree with this mechanism than humans ($M = 0.931,\ SD = 0.254,\ t(391) = -5.27,\ p < .001$). The correlations between digital twin and human responses were $r = 0.20$ and $r = 0.19$ for TikTok and Netflix, respectively.

Presence of Content Strategization

We measured strategic interaction with platform algorithms using two distinct questions. On both measures, digital twins reported engaging in significantly more strategic behavior.

First, when asked if they use "thumbs-up" or similar features to influence future recommendations, digital twins on TikTok ($M = 0.782,\ SD = 0.414$) reported doing so more than humans ($M = 0.530, SD = 0.500, t(233) = -8.86, p < .001$). The

effect was even more pronounced for Netflix, with twins ($M = 0.995$, $SD = 0.071$) reporting far more strategic use of this feature than humans ($M = 0.543$, $SD = 0.499$, $t(391) = -17.94$, $p < .001$). The correlations between digital twin and human responses were $r = 0.56$ and $r = 0.08$ for TikTok and Netflix, respectively.

Second, when asked how they react to undesirable content, digital twins were more likely to report taking strategic action (e.g., scrolling past faster, changing interaction patterns). This was true for TikTok, where twins ($M = 0.957$, $SD = 0.203$) scored higher than humans ($M = 0.509$, $SD = 0.501$, $t(233) = -13.77$, $p < .001$). The pattern held for Netflix, with twins ($M = 0.939$, $SD = 0.240$) again reporting more strategic reactions than humans ($M = 0.311$, $SD = 0.464$, $t(391) = -25.67$, $p < .001$). The correlations between digital twin and human responses were $r = 0.21$ and $r = 0.17$ for TikTok and Netflix, respectively.

Preference for User Controls

The largest divergence between the two groups was observed in their stated preference for controls over recommendation systems. Digital twins expressed a desire for a significantly greater number of controls than humans. On TikTok, twins ($M = 4.880$, $SD = 0.374$) selected far more control options than their human counterparts ($M = 1.991$, $SD = 1.352$, $t(233) = -32.49$, $p < .001$). The result was similarly stark for Netflix, where twins ($M = 4.888$, $SD = 0.361$) also desired significantly more controls than humans ($M = 1.862$, $SD = 1.248$, $t(391) = -47.50$, $p < .001$). The correlations between digital twin and human responses were $r = 0.11$ and $r = 0.10$ for TikTok and Netflix, respectively.

S10.19.4 Results - Additional Analyses (Non-Preregistered)

A key observation, consistent across all the metrics reported above, is that human responses are noisier than digital twin responses, with the latter consistently showing smaller standard deviations.

Additionally, we find differences in how humans and digital twins respond to social pressure, perhaps surprisingly with digital twins being more responsive to social pressure across both platforms.

We ask participants whether they "watch, listen to, or 'like' content that [they] do not particularly like just to be polite or support the creator." Humans reported low rates of this behavior on TikTok ($M = 0.11$, $SD = 0.25$) and Netflix ($M = 0.07$, $SD = 0.26$), compared to those of their digital twins on TikTok ($M = 0.25$, $SD = 0.44$) and Netflix ($M = 0.23$, $SD = 0.42$).

Similarly, we asked participants whether they click "like" content that [they] do not particularly like due to social pressure, and humans reported lower rates on TikTok ($M = 0.04$, $SD = 0.19$) and Netflix ($M = 0.04$, $SD = 0.19$) than their digital twins on TikTok ($M = 0.18$, $SD = 0.39$) and Netflix ($M = 0.14$, $SD = 0.35$).

S10.19.5 Discussion

We find that human and digital twin behavior differ significantly across a broad range of self-reported metrics on platform usage, knowledge about recommendation algorithms, strategization, preference for controls, and social behavior. The digital twins self report more platform usage, are more aware of how recommendation algorithms

work, are more likely to strategize, prefer more control over their algorithms, and are more prone to changing their behavior on recommendation systems due to social pressure.

REFERENCES

1. L. P. Argyle, E. C. Busby, N. Fulda, J. R. Gubler, C. Rytting, D. Wingate, Out of one, many: Using language models to simulate human samples. *Polit. Anal.* **31**, 337–351 (2023).

2. D. Dillion, N. Tandon, Y. Gu, K. Gray, Can AI language models replace human participants? *Trends Cogn. Sci.* **27**, 597–600 (2023).

3. A. Filippas, J. J. Horton, B. S. Manning, "Large language models as simulated economic agents: What can we learn from homo silicus" in *EC '24: Proceedings of the 25th ACM Conference on Economics and Computation* (2024), pp. 614-615

4. A. Ashokkumar, L. Hewitt, I. Ghezae, R. Willer, Large language models can predict the results of social science experiments. *Nature* **656**, 115–122 (2026).

5. C. R. Jones, B. K. Bergen, Large language models pass the turing test. arXiv:2503.23674 [cs. CL] (2025).

6. S. J. Westwood, The potential existential threat of large language models to online survey research. *Proc. Natl. Acad. Sci. U.S.A.* **122**, e2518075122 (2025).

7. Z. Cui, N. Li, H. Zhou, A large-scale replication of scenario-based experiments in psychology and management using large language models. *Nat. Comput. Sci.* **5**, 627–634 (2025).

8. D. Broska, M. Howes, A. van Loon, The mixed subjects design: Treating large language models as potentially informative observations. *Sociol. Methods Res.* **54**, 1074–1109 (2025).

9. S. Schröder, T. Morgenroth, U. Kuhl, V. Vaquet, B. Paaßen, Large language models do not simulate human psychology. arXiv:2508.06950 [cs.AI] (2025).

10. Y. Gao, D. Lee, G. Burtch, S. Fazelpour, Take caution in using LLMs as human surrogates. *Proc. Natl. Acad. Sci. U.S.A.* **122**, e2501660122 (2025).

11. J. Bisbee, J. D. Clinton, C. Dorff, B. Kenkel, J. M. Larson, Synthetic replacements for human survey data? The perils of large language models. *Polit. Anal.* **32**, 401–416 (2024).

12. Z. Lin, Six fallacies in substituting large language models for human participants. *Adv. Methods Pract. Psychol. Sci.* **8**, 25152459251357566 (2025).

13. S. Santurkar, E. Durmus, F. Ladhak, C. Lee, P. Liang, T. Hashimoto, “Whose opinions do language models reflect?” in *International Conference on Machine Learning* (PMLR, 2023), pp. 29971–30004.

14. F. Motoki, V. Pinho Neto, V. Rodrigues, More human than human: Measuring ChatGPT political bias. *Public Choice* **198**, 3–23 (2024).

15. A. Li, H. Chen, H. Namkoong, T. Peng, LLM generated persona is a promise with a catch. arXiv:2503.16527 [cs.CL] (2025).

16. A. Wang, J. Morgenstern, J. P. Dickerson, Large language models that replace human participants can harmfully misportray and flatten identity groups. *Nat. Mach. Intell.* **7**, 400–411 (2025).

17. J. S. Park, C. Q. Zou, J. Kamphorst, N. Egan, A. Shaw, B. M. Hill, C. Cai, M. R. Morris, P. Liang, R. Willer, M. S. Bernstein, Generative agent simulations of 1,000 people. arXiv:2411.10109 [cs.AI] (2024).

18. O. Toubia, G. Z. Gui, T. Peng, D. J. Merlau, A. Li, H. Chen, Database report: Twin-2K-500: A data set for building digital twins of over 2,000 people based on their answers to over 500 questions. *Market. Sci.* **44**, 1446–1455 (2025).

19. J. Ludwig, S. Mullainathan, A. Rambachan, Large language models: An Applied econometric framework. *Annu. Rev. Econ.* **18**, 283-316 (2026).

20. J. Hullman, D. Broska, H. Sun, A. Shaw, This human study did not involve human subjects: Validating llm simulations as behavioral evidence. arXiv:2602.15785 [cs.AI] (2026).

21. A. Kolluri, S. Wu, J. S. Park, M. S. Bernstein, Finetuning LLMs for human behavior prediction in social science experiments. arXiv:2509.05830 [cs.LG] (2025).

22. J. P. Beauchamp, D. Cesarini, M. Johannesson, E. Lindqvist, C. Apicella, On the sources of the height–intelligence correlation: New insights from a bivariate ace model with assortative mating. *Behav. Genet.* **41**, 242–252 (2011).

23. M. Binz, E. Akata, M. Bethge, F. Brändle, F. Callaway, J. Coda-Forno, P. Dayan, C. Demircan, M. K. Eckstein, N. Éltető, T. L. Griffiths, S. Haridi, A. K. Jagadish, L. Ji-An, A. Kipnis, S. Kumar, T. Ludwig, M. Mathony, M. Mattar, A. Modirshanechi, S. S. Nath, J. C. Peterson, M. Rmus, E. M. Russek, T. Saanum, J. A. Schubert, L. M. Schulze Buschoff, N. Singhi, X. Sui, M. Thalmann, F. J. Theis, V. Truong, V. Udandarao, K. Voudouris, R. Wilson, K. Witte, S. Wu, D. U. Wulff, H. Xiong, E. Schulz, A foundation model to predict and capture human cognition. *Nature* **644**, 1002–1009 (2025).

24. G. Gui, O. Toubia, The challenge of using LLMs to simulate human behavior: A causal inference perspective. arXiv:2312.15524 [cs.AI] (2025).

25. S. Gupta, V. Shrivastava, A. Deshpande, A. Kalyan, P. Clark, A. Sabharwal, T. Khot, "Bias runs deep: Implicit reasoning biases in persona-assigned LLMs" in *International Conference on Learning Representations* (2024), pp. 21849–21874.

26. J. Dodge, M. Sap, A. Marasović, W. Agnew, G. Ilharco, D. Groeneveld, M. Mitchell, M. Gardner, "Documenting large webtext corpora: A case study on the colossal clean crawled corpus" in *Proceedings of the 2021 Conference on Empirical Methods in Natural Language Processing* (Association for Computational Linguistics, 2021), pp. 1286–1305.

27. A. Zewail, A. Figueroa, J. Graham, M. Atari, Moral stereotyping in large language models. *Proc. Natl. Acad. Sci. U.S.A.* **123**, e2519941123 (2026).

28. T. Chen, C. Guestrin, "Xgboost: A scalable tree boosting system" in *Proceedings of the 22nd ACM SIGKDD International Conference on Knowledge Discovery and Data Mining* (Association for Computing Machinery, 2016), pp. 785–794.

29. N. Shahbazi, Y. Lin, A. Asudeh, H. V. Jagadish, Representation bias in data: A survey on identification and resolution techniques. *ACM Comput Surv* **55**, 1–39 (2023).

30. J. Henrich, S. J. Heine, A. Norenzayan, The weirdest people in the world? *Behav. Brain Sci.* **33**, 61–83 (2010).

31. G. Aher, R. I. Arriaga, A. T. Kalai, "Using large language models to simulate multiple humans and replicate human subject studies" in *International Conference on Machine Learning* (PMLR, 2023), pp. 337–371.

32. C. Camerer, G. Loewenstein, M. Weber, The curse of knowledge in economic settings: An experimental analysis. *J. Polit. Econ.* **97**, 1232–1254 (1989).

33. E. J. Johnson, D. Goldstein, Do defaults save lives? *Science* **302**, 1338–1339 (2003).

34. J. Huber, J. W. Payne, C. Puto, Adding asymmetrically dominated alternatives: Violations of regularity and the similarity hypothesis. *J. Consum. Res.* **9**, 90–98 (1982).

35. I. Simonson, Choice based on reasons: The case of attraction and compromise effects. *J. Consum. Res.* **16**, 158–174 (1989).

36. D. Pichert, K. V. Katsikopoulos, Green defaults: Information presentation and pro-environmental behaviour. *J. Environ. Psychol.* **28**, 63–73 (2008).

37. P. Briñol, M. J. McCaslin, R. E. Petty, Self-generated persuasion: Effects of the target and direction of arguments. *J. Pers. Soc. Psychol.* **102**, 925–940 (2012).

38. G. Pennycook, D. G. Rand, The psychology of fake news. *Trends Cogn. Sci.* **25**, 388–402 (2021).

39. G. Pennycook, Z. Epstein, M. Mosleh, A. A. Arechar, D. Eckles, D. G. Rand, Shifting attention to accuracy can reduce misinformation online. *Nature* **592**, 590–595 (2021).

40. T. Harcup, D. O'Neill, What is news? *Journal. Stud.* **18**, 1470–1488 (2017).

41. Reuters Institute, "Digital news report 2023" (Tech. Rep., University of Oxford, 2023); https://reutersinstitute.politics.ox.ac.uk/sites/default/files/2023-06/Digital_News_Report_2023.pdf.

42. R. Widjaya, S. Bestvater, A. Smith, "Who U.S. adults follow on TikTok" (Tech. Rep., Pew Research Center, 2024); www.pewresearch.org/data-labs/2024/10/08/who-u-s-adults-follow-on-tiktok/.

43. J. Berger, E. M. Schwartz, What drives immediate and ongoing word of mouth? *J. Market. Res.* **48**, 869–880 (2011).

44. J. Berger, K. L. Milkman, What makes online content viral? *J. Market. Res.* **49**, 192–205 (2012).

45. J. Berger, Arousal increases social transmission of information. *Psychol. Sci.* **22**, 891–893 (2011).

46. R. A. Emmons, M. E. McCullough, Counting blessings versus burdens: An experimental investigation of gratitude and subjective well-being in daily life. *J. Pers. Soc. Psychol.* **84**, 377–389 (2003).

47. D. DeSteno, Y. Li, L. Dickens, J. S. Lerner, Gratitude. *Psychol. Sci.* **25**, 1262–1267 (2014).

48. R. Oguni, C. Ishii, Gratitude promotes prosocial behavior even in uncertain situation. *Sci. Rep.* **14**, 14379 (2024).

49. M. Bukowski, A. Potoczek, K. Barzykowski, J. Lautenbacher, M. Inzlicht, What do we manipulate when reminding people of (not) having control? in search of construct validity. *Behav. Res. Methods* **56**, 3706–3724 (2024).

50. C. Y. Chen, L. Lee, A. J. Yap, Control deprivation motivates acquisition of utilitarian products. *J. Consum. Res.* **43**, 1031–1047 (2017).

51. C. Lembregts, M. Pandelaere, Falling back on numbers: When preference for numerical product information increases after a personal control threat. *J. Market. Res.* **56**, 104–122 (2019).

52. J. A. Whitson, A. D. Galinsky, Lacking control increases illusory pattern perception. *Science* **322**, 115–117 (2008).

53. S. Schnall, J. Roper, D. M. T. Fessler, Elevation leads to altruistic behavior. *Psychol. Sci.* **21**, 315–320 (2010).

54. L. C. Walsh, C. N. Armenta, G. Itzchakov, M. M. Fritz, S. Lyubomirsky, More than merely positive: The immediate affective and motivational consequences of gratitude. *Sustainability* **14**, 8679 (2022).

55. R. Oliveira, A. Baldé, M. Madeira, T. Ribeiro, P. Arriaga, The impact of writing about gratitude on the intention to engage in prosocial behaviors during the covid-19 outbreak. *Front. Psychol.* **12**, 588691 (2021).

56. K. H. Greenaway, S. A. Haslam, T. Cruwys, N. R. Branscombe, R. Ysseldyk, C. Heldreth, From “we” to “me”: Group identification enhances perceived personal control with consequences for health and well-being. *J. Pers. Soc. Psychol.* **109**, 53–74 (2015).

57. G. Sedek, M. Kofta, When cognitive exertion does not yield cognitive gain: Toward an informational explanation of learned helplessness. *J. Pers. Soc. Psychol.* **58**, 729–743 (1990).

58. D. M. Webster, A. W. Kruglanski, Individual differences in need for cognitive closure. *J. Pers. Soc. Psychol.* **67**, 1049–1062 (1994).

59. T. Zhang, V. Kishore, F. Wu, K. Q. Weinberger, Y. Artzi, BERTScore: Evaluating text generation with BERT. arXiv:1904.09675 [cs.CL] (2020).

60. A. V. Wilson, S. Bellezza, Consumer minimalism. *J. Consum. Res.* **48**, 796–816 (2022).

61. J. M. Jachimowicz, S. Duncan, E. U. Weber, E. J. Johnson, When and why defaults influence decisions: A meta-analysis of default effects. *Behav. Pub. Pol.* **3**, 159–186 (2019).

62. K. L. Keller, “Managing the growth tradeoff: Challenges and opportunities in luxury branding” in *Advances in Luxury Brand Management*, Journal of Brand Management: Advanced Collections, J.-N. Kapferer, J. Kernstock, T. Brexendorf, S. Powell, Eds. (Palgrave Macmillan, 2017), pp. 179–198.

63. S. Bellezza, A. Keinan, Brand tourists: How non–core users enhance the brand image by eliciting pride. *J. Consum. Res.* **41**, 397–417 (2014).

64. E. Park, K. Lane, S. Bellezza, H. A. Chen, G. Eckhardt, R. Hamilton, Eds., "NFT for conspicuous consumption" in *Advances in Consumer Research*, H. A. Chen, G. Eckhardt, R. Hamilton, Eds. (Association for Consumer Research, 2022), vol. 50.

65. Human Rights Watch. EU Parliament approves supply chain law. News release, Human Rights Watch (2024); www.hrw.org/news/2024/04/24/eu-parliament-approves-supply-chain-law [accessed: 27 September 2025].

66. P. J. Curran, S. G. West, J. F. Finch, The robustness of test statistics to nonnormality and specification error in confirmatory factor analysis. *Psychol. Methods* **1**, 16–29 (1996).

67. The White House. The president's initiative on junk fees and related pricing practices (2022). https://www.presidency.ucsb.edu/documents/white-house-press-release-the-presidents-initiative-junk-fees-and-related-pricing.

68. A. M. McCright, R. E. Dunlap, C. Xiao, Perceived scientific agreement and support for government action on climate change in the USA. *Clim. Change* **119**, 511–518 (2013).

69. F. Dell'Acqua, B. Kogut, P. Perkowski, Super Mario meets AI: Experimental effects of automation and skills on team performance and coordination. *Rev. Econ. Stat.* **107**, 951–966 (2025).

70. J. B. Kessler, C. Low, C. D. Sullivan, Incentivized resume rating: Eliciting employer preferences without deception. *Am. Econ. Rev.* **109**, 3713–3744 (2019).

71. G. Rusak, B. S. Manning, J. J. Horton, AI agents can enable superior market designs (2025).

72. Y. Zhang, R. Gosline, Human favoritism, not AI aversion: People's perceptions (and bias) toward generative AI, human experts, and human–GAI collaboration in persuasive content generation. *Judgm. Decis. Mak.* **18**, e41 (2023).

73. S. Lewandowsky, S. van der Linden, Countering misinformation and fake news through inoculation and prebunking. *Eur. Rev. Soc. Psychol.* **32**, 348–384 (2021).

74. G. Pennycook, D. G. Rand, Who falls for fake news? The roles of bullshit receptivity, overclaiming, familiarity, and analytic thinking. *J. Pers.* **88**, 185–200 (2020).

75. G. Ceylan, I. A. Anderson, W. Wood, Sharing of misinformation is habitual, not just lazy or biased. *Proc. Natl. Acad. Sci. U.S.A.* **120**, e2216614120 (2023).

76. S. Vosoughi, D. Roy, S. Aral, The spread of true and false news online. *Science* **359**, 1146–1151 (2018).

77. J. J. Van Bavel, S. Rathje, M. Vlasceanu, C. Pretus, Updating the identity-based model of belief: From false belief to the spread of misinformation. *Curr. Opin. Psychol.* **56**, 101787 (2024).

78. B. K. Orme, K. Chrzan, *Becoming an Expert in Conjoint Analysis: Choice Modeling for Pros* (Sawtooth Software, 2017).

79. L. Boussioux, J. N. Lane, M. Zhang, V. Jacimovic, K. R. Lakhani, The crowdless future? Generative AI and creative problem-solving. *Organ. Sci.* **35**, 1589–1607 (2024).

80. B. C. Lee, J. J. Chung, An empirical investigation of the impact of ChatGPT on creativity. *Nat. Hum. Behav.* **8**, 1906–1914 (2024).

81. J. A. Olson, J. Nahas, D. Chmoulevitch, S. J. Cropper, M. E. Webb, Naming unrelated words predicts creativity. *Proc. Natl. Acad. Sci. U.S.A.* **118**, e2022340118 (2021).

82. O. Toubia, J. Berger, Optimally sequencing semantic search predicts creativity. *PLOS ONE* **21**, e0352328 (2026).

83. K. Gray, S. Anderson, E. E. Chen, J. M. Kelly, M. S. Christian, J. Patrick, L. Huang, Y. N. Kenett, K. Lewis, “Forward flow”: A new measure to quantify free thought and predict creativity. *Am. Psychol.* **74**, 539–554 (2019).

84. R. Catapano, Z. L. Tormala, D. D. Rucker, Perspective taking and self-persuasion: Why "putting yourself in their shoes" reduces openness to attitude change. *Psychol. Sci.* **30**, 424–435 (2019).

85. A. Alesina, P. Giuliano, "Preferences for redistribution" in *Handbook of Social Economics*, J. Benhabib, A. Bisin, M. O. Jackson, Eds. (North-Holland, 2011), Vol. 1, Ch. 4, pp. 93–131.

86. M. Gilens *Affluence and Influence: Economic Inequality and Political Power in America* (Princeton Univ. Press, 2012).

87. M. A. Hussein, C. Lee, S. C. Wheeler, How do consumers react to ads that meddle in out-party primaries? *J. Consum. Res.* **51**, 1186–1208 (2025).

88. G. Heltzel, K. Laurin, Polarization in america: Two possible futures. *Curr. Opin. Behav. Sci.* **34**, 179–184 (2020).

89. Pew Research Center, As partisan hostility grows, signs of frustration with the two-party system (2022); www.pewresearch.org/politics/2022/08/09/as-partisan-hostility-grows-signs-of-frustration-with-the-two-party-system/ [accessed 25 September 2025].

90. M. A. Hussein, Z. L. Tormala, Undermining your case to enhance your impact: A framework for understanding the effects of acts of receptiveness in persuasion. *Pers. Soc. Psychol. Rev.* **25**, 229–250 (2021).

91. M. A. Hussein, S. C. Wheeler, Reputational costs of receptiveness: When and why being receptive to opposing political views backfires. *J. Exp. Psychol. Gen.* **153**, 1425–1448 (2024).

92. P. E. Green, V. Srinivasan, Conjoint analysis in consumer research: Issues and outlook. *J. Consum. Res.* **5**, 103–123 (1978).

93. C. J. Frank, P. F. Magnone, O. Netzer, *Decisions Over Decimals: Striking the Balance Between Intuition and Information* (John Wiley & Sons, 2022).

94. F. Shaddy, E. M. S. Friedman, O. Toubia, Fairness perceptions in demographic targeting. *J. Consum. Res.* **53**, 22–47 (2026).

95. S. H. Cen, A. Ilyas, J. Allen, H. Li, A. Madry, “Measuring strategization in recommendation: Users adapt their behavior to shape future content” in *Proceedings of the 25th ACM Conference on Economics and Computation* (Association for Computing Machinery, 2024), pp. 203–204.
